# Cavity-driven attractive interactions in quantum materials

**DOI:** 10.1038/s41586-026-10609-1

**Published:** 2026-05-27

**Authors:** F. Helmrich, H. S. Adlong, M. Kroner, I. Khanonkin, G. Scalari, J. Faist, A. İmamoğlu, T. F. Nova

**Affiliations:** 1 Institute for Quantum Electronics, ETH Zürich, Zürich, Switzerland; 2 Institute for Theoretical Physics, ETH Zürich, Zürich, Switzerland

**Keywords:** Quantum fluids and solids, Sub-wavelength optics, Polaritons, Terahertz optics, Quantum optics

## Abstract

Many-body phenomena in quantum materials emerge from the interplay among a broad continuum of electronic states and controlling these interactions is critical for engineering new phases. One promising approach exploits light confined within optical cavities to tailor electronic properties^1^. Here we demonstrate that terahertz cavity photons can mediate attractive interactions in a tunable van der Waals (vdW) material and reorganize a continuum of electron–hole transitions into an exciton-like state. We introduce a broadband, sub-wavelength time-domain microscope that integrates exfoliated, dual-gated 2D quantum materials into a terahertz cavity. This approach enables the spectroscopic measurement of the field-tunable bandgap of bilayer graphene^2^ (BLG) in the terahertz range and, at resonance, reveals ultrastrong coupling^3^ (USC) with an effective interaction strength exceeding *g*/*ω*_c_ ≈ 40% of the bare photon energy. Crucially, we identify a cavity-induced resonance emerging from the interband continuum that resembles Coulomb-bound excitons and remains stable across a broad temperature range. Our findings propose an experimental platform for designing and investigating hybrid light–matter phases in 2D quantum matter.

## Abstract (web summary)

A broadband, sub-wavelength time-domain microscope is described, enabling the observation that terahertz light trapped within a cavity can mediate attractive interactions in a tunable van der Waals material.

## Main

The control of quantum materials through light has grown into a notable research direction in materials science and condensed matter physics^4^. At the forefront of modern research is the vision that ground-state properties of materials could be modified through interaction with cavity-confined electromagnetic fields^1^. When material excitations couple to these photonic modes, hybrid light–matter states known as polaritons can emerge. In the USC regime, in which interaction strengths rival excitation energies, these hybrid systems are predicted to exhibit markedly altered macroscopic properties, with ground states that contain virtual light and matter excitations^3^. Theory suggests that such coupling may control superconductivity^5^, trigger ferroelectric phase transitions^6,7^, stabilize superradiant excitonic insulators^8^ or control intertwined orders^9^. Experimental studies further underscore the impact of confined fields: in solid-state systems, cavity coupling has tuned metal–insulator transitions^10^ and non-perturbatively altered transport in both integer and fractional quantum Hall phases^11,12^. Yet, experimental evidence for cavity-mediated control of quantum phases in ground or excited states remains scarce.

**Fig. 1:**
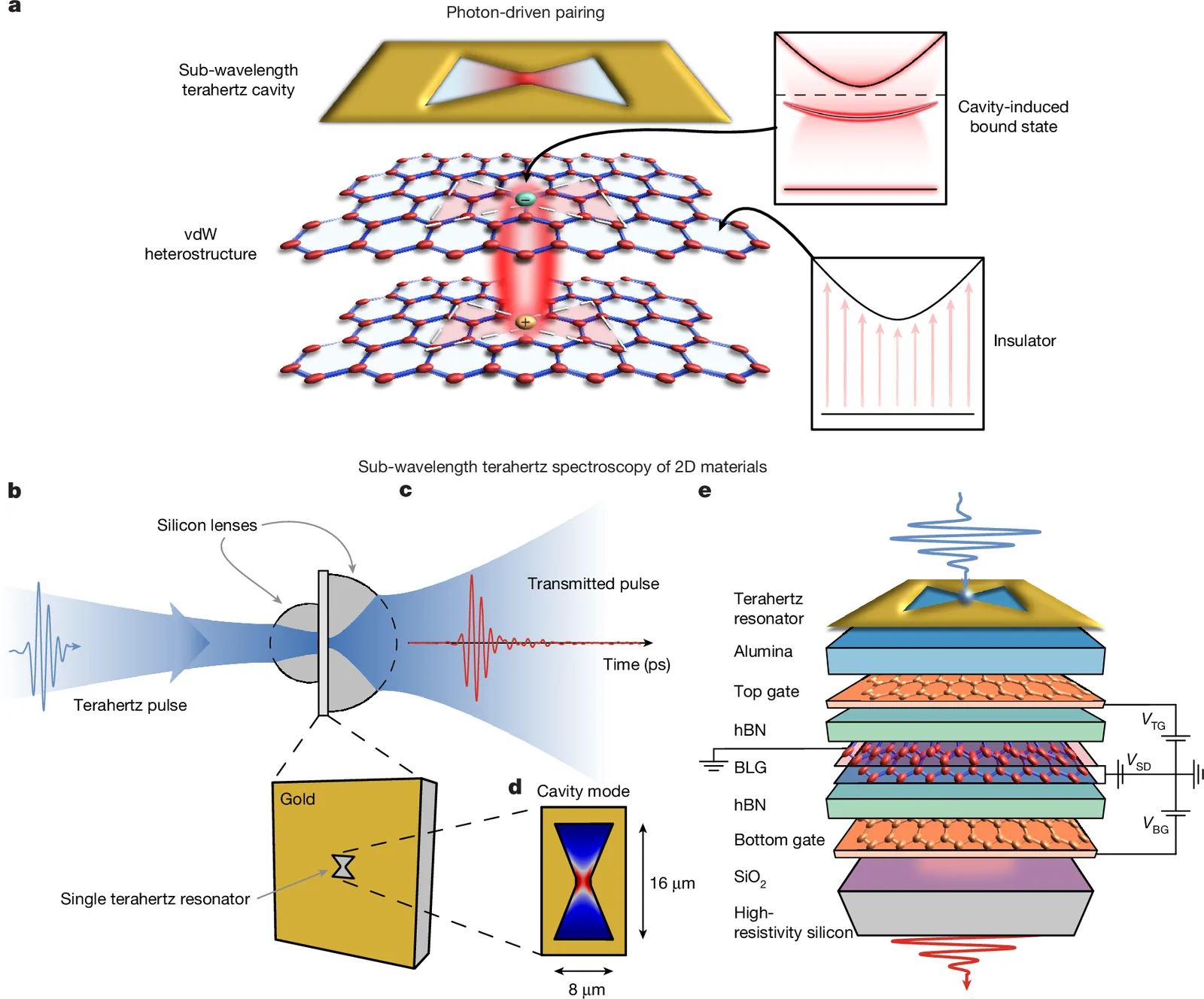
Sub-wavelength terahertz spectroscopy of cavity-induced bound states. **a**, Concept of cavity-induced pairing. A 2D insulating material is coupled to a sub-wavelength terahertz cavity. In the insulator, a broad continuum of interband electron–hole transitions from the valence band to the conduction band underpins its optical response. When the cavity is tuned to resonance with the bandgap of the material, the localized cavity field acts as an effective pairing interaction that binds electron–hole pairs across a wide momentum range, reorganizing the continuum into a discrete bound state. **b**, Schematic of the terahertz spectroscopy set-up. A few-cycle terahertz pulse (blue line) is focused on a 2D heterostructure with a bow-tie resonator and solid immersion lenses. **c**, Terahertz pulse transmitted through the lens–resonator assembly, as detected by electro-optic sampling (red line). **d**, In-plane electric field of the resonant cavity mode calculated using finite-element methods. The field is concentrated at the apex of the bow tie. **e**, Lateral view of the sample structure. The heterostructure consists of BLG, few-layer graphite gates and hBN dielectrics placed on a high-resistivity Si/SiO_2_ substrate. The device is insulated from the bow-tie resonator using alumina dielectric.

Here, drawing on predictions^13–15^ and experiments^16,17^ in quantum well systems, we aim to demonstrate that electromagnetic fields confined within a terahertz cavity can mediate an effective attractive interaction among charged particles and drive the emergence of a bound state from a continuum of electron–hole transitions, leading to a profound reshaping of the excitation spectrum in a vdW material (Fig. 1a).

To reach this goal, we have developed an experimental platform specifically designed to overcome the limitations that have hindered progress in exploring light–matter coupling and spectroscopy in microstructured quantum materials. Our strategy focuses on vdW materials, which exhibit naturally enhanced light–matter coupling—thanks to reduced dimensionality—and a rich variety of quantum and correlated phases, ranging from superconductivity^18^ and Wigner–Mott states^19,20^ to exotic magnetism^21^ and fractional Chern insulators^22^. Crucially, when carrier density and displacement field are controlled independently through dual electrostatic gating, several phases can often be accessed within a single device, making these systems particularly suitable for investigating light–matter interactions across a broad range of many-body phenomena.

A common strategy to realize USC is to boost the light–matter interaction strength by confining the electromagnetic field within a cavity mode that is much smaller than the photon wavelength^23^. However, integrating 2D materials into such cavities poses substantial technical challenges, especially because ultrastrong light–matter coupling typically occurs near collective excitations in the terahertz range. Although sub-wavelength localization of terahertz fields can be efficiently achieved using microstructured metallic resonators, conventional approaches rely on metasurfaces of repeating cavities placed on top of the material of interest^24^. This method is incompatible with exfoliated 2D materials, which are usually limited to in-plane dimensions of just a few microns.

Recent advancements have enabled spectroscopy of individual metallic resonators by adapting solid immersion lenses to the terahertz regime^25^. Building on this concept, we have developed a method that simultaneously delivers the broadband frequency operation essential for quantum materials spectroscopy^26^ and strong field confinement, all while maintaining compatibility with gate-tunable vdW devices.

## Experimental set-up and material system

In our set-up (Fig. 1c), a free-space terahertz beam is tightly focused using a high-refractive-index, transparent hyper-hemispherical silicon immersion lens. The beam impinges on a high-resistivity silicon substrate coated with gold, except for a broadband bow-tie resonator. This resonator steers the field onto a spot smaller than 2 microns, as represented by the cavity mode in Fig. 1d. The transmitted terahertz field (Fig. 1c), with a bandwidth ranging from 1 to 6 THz (4 to 24 meV), is then recollimated by a secondary hemispherical immersion silicon lens and detected using conventional far-field methods (see Methods and Supplementary Information Section 6.1). Crucially, only the field that traverses the resonator focus is transmitted, enabling sub-wavelength, broadband terahertz spectroscopy of micron-sized samples positioned beneath the antenna (Fig. 1e). The cavity gap size is chosen to maximize field confinement and spatial resolution, while effectively mitigating nonlocal effects^27^.

We chose Bernal-stacked BLG as our material platform because electrostatic gating allows its low-energy electronic structure to be continuously tuned into the terahertz range. While Pristine BLG is a semimetal with quasi-parabolic bands, electrostatic doping drives it into a metallic regime and a perpendicular displacement field opens a bandgap of controllable size^28^. As a result, interband transitions^2^ and excitonic features^29^ can be tuned smoothly from the mid-infrared down to terahertz energies, making BLG ideally matched to the bandwidth of our set-up.

The device (Fig. 1e) consists of BLG encapsulated between two insulating hexagonal boron nitride (hBN) layers and is fully tunable with two few-layer graphite gates, allowing independent control of the charge density *n* and out-of-plane displacement field *D*.

The stack is completed by the terahertz resonator, which is electrically insulated from the heterostructure and its electrical connections by means of an alumina layer (see Methods and Supplementary Information Section 1 for fabrication details).

## Transport characteristics and terahertz transmission

**Fig. 2:**
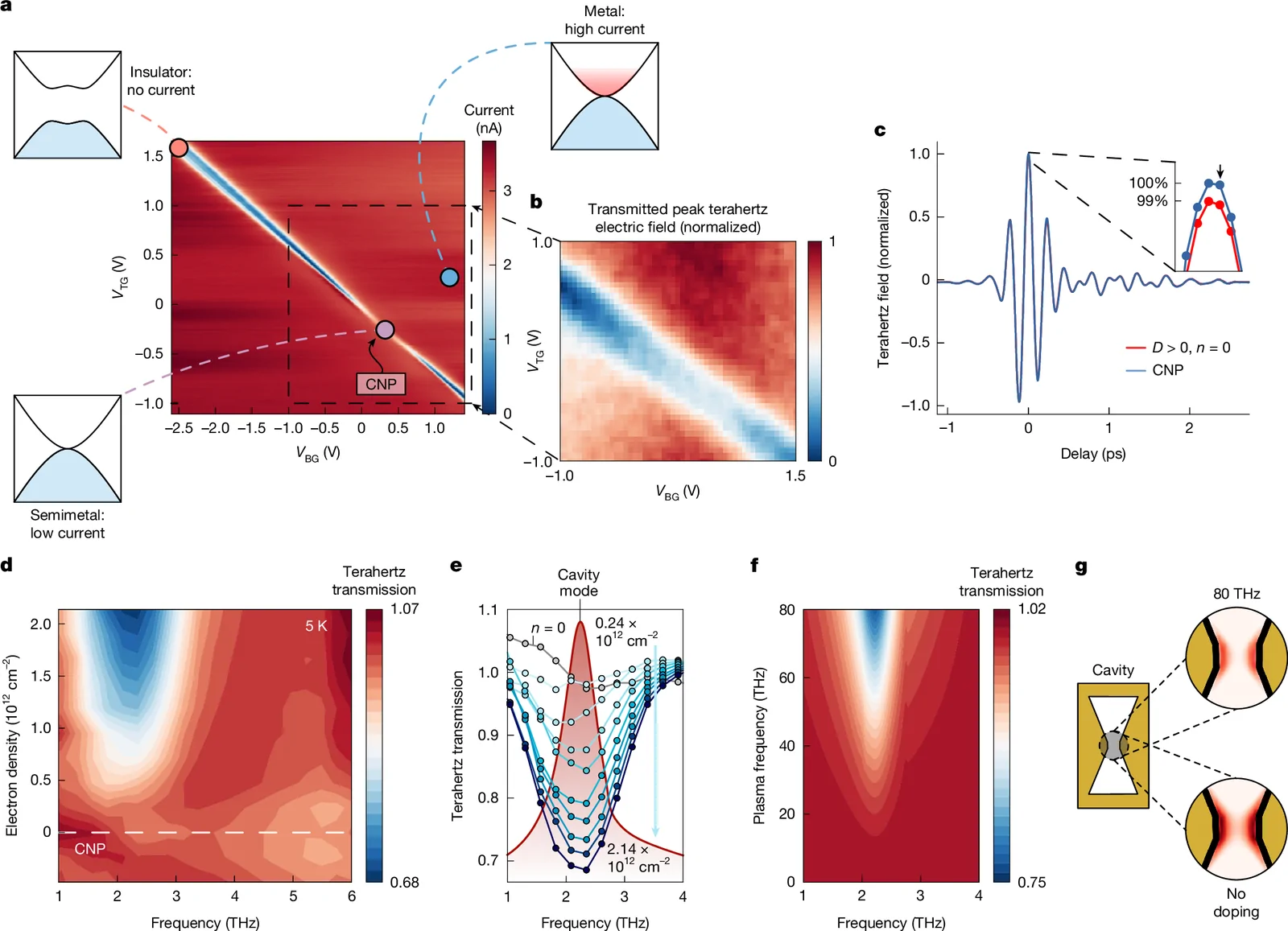
Gate-tuned transport and terahertz cavity response of BLG. **a**, Dual-gate-dependent two-point transport measurement of BLG. The coloured dots indicate key points: the CNP (purple), the insulating phase at which the terahertz reference spectrum is recorded (red, at *D* = −0.38 V nm^−1^) and a representative metallic state (blue). **b**, Normalized terahertz field transmission at a fixed time delay, with varying *V*_TG_ and *V*_BG_. The chosen time delay corresponds to the terahertz field maximum (indicated by the black arrow in panel **c** inset). Note that this single-time-delay measurement does not provide any spectral information, which can only be obtained by measuring the full time-domain trace for each (*V*_BG_, *V*_TG_) pair. As shown in Supplementary Information Section 6.5, the general trends observed depend only weakly on the chosen time delay. **c**, Transmitted terahertz pulses when BLG is at the CNP (blue) and in an insulating state with |*D*| > 0 (red). The inset shows a closer view of the maximum of the waveforms. Both traces are normalized to the maximum of the blue curve. All measurements were taken at 5 K. **d**, Contour plot of terahertz transmission spectra of BLG as a function of electron density *n* at *D* = 0. All spectra are normalized to the insulating state at *D* = −0.38 V nm^−1^. The measurements were taken at 5 K. **e**, Line cuts of the spectra in panel **d** showing the dip in transmission. The red line represents the bare cavity transmission spectrum, calculated using finite-element methods. The grey line represents the referenced transmission spectrum for *n* = 0, while the coloured lines represent the spectra for increasing electron densities (from lighter to darker blue). **f**, Finite-element simulations of the transmission spectra of the bow-tie resonator coupled to a conductive 2D material modelled by a Drude conductivity with variable plasma frequency. The spectra are referenced to the bare cavity. **g**, Simulated electric-field mode profiles for different densities. The mode profiles are evaluated at the cavity resonance for a plasma frequency of 0 THz and 80 THz.

The gate-voltage-dependent two-point conductance of our device at 5 K is shown in Fig. 2a. Along the diagonal defined by *V*_TG_ = −*αV*_BG_, the carrier density remains near zero while the displacement field *D* increases. Here *V*_TG_ and *V*_BG_ are the voltages applied to the top and bottom gates, respectively, and the lever arm *α* is given by the gate capacitances (Methods). The *D*-induced inversion-symmetry breaking between the two graphene layers opens a bandgap and results in the conductance minimum characteristic of the insulating state. By contrast, along *V*_TG_ = *αV*_BG_, the carrier density *n* increases while no bandgap is opened, and the system becomes metallic. At the charge-neutrality point (CNP), at which *n* = 0 and *D* = 0, BLG remains semimetallic (purple dot in Fig. 2a).

Figure 2c shows the terahertz pulse transmitted through the sample when the device is biased at the CNP (blue line). Tuning the device away from the CNP results in reproducible (Supplementary Fig. 3) changes in the transmitted terahertz field at the percent level. A representative trace measured in the insulating phase (*D* ≠ 0 and *n* = 0) is shown by the red line in the same figure. To visualize this gate dependence more systematically, Fig. 2b presents the terahertz transmission as a function of gate voltage, measured at a fixed time delay (black arrow in the inset of Fig. 2c). The transmission map closely resembles the transport data in Fig. 2a, with a clear charge-neutrality line that tracks the insulating region with nearly the same slope and intercept, albeit with a substantially larger width. This agreement shows that the terahertz signal is sensitive to the electronic state of BLG while remaining comparatively insensitive to other elements of the stack, including the charged gates. We attribute this selectivity to the reduced charge mobility in the non-encapsulated gates, which suppresses their Drude-like response relative to that of the high-mobility BLG.

## Doping-induced cavity-mode suppression

To gain insight into the optical properties of the coupled BLG/cavity system, we measured the spectrally resolved terahertz transmission of doped BLG. Figure 2d shows the terahertz spectra measured for different charge densities with *n* ≠ 0 and *D* = 0. Each spectrum is referenced to the transmission taken for BLG in an insulating state (*D* = −0.38 V nm^−1^ and *n* = 0; red dot in Fig. 2a), in which the bandgap exceeds the terahertz bandwidth of our set-up and the material becomes transparent for terahertz radiation. Effectively, our data are normalized to the transmission spectrum of the bare cavity (Supplementary Information Section 6.1). As the electron density increases, a pronounced dip in the terahertz transmission emerges around 2.3 THz. The dip coincides with the peak position of the simulated cavity transmission spectrum (Fig. 2e), consistent with observations in ref. ^30^. Carrier injection increases both absorption and, by means of a larger impedance mismatch, reflectance of the BLG layer. This enhances damping, broadens the linewidths (Supplementary Information Section 6.3) and reduces coupling of the resonator to the external field, thereby lowering transmission. The coupled system acts as a terahertz attenuator around 2.3 THz with modulation depths of around 30% in power for relatively low doping. We performed finite-element simulations modelling the electromagnetic interaction between our bow-tie resonator and a conductive 2D material described by a Drude model (see Methods and Supplementary Information Section 9). By varying the plasma frequency, the simulations reproduce the experimentally observed transmission dip at the cavity resonance (Fig. 2f) and reveal a reduced mode intensity at higher carrier densities (Fig. 2g), corroborating the role of carrier-induced mode suppression.

## USC and cavity-driven pairing

**Fig. 3:**
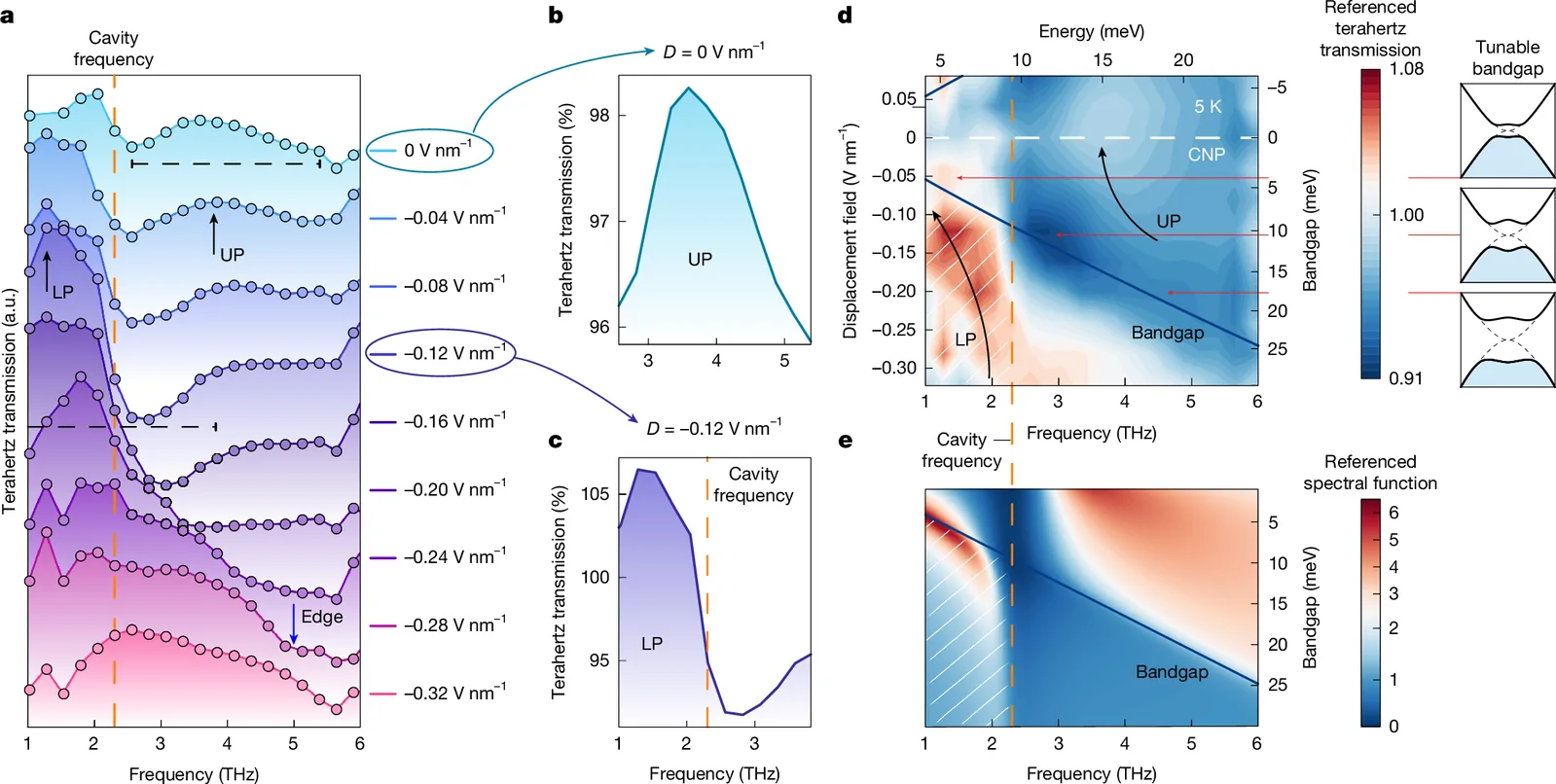
Observation of polariton formation. **a**, BLG transmission spectra at various displacement fields (*D*) under zero doping (*n* = 0) measured at 5 K. All spectra are referenced to the insulating state at *D* = −0.36 V nm^−1^ and vertically offset for clarity. The cavity resonance frequency is indicated by a dashed orange line. A blue arrow points to the closing bandgap edge and black arrows highlight the LP and UP branches. **b**, Detail of the *D* = 0 V nm^−1^ curve from panel **a** (the frequency range selected is shown by the dashed line in panel **a**). The axes are rescaled to exclude the LP contribution and isolate the UP peak. **c**, Detail of the *D* = −0.12 V nm^−1^ curve from panel **a**. The axes are rescaled to highlight the LP peak (the frequency range selected is shown by the dashed line in panel **a**). **d**, Contour plot representation of the spectra in panel **a**. The blue line represents the bandgap calculated using the self-consistent Hartree model for screening from ref. ^28^ and the dashed line marks the CNP position. **e**, Calculated spectral function as a function of bandgap. The spectral function is referenced to that of the bare cavity. The hatched areas in panels **d** and **e** represent the regions below the cavity frequency and below the band edge at which the bound state is expected. a.u., arbitrary units.

**Extended Data Fig. 1:**
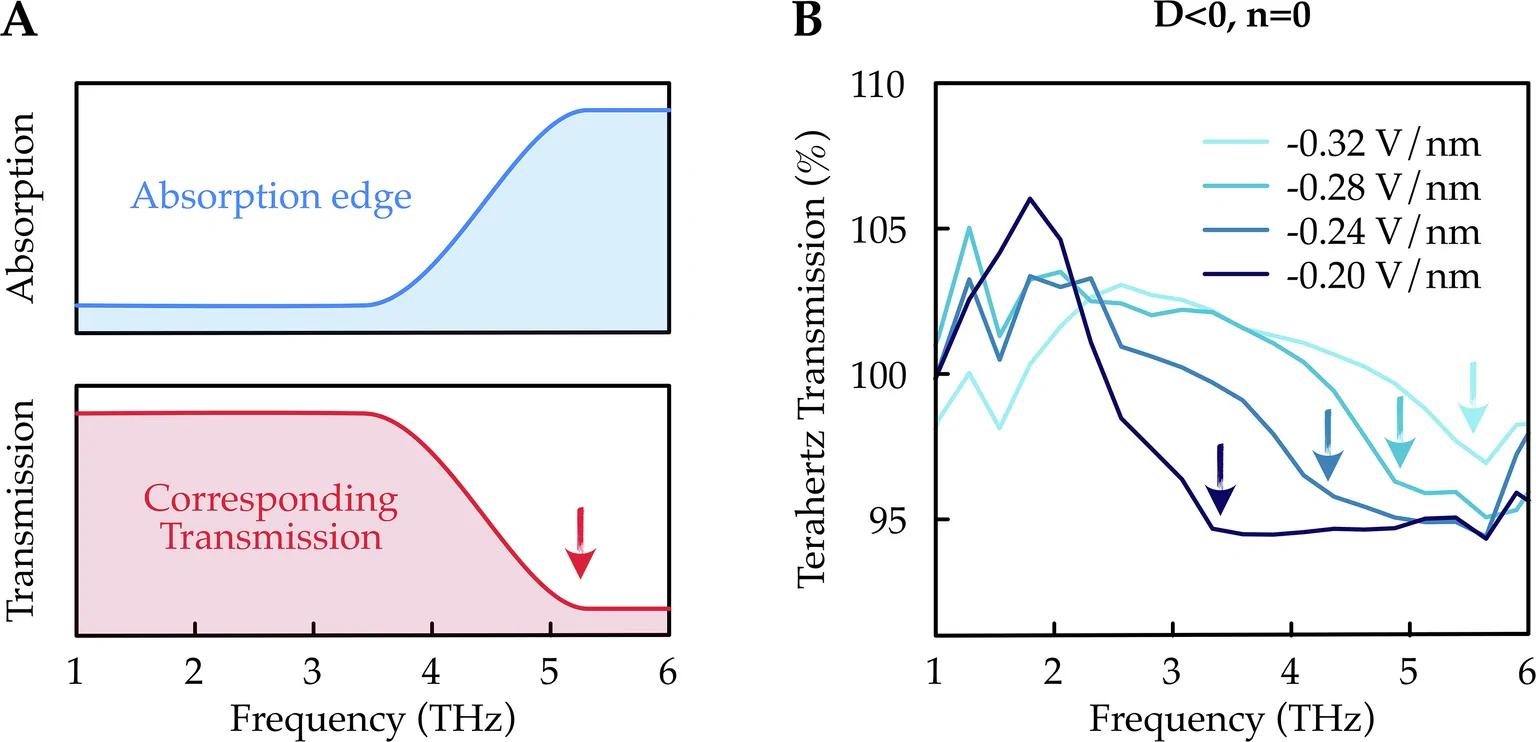
Band-edge absorption. **a**, Schematic of THz absorption by a band edge (top) and the corresponding transmission spectrum (bottom), with an arrow marking the location of the band edge. **b**, Experimental transmission spectra at various displacement fields |*D*| > 0 and *n* = 0 referenced to the insulating state *D* = −0.36. The arrows mark the onset of absorption, similarly to panel **a**. Data obtained from device D1 at 5 K.

The referenced transmission spectra as a function of displacement field *D* ≠ 0 for *n* = 0 are shown in Fig. 3a (line cuts) and Fig. 3d (*D* versus frequency map). At large negative displacement fields (*D* ≪ 0), the bandgap of insulating BLG is larger than our spectral window. For decreasing fields, an absorption edge emerges and shifts from higher to lower frequencies (blue arrow in Fig. 3a and Extended Data Fig. 1). This feature is consistent with the field-tunable bandgap of BLG, measured here spectroscopically for the first time in this frequency range. The calculated bandgap field dependence (see also Methods and Supplementary Information Section 11) is shown in Fig. 3d (blue line), in excellent agreement with the measured edge.

**Extended Data Fig. 2:**
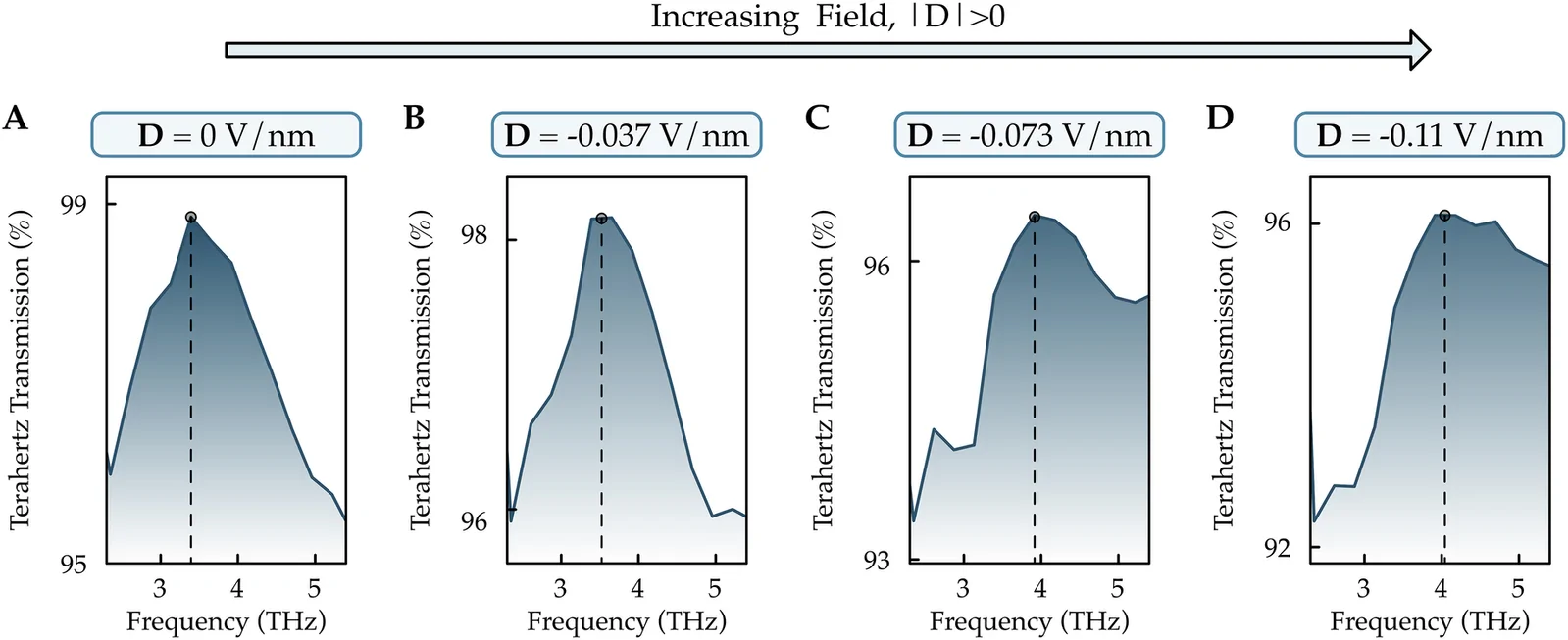
UP peak as a function of displacement field. The data are adapted from Fig. 5a. The frequency window is restricted to 2.3–5.4 THz (that is, from just above the cavity frequency up to near the upper end of our spectral window), thereby removing the LP contribution and excluding the noisy high-frequency tails. The vertical axis is rescaled independently for each *D* (min-to-max within the shown frequency window) to highlight the UP peak. The dashed lines highlight the peak position. **a**, *D* = 0 V nm^−1^. **b**, *D* = −0.037 V nm^−1^. **c**, *D* = −0.073 V nm^−1^. **d**, *D* = −0.11 V nm^−1^.

As the gap approaches the cavity frequency with further decreasing fields, the spectrum undergoes substantial changes. Two dispersive peaks develop: a strong, narrow peak below and a weak, broad peak above the cavity frequency (Fig. 3a–d). These modes correspond to the lower polariton (LP; Fig. 3c) and upper polariton (UP; Fig. 3b and Extended Data Fig. 2), forming around a deepening spectral gap (the blue dip in Fig. 3d).

This behaviour is characteristic of a strongly coupled light–matter system in which a cavity mode interacts with a matter excitation. As the two modes are tuned through resonance, an avoided crossing develops, leading to hybrid modes—the polaritons. A simple analogy to a (classical) Fabry–Pérot cavity coupled to a Lorentz oscillator reproduces the salient features, highlighting the generality of this response (see Methods and Supplementary Information Section 7). The estimated coupling strength, defined as the normalized light–matter interaction *η* = *g*/*ω*_c_ ≈ 40% (Supplementary Information Section 7.2), places our system well into the USC regime (*η* > 10% (ref. ^3^)).

We note that, in conventional USC, the observed behaviour arises from coupling a cavity mode to a pre-existing discrete matter resonance, such as an exciton in interband systems^31,32^. By strong contrast, our system couples the cavity field to a continuum of interband electron–hole transitions^33^, for which individual oscillator strengths are weak and linewidths are broad, such that well-defined polaritonic modes are not generally expected.

The very emergence of polaritons in our experiment therefore suggests a fundamentally different mechanism: as predicted in ref. ^13^, the cavity field can act as an effective pairing interaction that binds electron–hole pairs across a broad momentum range. This photon-mediated interaction reorganizes the continuum into a discrete state: the LP (black line in Fig. 3d) emerges as a bound-like state ‘pulled out’ of the continuum (the hatched area below the gap and cavity lines in Fig. 3d), whereas in the UP, the cavity mode hybridizes with higher-energy transitions (black line in Fig. 3d). Such a continuum-to-discrete reorganization departs substantially from standard USC physics and illustrates the profound ability of cavity electromagnetic fields to mediate attractive interactions in 2D materials.

**Fig. 4:**
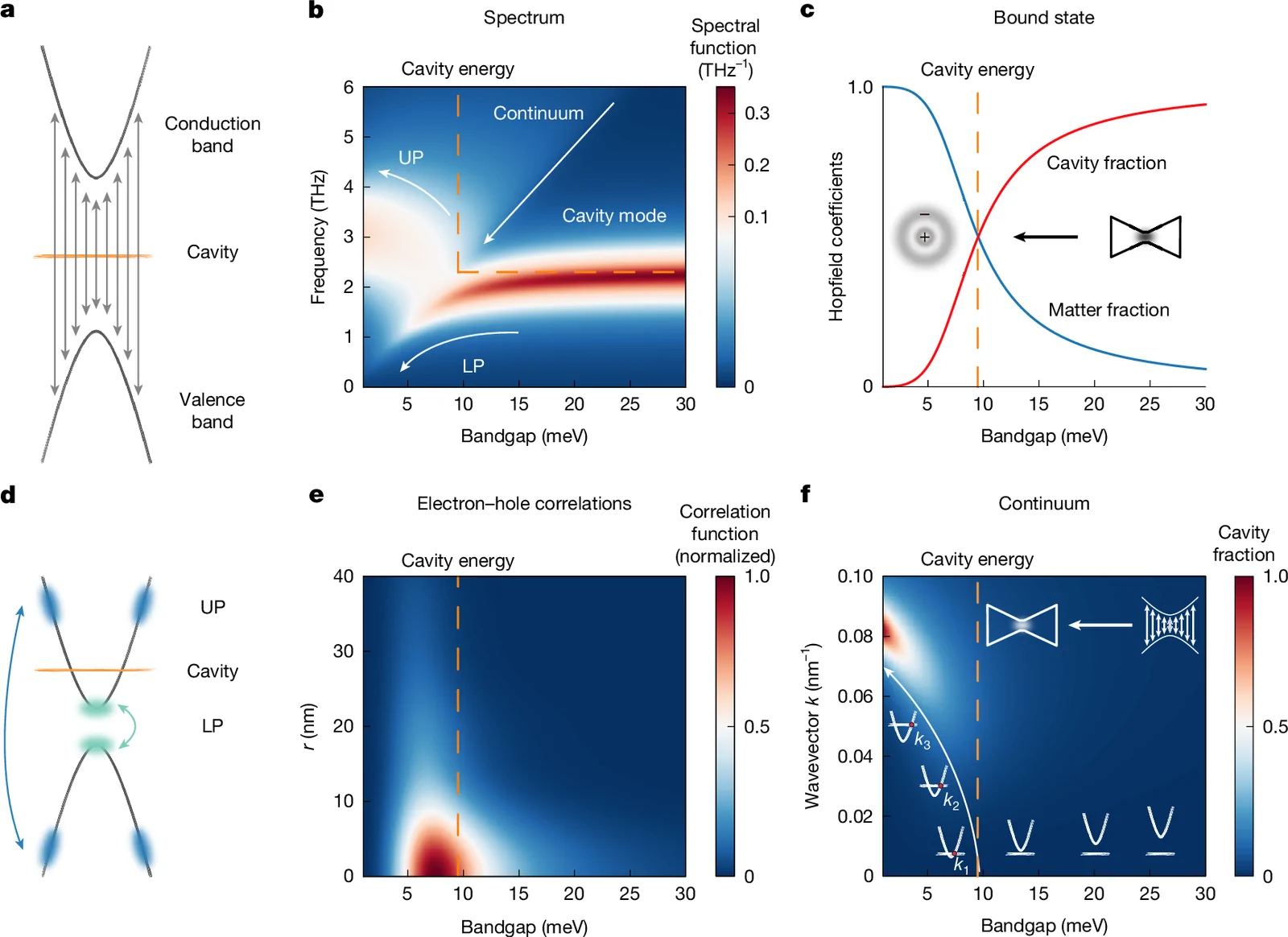
Theory. **a**, Schematic representation of the model. A continuum of electron–hole excitations interacts with a single cavity mode. **b**, Calculated spectral function for the ultrastrongly coupled system as a function of the bandgap. Note that only the (physical) spectrum for positive frequencies is shown. For negative frequencies, the spectrum exhibits a qualitatively similar behaviour (see Supplementary Information Section 8). Note that the quantity shown here is identical to that in Fig. 3e except that it is not normalized by the bare cavity spectrum. **c**, Cavity and integrated matter content of the bound state as a function of gap energy. **d**, Schematic of polariton formation. The LP is mainly formed by states at the band edge, whereas the UP has its weight at higher electronic momenta. **e**, Electron–hole correlation function *γ*(*r*) of the bound state as a function of electron–hole distance *r* and bandgap energy. The scale has been normalized to the maximum. **f**, The cavity content of the continuum states is plotted as a function of bandgap and momentum *ħk*, in which *ħ* is the reduced Planck constant and *k* is the electronic wavevector. The white line marks the momentum at which the dispersionless cavity mode intersects the parabolic electron–hole dispersion.

To support this interpretation, we have developed a quantum mechanical model (see Methods and Supplementary Information Section 8) in which the 2D conduction and valence bands are approximated by parabolic dispersions separated by a tunable gap. In this framework, each electronic momentum represents a potential electron–hole transition that couples to the cavity field with rate *g* (Fig. 4a). Notably, by neglecting Coulomb interactions, any bound-state formation can be attributed solely to the photon-mediated interaction. Our USC-compatible model reproduces the main experimental spectral features (Fig. 3e) and offers deeper insight into the underlying physics.

We first analyse the photonic spectral function *A*(*ω*) (Fig. 4b), which encodes the linear response of the coupled system to an external light field. For bandgaps much larger than the cavity frequency, the system consists of a discrete, bound cavity mode and a continuum of electronic transitions above the gap. As the bandgap decreases, the cavity mode hybridizes with the continuum, leading to two polariton branches. Focusing on the lowest-energy mode, which is initially predominantly photonic, we observe its gradual transformation into the LP as the gap approaches the cavity frequency. This evolution is clearly reflected in the Hopfield coefficients^34^ (Fig. 4c), which capture the cavity and matter content of the LP state and reveal a shift from a predominantly cavity-dominated state to one that is largely matter-like. Notably, as the hybridization with the continuum occurs, the emerging state retains the inherent bound character imparted by the cavity photons. Note that the matter component of the LP is mainly composed of electron–hole transitions near the band edge, corresponding to low-momentum states (Fig. 4d).

To further investigate the nature of this emergent bound state, we calculate the electron–hole correlation function *γ*(*r*) as a function of bandgap (Fig. 4e), which measures the probability of finding a valence-band hole at a distance *r* from a conduction-band electron. As the bandgap approaches the cavity frequency, the correlation function develops a pronounced short-range enhancement, signalling the formation of a bound electron–hole pair. This behaviour sharpens the central point of our interpretation: the phenomenology of Fig. 3 can only be reproduced classically (Supplementary Information Section 7) by considering the interaction of the cavity with a discrete bound state. Yet, even for bandgaps well above the cavity frequency^29^ (Extended Data Fig. 1), our experiment reveals no discrete resonance, including conventional Coulomb-bound excitons. We attribute their absence to the very small binding energies and weak oscillator strengths expected at the vanishingly small bandgaps relevant here—for which the exciton Bohr radius can exceed tens of nanometres^35,36^—together with strong Coulomb screening by the nearby gates, located approximately 20 nm from the BLG layer. Within our quantum model, *γ*(*r*) therefore makes the origin of the bound state explicit: photon-mediated interactions in the cavity bind electrons and holes together even when bare Coulomb interactions alone would not produce a bound exciton.

Finally, we consider the UP, whose matter component is predominantly formed by high-energy transitions involving large-momentum states (Fig. 4d). As the bandgap is reduced below the cavity frequency, these continuum states become progressively dressed by cavity photons, as illustrated in Fig. 4f. We emphasize that the persistence of the UP at *D* = 0, in both experiment and theory, does not imply that the bound state survives in this limit. Rather, it is the expected continuation of the cavity-like upper branch, entirely analogous to Landau polariton systems, in which the UP also remains visible as *B* → 0 (ref. ^24^).

## Temperature dependence

**Fig. 5:**
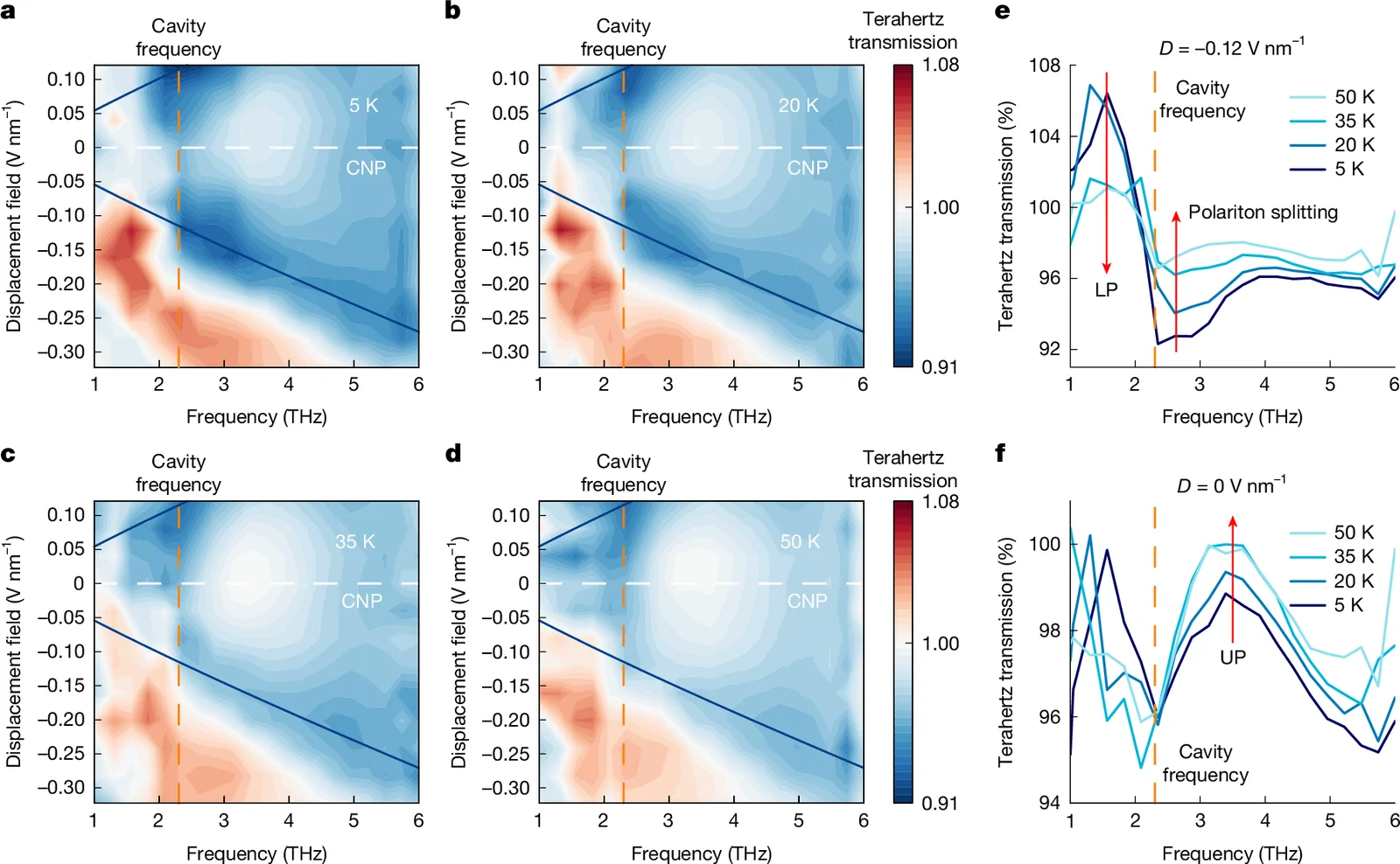
Temperature dependence. **a**–**d**, Contour plots of the transmission spectra of BLG for varying *D* and *n* = 0 measured at 5 K (**a**), 20 K (**b**) 35 K (**c**) and 50 K (**d**). All spectra are normalized to the spectrum of the insulating phase obtained at *D* = −0.38 V nm^−1^ at the respective temperature. The calculated bandgap is represented by a solid blue line, whereas a dashed white line indicates the CNP. **e**,**f**, Line cuts extracted from the spectra presented in panels **a**–**d** at *D* = 0.12 V nm^−1^ (**e**) and at *D* = 0 V nm^−1^ (**f**).

Figure 5 shows the temperature evolution of the polariton spectra. As the temperature increases, the spectral contrast decreases: the LP and the spectral dip become progressively less pronounced (Fig. 5e), whereas the UP becomes relatively more prominent (Fig. 5f). This trend is consistent with enhanced dissipation and thermally activated carriers across the gap. These effects preferentially wash out the lower-energy features associated with states near the band edge, whereas the higher-energy UP remains comparatively less affected (Fig. 4d).

Notably, the anticrossing remains visible up to 100 K (see Fig. 5 and Supplementary Information Section 4.2), a regime in which *k*_B_*T* exceeds *g* and is comparable with the bare cavity energy *ħω*_c_. This shows that increasing temperature reduces the spectral contrast without destroying the underlying light–matter hybridization over the measured range, underscoring the robustness of photon-mediated pairing. Such behaviour is consistent with previous work indicating that USC signatures can persist at elevated temperature at which the thermal energy exceeds the light–matter coupling strength as long as the transition dipole strength is preserved and the cavity and matter resonances do not qualitatively change character^37,38^.

## Discussion

We have shown that terahertz cavities can be used to generate attractive interactions in quantum materials. Our newly developed far-infrared spectroscopy platform for microstructured vdW heterostructures reveals two clearly distinct regimes. Off resonance, it measures the intrinsic terahertz bandgap of BLG, providing direct access to the bare material response. On resonance, by contrast, light–matter hybridization takes over and the measured spectrum reflects the coupled system itself. For applications that require spectroscopy without cavity hybridization, the same architecture can operate in a ‘spectroscopy-only’ mode by increasing the spacer between the resonator and the vdW stack (from the present roughly hundreds of nanometres to several microns)^12^, thereby reducing the light–matter coupling while preserving sub-wavelength focusing and spatial resolution.

In this broader context, our platform overcomes key limitations of existing terahertz spectroscopic approaches for microstructured devices, which often suffer from bandwidth constraints or incompatibility with dual gating. Although on-chip terahertz techniques have proved to be highly effective in examining the ultralow-energy electrodynamics of 2D materials^39^, they face inherent bandwidth limitations^40^, typically not exceeding 1.5 THz or 6 meV, as well as a lack of independent tuning of charge density and displacement field. Yet, many of the most relevant excitations in quantum and vdW materials lie at energies of several to tens of meV, including phonons^41^, Mott gaps and electronic bands in moiré systems^42^, excitonic insulator binding energies^43^ and the gaps and collective excitations of Wigner crystals^44^. With a bandwidth exceeding 20 meV, our method extends well beyond this range and bridges the gap between on-chip terahertz approaches and photoconductivity measurements^29^, while also providing robust in situ referencing, time and spatial resolution and direct access to the full optical response of the material through amplitude-resolved and phase-resolved transmission. It also offers a notable advantage over terahertz scanning-probe methods, which can reach nanometric spatial resolution but remain difficult to combine with full dual gating and typically require extensive calibration, together with detailed modelling of the probe–sample interaction, to extract reliable spectroscopic information^45^.

More generally, our approach is versatile and can be combined with different terahertz generation schemes and cavity geometries, making it scalable across a wide spectral range from the terahertz to the mid-infrared.

Beyond spectroscopy, our platform opens a route to cavity quantum electrodynamics studies in 2D materials with a key advantage unique to vdW systems: in situ tunability. Cavity-induced effects have been controlled by changing the resonance conditions^10^ or the material phase^11,12^. Here electrostatic control provides access to both within the same device, allowing us to bring excitations on and off resonance with the cavity while driving the material into and out of a given phase. This makes it possible to track how USC depends on, and in turn reshapes, the underlying order within each phase and across phase transitions.

Although our observation of photon-mediated bound states is based on linear-response terahertz spectroscopy and therefore cannot be taken as direct evidence of ground-state pairing, it raises a broader question: can cavity-driven pairing extend to ground-state collective phenomena? Theory predicts that cavity coupling can reshape superconducting instabilities and critical temperatures^5,46^. Our architecture provides a natural setting for exploring these ideas in tunable 2D platforms. Particularly promising systems include gate-defined superconductors in moiré graphene^18^ and magnetic-field-tuned phases in Bernal BLG^47^, in which tailored light–matter coupling could be used to control, and potentially enhance, superconducting phase transitions.

More immediately, in the present system, our findings raise fundamental questions about the nature of the cavity-controlled ground state. The appearance of excitonic features at finite bandgaps prompts us to ask how cavity-mediated correlations evolve as the gap closes. The highly localized, non-uniform cavity fields used here provide a powerful test bed for exploring theoretical proposals and constraints on equilibrium cavity-induced ordering^8,48,49^. Although uniform-field no-go theorems hinder equilibrium superradiance, these constraints may be relaxed when the cavity field is strongly spatially inhomogeneous^8^, as in our sub-wavelength terahertz resonator. If an equilibrium superradiant-like instability were to occur in this regime, it would be expected to manifest as a gate-tunable softening of the lowest hybrid mode towards zero frequency, together with the onset of a spontaneous coherent cavity-field amplitude at *D* = 0. If the ordered phase also developed an inversion-odd electronic or structural component, symmetry-sensitive nonlinear probes such as second-harmonic generation could provide a complementary diagnostic. Together with spectroscopic signatures of mode softening, such measurements could help assess whether cavity-mediated interactions can stabilize ordered phases with excitonic-insulator character^43,48^.

Looking ahead, integrating our technique with dilution refrigerators operating at millikelvin base temperatures is the next frontier. By delivering low-power, low-photon-energy terahertz radiation directly to the sample, our method avoids the heating typically associated with optical laser pulses in on-chip terahertz set-ups. This capability would enable broadband far-infrared spectroscopy and cavity control of 2D materials under ultralow-temperature conditions, opening a route to examining how cavity fields reshape correlated phases and their collective excitations, from superconducting gaps^18,47^ and Wigner crystal modes^44^ to fractional excitons in fractional Chern insulators^50^.

## Methods

### Sample fabrication and mounting

BLG and hBN flakes were exfoliated from bulk crystals onto SiO_2_/Si substrates; for device D1, few-layer graphite flakes were also used as gates. The flakes were assembled into dual-gated vdW heterostructures by polymer-based dry stacking and transferred onto high-resistivity Si/SiO_2_ chips. Electrical contacts were patterned by optical lithography and formed by Cr/Au deposition using edge-contact and surface-contact geometries; in device D2, the top and bottom gates were instead defined as ultrathin evaporated Cr films. The completed heterostructures were electrically insulated from the resonator plane by an alumina spacer thicker than 140 nm grown by atomic layer deposition. A complementary bow-tie resonator was then fabricated on top of the device by a further round of optical lithography and Ti/Au evaporation (approximately 200 nm total thickness). To protect the device during lens mounting, we patterned an SU-8 support layer (1–2 μm) on the finished chip. Finally, the chip was mounted between silicon solid-immersion lenses in a custom copper holder and wire-bonded for electrical access. More fabrication details are provided in Supplementary Information Section 1.

### Electrostatics of dual-gated BLG heterostructures

The electrostatics of dual-gated vdW heterostructures can be modelled within the framework of a simple plate capacitor model. The electron density is then given by

1 $$n=\frac{{{\epsilon }}_{{\rm{hBN}}}{{\epsilon }}_{0}}{e}\left(\frac{{V}_{{\rm{TG}}}}{{d}_{{\rm{TG}}}}+\frac{{V}_{{\rm{BG}}}}{{d}_{{\rm{BG}}}}\right)$$

and the out-of-plane displacement field

2 $$D=\frac{{{\epsilon }}_{{\rm{hBN}}}{{\epsilon }}_{0}}{2}\left(\frac{{V}_{{\rm{TG}}}}{{d}_{{\rm{TG}}}}-\frac{{V}_{{\rm{BG}}}}{{d}_{{\rm{BG}}}}\right).$$

Here *V*_TG(BG)_, *d*_TG(BG)_, *e*, *ϵ*_hBN_ and *ϵ*_0_ denote the top and bottom gate voltages, the corresponding hBN layer thicknesses, the elementary charge, the hBN dielectric constant ≈ 3.4 (ref. ^51^) and the vacuum permittivity, respectively. Note that, for simplicity, we include the shift of charge neutrality from 0 V owing to residual doping in the definition of *V*_BG_ and *V*_TG_ (see Supplementary Information Section 6.4 for details). The lever arm *α* is then given by

3 $$\alpha =\frac{{d}_{{\rm{TG}}}}{{d}_{{\rm{BG}}}}.$$

### Terahertz time-domain spectroscopy

Terahertz time-domain spectroscopy measurements were performed using few-cycle terahertz pulses generated by optical rectification of infrared pulses from a fibre oscillator in the organic crystal N-benzyl-2-methyl-4-nitroaniline (BNA; see Supplementary Information Section 3 for details). The transmitted terahertz electric field was recorded directly in the time domain by electro-optic sampling in optically contacted GaP and frequency-domain spectra were obtained by Fourier transformation of the measured waveforms. For transmission measurements, the sample was mounted between Si solid-immersion lenses and placed in a continuous-flow cryostat equipped with terahertz-transparent windows.

### Transport measurements

We access the DC transport characteristics by two-point source–drain current measurements at fixed bias using DC source meters (see also Supplementary Information Section 2). The sample is mounted in the same cryostat as for terahertz spectroscopy.

### Second device

To assess device-to-device reproducibility, we fabricated a second BLG device (D2) using thin metallic chromium gates (4 nm) instead of graphite. Apart from the increased disorder associated with the rougher metal–hBN interface, D2 is otherwise closely similar to the main device (D1): the terahertz resonator has the same nominal dimensions and is positioned at a comparable distance from the BLG. As shown in Supplementary Figs. 15 and 9, transport and terahertz transmission measurements on D2 exhibit the same qualitative features as those observed in D1, demonstrating that our main observations are robust against device variability and persist even in the more disordered sample.

### On plasmon absorption

Plasmonic modes in doped BLG can, in principle, absorb terahertz radiation^39,52–54^. However, at fixed wavevector, their frequency is expected to follow the characteristic square-root density dependence, *ω*_pl_ ∝ √*n* (ref. ^55^), which is not observed in our data (Fig. 2d,e). Moreover, using this model together with the dimensions of our cavity, we find that the corresponding plasmon modes should remain below the cavity frequency over the full density range explored here (Supplementary Information Section 10). We therefore conclude that plasmon absorption does not account for the observed features.

### Quantum model of a cavity mode coupled to electron–hole continuum

To describe the coupling between a terahertz cavity mode and interband excitations in BLG, we use the Hamiltonian (see refs. ^14,15^ and Supplementary Information Section 8 for details)

4 $$H=\hbar {\omega }_{{\rm{c}}}{a}^{\dagger }a+\sum _{{\bf{k}}}{{\epsilon }}_{c,{\bf{k}}}{c}_{{\bf{k}}}^{\dagger }{c}_{{\bf{k}}}+\sum _{{\bf{k}}}{{\epsilon }}_{v,{\bf{k}}}{v}_{{\bf{k}}}^{\dagger }{v}_{{\bf{k}}}+\hbar \sum _{{\bf{k}}}g(a+{a}^{\dagger })({v}_{{\bf{k}}}^{\dagger }{c}_{{\bf{k}}}+{c}_{{\bf{k}}}^{\dagger }{v}_{{\bf{k}}}),$$

in which *ω*_c_ is the cavity frequency, *g* is the light–matter coupling strength and **k** is the electronic wavevector. We model the low-energy conduction and valence bands by parabolic dispersions separated by a tunable bandgap *E*_g_,

5 $${{\epsilon }}_{{\bf{k}}}^{c}=-{{\epsilon }}_{{\bf{k}}}^{v}+{E}_{{\rm{g}}}=\frac{{\hbar }^{2}{k}^{2}}{2m}$$

with effective mass *m* ≈ 0.04*m*_e_. Here *a*^(†)^, *c*^(†)^ and *v*^(†)^ denote photon, conduction-band electron and valence-band electron annihilation (creation) operators, respectively. Within the dilute-excitation bosonic approximation for interband electron–hole coherences, defined by

6 $${b}_{{\bf{k}}}^{\dagger }={c}_{{\bf{k}}}^{\dagger }{v}_{{\bf{k}}},$$

the Hamiltonian becomes

7 $$H\approx {{\epsilon }}_{{\rm{c}}}{a}^{\dagger }a+\sum _{{\bf{k}}}{{\epsilon }}_{{\bf{k}}}{b}_{{\bf{k}}}^{\dagger }{b}_{{\bf{k}}}+\hbar \sum _{{\bf{k}}}g(a+{a}^{\dagger })({b}_{{\bf{k}}}+{b}_{{\bf{k}}}^{\dagger }),$$

with *ϵ*_c_ = *ħω*_c_. We diagonalize equation (7) using a Hopfield–Bogoliubov transformation^14,34,56^, which yields both the polariton energies and the corresponding eigenstates.

The eigenenergies satisfy the secular equation

8 $${{\epsilon }}^{2}-{{\epsilon }}_{{\rm{c}}}^{2}-2{{\epsilon }}_{{\rm{c}}}\Sigma ({\epsilon })=0,$$

in which the photonic self-energy is

9 $$\Sigma ({\epsilon })=\sum _{{\bf{k}}}\left(\frac{{\hbar }^{2}{g}^{2}}{{\epsilon }-{{\epsilon }}_{{\bf{k}}}}-\frac{{\hbar }^{2}{g}^{2}}{{\epsilon }+{{\epsilon }}_{{\bf{k}}}}\right).$$

The experimentally relevant quantity is the photonic spectral function

10 $${\mathcal{A}}({\epsilon })=-\frac{1}{\pi }{\rm{Im}}{\mathcal{G}}({\epsilon }),$$

in which ${\mathcal{G}}$ is the retarded photonic Green’s function in the frequency domain (see Supplementary Information Section 8). The electron–hole correlation function of the LP state is defined as

11 $$\mathop{\gamma }\limits^{ \sim }(r)={\langle {c}_{r}^{\dagger }{c}_{r}{v}_{0}{v}_{0}^{\dagger }\rangle }_{{\rm{LP}}}-{\langle {c}_{r}^{\dagger }{c}_{r}{v}_{0}{v}_{0}^{\dagger }\rangle }_{{\rm{vac}}},$$

in which *r* is the electron–hole distance, the electronic operators are transformed into real space and |vac⟩ is the ground state of the coupled system.

### Relation to Fano physics

The Fano problem, in its most general form, describes a discrete excitation coupled to a continuum of states^57^. In the conventional weak-coupling limit, this interaction does not create a new branch of excitations. Instead, it modifies a single resonance: the mode is shifted in energy and acquires extra linewidth from decay into the continuum, as encoded in the real and imaginary parts of the self-energy Σ(*ϵ*). Depending on how the system is examined, this can produce the characteristic asymmetric Fano line shape (for example, in the scattering cross-section).

Our system (defined in equation (7)) belongs to the same general class of discrete–continuum Hamiltonians but operates in a qualitatively different regime. Here the light–matter coupling is sufficiently strong that the continuum can no longer be treated as a small perturbation of the cavity mode. Because the continuum is semi-infinite and its band edge can be tuned through the cavity resonance, hybridization can push one branch below the continuum threshold. Once this happens, decay into continuum states is no longer possible for that mode. Its linewidth is then set only by its cavity-mode content and can, in principle, even become narrower than the bare cavity linewidth. The lower branch is therefore not a broadened cavity resonance but a discrete bound state, whereas a second bright hybrid branch remains at higher energy and leads to the continuum-broadened UP response.

The essential distinction is therefore between a weak-coupling Fano regime, with a single shifted and broadened resonance, and the strong-coupling regime relevant here, with two hybrid branches separated by an avoided crossing. In Supplementary Information Section 8.1, we show this crossover explicitly by calculating the photonic spectral function while varying *g*: for small coupling, the continuum produces only the conventional Fano-like reshaping of the cavity line, whereas at the experimental coupling strength, the spectrum develops distinct LP and UP branches.

### Transfer-matrix simulations

To complement the quantum model, we perform classical transfer-matrix simulations^58^ for a Fabry–Pérot cavity with the active material placed at the field antinode (see Supplementary Information Section 7). In this approach, the material response is specified a priori through a dielectric function *ϵ*(*ω*).

When *ϵ*(*ω*) is modelled by a Lorentz oscillator, corresponding to a discrete resonance, the calculated referenced transmission reproduces the observed asymmetric polariton dispersion and avoided crossing. By contrast, when *ϵ*(*ω*) is modelled only as a semi-infinite absorption edge, as expected for bare interband transitions, the spectra show only Fano-like reshaping of the cavity line and no polariton formation.

This contrast reflects a basic limitation of transfer-matrix simulations: because *ϵ*(*ω*) is fixed by construction, the cavity cannot reshape the continuum. The bound state in our system arises precisely from such cavity-induced redistribution of spectral weight within the electron–hole continuum. This effect is captured by the quantum model but not by continuum-only transfer-matrix calculations. Accordingly, the measured anticrossing can be reproduced within transfer-matrix simulations only by introducing a discrete resonance by hand, consistent with a cavity-induced exciton-like bound state.

### Finite-element simulations

To model the electromagnetic response of the bow-tie resonator, we perform frequency-domain finite-element simulations using COMSOL Multiphysics. These calculations provide both the spatial field distribution and the transmission spectrum (see Supplementary Information Section 9 for details).

### Self-consistent calculation of the displacement-field-induced bandgap

We calculate the BLG bandgap *E*_g_ as a function of the applied displacement field *D* using the self-consistent model of ref. ^59^, which accounts for the finite polarizability and screening of the bilayer system. Further details are given in Supplementary Information Section 11.

## Online content

Any methods, additional references, Nature Portfolio reporting summaries, source data, extended data, supplementary information, acknowledgements, peer review information; details of author contributions and competing interests; and statements of data and code availability are available at https://doi.org/10.1038/s41586-026-10609-1.

## Supplementary information

Supplementary Information Supplementary Sections 1–11 including Supplementary Figs. 1–26 and Supplementary references.

Peer Review file

## Data Availability

The numerical simulations and measurement datasets underlying the figures in this paper are available from the corresponding author on request. The raw data that support the findings of this article are also available in the ETH Research Collection (https://doi.org/10.3929/ethz-c-000797558).

## Code availability

The code used in the theoretical part of this study is available from the corresponding author on request and in the ETH Research Collection (http://doi.org/10.5905/ethz-1007-932).
