# Supplementary material for: Cavity-driven attractive interactions in quantum materials
Source: Nature. 2026 May 27;654(8118):361–8. doi: 10.1038/s41586-026-10609-1 (PMC13661254; doi:10.1038/s41586-026-10609-1)
Supplement: Supplementary file 1 [file 41586_2026_10609_MOESM1_ESM.pdf]

nature portfolio

https://doi.org/10.1038/s41586-026-10609-1

Supplementary information

Cavity-driven attractive interactions in quantum materials

In the format provided by the
authors and unedited

Supplementary Information:
Cavity-Driven Attractive Interactions in Quantum Materials

F. Helmrich[1], H. S. Adlong[1,2], M. Kroner[1], I. Khanonkin[1], G. Scalari[1], J. Faist[1], A. İmamoğlu[1], and T. F. Nova[1]

[1]Institute for Quantum Electronics, ETH Zürich, Auguste-Piccard-Hof 1, 8093 Zürich, Switzerland

[2]Institute of Theoretical Physics, ETH Zürich, Wolfgang-Pauli-Strasse 27, 8093 Zürich, Switzerland

Contents

1 Sample Fabrication **3**

2 Cryogenic sample characterization **6**

3 Terahertz Time-Domain Spectroscopy **7**

4 Additional data device D1 **9**

4.1 Evolution of the UP with D and T . . . 9

4.2 Temperature-dependent data . . . 10

4.3 Impact of doping on polariton formation . . . 11

4.4 Extended doping-dependent data . . . 11

5 THz spectroscopy data of device D2 **12**

6 Data analysis **14**

6.1 THz time-domain data . . . 14

6.2 Band-edge absorption . . . 15

6.3 Doping-dependence of cavity linewidth . . . 15

6.4 Transport . . . 17

6.4.1 Device D1 . . . 17

6.4.2 Device D2 . . . 17

6.5 Single time-delay data . . . 20

7 Fabry-Pérot cavity **21**

7.1 Model 21

7.2 Fitting of polariton energies 23

7.3 Connection to the Fano effect 25

7.4 Transfer-matrix simulations 27

8 Quantum model for ultrastrong light-matter coupling **28**

8.1 Weak- and strong-coupling limits of the discrete–continuum model 33

9 Finite-element simulations **36**

10 Plasmon dispersion in dual-gated BLG devices **37**

11 Self-consistent calculation of field-induced bandgap **38**

1 Sample Fabrication

We studied two samples in this work: D1, presented in the main text and D2, presented in the SI. Both samples consist of a gate-tunable van der Waals heterostructure and a bow tie-shaped terahertz cavity. For the heterostructure, we used the scotch-tape method to exfoliate Kish graphite and hexagonal boron nitride (hBN) from bulk crystals onto p-doped SiO_2-on-Si chips (285 nm/520 μm). We identified bilayer graphene, few-layer graphite, and hBN flakes in an optical microscope and determined their thicknesses using the optical contrast technique. The thickness of hBN flakes was further confirmed with atomic force microscopy (AFM, non-contact mode) giving 16 nm for the top and 26 nm for the back hBN flakes (D1), and 23 nm for the top and 20 nm for the back hBN flakes (D2). In D1, we chose graphite flakes for both top and back gates with a thickness of four graphene layers to minimize THz absorption and achieve the highest device quality. In D2, both gates are made from 4 nm evaporated Cr thin films. The continuity of the 4 nm Cr film used for the gates in device D2 was confirmed by scanning electron microscopy (SEM). The device structures, including layer thicknesses, are shown side-by-side in Fig. S1.

We assembled the individual flakes into a heterostructure using a polymer-based top-down dry-stacking technique in an inert Ar atmosphere at 120 °C to 140 °C. The stamp consisted of a polymethyl methacrylate (PMMA) hemisphere mounted on a glass slide and a bisphenol-A polycarbonate (PC) thin film attached to PMMA. After completion of the stack, we deposited it on a target substrate of high-resistivity SiO_2-on-Si (285 nm/520 μm) by melting the PC film at 180 °C with pre-patterned markers and rough electrodes (8 nm Ti/192 nm Au). For D2, we placed the heterostructure on a pre-patterned metallic back gate (4 nm Cr). We chose Si with high resistivity as the substrate to have the highest possible THz transmission and index matching with the immersion lenses[1]. The stack was placed on the target substrate aligned with the center of the immersion lenses. Subsequently, we dissolved the PC residues by immersing the chip in chloroform at 60 °C for 30 minutes.

We then etched the heterostructure into a rectangular shape using optical lithography with a direct laser writer and reactive ion etching (RIE) with a mixture of CHF_3 and O_2 gas [2] (only D1). Contacts to bilayer graphene and the graphite back gate were made by optical lithography and a combination of RIE and electron-beam evaporation of Cr (10 nm) and Au (70 nm) (D1) and 10 nm Cr/53 nm Au (D2). In this process, we used the same mask for etching

and metal evaporation. For D1, we contacted the graphite top gate using a separate step of optical lithography and evaporation of Cr (8 nm)/Au (52 nm) without prior etching. We also patterned the graphite top gate using RIE (O_2 gas) to avoid short circuits from the top gate to other leads. After this step, the etch contacts to bilayer graphene were re-made with the same recipe as above to ensure a good contact (with deposition of 10 nm Cr and 60 nm Au). For D2, both gates were contacted with optical lithography and evaporation of 10 nm Cr/53 nm Au. Fig. S1 presents micrographs of devices D1 and D2 after these fabrication steps.

Subsequently, we grew $\sim$ 140 nm of alumina (AlO_x) using atomic layer deposition (ALD) with trimethylaluminate (TMA) at 180 °C and water (H_2O) as precursor gases to electrically insulate the heterostructure and its leads from the resonator plane (D1 and D2). We verified the thickness with optical ellipsometry. We performed the ALD growth in four successive steps in between which we cleaned both the sample and growth chamber. One of the cleaning steps also involved a quick dip of D1 into an aqueous KI/I_2 solution to etch any gold structures pinching through the oxide. We also opened up the oxide layer to have access to the bond pads on the substrate. This was achieved with optical lithography with a mask aligner and Al-selective wet etching of the oxide layer at 50 °C. The etching solution consisted of a mixture of water, acetic acid, nitric acid, and phosphoric acid. To further improve dielectric insulation, we deposited another $\sim$ 150 nm of alumina with electron-beam evaporation in a next step (only D1). To still allow close proximity of the THz cavity and BLG, we patterned this second layer with photolithography in a lift-off process to only cover regions away from the heterostructure.

We defined the terahertz cavity with another round of optical lithography and metal evaporation (8 nm Ti/192 nm Au) on top of the heterostructure (devices D1 and D2). The terahertz cavity consists of a bow tie-shaped opening in a circular metal screen with a diameter of 4 mm (see Fig. S1 D/H). We patterned a pedestal structure for the solid immersion lens to reside on using the epoxy-based negative photoresist SU8 (thickness $\approx$ 2.2 µm) and optical lithography with a mask aligner. The pedestal structure did not cover the surroundings of the bow-tie opening and heterostructure (red circle in Fig. S1D/H). Finally, we mechanically mounted two Si solid immersion lenses (SILs) on both the front and back sides of the sample chips in a custom copper sample holder and wire-bonded the samples using gold wires.

Note that large-area 2D materials obtained by direct growth [3, 4] or exfoliation using metallic layers [5, 6] are better adapted to the sample-size requirements of terahertz spectroscopy than exfoliated flakes. However, such large flakes are not commonly assembled into

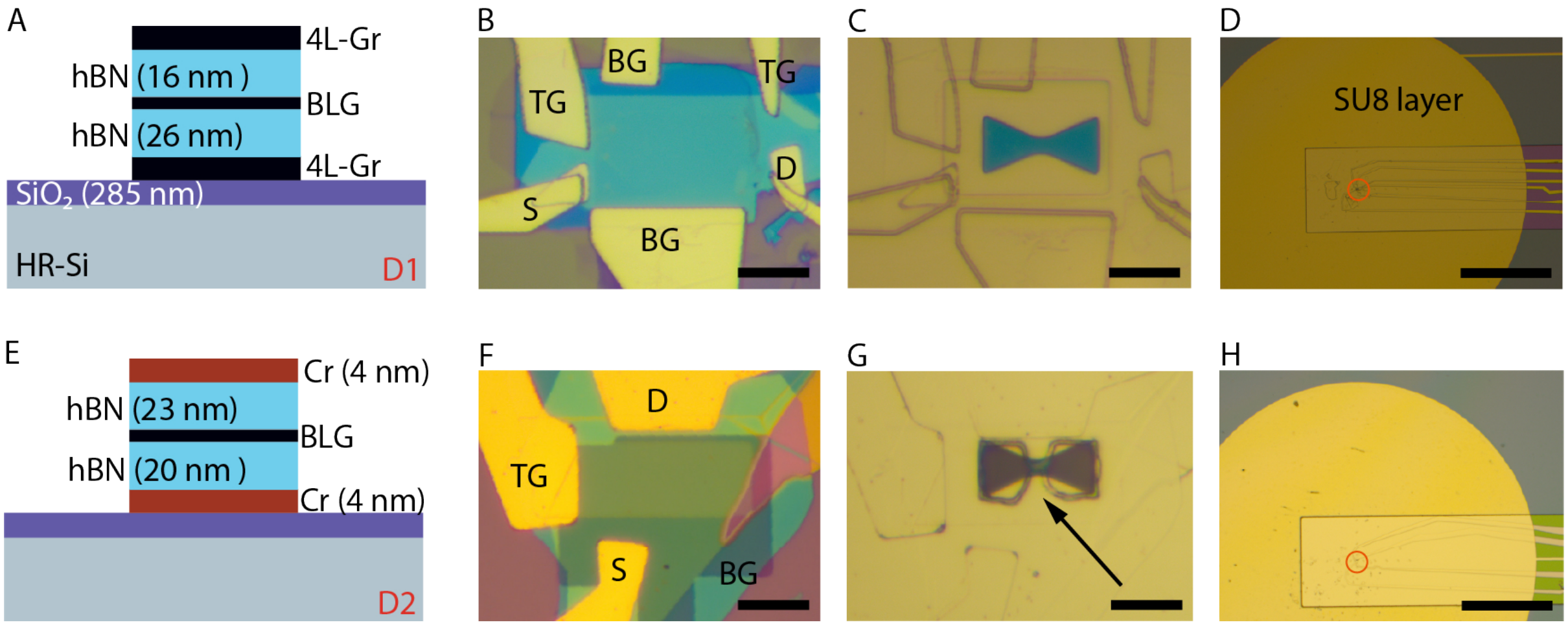

Figure S1: Devices structure and fabrication. **A/E** Schematics of the heterostructure of D1 and D2, respectively. **B-D/F-H** Micrographs of various fabrication steps in D1 and D2, respectively. The devices are shown after fabrication of all electrodes (**B,F**), after deposition of dielectric insulation layers and fabrication of bowtie resonator (**D,G**), as well as after fabrication of the SU8 spacer layer (**E,H**). The black arrow in **G** points to polymer residues deposited during the fabrication of the resonator in D2. The red circles in **E** and **H** mark the locations of the resonator in each device. In **B,C** and **F,G**, the scale bar is 10 µm. In **D/H** the scale bar is 1.0 mm.

tunable high-quality heterostructures achievable with exfoliation. Thus, they do not provide a fitting material platform for our experiments.

2 Cryogenic sample characterization

The sample was mounted mechanically with two Si SILs in a custom sample holder made from copper with electrical access. We placed this assembly in an optical continuous-flow cryostat with polymethylpentene windows (TPX, transparent to THz radiation) and cooled it down with liquid He to a base temperature of $\gtrsim 5\,\mathrm{K}$. With a heater and adjusting the He flow rate, we were able to tune the temperature between $\gtrsim 5\,\mathrm{K}$ and 200 K. We controlled the voltage on the electrodes for top and back gates using two separate source meters. The bilayer graphene flake was contacted from two opposing sides in order to measure 2-point electrical transport. We kept one of these contacts on ground for all measurements and connected the other one to a third source meter to apply a bias of $100\,\mu\mathrm{V}$. This resulted in a current on the order of a few nA easily detected using the same source meter. All electrical contacts to the samples were connected in series with cold $9.1\,\mathrm{k\Omega}$ resistors. To characterize the device, we first established the voltage limits for the two gates to avoid leakage currents and then varied the top and back gate individually to obtain the charge map displayed in Fig. 2A of the main text.

3 Terahertz Time-Domain Spectroscopy

We generated coherent broadband, pulsed THz radiation by optical rectification of ultrafast laser pulses derived from a fiber oscillator (Coherent Fidelity 2, wavelength 1070 nm, pulse width 45 fs, 70 MHz repetition rate, 2 W cw-equivalent output power) in the organic crystal N-benzyl-2-methyl-4-nitroaniline (BNA) [7]. The THz radiation was then focused onto the sample using gold-coated off-axis parabolic mirrors. The transmitted THz field was probed coherently using electro-optic (EO) sampling with a mechanically delayed portion of the fundamental laser pulse. The THz and the linearly polarized probe pulse were overlapped on a 200 µm thick [110]-cut GaP crystal, optically contacted to a 2 mm-thick [100] GaP crystal. Due to GaP's strong electro-optic response, the incident THz field induces ellipticity in the probe beam's polarization proportional to the field strength. The THz pulse train was modulated at 2.5 kHz using a chopper, and the resulting polarimetry signal was demodulated at this frequency with a lock-in amplifier. The time-domain profile of the transmitted THz pulse was recorded by varying the time delay between THz and probe pulses (see also Fig. 1 and 2 of the main text). The gate-dependent THz transmission data of Fig. 2B of the main text was acquired in a similar fashion, but fixing the stage position to the maximum value of transmission at the CNP and varying both top and back gates.

Since the transmission through the lens-resonator assembly is inherently low due to the sub-wavelength nature of the resonator opening, the signal was averaged over multiple acquisitions of the time trace at the same voltage configurations. In order to obtain the frequency-domain spectrum, the time-domain signal was Fourier transformed. Crucially, for every sweep in D or n we acquire an *in-situ* reference at the insulating configuration ($n = 0$, $|D| \gg 0$), and we re-acquire this reference at the start of each averaging block during long scans (Figure S10). Because the reference contains the day's coupling efficiency, beam pointing, and optics, taking the ratio removes alignment-dependent throughput and phase, isolating the BLG response. This interleaved referencing compensates slow drifts over hours to days and enabled week-long acquisitions (e.g., the 50 K dataset) without loss of comparability. Each gate-dependent THz-transmission map required a minimum of 3 days to a maximum of 8 days of continuous data acquisition. We obtained a usable bandwidth from 1 to 6 THz for D1 (see Fig. S2) and 1.5 to 5.5 THz for D2.

The temperature series and gate maps were taken on different days and under different

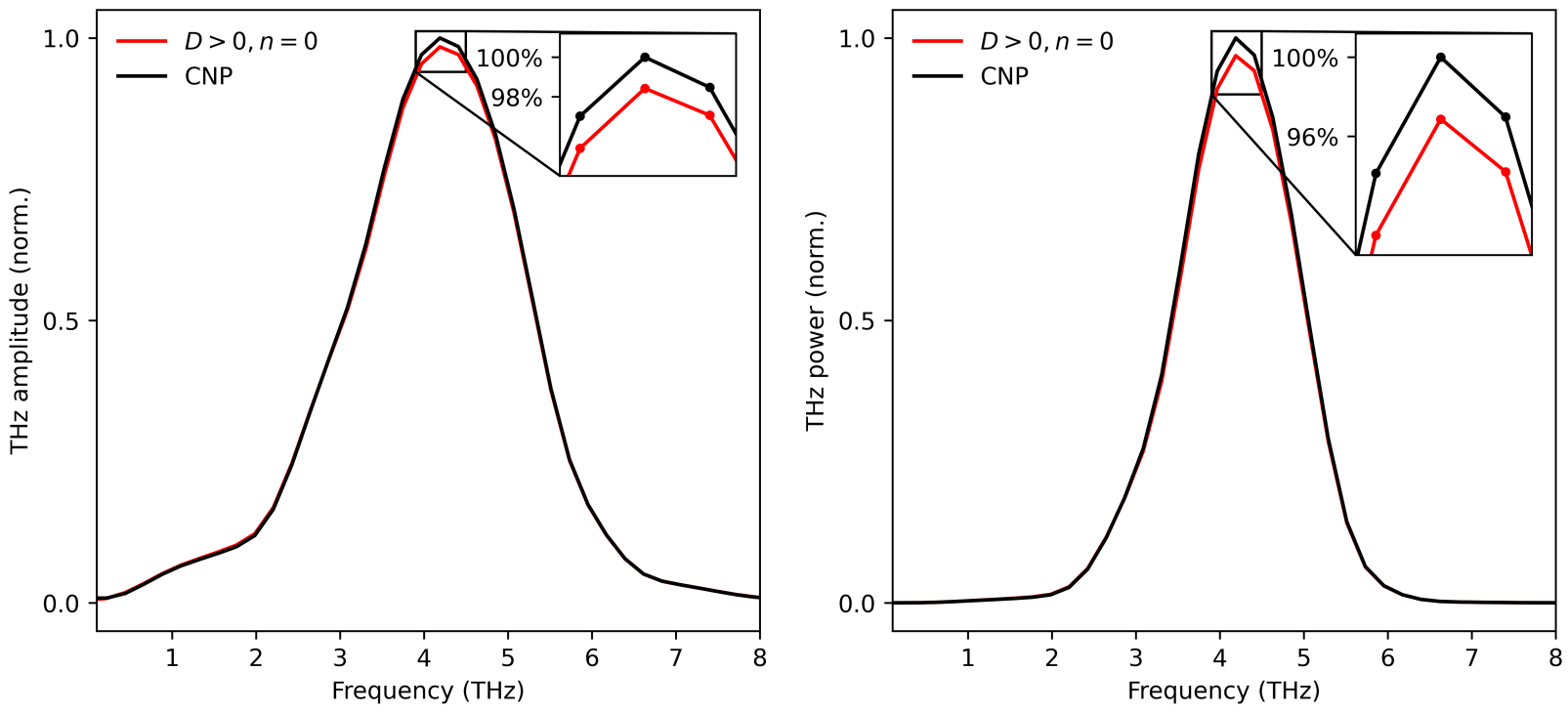

Figure S2: Amplitude (left) and power (right) spectra of transmitted THz pulses at different electronic configurations of bilayer graphene as shown in Fig. 2C of the main text (device D1). The temperature was 5 K.

alignments, yet all salient features are reproducible and recur in both time and frequency domains—see Fig. S3 for a representative collection of referenced transmission spectra taken at 5 K and adapted from Figs. 3D and 5A. These measurements, taken weeks apart under different alignments and cooldowns, are extremely reproducible for each given displacement field D. The relative difference between any two curves is $\leq 3\%$.

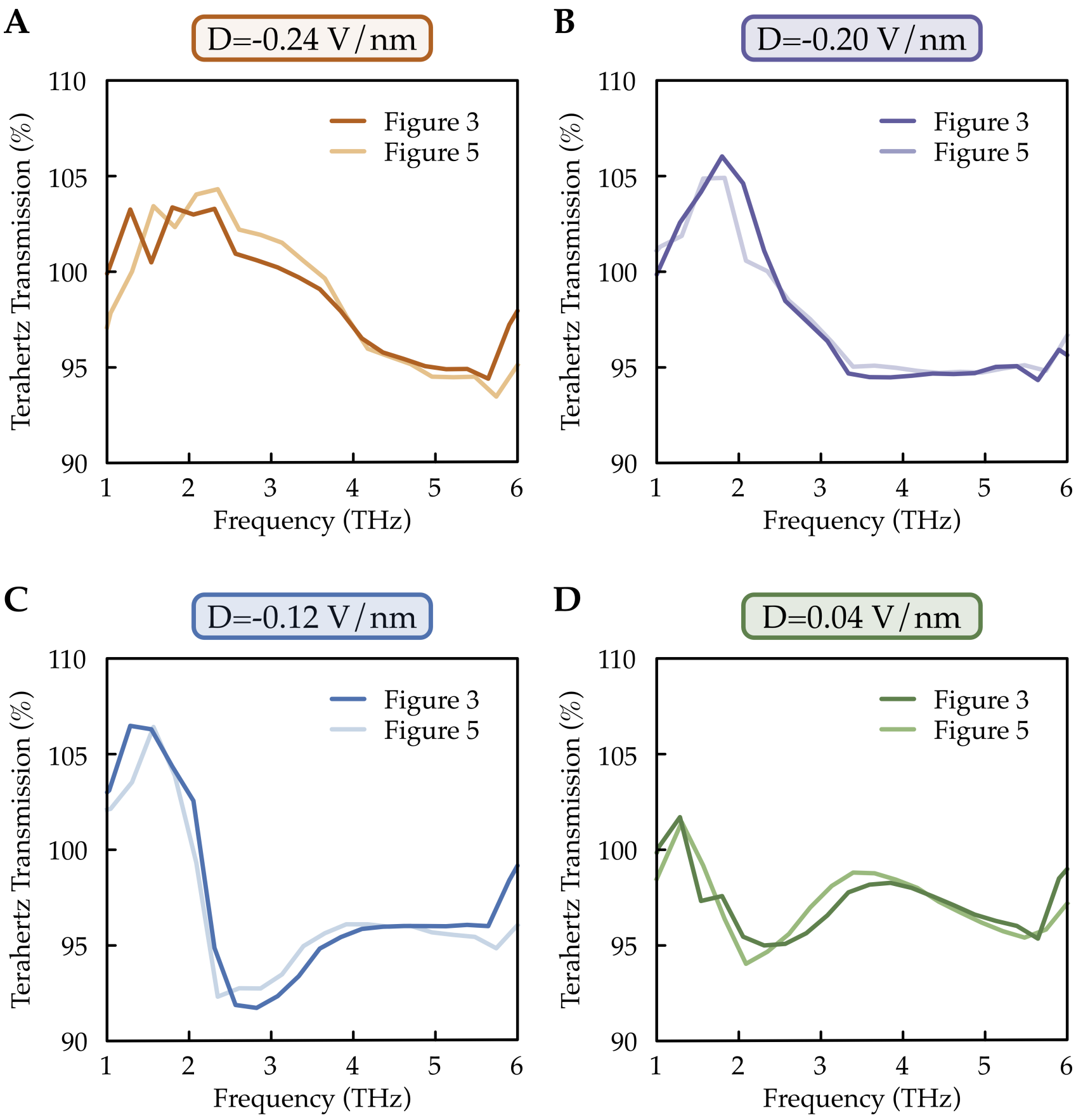

Figure S3: Referenced 5 K transmission for two separate measurements at different D and $n = 0$, adapted from Figs. 3D and 5A. **A** $D = -0.24\,\text{V/nm}$. **B** $D = -0.2\,\text{V/nm}$. **C** $D = -0.12\,\text{V/nm}$. **D** $D = 0.04\,\text{V/nm}$.

4 Additional data device D1

4.1 Evolution of the UP with D and T

In the colormap representation of Fig. 3 and Fig. 5, the much stronger LP and polaritonic gap dominate the dynamic range, which can make the UP appear visually suppressed when a single common color scale is used. To better highlight the UP, in Fig. S4 we replot the data from Fig. 5A of the main text over the restricted frequency range 2.3–5.4 THz, spanning from the cavity frequency to near the upper limit of our spectral window. This removes the LP contribution and excludes the noisier high-frequency tail, making the evolution of the UP more clearly visible. For each displacement field D, we rescale the vertical axis in order to emphasize the UP peak and its dispersive evolution. This presentation avoids the potentially misleading impression that can arise from a colormap with a large dynamic range, where the UP is present but visually compressed relative to the LP.

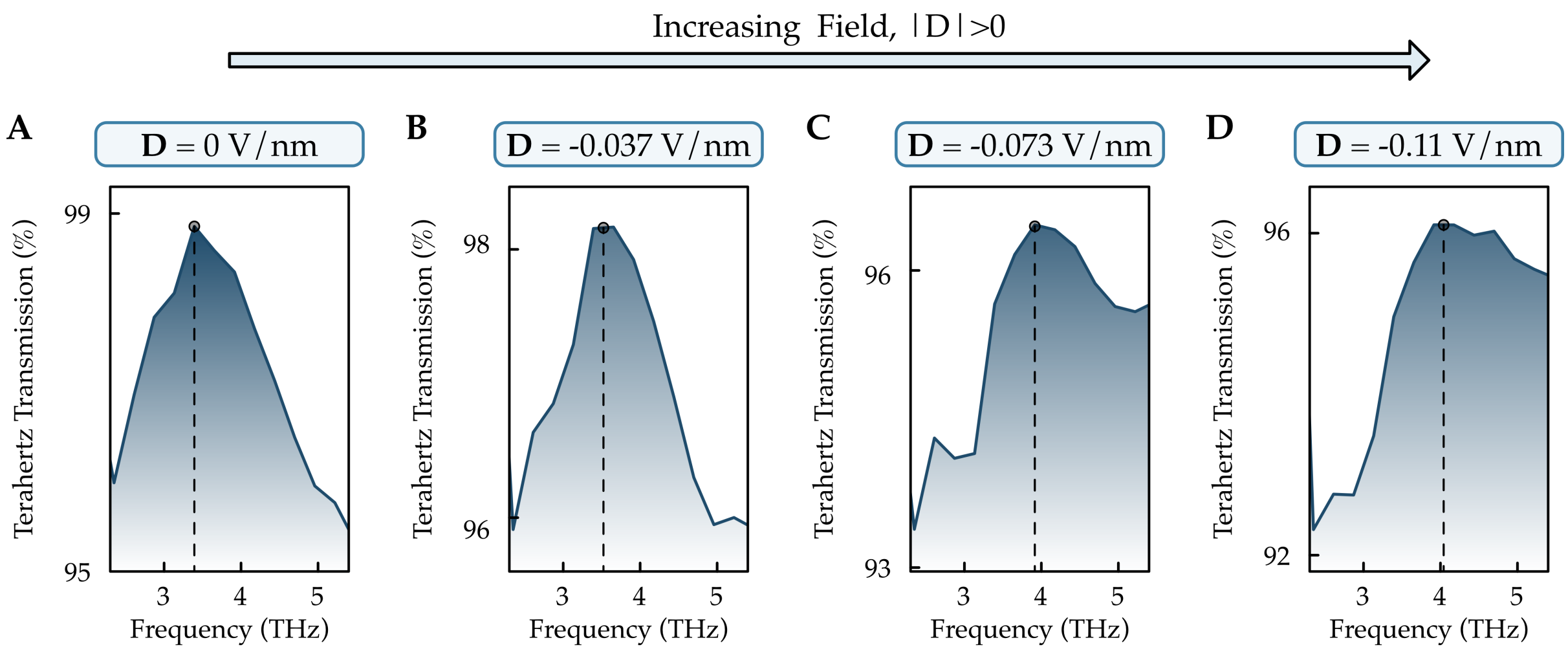

Figure S4: Upper polariton peak as a function of displacement field. The data is adapted from Fig. 5A of the main text. The frequency window is restricted to 2.3–5.4 THz (i.e., from just above the cavity frequency up to near the upper end of our spectral window), thereby removing the LP contribution and excluding the noisy high-frequency tails. The vertical axis is rescaled *independently for each D* (min-to-max within the displayed frequency window) to highlight the UP peak. The broken line highlights the peak position. **A** $D = 0\,\mathrm{V/nm}$. **B** $D = -0.037\,\mathrm{V/nm}$. **C** $D = -0.073\,\mathrm{V/nm}$. **D** $D = -0.11\,\mathrm{V/nm}$.

4.2 Temperature-dependent data

Fig.S5 presents the temperature dependence of the polariton splitting, normalized to its maximum value (extracted from the spectral maps in Fig. 5). The splitting is quantified by fitting both the lower and upper polariton peaks and calculating the difference between their positions. This analysis is performed for every displacement field value across all temperatures. Notably, the splitting remains constant within the experimental uncertainty.

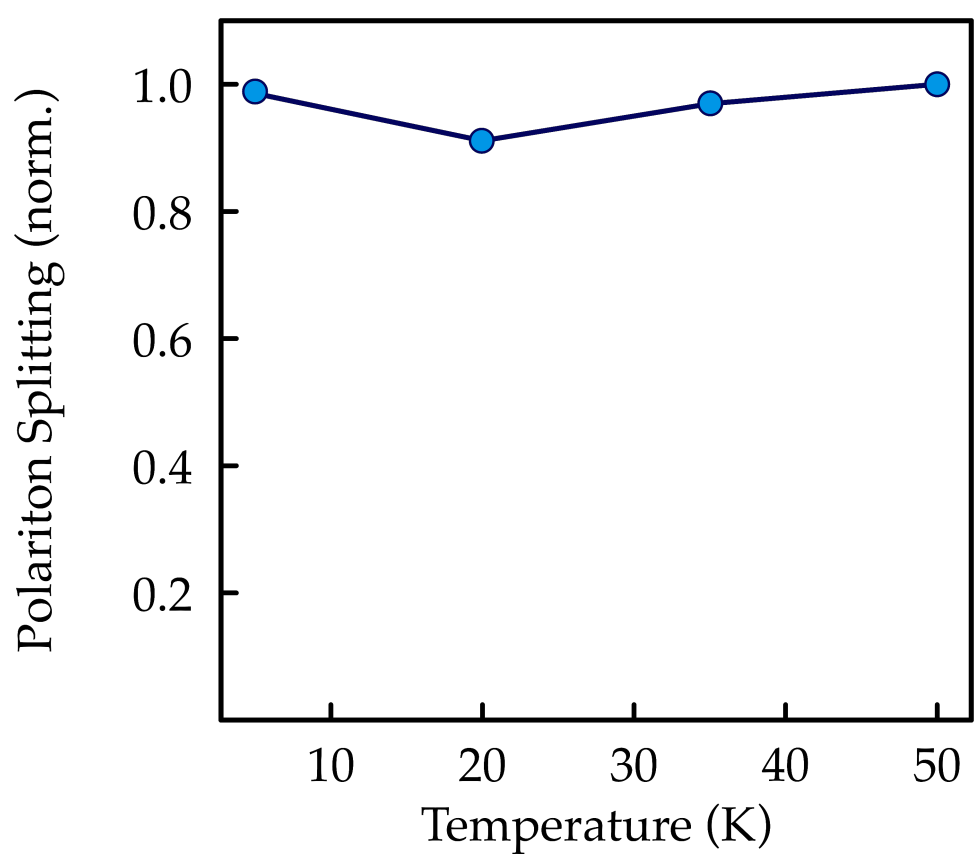

Figure S5: Temperature dependence of polariton splitting. Normalized. Obtained for $D = -0.16\,\mathrm{V/nm}$. Extracted from Fig. 5.

In addition to the temperature-dependent data presented in the main text Fig.5 ($5\,\mathrm{K} \leq T \leq 50\,\mathrm{K}$), we have performed measurements of the polariton dispersion at 100 K and 200 K on device D1 with identical voltage settings as for the lower temperatures. These data are depicted

in Fig. S6 as a contour plot (similar to Fig. 5). At 100 K, the polariton splitting and band edge are still visible albeit with reduced contrast, continuing the trend observed between 5 and 50 K. The poor signal-to-noise ratio prevents us from extracting the splitting quantitatively in this case. For 200 K, the spectral signatures of polaritons and the band edge are no longer discernible. In both measurements, the signal-to-noise ratio is poorer than in the measurements at lower temperatures due to the worse transmission of the Si immersion lenses at elevated temperatures, caused by the absorption of thermal carriers in Si.

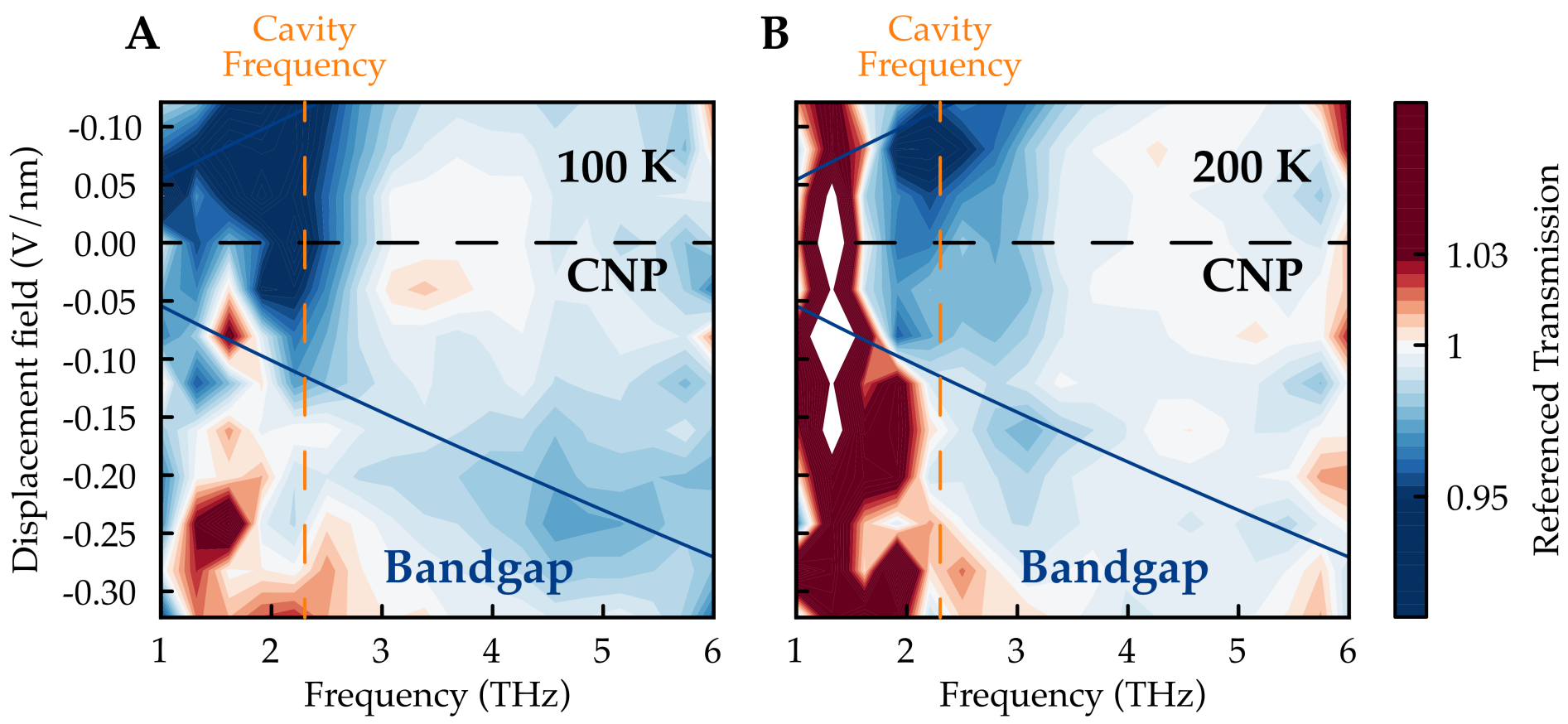

Figure S6: Displacement field-dependent THz transmission spectra at **A** 100 K and **B** 200 K measured on device D1. The voltage parameters are identical to Fig. 5 of the main text. The data is presented in a differently scaled color map as compared to Fig. 5.

4.3 Impact of doping on polariton formation

To investigate polariton formation in the presence of carriers, we present measurements at finite D (where both LP and UP are visible) and light doping of BLG in Fig. S7. We have performed the experiment for n-type doping and find that even a modest density $\left(n \sim 2 \times 10^{11}\,\mathrm{cm}^{-2}\right)$ dramatically suppresses the polariton contrast by the added losses of the resident carriers.

4.4 Extended doping-dependent data

In the main text we present doping-dependent spectra (Fig. 2E) as line cuts in a reduced spectral window from 1 to 4 THz to highlight the main absorptive feature at 2.3 THz. The full spectral window is available in Fig. 2D as a contour plot. Here, we provide the corresponding 1–6 THz line cuts in Fig. S8 together with a reprint of Fig. 2E.

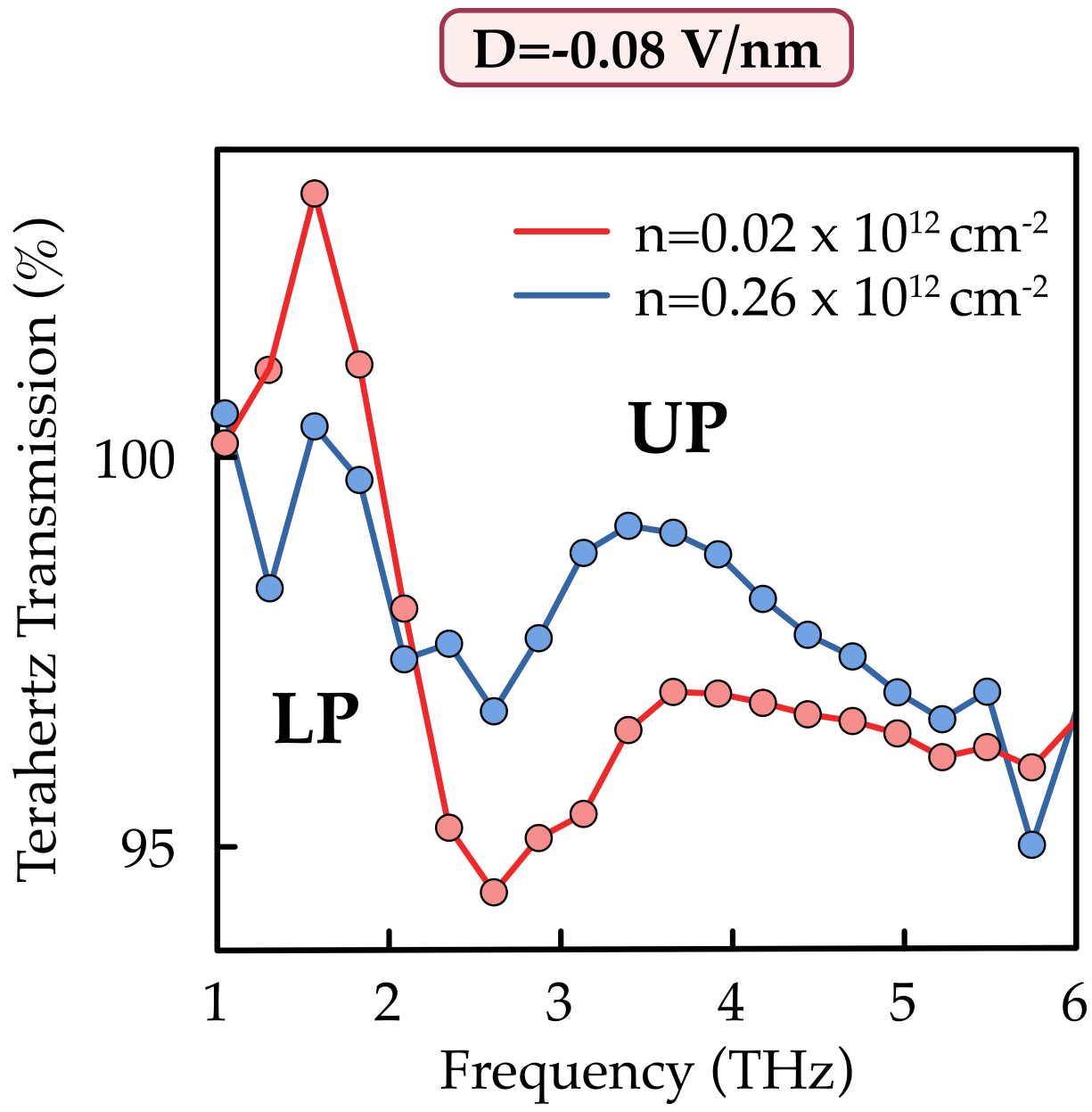

Figure S7: Influence of doping on spectrum at finite displacement field. Experimental transmission spectra obtained with n-type doping at $D = -0.08\,\text{V/nm}$. The doping is non-zero for the blue curve and vanishes for the red curves. The spectra are referenced to the insulating state at $D = -0.36\,\text{V/nm}$ and measured at 5 K on D1.

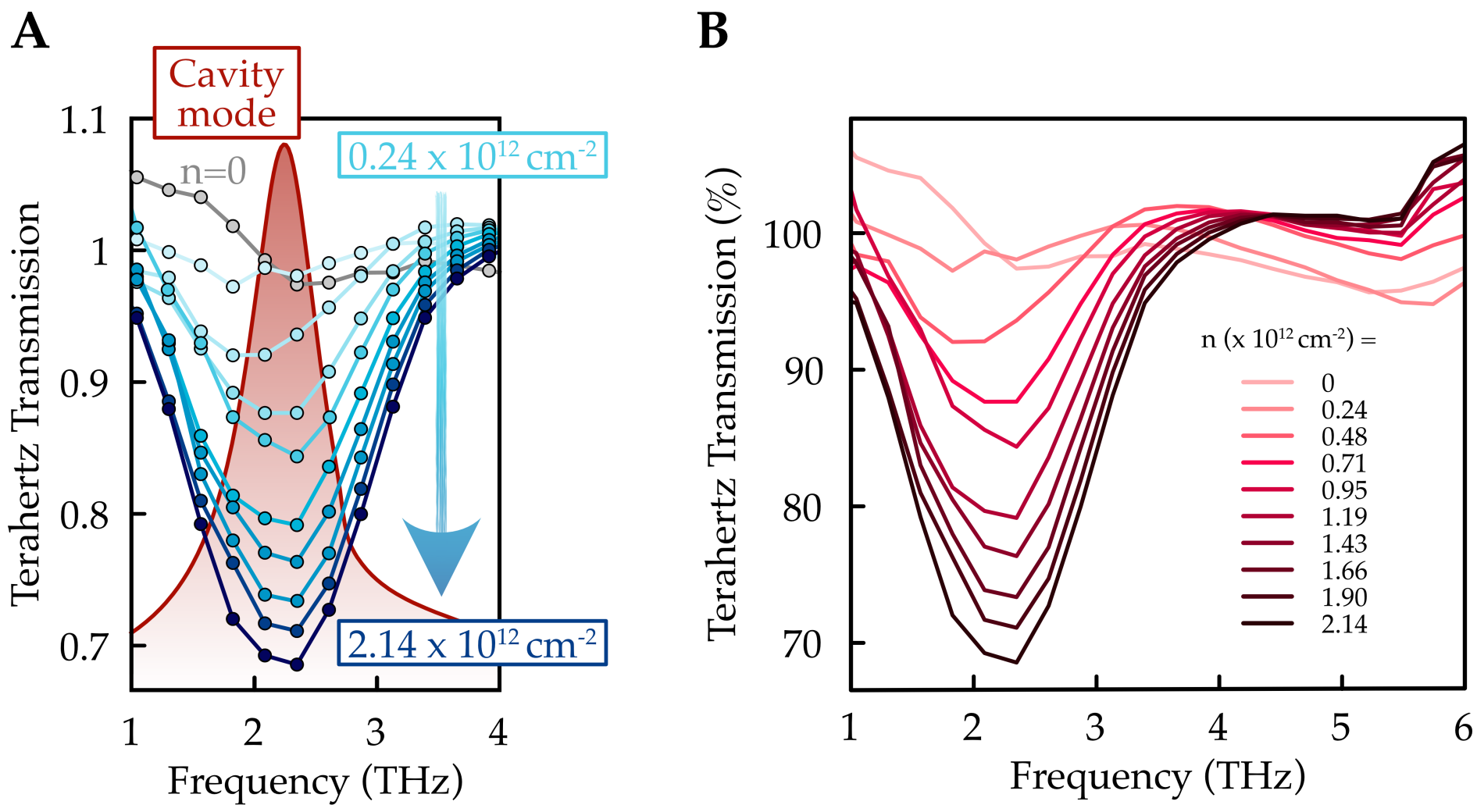

Figure S8: Doping-dependent transmission spectra. **A** Referenced transmission spectra measured at $D = 0$ for increasing electron density (blue) overlaid with the simulated cavity transmission spectrum (red). This panel is a reproduction of Fig. 2E of the main text. **B** Same experimental data as in panel **A** but displayed on an extended spectral axis from 1 to 6 THz. The spectra are referenced to the insulating state at $D = -0.38\,\text{V/nm}$ and were measured at 5 K on device D1.

5 THz spectroscopy data of device D2

In Fig. S9, we present THz spectroscopy measurements on device D2 at zero doping ($n = 0$) as a function of displacement field D, analogous to the data shown for device D1 in Fig. 3D of the main text. Although device D2 has a substantially lower signal-to-noise ratio than D1, owing to its reduced transmission and larger disorder, the key spectroscopic features remain

the same.

As in D1, we observe interband absorption that follows the theoretical bandgap expected from the model of Ref. [8] (black line), together with the emergence of a polaritonic splitting as the bandgap approaches the cavity frequency of approximately 2.3 THz. This feature becomes most pronounced near the crossing between the bandgap line and the cavity mode, where the spectra develop a dip between two shoulders, consistent with an anticrossing rather than a simple band-edge response. Despite the reduced contrast, we also resolve a feature consistent with the upper polariton (Fig. S9A,B), which is central to our interpretation and is not naturally accounted for within a conventional Fano picture. By contrast, the narrower bandwidth and increased noise make the lower-polariton position more difficult to extract reliably in D2.

The overall agreement between D1 and D2 shows that the cavity-induced exciton binding is robust against electronic disorder. In D2, both gates are made of thin metallic films, in contrast to the graphite gates used in D1, and the rough metal–hBN interface of the back gate likely introduces additional disorder. At the same time, these ultrathin metallic gates reduce the absolute transmission by about a factor of two, limiting the usable spectral window to 1.5 THz to 5.5 THz.

Because gate leakage in D2 prevents measurements at large negative displacement fields, we instead access the same physics at positive D. This is justified by the symmetry of the bilayer-graphene band structure under reversal of the sign of D. Since the ranges of $|D|$ explored in D1 and D2 are comparable, the two devices probe the same regime. For easier comparison with D1, we therefore flipped the vertical axis in Fig. S9.

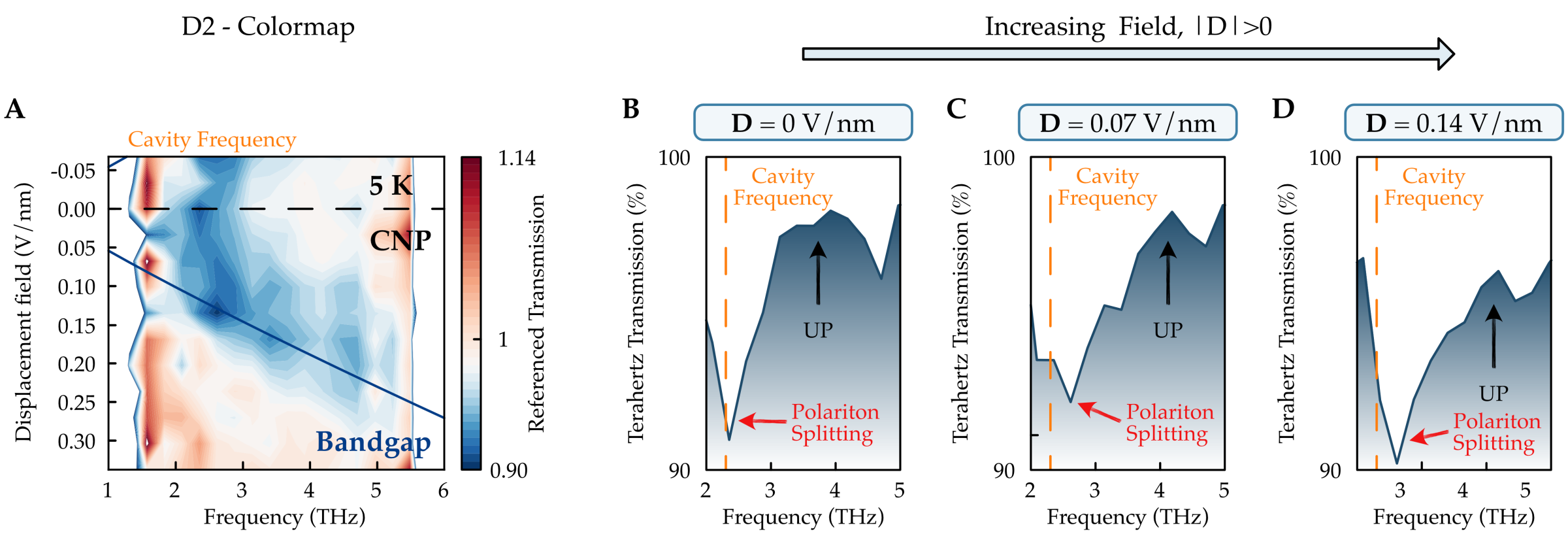

Figure S9: THz spectroscopy of device D2 for varying displacement field D at zero doping $n = 0$. **A** The spectra are normalized to the insulating state at $D = 0.39\,\mathrm{V/nm}$ and all obtained at 5 K. The horizontal black line marks the CNP and the oblique black line represents the calculated band gap within the model of Ref. [8]. Note that the vertical axis is flipped for easier comparison with Figs. 3D and 5 of the main text. **B** Line cuts for three representative displacement fields. The frequency window is restricted to highlight the UP and polariton splitting while removing the LP contribution and the noisy high-frequency tails.

6 Data analysis

6.1 THz time-domain data

To obtain the referenced THz spectra shown in the main text, we first Fourier transformed the experimental time-domain traces for each voltage configuration and then divided the resulting power spectra by the power spectrum of a reference trace recorded in the insulating state: at $D = -0.38\,\mathrm{V/nm}$ and $n = 0$ for the data in Fig. 2D-E and Fig. 5 of the main text (device D1), at $D = -0.36\,\mathrm{V/nm}$ and $n = 0$ for the data in Fig. 3 of the main text (device D1), and at $D = 0.39\,\mathrm{V/nm}$ for device D2. Figure S10 schematically illustrates this referencing procedure.

A sketch of the resulting lineshape for an idealized polaritonic doublet is shown in Fig. S11, where the cavity mode and the polariton branches are approximated as Lorentzian resonances. In a complementary metamaterial resonator [9], both the bare cavity mode (blue curve in Fig. S11A) and the polaritons (red curve) appear as peaks in transmission. In our referencing scheme, the spectrum containing the polaritons is divided by the bare-cavity spectrum. The resulting referenced spectrum (Fig. S11B) exhibits two peaks slightly shifted relative to the polariton frequencies (marked by black arrows), together with a dip near the cavity frequency, consistent with our experimental observations.

The individual time-domain THz traces were windowed using an asymmetric Gaussian window before Fourier transformation. Since the time-domain trace was measured multiple times for each voltage configuration, we averaged the resulting referenced power spectra. We

applied this procedure for all THz spectra displayed in the main text. For the extraction of the polariton frequencies and the coupling strength g from experimental data, we employ a fitting method based on transfer-matrix simulations of coupled cavity-oscillator models described in SI 7.2.

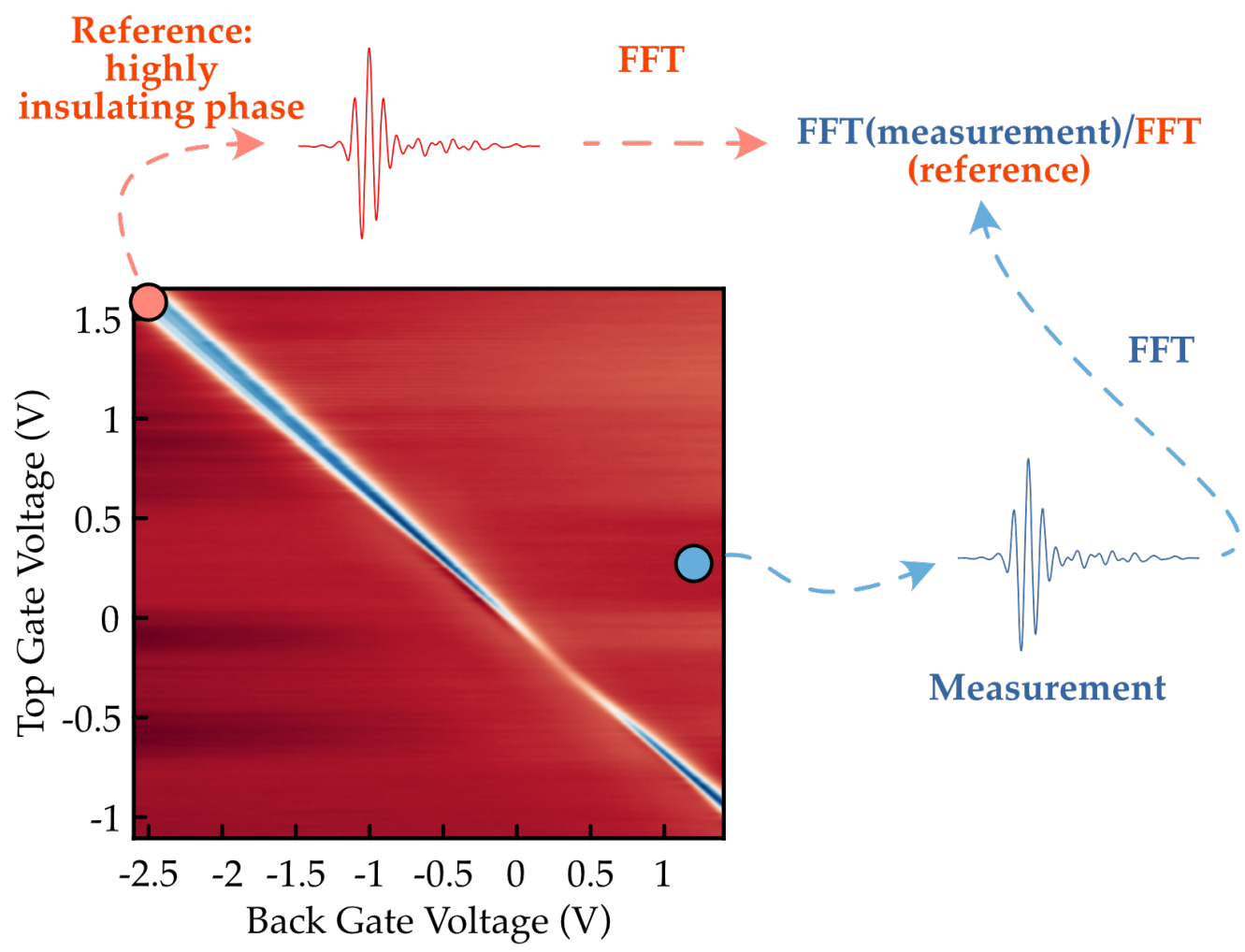

Figure S10: Normalization procedure: we acquire a time-domain trace in a point of interest (blue) and divide its spectrum by the spectrum of the trace taken in a highly insulating phase of BLG (red).

6.2 Band-edge absorption

The spectra obtained in this way are referenced spectra, in which absorption appears as a reduction of the transmission below unity, as illustrated schematically in Fig. S12A for the case of an interband absorption edge. In Fig. S12B we show the first four traces measured at $n = 0$ and large negative D, where the gap approaches ω_c but a clear anticrossing has not yet fully developed; the trace at -0.20 V/nm already shows the onset of the lower polariton. In this regime, the interband edge is still clearly identifiable (arrows) and appears as a *reduction in transmission* on the high-frequency side, as expected.

6.3 Doping-dependence of cavity linewidth

We fit the transmission dips in the density dependent measurement Fig. 2D-E with a Gaussian lineshape to estimate the full width at half-maximum (FWHM). The resulting FWHM as a function of density n together with the FWHM extracted from FEM simulations in Fig. 2F as a function of the plasma frequency ω_p are plotted in Fig. S13. With increasing doping, we observe an increase in the linewidth in both the measurements and simulations due to the

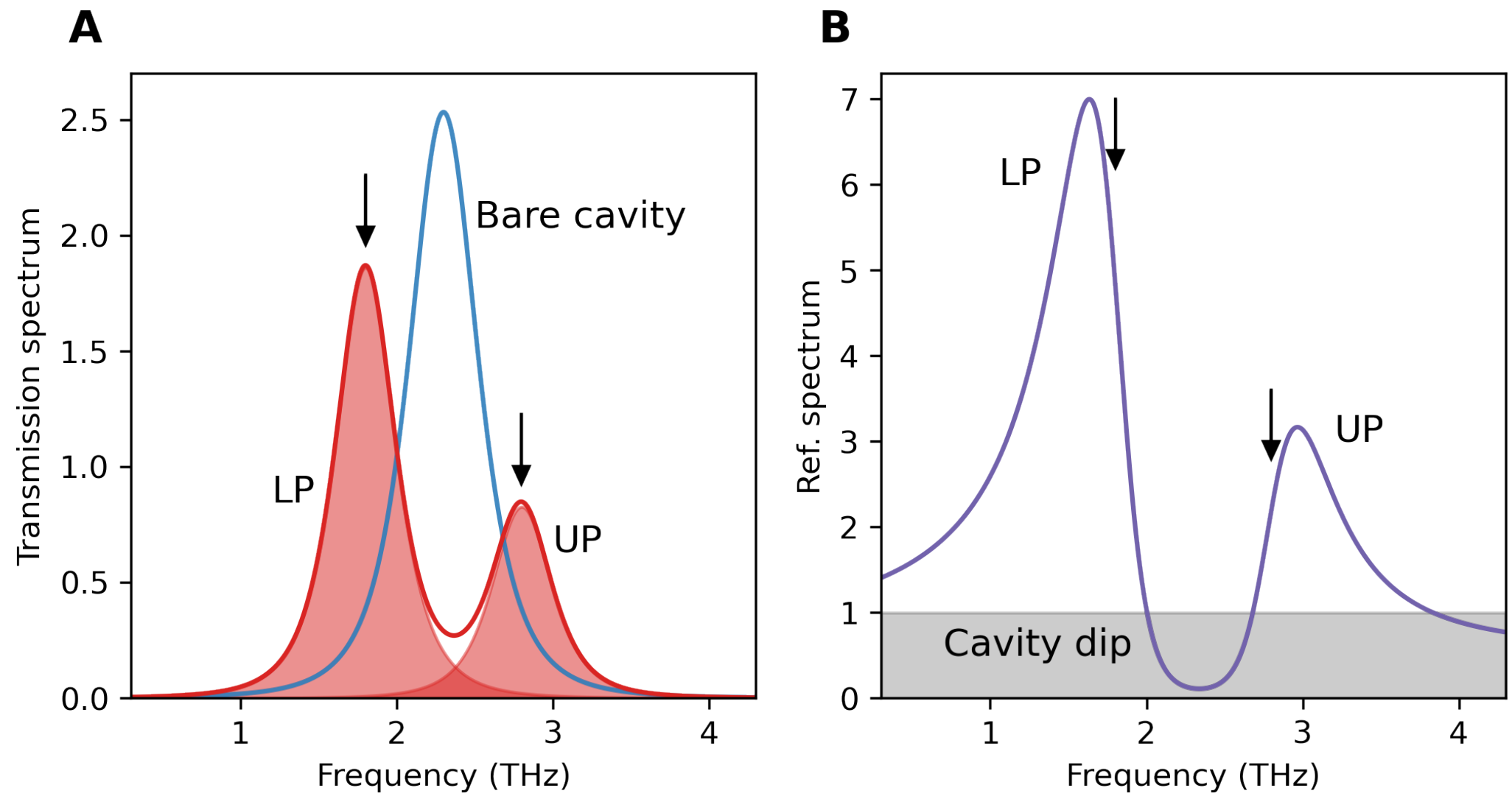

Figure S11: Schematic of the spectral referencing scheme. **A** Lorentzian transmission spectra for a strongly coupled system consisting of LP and UP (red line) as well as for the bare cavity (blue line). The red-shaded areas designate the individual transmission spectra of LP and UP. The arrows mark the LP and UP line positions (i.e. the peak positions). **B** Referenced transmission spectrum obtained by dividing the polariton spectrum (red line in panel A) by the bare cavity spectrum (blue line in panel A). The arrows mark the line positions extracted from panel A. The gray shading highlights transmission below unity, i.e. locations where the bare transmission is larger than the one of the polaritonic system.

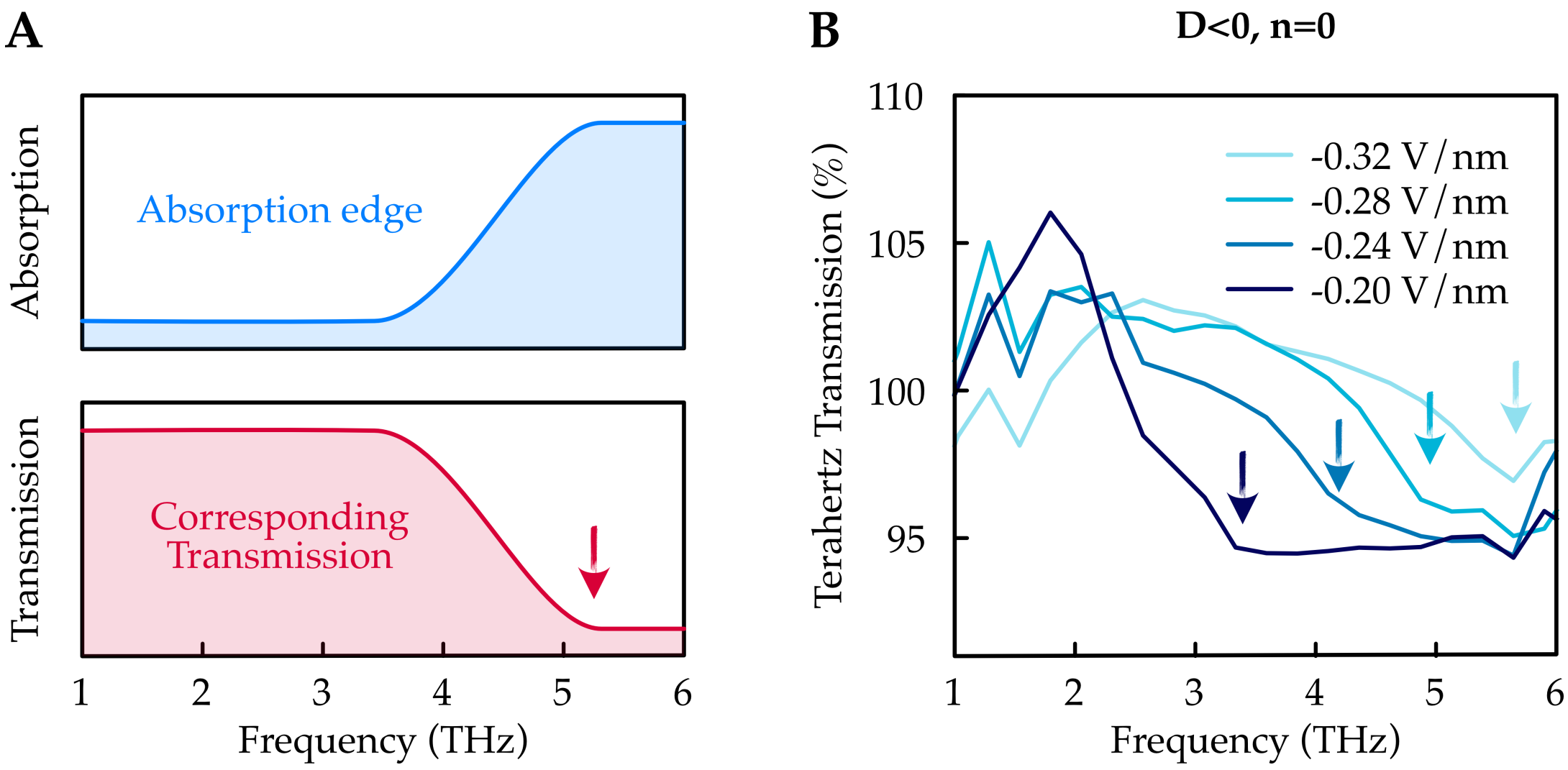

Figure S12: Band edge absorption. **A** Schematic of THz absorption by a band edge (top) and the corresponding transmission spectrum (bottom) with an arrow marking the location of the band edge. **B** Experimental transmission spectra at various displacement fields $|D| > 0$ and $n = 0$ referenced to the insulating state $D = -0.36$. The arrows mark the onset of absorption, similarly to panel A. Data obtained from device D1 at 5 K.

enhanced Drude absorption by the added carriers.

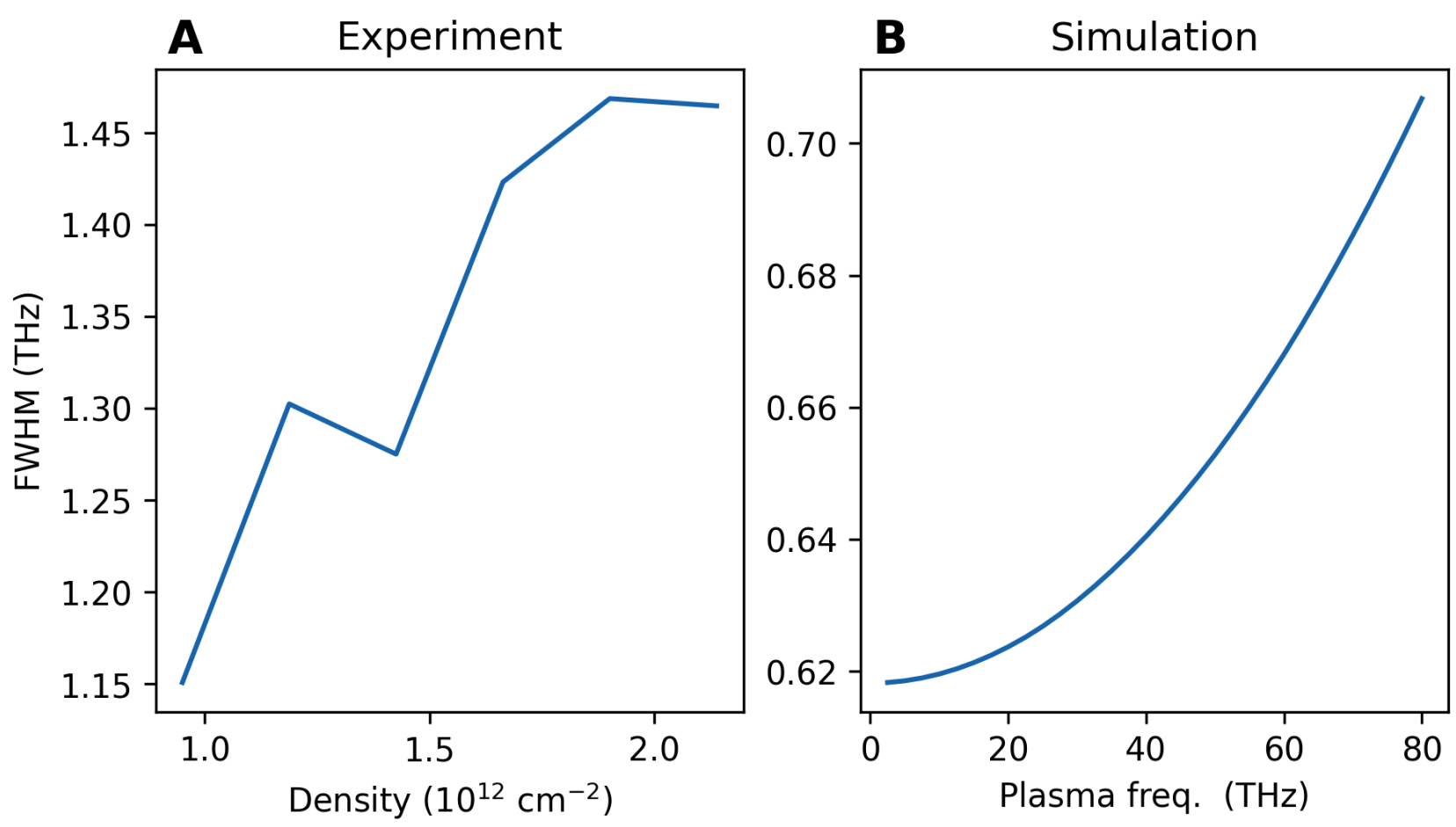

Figure S13: Linewidth as function of doping. A FWHM of the spectral dip in the density-dependent measurement of Fig. 2D-E. **B** FWHM of the simulated spectra in Fig. 2F as a function of the plasma frequency ω_p.

6.4 Transport

6.4.1 Device D1

From the transport measurements, we extract the BG voltage for each TG voltage where the current is minimal. These voltage points determine the charge neutrality line (CNL) where the doping is fixed to be zero and we change only the displacement field. We then fit a straight line to these points (see Fig. S14) to determine the lever arm $\alpha \approx 0.65$. This value agrees well with the lever arm determined from the experimental hBN thicknesses obtained by AFM measurements

$$\alpha' = \frac{d_{\text{top}}}{d_{\text{back}}} \approx 0.62. \tag{1}$$

Since the current value along the CNL decreases as the magnitude of the displacement field (and thus the bandgap) is increased, we can determine the charge neutrality point (where the bandgap is minimal) by finding the voltage configuration (at $V_{\text{TG}} = -0.225\,\text{V}$ and $V_{\text{BG}} = 0.3\,\text{V}$) on the CNL with the largest current. We then determine the displacement field D and charge density n from the plate capacitor model (as stated in the main text) using the dielectric constant of hBN of $\varepsilon = 3.4$ [10].

6.4.2 Device D2

For Device D2, we perform analogous transport characterization measurements as for device D1. We measure the current for a fixed source-drain bias as a function of the top and back gate

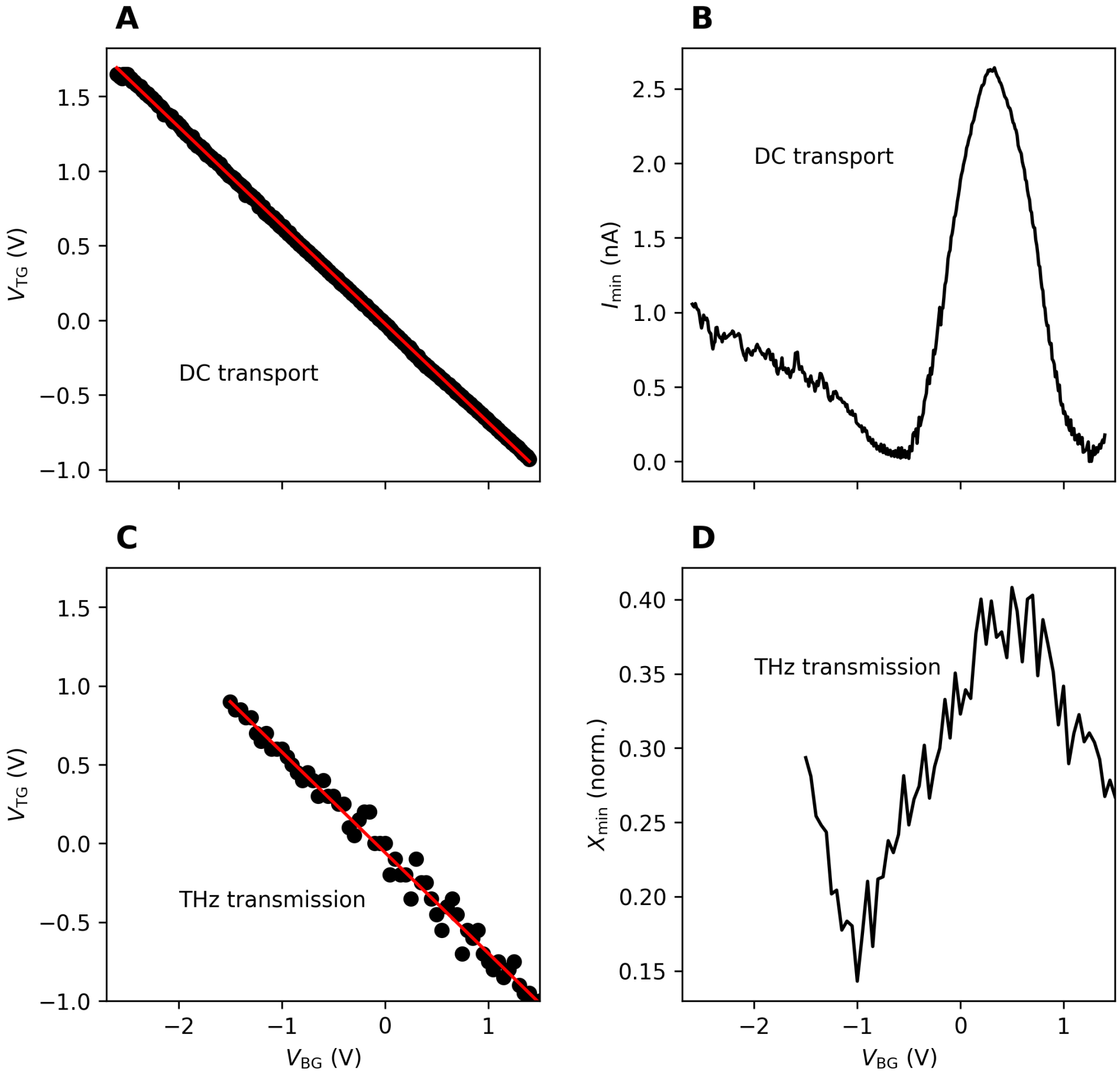

Figure S14: Analysis of transport data in device D1. **A** Current minimum positions as function of top and back gate voltages as obtained from 2-point transport measurements (see Fig. 2A of the main text) (black dots) and linear fit (red line) determining the lever arm $\alpha \approx 0.65$. Measurements along this line keep the charge density constant while changing the displacement field only. **B** Current with the back and top gate voltages scanned along the charge neutrality line. The maximum value determines the gate voltages at the charge neutrality point (CNP). **C** Positions of THz transmission minima (black dots) at fixed pump-probe delay as function of top and back gate voltages (see Fig. 2B of the main text). The red line is a linear fit giving a lever arm $\alpha'' \approx 0.63$. **D** THz transmission at fixed pump probe delay with the back and top gate voltages scanned along the charge neutrality line.

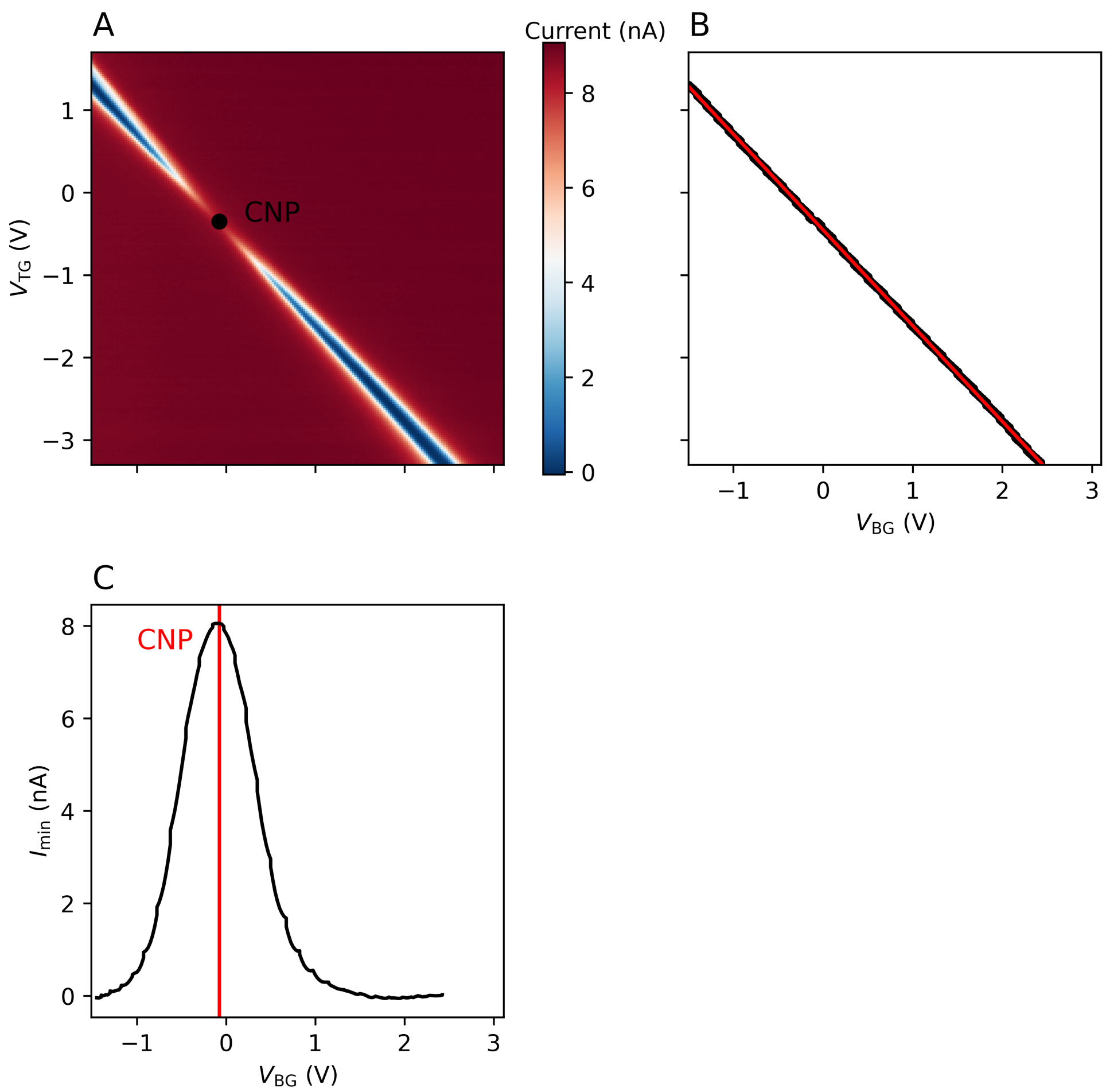

Figure S15: Analysis of transport data in device D2. **A** Current map as function of top and back gate voltages. **B** Current minimum positions as function of top and back gate voltages as obtained from 2-point transport measurements (see Fig. 2A of the main text) (black dots) and linear fit (red line) determining the lever arm $\alpha \approx 1.16$. Measurements along this line keep the charge density constant while changing the displacement field only. **C** Current with the back and top gate voltages scanned along the charge neutrality line. The maximum value determines the gate voltages at the charge neutrality point (CNP, red vertical line).

voltages. The results are displayed in Fig. S15. Similar to D1, we observe the emergence of a resistive CNL. Fitting the positions of the current minima with a linear model (Fig. S15B) as discussed above, yields a lever arm (see Eq. (1))

$$\alpha_{D2} \approx 1.16 \tag{2}$$

in decent agreement with hBN thicknesses of 23 nm and 20 nm for top and back gates, respectively. We determined the CNP as the location of the maximum current along the CNL. Within our experimental resolution, it is located at $V_{TG} = -0.35\,\text{V}$ and $V_{BG} = 0.075\,\text{V}$.

6.5 Single time-delay data

For fixed-delay THz transmission measurements (Fig. 2B, device D1), we sampled the electro-optic signal at the maximum of the transmitted waveform for each pair of top- and bottom-gate voltages, thereby constructing the 2D map. We then repeated the full scan multiple times and averaged the resulting maps to improve the signal-to-noise ratio. We normalized the signal of each scan to the maximum value to account for slow drifts and smoothed the data with a Gaussian filter. The normalization procedure is as follows

$$T_{\mathrm{norm}} = \frac{T - T_{\mathrm{min}}}{T_{\mathrm{max}} - T_{\mathrm{min}}},$$

where $T(V_{\mathrm{TG}}, V_{\mathrm{BG}})$ denote the peak transmission as a function of the top- and back-gate voltages, with T_{max} and T_{min} being its maximum and minimum values in the considered range of voltages. In Fig. S16, we present the normalized alongside the raw unnormalized data. We also determined the gate lever arm from the minimum values of transmission along the CNL and obtained $\alpha'' \approx 0.63$ (Fig. S14C). An accurate determination of charge neutrality is not possible from these measurements due to the markedly higher noise as compared to transport (see Fig. S14D) as well as the lower voltage resolution.

The choice of time delay t_0 in this experiment is arbitrary. We choose to fix the time delay to the maximum of the time-domain waveform to obtain the largest signal possible (see Fig. 2). In the right panel of Fig.S17, we show additional data from D1 sampled at various time delays (marked in the time trace in the left panel of Fig.S17) with $n = 0$ and varying D, together with a cut along the same direction taken from the map in Fig. 2B. These traces all reveal similar trends, showing that the choice of time delay does not affect the observed features in the map.

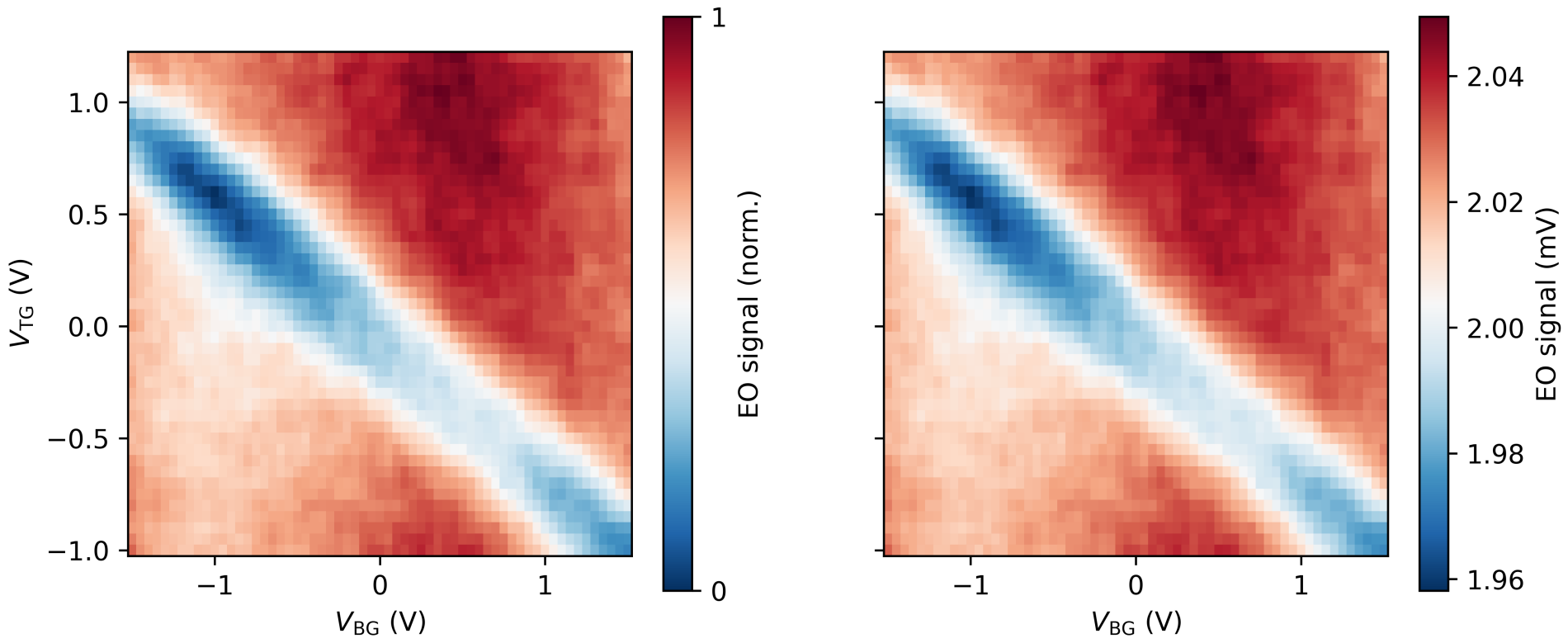

Figure S16: Normalized and unnormalized single time-domain data as presented in Fig. 2B of the main text.

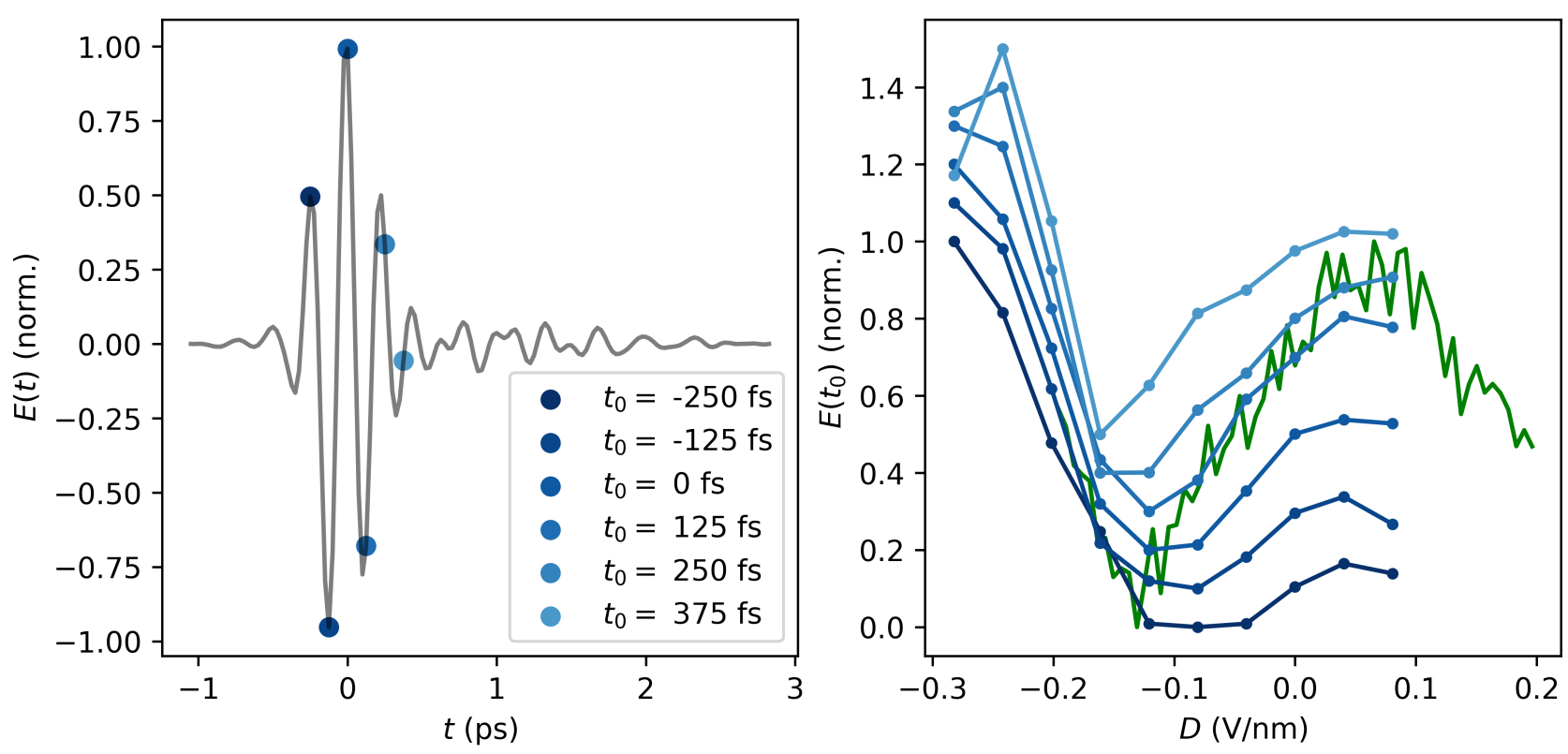

Figure S17: Displacement field-dependent THz transmission at various time delays.**Left** Transmitted THz pulse at the CNP with various time-delays marked. **Right** Displacement field-dependence for each time delay marked on the left panel (blue curves) along a cut at constant $n = 0$ through the map in Fig. 2B (green curve). The curves are off-set by 0.1 for clarity. The data measured at 5 K on device D1.

7 Fabry-Pérot cavity

7.1 Model

We introduce a minimal Fabry–Pérot (FP) model to motivate the generality of our observations. The setup includes a *thin* excitonic film ($t \ll \lambda$) placed at the center of the cavity (at the field antinode, Fig. S18E). The film is described by a single Lorentz pole,

$$\varepsilon(\omega) = \varepsilon_\infty + \frac{\omega_p^2}{\omega_0^2 - \omega^2 - i\Gamma\,\omega}, \tag{3}$$

with high-frequency dielectric constant ε_∞, exciton frequency ω_0, damping Γ, and oscillator strength encoded in $\omega_p = 2\pi f_p$.

To introduce the phenomenology of the model, we first consider—*unlike the experiment*—the idealized limit of *sharp* (high-Q) cavity and matter resonances. The central frequency of the Lorentz oscillator is externally controllable, just as in the experiment. This setup captures the universal behavior of ultrastrong coupling between two modes. The *unnormalized* terahertz transmissions of the bare cavity (Figure S18A) and of the isolated material layer are (Fig. S18C) shown as a function of exciton frequency in (Fig. S18B) and (Fig. S18D), respectively. When the material is placed in the cavity ((Fig. S18E)), the two resonances hybridize and produce the avoided crossing evident in the *unnormalized* transmission plotted in Fig. S18F. To mimic the experimental procedure exactly, we then calculate the referenced spectrum by dividing the coupled-system transmission of Figure S18F by the bare-cavity transmission, thereby *highlighting only the changes induced by mode interaction*.

With the working model in hand, we can now explore how a strongly coupled light–matter

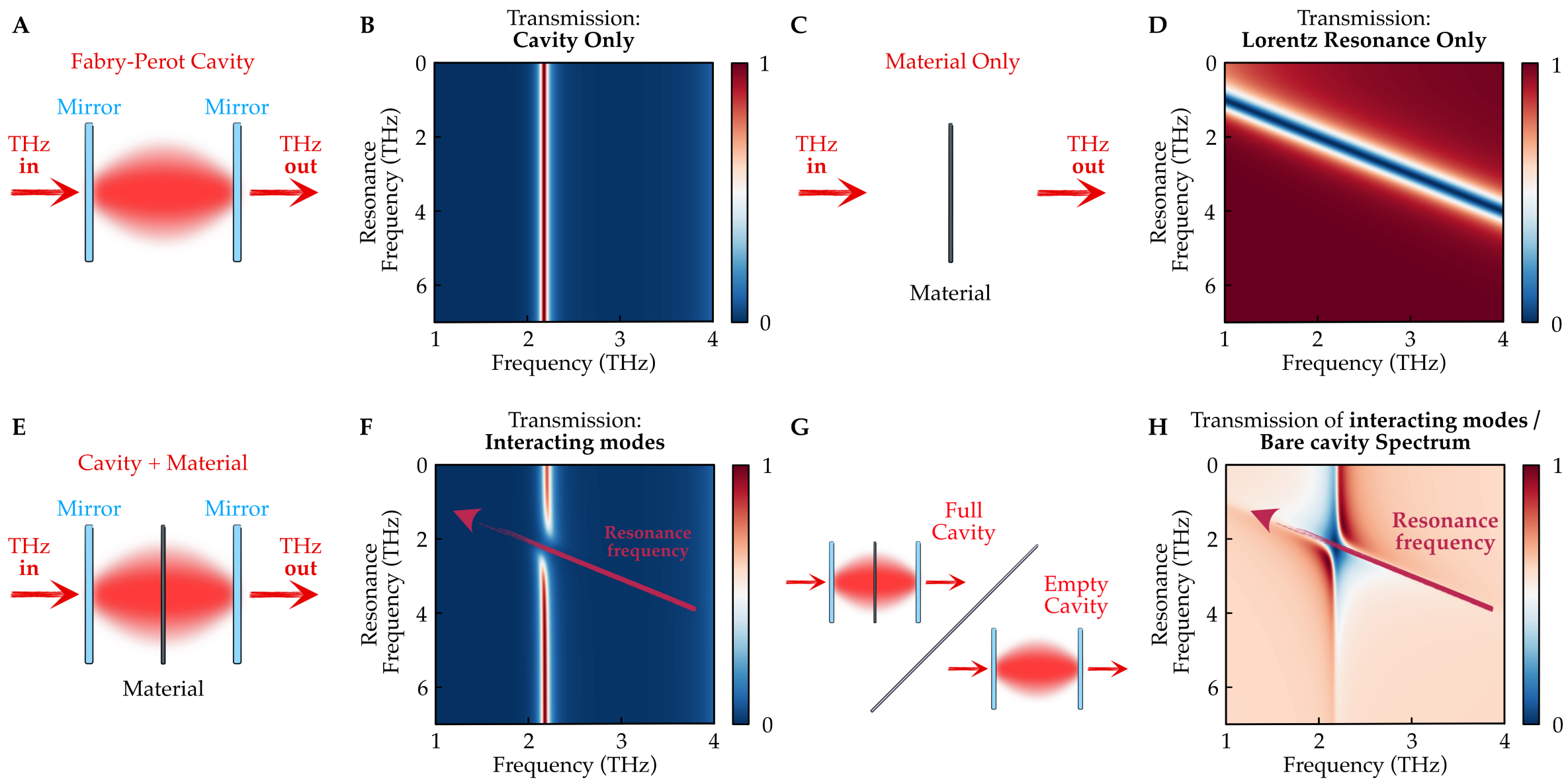

Figure S18: Strong coupling of an excitonic film to the fundamental mode of a FP cavity. **A** Schematic of a Fabry-Pérot (FP) cavity formed between two parallel metallic mirrors. The fundamental mode is sketched in red. Red arrows mark the incident and transmitted THz fields. **B** Normalized transmission spectrum of the empty FP cavity when the frequency ω_0 of the exciton is tuned. **C** Schematic of the thin film with incident and transmitted THz fields marked by red arrows. **D** Normalized transmission spectrum of a the excitonic film as defined in Eq. (3) as the resonance frequency ω_0 is varied. **E** Schematic of the coupled system. An excitonic thin film is placed in the center of a FP cavity and couples to its fundamental mode. The system is probed in transmission (red arrows). **F** Normalized transmission spectra of the coupled exciton and FP cavity system as function of resonance frequency ω_0. An anti-crossing is observed when the material and cavity frequency are resonant. **G** Schematic for in-situ referencing. The spectrum of the coupled system is divided by the spectrum of the empty cavity. **H** Transmission spectra of the coupled system referenced to the bare cavity spectrum. The spectra are computed from the data in panels B and F and are normalized for clarity. All spectra in this figure have been calculated with the transfer matrix method.

system evolves as the coupling strength is increased, and how this would appear in a *normal-*

ized transmission measurement analogous to ours (division by the bare-cavity spectrum). In the model, the coupling is tuned by increasing the Lorentz oscillator's plasma frequency (oscillator strength). As the coupling grows (Fig. S19A–H), the polariton splitting increases, the lower polariton (LP) gains contrast and becomes more pronounced, while the upper polariton (UP) weakens —an asymmetric intensity evolution that mirrors the experiment.

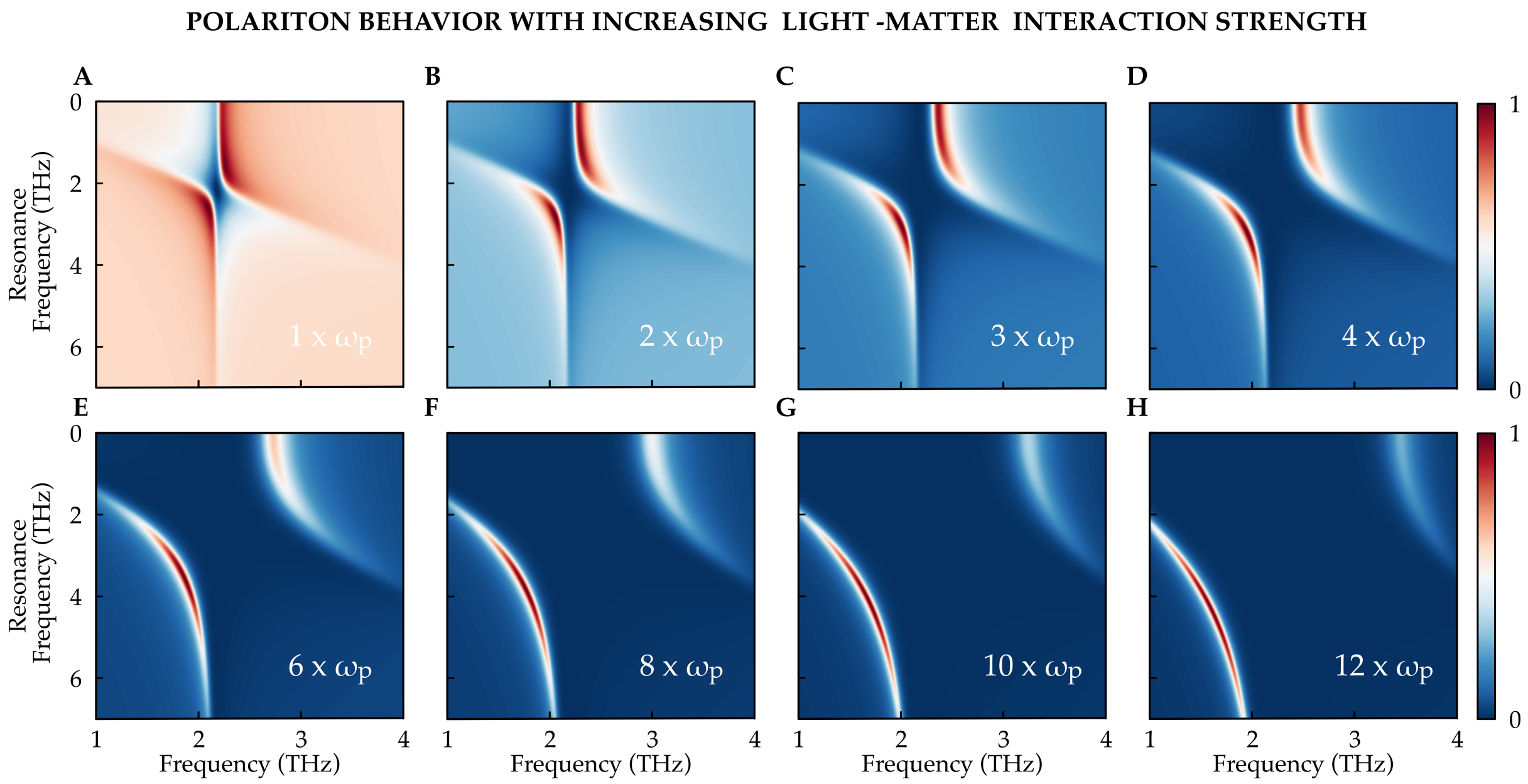

Figure S19: Weak to ultrastrong coupling of a Lorentz oscillator in a FP cavity. **A-H** Referenced transmission spectra of a Lorentz oscillator with tunable frequency ω_0 placed at the center of a FP cavity at various plasma frequencies ω_p (marked in each panel). The spectra are computed using the transfer-matrix method and normalized to the bare cavity transmission without oscillator present. The color scale is normalized for each panel separately.

7.2 Fitting of polariton energies

For a quantitative comparison with the data (Figure S20), the cavity parameters are fixed by the bare-cavity spectrum calculated with finite-elements methods, while the Lorentz-oscillator parameters (oscillator strength and damping) are treated as fit variables; the resonance frequency is set to $\omega_0(D)$, equal to the displacement-field–controlled gap. With this procedure we faithfully reproduce the observed spectra. The experimental dataset and the corresponding simulations are shown side-by-side in Fig. S20A and Fig. S20B, respectively.

In this the two-oscillator model and in the thin-film limit, the zero-detuning splitting obeys

$$\Omega_{\text{Rabi}} = 2g \approx f_p \sqrt{F}, \qquad F = \frac{t}{L_c}, \tag{4}$$

where F is the mode–volume filling fraction and L_c is the cavity length. Using the fitted parameters we obtain $g \simeq 0.99$ THz, i. e. $g/\omega_c \simeq 0.43 \pm 0.04$. As a cross check, we also estimate the interaction strength directly from the data by fitting the two observed experimental peaks at zero detuning with Lorentzian line shapes and taking half their separation. We obtain a similar $g/\omega_c \approx 0.43$ consistent with the FP analysis.

An important benefit of the FP analysis is that it also provides the differential absorption

$$\Delta A = A(\omega, D) - A_{\text{empty cavity}}(\omega), \tag{5}$$

allowing us to quantitatively locate the polariton energies. In Fig. 3D of the main text, we plot the LP/UP lines extracted from this model.

This analysis places our system robustly in the ultrastrong coupling (conventionally $g/\omega_c > 0.1$).

Finally, the compelling agreement of the data with the FP model underscores that a Lorentzian matter resonance is required to explain the observations, reinforcing our interpretation in terms of cavity-induced excitonic pairing.

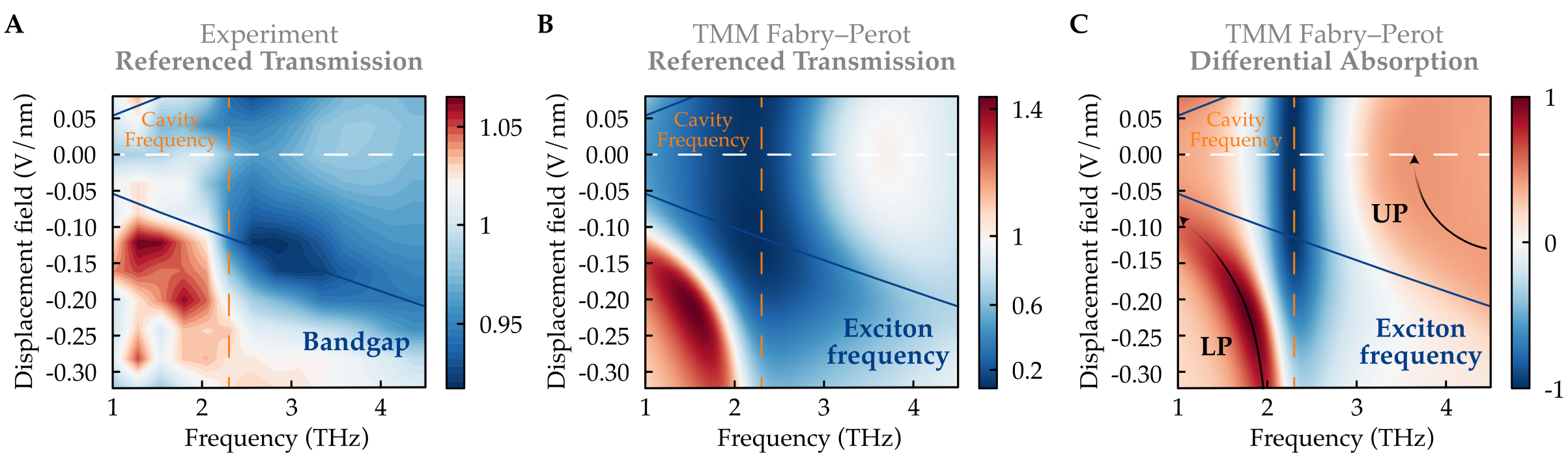

Figure S20: **A** *Experimental data*. Referenced transmission spectra for $D \neq 0$ and $n = 0$. Temperature = 5 K. **B** Simulated referenced transmission spectra using the transfer matrix method. Interaction of a cavity mode in a Fabry–Pérot setting with an exciton defined by the Lorentz model. **C** Differential absorption $\Delta A = A(\omega, D) - A_{empty\ cavity}(\omega)$. The black lines are fits to the maxima in ΔA, which identify the position of the upper (UP) and lower (LP) polaritons.

Lastly, to overcome the high dynamic range that visually suppresses the weaker UP branch in a global colormap in Fig. 3, we have isolated the LP and UP regions and applied independent color scales to each (Fig. S21).

Overlaid on this plot are the LP and UP lines obtained from the transfer-matrix modelling. When viewed with this optimized dynamic range, it is clear that the experimental peaks con-

sistently track the modeled UP and LP lines.

We reiterate that the data in Fig.3 is referenced—that is, divided by the strongly peaked bare-cavity spectrum. Mathematically, dividing a double-peaked spectrum (the coupled polaritons) by a strongly peaked function (the bare cavity resonance) inherently shifts the apparent local maxima of the resulting ratio away from the true physical resonance poles (see the arrows in Fig. S11). Consequently, the apparent peaks visible in Fig. S21 cannot and should not perfectly overlap with the actual energy levels of the lower and upper polaritons.

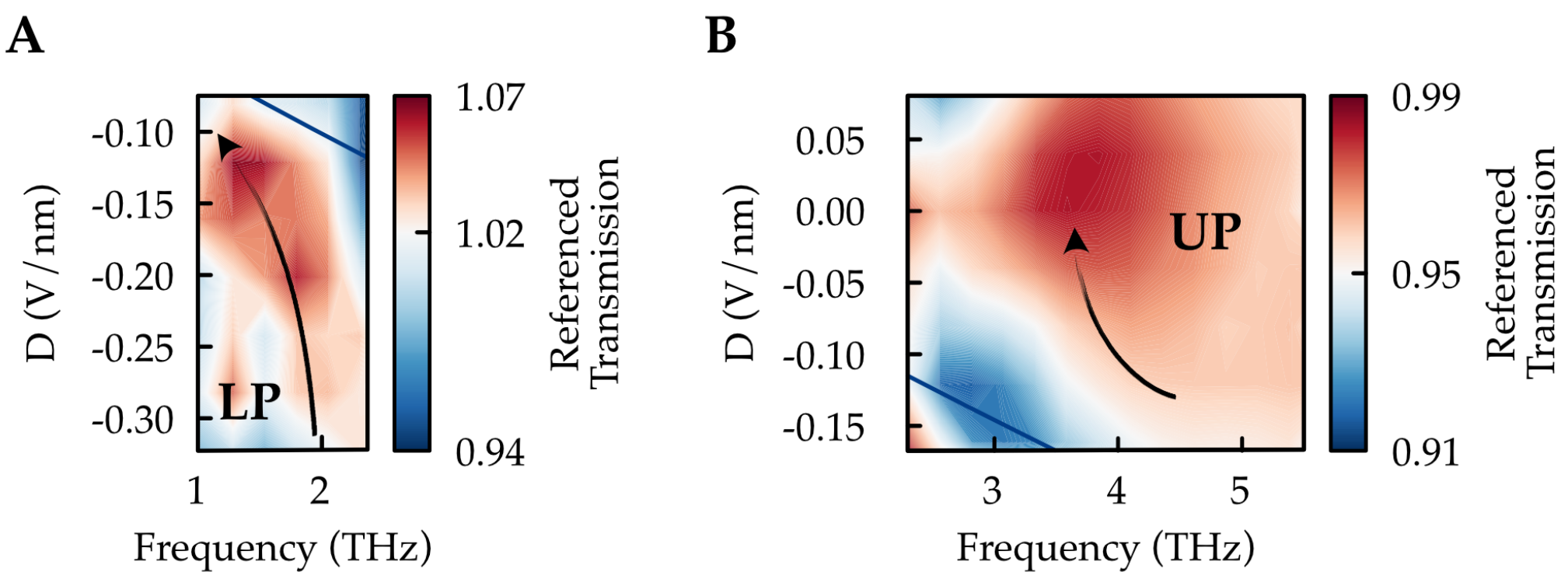

Figure S21: Polariton dispersion for LP and UP, adapted from Figure 3 of the main text A Lower Polariton dispersion. **B** Upper Polariton dispersion.

7.3 Connection to the Fano effect

Our simulations clarify how our system is related to, yet distinct from, the conventional Fano effect [11]. In the broadest sense, Fano physics describes a discrete excitation coupled to a continuum. In its usual *weak-coupling manifestation*, this interaction produces a *single* resonance that is shifted, broadened, and often rendered asymmetric through interference [12, 13]; it does not generate a true normal-mode splitting. By contrast, our data exhibit two *dispersive* branches whose separation is minimal at zero detuning and that follow an avoided crossing as the BLG gap is tuned. This behavior requires two hybrid modes in the optical response, rather than a single Fano-distorted resonance.

This distinction is borne out directly by the transfer-matrix calculations in Fig. S22. A Lorentzian dielectric response, corresponding to a discrete resonance, reproduces the observed asymmetric LP/UP dispersion and avoided crossing (Fig. S22A–C). By contrast, a semi-infinite absorption edge—even when augmented by a pronounced edge enhancement resembling a van Hove singularity—yields only Fano-like reshaping of the cavity line, but no polariton formation (Fig. S22D–F).

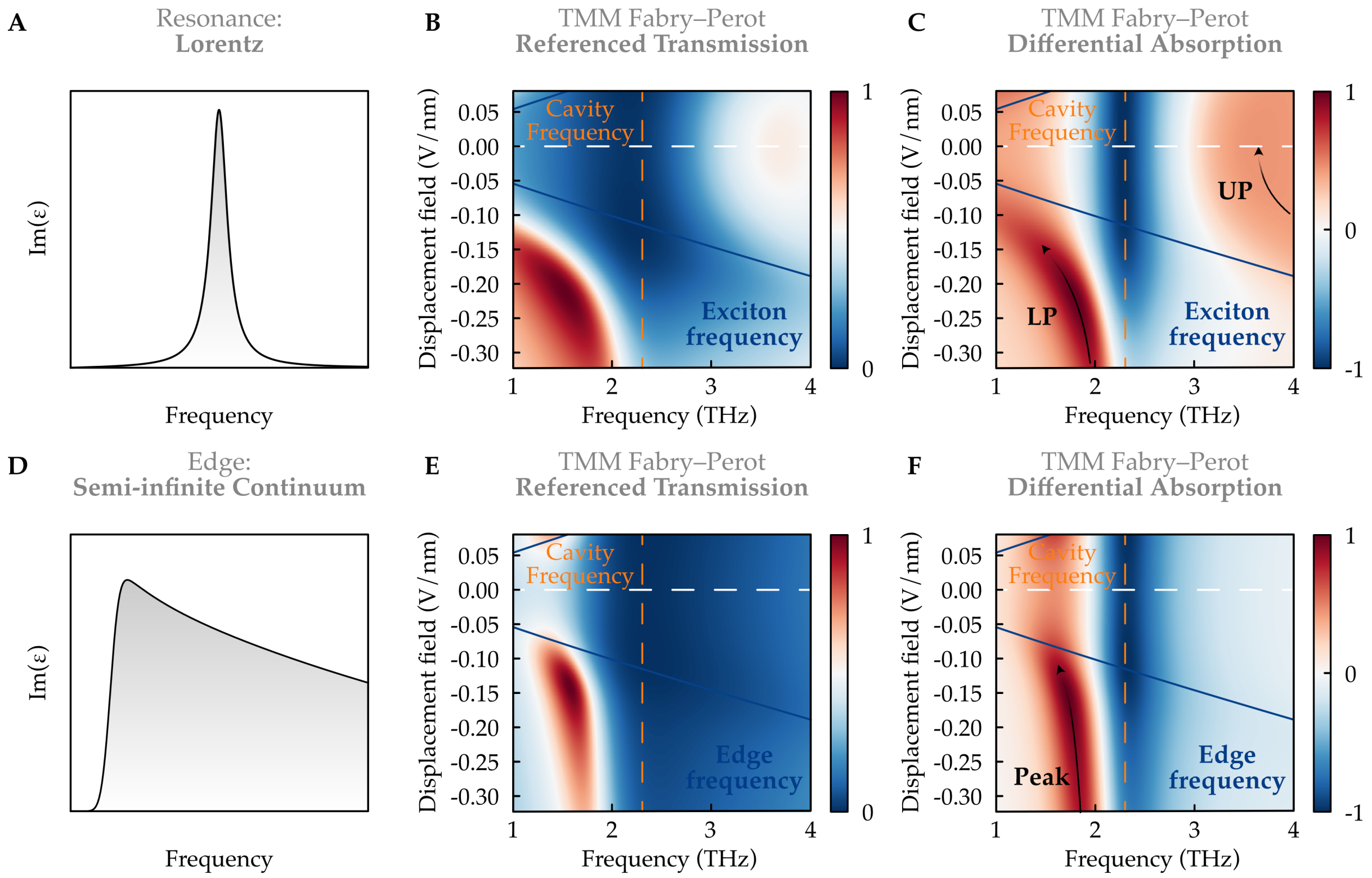

Figure S22: Electrodynamics of a Lorentzian resonance and a continuum in a FP cavity. A/D Dissipative part of the dielectric constant of a Lorentz oscillator **A** and of a continuum with an edge enhancement **D**. **B/E** Transmission spectra of a FP cavity containing at its center either a Lorentz oscillator of variable frequency (**B**) or a material with continuum absorption (**E**). The spectra are calculated using the transfer-matrix method and are normalized to the bare cavity transmission spectrum. **C/F** Differential absorption spectra computed for the same parameters as in **B/E**.

The reason is fundamental. In transfer-matrix simulations, the dielectric function $\epsilon(\omega)$ is specified *a priori* and remains fixed. A continuum-only transfer-matrix model can therefore reproduce the Fano-like reshaping of a cavity resonance, but it cannot redistribute spectral weight within the continuum so as to generate a new discrete mode below the continuum edge. That missing ingredient is captured by the quantum model discussed in the main text and introduced in detail in SI 8, where the semi-infinite electron–hole continuum is strongly coupled to the cavity. In that case, hybridization can expel one mode below the continuum threshold, producing a discrete bound state, while a second bright branch remains above the edge. This is precisely the LP/UP phenomenology observed experimentally. Our spectra therefore require, and our combined modeling corroborates, the emergence of a discrete bound mode that is absent in the bare off-resonant response and arises through cavity-induced hybridization with the interband continuum.

This comparison motivates the quantum treatment introduced below. Transfer-matrix simulations are useful for testing whether a prescribed optical response is sufficient to reproduce

the data, but they cannot describe the cavity-induced reorganization of the continuum itself. To capture that effect, one must use a model in which both the cavity field and the electron–hole continuum are treated as dynamical degrees of freedom that hybridize self-consistently, as in the quantum model.

For clarity, the simulations in Fig. S22 are shown over a restricted spectral range that isolates the relevant physics and avoids visual clutter from higher-order Fabry–Pérot modes absent in the bow-tie resonator.

7.4 Transfer-matrix simulations

The spectra presented in the previous sections are calculated with the transfer matrix (TM) formalism [14], appropriate for layered (one-dimensional) electromagnetic systems. Briefly, in a TM simulation, each material layer is represented by a 2×2 matrix encoding the electromagnetic boundary conditions and propagation inside the material for s- and p-polarized light at frequency ω (with appropriate dielectric constants). The response of the arbitrary material stacks can be simulated in this way by simply multiplying the matrices. In our case, the material stack is schematically shown in Fig. S18E and consists of two metallic mirror forming the cavity, an excitonic film with variable frequency ω_0 at its center and vacuum spacers in-between. The mirrors of thickness d_{mirror} are characterized by a Drude model

$$\varepsilon_{\text{mirror}}(\omega) = \varepsilon_\infty - \frac{\omega_p^2}{\omega^2 + i\Gamma\,\omega}, \tag{6}$$

where we have introduced the high-frequency dielectric constant ε_∞, damping Γ, and plasma frequency ω_p. Both the plasma frequency and the mirror thickness control the reflectivity of the mirrors and thus the cavity losses and line width.

8 Quantum model for ultrastrong light-matter coupling

In order to study the cavity-induced bound state and the associated polaritonic response, we introduce a minimal model (similar to the work on intersubband systems [15, 16]) in which the electrons and holes have a quadratic dispersion

$$\epsilon^c_{\mathbf{k}} = -\epsilon^v_{\mathbf{k}} + E_g = \frac{\hbar^2 k^2}{2m}. \quad (7)$$

and couple to a single terahertz cavity mode at frequency ω_c. Here, the electronic dispersion in bilayer graphene is modeled using an effective mass $m \approx 0.04 m_0$ while a tunable bandgap E_g defines the separation between the conduction and valence bands. All vertical interband transitions are assumed to couple to the cavity mode, with a light–matter coupling strength g that is independent of k and reduces with decreasing E_g as observed in bilayer graphene [17]. Within the Coulomb gauge and neglecting electron–hole interactions, the full Hamiltonian of the system is given by

$$H = \hbar\omega_c\, a^\dagger a + \sum_{\mathbf{k}} \epsilon_{c,\mathbf{k}} c^\dagger_{\mathbf{k}} c_{\mathbf{k}} + \sum_{\mathbf{k}} \epsilon_{v,\mathbf{k}} v^\dagger_{\mathbf{k}} v_{\mathbf{k}} + \hbar \sum_{\mathbf{k}} g(a + a^\dagger)\left(v^\dagger_{\mathbf{k}} c_{\mathbf{k}} + c^\dagger_{\mathbf{k}} v_{\mathbf{k}}\right) + \hbar D(a + a^\dagger)^2. \quad (8)$$

Here, $a^\dagger$ (a) creates (annihilates) a cavity photon, while $c^\dagger_{\mathbf{k}}$ and $v^\dagger_{\mathbf{k}}$ create electrons in the conduction and valence bands, respectively. The term proportional to D represents the diamagnetic (or A^2) contribution. Although a fully gauge-invariant treatment would include this term, predictions indicate that unlike conventional semiconductors bilayer graphene can be well approximated without it [18]. Moreover, our experimental observation of no discernible cavity frequency shift upon doping suggests that omitting the A^2 term might be reasonable under our setting. Thus, we focus solely on the light–matter coupling parameter g to capture the essential physics of photon-mediated pairing, while we abstain from any discussions on the ground state properties.

To study this Hamiltonian, which explicitly includes counter-rotating terms, we introduce electron–hole coherence operators:

$$b^\dagger_{\mathbf{k}} = c^\dagger_{\mathbf{k}} v_{\mathbf{k}}, \quad (9)$$

which, in the dilute limit of few excitations, approximately obey bosonic commutation rela-

tions [19]

$$\left[b_{\mathbf{k}}, b_{\mathbf{k}'}^{\dagger}\right] = \delta_{\mathbf{k},\mathbf{k}'}(v_{\mathbf{k}}^{\dagger}v_{\mathbf{k}'} - c_{\mathbf{k}'}^{\dagger}c_{\mathbf{k}}) \approx \delta_{\mathbf{k},\mathbf{k}'}. \tag{10}$$

Such an approximation allows us to apply Bogoliubov theory and can be justified a posteriori by considering the number of particle-hole pairs in the Bogoliubov vacuum. However, in general, we can expect this to be a reasonable approximation in the case of a non-vanishing bandgap. With this bosonization, the Hamiltonian can be recast as

$$H \approx \hbar\omega_c a^{\dagger}a + \sum_{\mathbf{k}} \epsilon_{\mathbf{k}} b_{\mathbf{k}}^{\dagger}b_{\mathbf{k}} + \hbar\sum_{\mathbf{k}} g(a + a^{\dagger})(b_{\mathbf{k}} + b_{\mathbf{k}}^{\dagger}), \tag{11}$$

where $\epsilon_{\mathbf{k}} = \epsilon_{c\mathbf{k}} - \epsilon_{v\mathbf{k}}$ is the electron-hole dispersion. Note that, formally, this Hamiltonian is a generalization of the bosonic Fano model [11] including counter-rotating terms. Since the Hamiltonian is quadratic in the bosonic operators, it can be diagonalized exactly via a Hopfield-Bogoliubov transformation. To this end, one defines polariton operators

$$p_i = x_i a + y_i a^{\dagger} + \sum_{\mathbf{k}} \Big(z_{i\mathbf{k}}\, b_{\mathbf{k}} + w_{i\mathbf{k}}\, b_{\mathbf{k}}^{\dagger} \Big), \tag{12}$$

with coefficients x, y, $z_{\mathbf{k}}$, and $w_{\mathbf{k}}$ to be determined. Inserting the ansatz into the Heisenberg equation of motion

$$\frac{\hbar}{i}\frac{d}{dt}p_i = [H, p_i] \tag{13}$$

yields the following set of eigenvalue equations for the eigenenergies ϵ_i and the expansion coefficients

$$\epsilon_i x_i = \epsilon_c x_i + \hbar g \sum_{\mathbf{k}} \Big(z_{i\mathbf{k}} - w_{i\mathbf{k}} \Big), \tag{14}$$

$$\epsilon_i y_i = -\epsilon_c y_i + \hbar g \sum_{\mathbf{k}} \Big(z_{i\mathbf{k}} - w_{i\mathbf{k}} \Big), \tag{15}$$

$$\epsilon_i z_{i\mathbf{k}} = \epsilon_{\mathbf{k}} z_{i\mathbf{k}} + \hbar g (x_i - y_i), \tag{16}$$

$$\epsilon_i w_{i\mathbf{k}} = -\epsilon_{\mathbf{k}} w_{i\mathbf{k}} + \hbar g (x_i - y_i). \tag{17}$$

These equations describe the hybridization between the cavity photon and the electron–hole excitations.

A notable feature of the 2D system is the ultraviolet divergence appearing in the photonic self-energy. To remedy this, a renormalization procedure is necessary (see Levinsen *et al.* [20]

for a discussion of this issue in the case of the rotating-wave approximation). Following their approach, we treat the cavity energy ϵ_c as a bare parameter that must be renormalized. In the case of a large bandgap $E_g \gg \epsilon_c$, the lowest polariton mode can be safely assumed to be at the energy of the bare cavity. In our system, the limiting case of large bandgap $E_g \gg \omega_c$ provides an exactly solvable limit. Here, the energy of the lowest polariton mode corresponds to the physical cavity energy $\epsilon_{p,c}$ yielding the renormalization equation for ϵ_c in terms of the physical frequency $\epsilon_{c,p}$ via the condition

$$\epsilon_{c,p}^2 - \epsilon_c^2 - 2\epsilon_c \Sigma(0) = 0, \tag{18}$$

where $\Sigma(\epsilon)$ is the photon self-energy given by

$$\Sigma(\epsilon) = \hbar^2 \sum_{\mathbf{k}} \left(\frac{g^2}{\epsilon - \epsilon_{\mathbf{k}}} - \frac{g^2}{\epsilon + \epsilon_{\mathbf{k}}} \right). \tag{19}$$

We explicitly fix the momentum cut-off to the physical momentum scale where the bands of bilayer graphene cease to be parabolic $\sim 300\,\text{meV}$.

The eigenvalue problem is solved numerically using a Gauss–Legendre quadrature rule to perform the required integrals, with the normalization of the Hopfield coefficients enforced by the bosonic commutation relations. Importantly, the Hopfield-Bogoliubov shifts the ground state from the uncoupled state $|0\rangle$ to $|vac\rangle$ such that

$$p_i |vac\rangle = 0, \tag{20}$$

which contains virtual photonic and matter excitations and finite light-matter entanglement [19]. In the excited states, the numerical diagonalization reveals substantial mixing between the cavity mode and the excitonic states, especially near the band edge, thereby confirming the role of the cavity in inducing excitonic features.

The spectral properties of the hybrid system are encoded in the retarded photonic Green's function

$$\mathcal{G}(t) = -i\theta(t) \langle [a(t), a(0)^\dagger] \rangle_{vac}, \tag{21}$$

where the expectation value is taken with respect to the Bogoliubov vacuum state $|vac\rangle$ and $\theta(t)$ denotes the Heaviside step function. The experimentally relevant photonic spectral func-

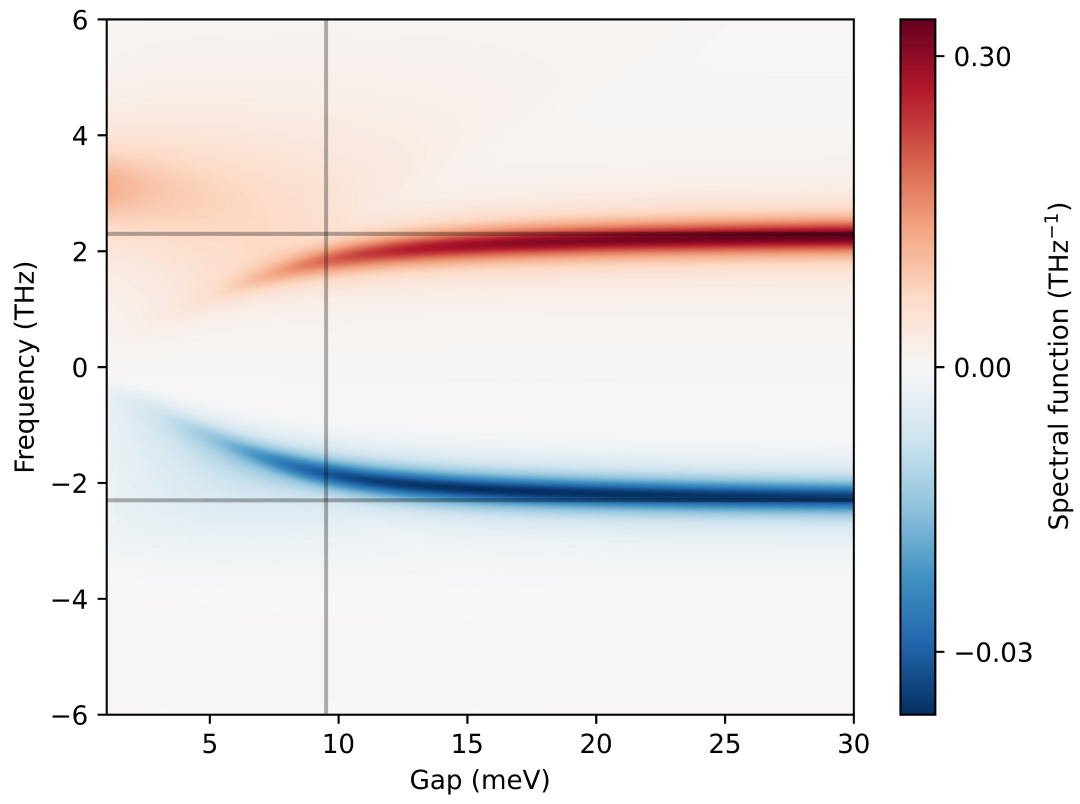

Figure S23: Photonic spectral function evaluated a as function of bandgap with (same parameters as shown in the main text Fig. 4B). Here, also negative frequency contributions are shown. The gray lines indicate the positive and negative cavity frequencies (horizontal) as well as the cavity energy (vertical), respectively.

tion is then given by

$$\mathcal{A}(\epsilon) = -\frac{1}{\pi} \operatorname{Im} \mathcal{G}(\epsilon) = -\frac{1}{\pi} \operatorname{Im} \sum_{i \in \{+\}} \frac{|x_i^2|}{\epsilon - \epsilon_i + i\tau} - \frac{|y_i^2|}{\epsilon + \epsilon_i + i\tau}, \tag{22}$$

where τ is the phenomenological cavity broadening and the sum extends over all positive energy Bogoliubov modes. We explicitly checked that there are no energetic instabilities in the model such that all physical modes have positive energy. Note that the expression of the spectral function has poles at both positive and negative energies (weighted by the normal and anomalous cavity Hopfield coefficients, respectively) implying spectral weight at positive and negative energies (see Fig. S23). The presence of spectral weight at negative energies is a direct consequence of the inclusion of counter-rotating terms in the light-matter coupling. However, only the positive-energy spectrum is experimentally accessible. We choose the broadening to be 1 meV (smaller than the experimental value to highlight the different features). To construct the referenced spectral function shown in Fig. 3E of the main text, we additionally use a slightly skewed Lorentzian cavity lineshape, motivated by the finite-element simulations, in order to better reproduce the observed intensity ratio of the lower- and upper-polariton branches. This phenomenological step affects only the plotted line shape, not the underlying eigenmode energies.

To further elucidate the nature of the discrete state induced by the cavity, we analyze its matter content via the real-space electron–hole correlation function of the lowest-energy po-

lariton state $|LP\rangle$ defined by

$$\tilde{\gamma}(r) = \langle c_r^\dagger c_r v_0 v_0^\dagger \rangle_{LP} - \langle c_r^\dagger c_r v_0 v_0^\dagger \rangle_{vac}, \tag{23}$$

representing the probability to find a conduction-band electron at distance r from a valence-band hole. The operator ordering in the expectation values is derived from the normal ordered form

$$\langle c_r^\dagger h_0^\dagger h_0 c_r \rangle = \langle c_r^\dagger c_r v_0 v_0^\dagger \rangle, \tag{24}$$

where we introduced the valence-band hole operator $h_0^\dagger = v_0$. In terms of the Hopfield coefficients, γ is found to be

$$\tilde{\gamma}(r) = \sum_{\mathbf{k},\mathbf{q}} e^{i(\mathbf{k}-\mathbf{q})\cdot\mathbf{r}}\, z_{\mathbf{k}} z_{\mathbf{q}}^* - \sum_{\mathbf{k},\mathbf{q}} e^{i(\mathbf{k}-\mathbf{q})\cdot\mathbf{r}}\, w_{\mathbf{k}} w_{\mathbf{q}}^*, \tag{25}$$

similar to the expression found in ref. [15]. Owing to the radial symmetry, the real-space profile is conveniently obtained by applying the Bessel transform

$$\sum_{\mathbf{k}} f(k)\, e^{i\mathbf{k}\cdot\mathbf{r}} = \int f(k)\, J_0(kr)\, k\, dk, \tag{26}$$

where $J_0(\xi)$ is the 0-th order Bessel function of the first kind. In the main text, we plot the weighted correlation function

$$\gamma(r) = M_{LP}\tilde{\gamma}(r), \tag{27}$$

where M_{LP} is the integrated matter content of the LP mode.

The short-range behavior of the correlation function presented in the main text is controlled by the cut-off in momentum space. If we send the cut-off momentum to higher values, the light-matter coupling approaches a true contact-like interaction independent of k. If we evaluate the model in this case, the electron-hole correlation function at each bandgap is well approximated by a modified Bessel function $K_0(\kappa r)$ (see Fig. S24), which is the bound-state wavefunction for a contact-like potential $V(r) \propto \delta(r)$ in two spatial dimensions. This observation further supports our interpretation that the cavity coupling mediates an electron–hole interaction, distinct from the Coulomb potential $V(r) \propto \frac{1}{r}$.

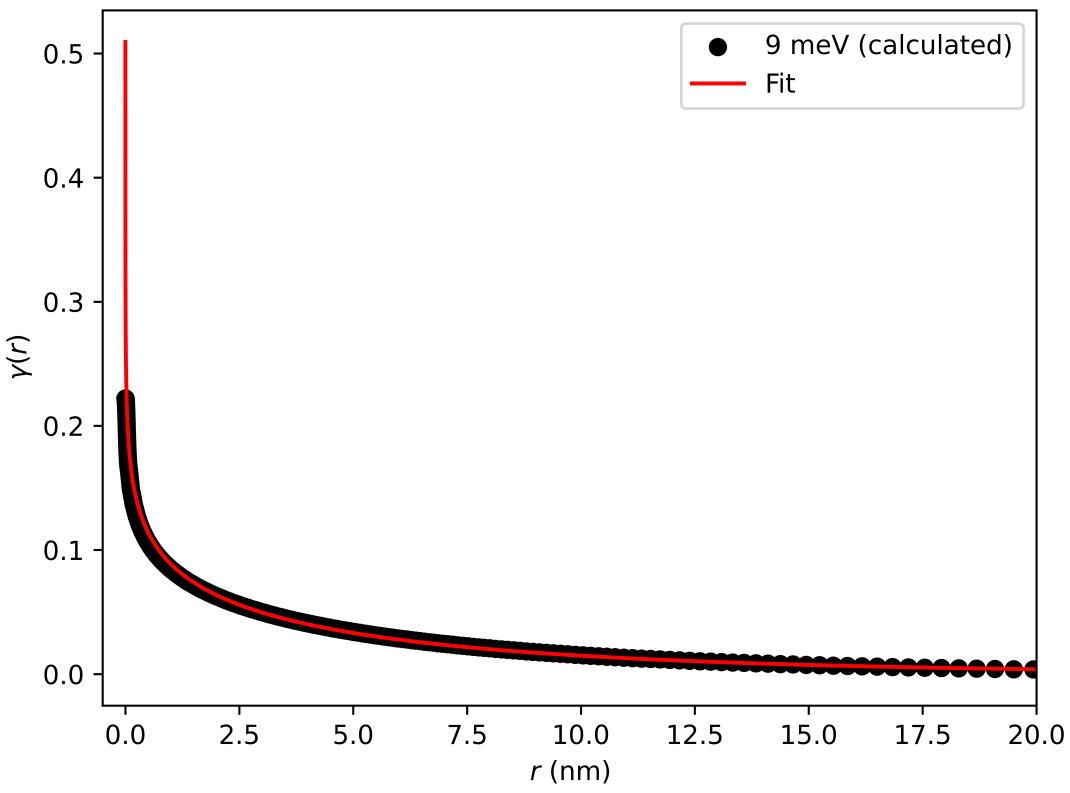

Figure S24: Electron-hole correlation function at bandgap 9 meV and fit with modified Bessel function $K_0(\kappa r)$. The correlation function is computed with a momentum cut-off $\Lambda \to \infty$.

8.1 Weak- and strong-coupling limits of the discrete–continuum model

To show explicitly how the same quantum model interpolates between the conventional weak-coupling Fano regime and the strong-coupling regime relevant to our experiment, we evaluate the photonic spectral function for weak (Fig. S25A,B, $g = g_0/8$) and strong coupling (Fig. S25C,D, $g = g_0$), where g_0 is the coupling strength used in the main text. To make the differences between the two regimes more apparent, we reduce the phenomenological line broadening compared to Fig. 4 of the main text.

In the *weak-coupling* case, the cavity mode displays the conventional Fano behavior expected for a discrete mode coupled to a continuum. Once the band edge is tuned below the cavity energy (crossing point of the two white lines in Fig. S25A), the cavity resonance enters the continuum and becomes broader, with only a small shift in its spectral position. This is illustrated by the line cuts in Fig. S25B, where the bare cavity resonance and the continuum-dressed resonance remain both single-peaked. The quantum model therefore recovers the usual weak-coupling Fano phenomenology rather than a true normal-mode splitting [11].

In the case of *strong coupling* (Fig. S25C/D), the spectrum behaves in a radically different way. As the continuum edge approaches the cavity energy (Fig. S25C), the discrete state bends downward and evolves into the LP, while the continuum acquires oscillator strength and develops into the UP mode. This behavior is fully consistent with the discussion in the main text (Fig. 4). The line cuts in Fig. S25D clearly resolve the two polaritonic modes that emerge when the continuum hybridizes strongly with the cavity mode. Importantly, once the LP lies below the continuum edge, decay into the continuum is no longer possible for that mode, so

its linewidth remains nearly cavity-limited. This is highlighted by the near-perfect overlap between the LP peak and the red-shaded area corresponding to the bare cavity spectrum shifted to the LP position in Fig. S25D, .

Taken together, these calculations show that the same quantum model captures both the conventional weak-coupling Fano regime and the strong-coupling regime relevant to our experiment. At small g, it reproduces the conventional Fano regime of a single shifted and broadened resonance. At large g, relevant to our experiment, the semi-infinite continuum and strong hybridization allow a discrete bound mode to emerge below threshold, yielding distinct LP and UP branches and an avoided crossing (see also the discussion in ref. [15]).

By combining a simple two-band model with a Hopfield-Bogoliubov diagonalization procedure, we explain the mechanism by which a THz cavity mode can induce and control excitonic pairing, in close correspondence with experimental observations.

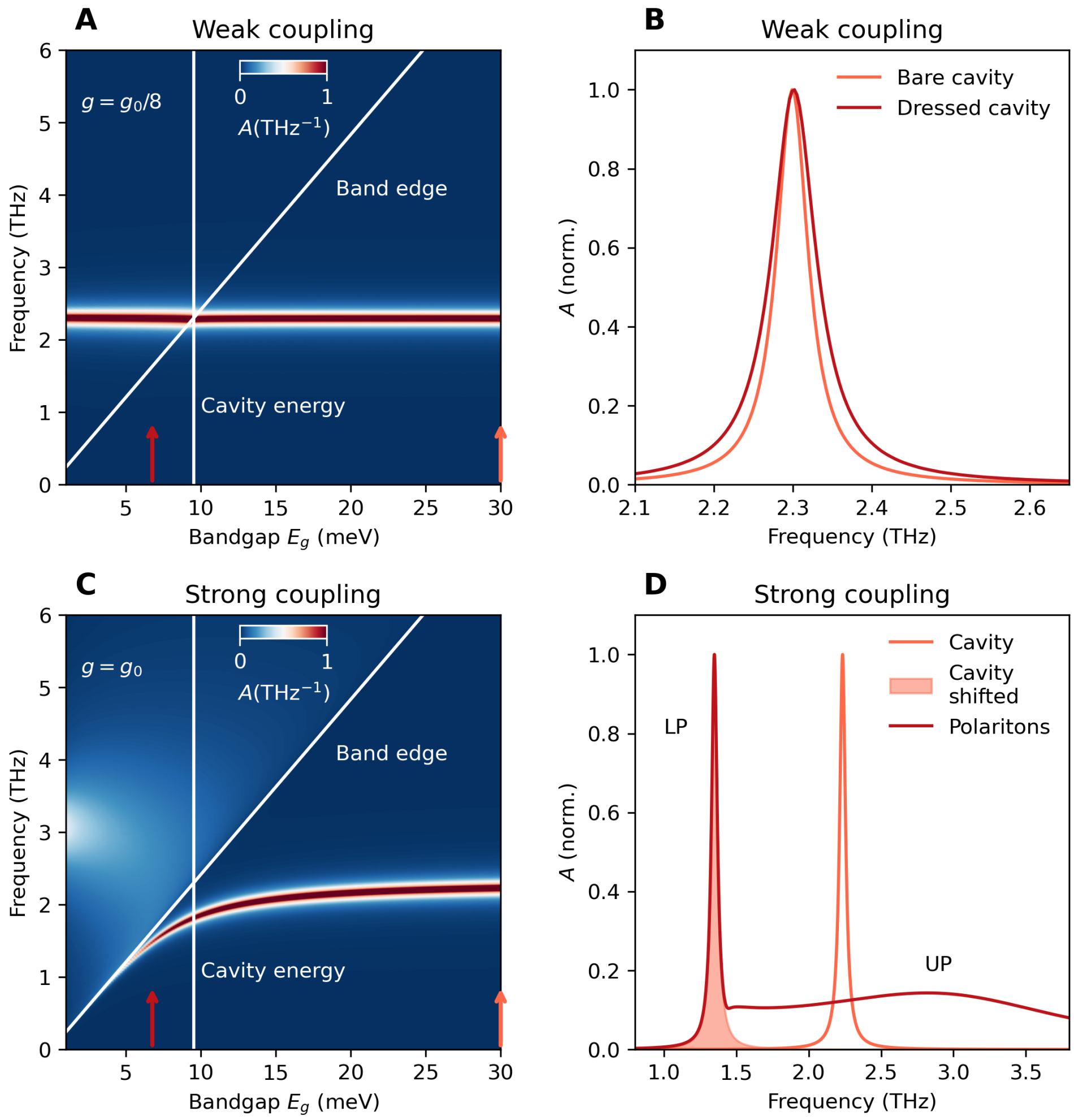

Figure S25: Weak and strong coupling limits of the model. **A** Spectral function as a function of bandgap and frequency for a reduced coupling constant $g = g_0/8$, where g_0 is the coupling employed in the main text. White lines mark the band edge as well as the cavity energy. **B** Line-cuts extracted from panel A at two distinct bandgaps E_g (marked by the vertical arrows in panel A). The spectra are normalized to their maxima. Dark red line: spectrum for $E_g \approx 6\,\text{meV} < \hbar\omega_c$, where ω_c is the cavity frequency, corresponding to the bare cavity mode. Light red line: spectrum for $E_g \approx 30\,\text{meV} > \hbar\omega_c$. **C** Same as panel A, but for stronger coupling $g = g_0$. The scaling of the color map is identical in panels A and C. **D** Same as panel B, but extracted from panel C. Dark red line: spectrum of the cavity mode. Light red line: spectrum exhibiting polariton modes. The red-shaded area corresponds to the cavity spectrum shifted to the position of the LP. The cavity broadening is set to 0.1 THz in all spectra.

9 Finite-element simulations

To simulate the bare cavity transmission spectrum and the mode profile, we performed electromagnetic finite-element simulations in the frequency domain using COMSOL Multiphysics (version 6.2). We model the bare cavity as a patterned gold sheet (200 nm thick) sandwiched between two silicon blocks (representing the substrate and the top lens). We do not model the oxide layers and air gaps in the structure. But since these layers are non-absorptive and far thinner than the wavelength, the error introduced is small. We define periodic boundary conditions and excite the system with an incident linearly polarized wave. The resulting transmission spectrum and mode profile are depicted in Fig. 2E and Fig. 1D of the main text, respectively. In addition, we model the doped cavity corresponding to the experiment where we introduce charge carriers into bilayer graphene. To achieve this, we place a material sheet with a Drude-type conductivity 200 nm below the resonator plane. We then vary the plasma frequency to mimic the variable charge density in the experiment. The results are depicted in Fig. 2F (spectra) and 2G (selected mode profiles) of the main text. In the spectra shown, we divide the transmission at different plasma frequencies by the bare cavity spectrum.

10 Plasmon dispersion in dual-gated BLG devices

In the main text, we attribute the transmission dip observed upon doping BLG (see Fig. 2D/E) to enhanced dissipation and impedance mismatch. An alternative mechanism involving plasmon modes in BLG can be ruled out. Following the model of Tomadin et al. [21], we have calculated the plasmon dispersion in a dual-gated device with vanishing bandgap. The plasmon velocity s is

$$s = \sqrt{\frac{4e}{m}\frac{(V_{\mathrm{BG}} - V_{\mathrm{BG},0})d_{\mathrm{TG}}}{d_{\mathrm{TG}} + d_{\mathrm{BG}}}} = \sqrt{\frac{4e}{m}\frac{(V_{\mathrm{TG}} - V_{\mathrm{TG},0})d_{\mathrm{BG}}}{d_{\mathrm{TG}} + d_{\mathrm{BG}}}} \tag{28}$$

where e is the electron charge, $m \approx 0.04m_0$ is the effective mass of conduction-band electrons in BLG (m_0 is the bare electron mass), d_{TG} (d_{BG}) is the hBN thickness for the top- (back-) gate, and $V_{\mathrm{TG},0}$, $V_{\mathrm{BG},0}$ denote neutrality offsets. The resulting plasmon dispersion is linear,

$$\omega_{\mathrm{Plasmon}} = sk, \tag{29}$$

where k is the wave vector. At fixed geometry (fixed k), the plasmon dispersion is thus proportional to the square root of the gate voltage (and corresponding densities), $\omega_{\mathrm{Plasmon}} \propto \sqrt{V_{\mathrm{BG}}}$. In Fig.S26, we plot the calculated plasmon frequency versus back-gate voltage across the experimentally accessed density range, for different lateral dimensions w that set the in-plane wave vector via $k = \frac{2\pi}{w}$. Because the smallest lithographic feature in our resonator is a 2 µm gap, the corresponding plasmon branch remains below the cavity frequency and only grazes the edge of our measurement bandwidth. Only an unrealistically small feature size of 1.4 µm (dark red dotted-dashed line in Fig. S26) would place the plasmon within the cavity frequency range at relevant densities.

The absence of any gate-induced (density-dependent) dispersion in the observed dip (Fig. 2D/E), together with the clear spectral mismatch of potential plasmon resonances in our heterostructure, rules out a plasmonic origin of the observed spectral features.

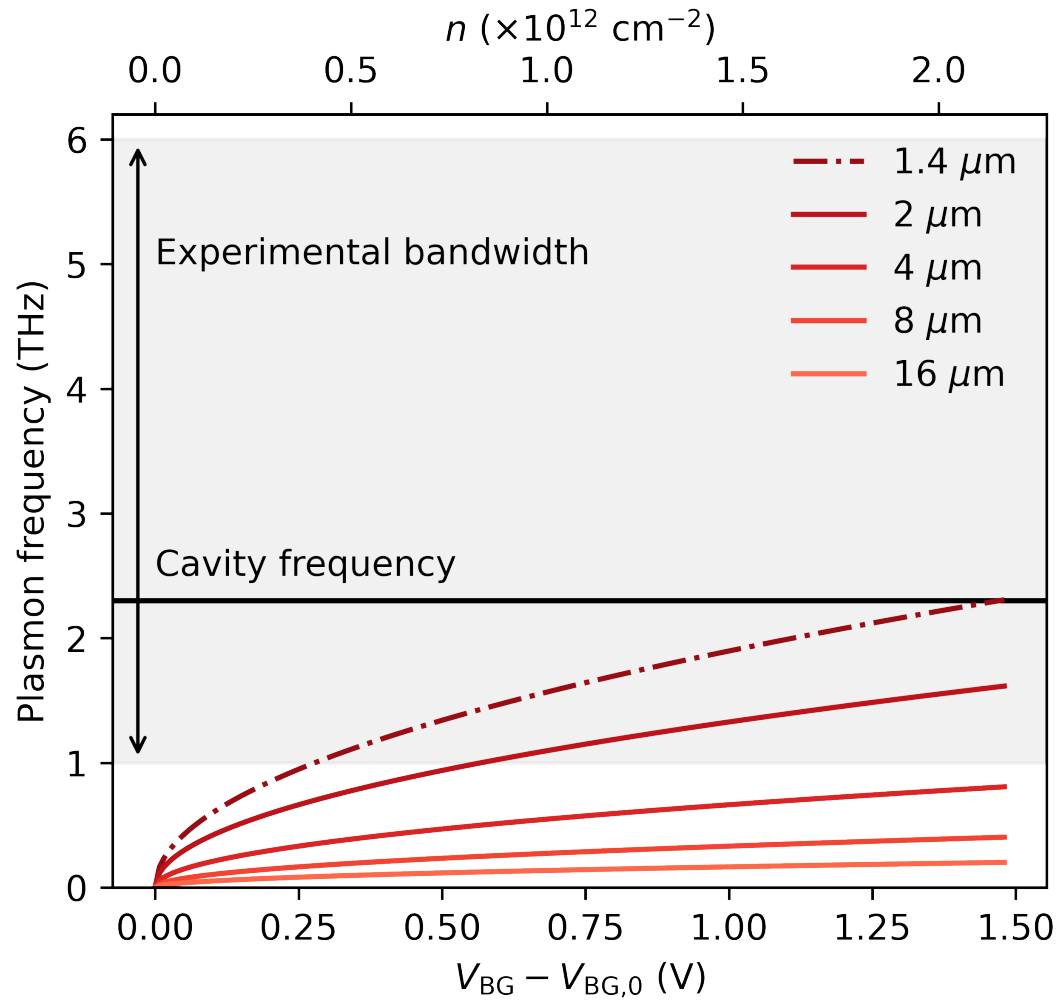

Figure S26: Calculated gate-dependent plasmon dispersion in dual-gated BLG device following Eq. (28). The wave vector is determined by geometric length scales of the resonator. Note that the smallest feature of the resonator in D1 and D2 is 2 µm. The experimental band width is limited between 1 and6 THz and the cavity resonance is at 2.3 THz (black line). The back gate voltage axis corresponds to the voltages applied experimentally (while keeping $D = 0$) inducing the electron density n.

11 Self-consistent calculation of field-induced bandgap

While the out-of-plane displacement field D sets the energy scale of the induced bandgap E_g of bilayer graphene, E_g is not merely the potential difference between the two layers, but is determined by its interplay with the screening of the electrons. Following ref. [8, 22, 23], we solve this problem self-consistently in the Hartree approximation. Within a low-energy tight-binding approximation, the bands of bilayer graphene are modeled by the Hamiltonian

$$H = \frac{1}{2m} \begin{pmatrix} U/2 & (p^\dagger)^2 \\ p^2 & -U/2 \end{pmatrix}, \tag{30}$$

where we introduced the effective mass $m \approx 0.04m_0$, the complex momentum $p = p_x + ip_y$ and the interlayer energy difference U. The basis states for such a matrix representation are $(|\psi_{tA}\rangle, |\psi_{bB}\rangle$, where $|\psi_{tA}\rangle$ and $|\psi_{bB}\rangle$ are the amplitudes on A sites of the top layer and B sites of the bottom layer, respectively. These are the non-dimer sites without direct neighbors in the other layer. Using the plate-capacitor model for the bilayer system, the on-site potential difference U is given by the sum of contributions of the displacement field D and the charge imbalance δn

$$U = \frac{deD}{\epsilon_0 \epsilon} + \frac{de^2}{2\epsilon_0 \epsilon}\delta n = U_0(D) + \frac{de^2}{2\epsilon_0 \epsilon}\delta n, \tag{31}$$

where $d = 0.34\,\text{nm}$ is the interlayer separation, ε_0 is the vacuum permittivity, ε and is the out-of-plane dielectric constant of bilayer graphene. The charge imbalance between the two graphene layers is

$$\delta n = \frac{mU}{\pi} \ln \frac{U}{4\gamma_1}, \tag{32}$$

where $\gamma_1 \approx 0.2\,\text{eV}$ is the interlayer hopping amplitude yielding the following self-consistent equation

$$U(D) = U_0(D) + \frac{de^2}{2\epsilon_0\epsilon} \frac{mU}{\pi} \ln \frac{U}{4\gamma_1}, \tag{33}$$

which we solve numerically for each value of D using the experimental thicknesses of hBN obtained from AFM measurements. The result is the curve in Fig. 3D and Fig. 5A,B,C,D of the main text.

References

[1] S. Rajabali, S. Markmann, E. Jöchl, M. Beck, C. A. Lehner, W. Wegscheider, J. Faist, and G. Scalari. An ultrastrongly coupled single terahertz meta-atom. *Nat. Comm.*, 13(1):2528, 2022.

[2] L. Wang, I. Meric, P. Y. Huang, Q. Gao, Y. Gao, H. Tran, T. Taniguchi, K. Watanabe, L. M. Campos, D. A. Muller, J. Guo, P. Kim, J. Hone, K. L. Shepard, and C. R. Dean. One-dimensional electrical contact to a two-dimensional material. *Science*, 342(6158):614–617, 2013.

[3] F. Valmorra, G. Scalari, C. Maissen, W. Fu, Ch. Schönenberger, J. W. Choi, H. G. Park, M. Beck, and J. Faist. Low-Bias Active Control of Terahertz Waves by Coupling Large-Area CVD Graphene to a Terahertz Metamaterial. *Nano Lett.*, 13(7):3193–3198, 2013.

[4] L. Ren, Q. Zhang, J. Yao, Z. Sun, R. Kaneko, Z. Yan, S. Nanot, Z. Jin, I. Kawayama, M. Tonouchi, J. M. Tour, and J. Kono. Terahertz and Infrared Spectroscopy of Gated Large-Area Graphene. *Nano Lett.*, 12(7):3711–3715, 2012.

[5] T. Handa, M. Holbrook, N. Olsen, L. N. Holtzman, L. Huber, H. I. Wang, M. Bonn, K. Barmak, J. C. Hone, A. N. Pasupathy, and X. Zhu. Spontaneous exciton dissociation in transition metal dichalcogenide monolayers. *Sci. Adv.*, 10(5):eadj4060, 2024.

[6] X. Jin, V. Aglieri, Y.-G. Jeong, A. Pezeshki, L. Skokan, M. Shagar, Y. Jia, P. Bianucci, A. Ruediger, E. Orgiu, A. Toma, and L. Razzari. Enhanced Terahertz Spectroscopy of a Monolayer Transition Metal Dichalcogenide. *Adv. Funct. Mater.*, 2025.

[7] I. C. Tangen, G. A. Valdivia-Berroeta, L. K. Heki, Z. B. Zaccardi, E. W. Jackson, Ch. B. Bahr, S.-H. Ho, D. J. Michaelis, and J. A. Johnson. Comprehensive characterization of terahertz generation with the organic crystal BNA. *J. Opt. Soc. Am. B*, 38(9):2780–2785, 2021.

[8] E. McCann and M. Koshino. The electronic properties of bilayer graphene. *Reports on Progress in Physics*, 76(5):056503, 2013.

[9] C. Maissen, G. Scalari, F. Valmorra, M. Beck, J. Faist, S. Cibella, R. Leoni, Ch. Reichl, Ch. Charpentier, and W. Wegscheider. Ultrastrong coupling in the near field of complementary split-ring resonators. *Phys. Rev. B*, 90:205309, 2014.

[10] A. Pierret, D. Mele, H. Graef, J. Palomo, T. Taniguchi, K. Watanabe, Y. Li, B. Toury, C. Journet, P. Steyer, V. Garnier, A. Loiseau, J.-M. Berroir, E. Bocquillon, G. Fève, C. Voisin, E. Baudin, M. Rosticher, and B. Plaçais. Dielectric permittivity, conductivity and breakdown field of hexagonal boron nitride. *Mater. Res. Express*, 9(6):065901, 2022.

[11] U. Fano. Effects of Configuration Interaction on Intensities and Phase Shifts. *Phys. Rev.*, 124:1866–1878, 1961.

[12] M. Kroner, A. O. Govorov, S. Remi, B. Biedermann, S. Seidl, A. Badolato, P. M. Petroff, W. Zhang, R. Barbour, B. D. Gerardot, R. J. Warburton, and K. Karrai. The nonlinear Fano effect. *Nature*, 451(7176):311–314, Jan 2008.

[13] M. F. Limonov, M. V. Rybin, A. N. Poddubny, and Y. S. Kivshar. Fano resonances in photonics. *Nature Photonics*, 11(9):543–554, 2017.

[14] S. J. Byrnes. Multilayer optical calculations. 2020.

[15] E. Cortese, I. Carusotto, R. Colombelli, and S. De Liberato. Strong coupling of ionizing transitions. *Optica*, 6(3):354–361, 2019.

[16] S. S. Kumar, M. M. Parish, and J. Levinsen. Microscopic theory of excitons bound by light. *Phys. Rev. B*, 106:205414, 2022.

[17] M. F. C. Martins Quintela, N. M. R. Peres, and T. Garm Pedersen. Tunable nonlinear excitonic optical response in biased bilayer graphene. *Phys. Rev. B*, 110:085433, 2024.

[18] T. Liu and Q. J. Wang. Magnetopolariton in bilayer graphene: A tunable ultrastrong light-matter coupling. *Phys. Rev. B*, 89(12):125306, 2014.

[19] C. Ciuti, G. Bastard, and I. Carusotto. Quantum vacuum properties of the intersubband cavity polariton field. *Phys. Rev. B*, 72(11):115303, 2005. Publisher: American Physical Society.

[20] J. Levinsen, G. Li, and M. M. Parish. Microscopic description of exciton-polaritons in microcavities. *Phys. Rev. Res.*, 1:033120, 2019.

[21] A. Tomadin, M. Carrega, and M. Polini. Microscopic theory of plasmon-enabled resonant terahertz detection in bilayer graphene. *Phys. Rev. B*, 103:085426, 2021.

[22] E. McCann. Asymmetry gap in the electronic band structure of bilayer graphene. *Phys. Rev. B*, 74:161403, 2006.

[23] E. Icking, L. Banszerus, F. Wörtche, F. Volmer, Ph. Schmidt, C. Steiner, S. Engels, J. Hesselmann, M Goldsche, K. Watanabe, T. Taniguchi, Ch. Volk, B. Beschoten, and Ch. Stampfer. Transport Spectroscopy of Ultraclean Tunable Band Gaps in Bilayer Graphene. *Adv. Electron. Mater.*, 8(11):2200510, 2022.
