# Supplementary material for: Cavity-driven attractive interactions in quantum materials
Source: Nature. 2026 May 27;654(8118):361–8. doi: 10.1038/s41586-026-10609-1 (PMC13661254; doi:10.1038/s41586-026-10609-1)
Supplement: Supplementary file 2 [file 41586_2026_10609_MOESM2_ESM.pdf]

nature portfolio

Peer Review File

Cavity-Driven Attractive Interactions in Quantum Materials

Corresponding Author: Dr Tobia Nova

This file contains all reviewer reports in order by version, followed by all author rebuttals in order by version.

Version 0:

Reviewer comments:

Referee #1

(Remarks to the Author)
I reviewed the manuscript “Cavity-Driven Attractive interactions in Quantum Materials” by Helmrich et al. A) (Summary) The manuscript reports on the cavity driven emerging of electron-hole collective resonances from the continuum in a double gated bilayer graphene at terahertz frequency. This article inserts in the well-studied, theoretically and experimentally, research line of ultrastrong-light matter interaction. By electrostatic tuning the band gap of the bilayer graphene across the THz resonance supported by an inverse metallic bow-tie, a vacuum Rabi frequency splitting > 40% was recorded. B) (Originality and significance) There is an extensive literature produced in more than 2 decades on the ultrastrong light-matter interaction. However, the authors provided here sufficient elements of novelty (even though a similar concept is presented in ref [63]), namely the double gate architecture, which allows a robust control over the optical response of the device, and the collective interaction promoted by the cavity, binding electron-hole pairs (although partly addressed in Ref [28], and theoretically predicted in [45]). C) (Data&Methodology), H) (Clarity&context) The methodology, quality of the presentation and approach are adequate and valid. The theoretical framework describing the experiments, even though exhaustively studied in literature, is robust and supported the hypothesis. The article is clear and well-written. G) References: are generally adequate. E) (Conclusions) I tend to agree with the authors about the potential of this approach for future applications, even though these are only summarily sketched. Where the authors would like to go from here?

Further points to be addressed, are reported below.

•The device architecture consists of a double gate structure. It is fully understandable the few layer graphite sheet acting as bottom gate (why the SiO2 layer between BG and HR Si?) but I wonder if the authors could directly bias the metallic inverse bow-tie using it as top gate instead of adding a few layer graphite top gate(?).
•Page 6, line 17-18: “This result…” I do not see why the THz signal is insensitive to the graphite gates. Do the authors have an estimation of the charge mobility? Normally, THz resonances are very sensitive to the absorption of monolayer graphene placed in close proximity. If the mode is able to efficiently interacts with the bilayer graphene, it should interact even more (because of a larger superposition with the optical mode supported by the cavity) with the graphite top gate. Probably this has been partly ruled out by the normalization (does it take into account also the few layer graphite gates?). I do not see how efficient gating can be achieved with a “…reduced charge mobility and consequent suppression of the Drude-like response…” with these gates.
•CNP: the authors identify this as the locus of n=0 (with n=carriers’ concentration). Are there any residual charges? In figure 3B are reported the spectra cut lines from Figure A: what are the 0.26x10^12 cm-2 and the 2.37x10^12 cm-2 referring to? Is this the available range of carriers’ concentration tuning?
•Page 7, line 15 and 22. The authors refer to the carriers’ “screening”. Is this rather absorption of the free carriers following Drude model?
•Spectroscopy: the spectral resolution is limited, I am wondering how reproducible is the approx 1% difference in Figure 2 C (inset) or Supp. Fig 2, after a full warm up of the system and realignment.
•Temperature range: why the authors acquired measurements only up to 50K? The splitting is still clearly visible. It would have been meaningful to observe and report the whole temperature curves, as in Y. Todorov et al “Ultrastrong Light-Matter Coupling Regime with Polariton Dots”, Phys Rev. Lett 105, 196402 (2010). This might have provided further insights into the strength of the excitonic features and their coherence at higher temperatures. I feel that this set of measurements is incomplete.

Referee #2

(Remarks to the Author)
Helmrich et al. performed terahertz (THz) spectroscopy on doubly gated bilayer graphene using a bowtie-shaped THz resonator with broadband transmission. Through transport measurements, they confirmed that applying a displacement field caused a gap to develop at the charge neutrality point (CNP) in the BLG. By comparing THz far-field transmission with D = 0 and finite D, they found that the relative THz transmission is modulated by the carrier density near the CNP. At the CNP, THz transmission exhibited the signature of the coupling between the cavity plasmon mode and the continuum of electron-hole transitions in BLG. They demonstrate the appearance of lower and upper polaritons as the band gap energy approaches the cavity energy by tuning the displacement field. Theoretical computations reveal that the lower polariton forms due to strong electron-hole correlations with the cavity energy, suggesting a cavity-induced bound state of electron-hole pairs. This work clearly demonstrates strong light-matter coupling in the THz regime. Despite the fact that plasmons in BLG cannot be treated as two-level systems and the THz resonator made of gold is not necessarily a high-finesse cavity, demonstrating strong coupling between the cavity mode and BLG carriers is rather surprising. However, due to the following technical issues, I cannot support the publication of this manuscript in Nature.

1. The authors chose a delay time that corresponds to the maximum of the THz field. What justifies this choice? How does relative THz transmission (the comparison between D = 0 and D > 0) change as the delay time changes? Alternatively, how does the delay time that exhibits the maximum THz fields change with the gate voltages?

2. In Fig. 2B, the data is shown as normalized relative THz transmission. The authors should consider presenting non-normalized and normalized signals together.

3. The experimental data in Figures 3A and 3B have sparse data points along the frequency axis. The data resolution is ~0.2 THz. What is the reason for the lower frequency resolution? Could you display the data points alongside the lines connecting them in Fig. 3B?

4. The authors drew simple curves and lines in Figures 4A and 4B, but I do not know how they created these lines from their data points. Those curves and lines are simply drawn as guides for the eyes rather than from quantitative curve fittings.

5. Nevertheless, the authors quantitatively extract the cavity resonance frequency of 2.3 THz and, more importantly, the vacuum Rabi frequency of 1 THz from these data. The authors should detail their experimental analysis and explain how they arrived at these values, providing proper error estimation. The authors state that the cavity coupling coefficient is 43%, claiming that they have at least two digits of significance. Since this is the most important quantitative claim the authors are making, they need to provide further quantitative justification.

6. The theoretical model presented in Fig. 5 needs a clearer description in the text. For example, the authors discuss the Hopefield coefficient and the electron-hole correlation function without providing a proper introduction. While these quantities are discussed in the supplementary materials, the main text does not reference them or the relevant citations.

7. The authors claim that cavity-induced electron-hole pairing is purely based on the theoretical model, but only the temperature-dependent effect shown in Fig. 6 can indirectly support this claim. While this is an exciting claim, I feel that the authors should also discuss other possible scenarios that could explain the experimental observations. Even better would be if they could present some controlled experiments, such as modifying the cavity coupling by changing the distance between the BLG and the THz cavity, or using a cavity of a different size, to demonstrate that electron-hole interactions are indeed greatly modified in the presence of strong coupling.

8. It seems that the authors performed the experiment on a single device. I wonder what the effect of disorder in the sample would be. At least repeating the experiment on one more device would help to ensure that the effect is not related to point 7 above.

Referee #3

(Remarks to the Author)
In this work the authors claim to demonstrate the emergence of photon mediated attractive interaction in bilayer graphene integrated with a THz cavity. This is demonstrated through the emergence of a bound state like spectral feature from the lower polariton formation. The basis of this claim is the fact that they are coupling the resonance of the THz resonator with continuum of electronic states in BLG whose bandgap can be tuned via gating. The authors developed a theoretical model that accounts for the ultrastrong coupling and the emergence of the LP state where the role of photon mediated interaction is found to be the main effect. They further go on to carry out temperature dependent measurements where the estimated Rabi splitting is found to stay constant and this is once again attributed to the photon mediated interaction which should be insensitive to temperature. Worth noting is the ability of the experimental technique to measure directly the bandgap of BLG under field tuning. While the idea of photon mediated attractive interaction arising from vacuum fluctuations in a cavity to bind charged particles is very intriguing, the experimental results reported in this manuscript does not show that. Below I discuss the main concerns:
• Firstly, the present experiments uses a THz probe to observe the polariton states. This is unlike the ones reported in Refs 23-25 from the Faist group where there is no external electromagnetic radiation being input. The experiments reported here

are similar to standard white light reflectivity or transmission experiments that have been reported since the first work of Weisbuch (1992). So what is new here? I am not questioning if polariton states are the new eigen states in the presence of cavity. But the results shown here does not in any way demonstrate the role of vacuum fluctuations.

• Emergence of polariton states when coupling of a continuum of electronic states to a cavity photon is used as the evidence for the formation of a bound state. Isnt't Fano resonances that have been studied extensively an example of a narrow spectral feature arising from the coupling between a discrete state with a continuum? Why is the feature observed not attributed to the usual Fano resonance?

• A bigger concern in this context is the following:

In Fig. 3, the authors investigate the effect of cavity frequency on terahertz (THz) transmission at finite charge density ($n \neq 0$) and zero displacement field ($D=0$). In both experiments (Figs. 3A, B) and simulations (Fig. 3C), the cavity manifests as a dip in transmission, consistent with screening of the cavity mode by injected carriers, as the authors correctly noted. In contrast, a bandgap in BLG at finite D and $n=0$ appears as a shoulder of a broad peak in THz transmission (Fig. 4A). Notably, the cavity dip is narrower than the absorption peak, which is expected since the latter arises from continuum transitions, not a resonance like excitonic transition.

When the gap energy and cavity frequency coincide, it is not surprising that a narrow dip superimposed on a broad peak yields a two-peak structure. However, the authors interpret this as the emergence of upper and lower polaritons, which is questionable. It is unclear why dressing the cavity dip with a continuum absorption would convert it into a peak.

Even in the context of exciton-polaritons especially in plasmonic systems, similar two-peak structures—arising from the superposition of a narrow spectral feature (here from cavity) and broad absorption—have led to overinterpretation. The authors should clarify why this is not the case.

• Isnt't the carrier induced mode screening just the fact that with increasing doping the transmission decreases because of loss. It is surprising that the authors do not see a change in linewidth.

• The interpretation of two peaks as resulting from light-matter coupling is further complicated by the observation of similar features at $D = 0$, where BLG should exhibit no bandgap at all.

One can understand this as the overlap of a cavity-induced dip with a weak absorption peak at $D = 0$. Even without a gap, the density of states is insufficient to screen THz transmission, producing a transmission peak. Meanwhile, the cavity resonance can still induce a sharp dip, even at low carrier density. Could the authors clarify why polariton formation should occur in the absence of a bandgap?

• With increasing temperature, thermally activated carriers can screen THz transmission, thus reducing the broad absorption feature. This can explain the temperature dependence observed. However, the authors instead invoke increased non-radiative decay at elevated temperatures. Since the transitions involve a continuum with broad momentum spread and no sharp resonance, it is unclear why non-radiative decay should play a major role.

Even if a reorganization into exciton-like states occurs, the resulting transitions would remain broad, since electron-hole pairs for all momenta across broad energy range are involved and bound by cavity photon-mediated interaction (unless this is a resonant effect).

• Experimental data in Fig. 4a and 4b are not convincing - especially the claim about upper polariton. In fact 4a with its asymmetric lineshape looks very much like the formation of a Fano resonance.

• Authors claim that the cavity-driven pairing "reproduces the main experimental spectral features (Fig 4C)". However, notable discrepancy is observed between Fig. 4C and 5B. In Fig. 4C the frequency for UP decreases with decreasing bandgap. In contrast, frequency for UP increases with decreasing bandgap in Fig. 5B. Could the authors comment on this inconsistency?

• The direct measurement of the bandgap of BLG under field tuning is a nice result. Overall, I also found the experimental technique to be very nice. However, the present work is not about these two aspects.

Some minor comments:

- Will be good to see the spectrum of the bare cavity. Since the cavity-induced dip in Fig. 3 is more prominent at higher n, could the authors perform experiments similar to Fig. 4 but at finite n.
- Could the authors include the line cut at $n = 0$ in Fig. 3B? Additionally, could they extend the x-axis to 6 THz, at least in the Supplementary Information. I wonder whether the data show two peaks near the cavity dip, as it seems so by from the map in Fig. 3A around $n = 0.25$?

In summary, while the idea of the paper is intriguing, the experimental results fail to support the claims and hence I do not recommend publication of this work in Nature. As a new THz spectroscopy technique, it will fit well in a discipline specific journal.

Referee #4

(Remarks to the Author)

I co-reviewed this manuscript with one of the reviewers who provided the listed reports.

Version 1:

Reviewer comments:

Referee #1

(Remarks to the Author)
In this revised version the authors answered all the queries raised in my previous review.

Specifically:

-C) Data and methodologies. Furhter Temperature measurements have been performed (more T points below and above 100 K might have helped, but it is a minor issue).The effect of gates on the measurements (mostly clarifications, though I wonder the 4 nm Cr uniformity and effectiveness of the second sample) have been exhaustively addressed.

D) Statistics.The reproducibility of the measurements from day to day (e.g. Fig R3) and on a different sample support the validity of their approach.

-B) and H) Originiality and context. The authors also modified the manuscript to better promote their approach and findings in a wider range of future applications and theoretical scenarios.

Overall, it is a nice physics and an interesting experimental configuration, appealing to a broad audience and I am therefore happy to support publication.

Referee #2

(Remarks to the Author)
The authors have successfully addressed the concerns I raised in my previous submission in a thorough and technically convincing manner. The additional data from the second device, the supplementary analyses and simulations, and the clarifications have significantly strengthened the manuscript. I therefore recommend accepting this manuscript following the minor revisions aimed at improving clarity and presentation outlined below.

1. Although the authors have softened the language surrounding 'vacuum fluctuations' and emphasized 'photon-mediated interactions', it would be helpful to explicitly reiterate in the main text conclusion (not only in the supplementary information) that the interpretation relies on linear-response spectroscopy and a quantum model rather than a direct probe of ground-state pairing.

2. A short intuitive sentence contrasting continuum reshaping with conventional exciton–polaritons could also help non-experts to appreciate why this system is unusual.

3. The explanation for the persistence of the upper polariton as the gap closes is now correct and well-motivated from a physical point of view (e.g. the Hopfield weight becoming photonic and the analogy to Landau polaritons). To help non-specialist readers avoid misinterpretation, the authors may wish to consider stating something along the following lines: 'The persistence of the UP at D = 0 does not imply survival of the bound state but rather reflects the cavity-like branch in the ultrastrong-coupling continuation.'

Referee #3

(Remarks to the Author)
The authors have now revised the manuscript to address the concerns raised by the reviewers. However, the revised version still fails to convince the reader about the validity of the experimental results. Below I address the main concerns:
1. To start off Device 2 data in figure R12 makes the case even weaker! I do not understand what the authors mean when they say: " Despite these disadvantages, the referenced spectra of D2 (Fig. R12) reproduce the key observations of D1: at n = 0 and finite D, we resolve the emergence of two branches and a zero-detuning gap consistent with the coupled-oscillator dispersion". It is unclear what is observed here that is similar to that of Device !. How does one even extract the Rabi splitting from the data of Device 2?

2. In linecut shown in R16 seems to be taken from the color map of R15. However it is unclear where the upper polariton is in the color map at -0.08 V/nm.

3. The authors continue to claim that they observe a dispersive upper polariton branch. However, the experimental data does not show that! As we show below how one can easily get the two features:
We have illustrated in the attachment how a transmission dip arising from the cavity mode (as seen in the n=0, D=0 case), combined with a broad peak in transmission (as seen in the n=0, D=-0.32V/nm case), can trivially produce two peaks. Just adding them gives the two peak feature!

In their response, the authors argue that the regime in Fig. 4 is distinct from that in Fig. 3 and that there is no expected transmission dip in the Fig. 4 case (charge-neutral), since it occurs at n=0 and there is no charge injection. However, even in Fig. 3 for the case of n=0, D=0 (where BLG should be a semimetal), the data clearly show a decrease in transmission coincident with the cavity resonance.
Secondly, if one accepts that the cavity mode is not visible in the gapped case, it is then unclear how two peaks emerge. Even if it comes from the redistributed oscillator strength of the matter mode, the matter mode manifests only as a shoulder (rather than a peak). If the bare cavity mode is not optically visible, it is not evident why the resulting hybrid modes should appear as two peaks rather than two dips, or a dip plus a distortion of the broad background. This point remains insufficiently addressed.
More significantly, the experimental signatures do not demonstrate anticrossing. The peak positions in Fig. 4B do not consistently follow the black guide line for the purported upper polariton. In fact, a plot of the extracted/quantified positions of two peaks for different D from the experimental data is also missing.
4. A related concern in Comment 6 also remains unresolved. I argued that the observed temperature dependence could arise from thermally activated carriers screening the THz response. The authors state that referencing each trace in Fig. 6 to an in situ measurement taken at constant D and the same temperature removes temperature-induced effects. This argument is incorrect: the density of thermally activated carriers depends not only on temperature but also on the bandgap, which is being tuned by the displacement field. Referencing to a fixed-D trace therefore cannot eliminate gap-dependent thermal activation effects. The authors' response does not address this physical point.
5. Minor comment: It will be helpful if the authors stuck to using frequency in X axis (Fig. 4b versus 5b).
In summary, the theoretical idea is interesting, however, the experimental results fail to support the claims even in the revised version. It appears that there is a simpler explanation for the observations reported. The second device data makes it even weaker! And hence I do not recommend publication of this work in Nature.

Referee #4

(Remarks to the Author)
I co-reviewed this manuscript with one of the reviewers who provided the listed reports.

Open Access This Peer Review File is licensed under a Creative Commons Attribution 4.0 International License, which permits use, sharing, adaptation, distribution and reproduction in any medium or format, as long as you give appropriate credit to the original author(s) and the source, provide a link to the Creative Commons license, and indicate if changes were made.
In cases where reviewers are anonymous, credit should be given to 'Anonymous Referee' and the source.
The images or other third party material in this Peer Review File are included in the article's Creative Commons license, unless indicated otherwise in a credit line to the material. If material is not included in the article's Creative Commons license and your intended use is not permitted by statutory regulation or exceeds the permitted use, you will need to obtain permission directly from the copyright holder.

To view a copy of this license, visit https://creativecommons.org/licenses/by/4.0/

Referee response:

Cavity-Driven Attractive Interactions in Quantum Materials

F. Helmrich[1], H. S. Adlong[1,2], I. Khanonkin[1], M. Kroner[1], G. Scalari[1], J. Faist[1], A. İmamoğlu[1], and T. F. Nova[1]

[1]Institute for Quantum Electronics, ETH Zürich, Auguste-Piccard-Hof 1, 8093 Zürich, Switzerland

[2]Institute of Theoretical Physics, ETH Zürich, Wolfgang-Pauli-Strasse 27, 8093 Zürich, Switzerland

Legend:

(i) Maroon: Reviewer comments,

(ii) *Italic: manuscript extracts and **changes***,

(iii) Black: Answers to comments.

Referee 1 (Remarks to the Author):

I reviewed the manuscript "Cavity-Driven Attractive interactions in Quantum Materials" by Helmrich et al.

A) (Summary) The manuscript reports on the cavity driven emerging of electron-hole collective resonances from the continuum in a double gated bilayer graphene at terahertz frequency. This article inserts in the well-studied, theoretically and experimentally, research line of ultrastrong-light matter interaction. By electrostatic tuning the band gap of the bilayer graphene across the THz resonance supported by an inverse metallic bow-tie, a vacuum Rabi frequency splitting > 40 % was recorded.

B) (Originality and significance) There is an extensive literature produced in more than 2 decades on the ultrastrong light-matter interaction. However, the authors provided here sufficient elements of novelty (even though a similar concept is presented in ref [63]), namely the double gate architecture, which allows a robust control over the optical response of the device, and the collective interaction promoted by the cavity, binding electron-hole pairs (although

partly addressed in Ref [28], and theoretically predicted in [45]).

We thank the referee for recognizing the novelty of our study. We would like to highlight that while Kipp et al. present a related concept, our device employs two independently addressable graphite gates that enable decoupled control of both carrier density and a static out-of-plane field, which we combine with broadband THz cavity spectroscopy. This capability is essential for the reported observations and, to our knowledge, has not been previously demonstrated.

C) (Data an Methodology) The methodology, quality of the presentation and approach are adequate and valid. The theoretical framework describing the experiments, even though exhaustively studied in literature, is robust and supported the hypothesis.

H) (Clarity and context) The article is clear and well-written.

G) References: are generally adequate.

We thank the referee for the positive comments regarding the robustness of our experimental and theoretical methodology (Point C), the clarity and overall presentation of the manuscript (Point H), and the adequacy of the cited literature (Point G).

E) (Conclusions) I tend to agree with the authors about the potential of this approach for future applications, even though these are only summarily sketched. Where the authors would like to go from here?

We revised the Conclusions to (i) clarify the "spectroscopy-only" use of our architecture, (ii) highlight the electrostatic tunability unique to 2D vdW systems and how it enables controlled studies of cavity-induced effects, (iii) emphasize how cavity coupling can modify correlated phases—including targeted tests of predicted superconductivity enhancement—and (iv) outline feasible probes of superradiant behavior.

We have demonstrated that THz cavities serve as powerful tools for generating attractive interactions in quantum materials. To validate this phenomenon, we developed an experimental platform specifically designed for the far-infrared spectroscopy of ***micro-structured vdW*** *heterostructures. Our measurements reveal two distinct regimes: when the cavity is off-resonance, we probe the intrinsic THz bandgap*

of BLG, providing an unparalleled window into the material's optical properties; conversely, tuning the system into resonance triggers ***light-matter*** *hybridization—a hallmark of ultrastrong coupling—where the spectroscopic signature reflects the coupled light–matter system, thereby enabling the observation of cavity-induced bound states.*

For applications that require spectroscopy without cavity hybridization, the same architecture can operate in a "spectroscopy-only" mode by increasing the spacer between the resonator and the vdW stack (from the present ~ 140 ***nm to several microns) [1], thereby reducing the light–matter coupling while preserving sub-wavelength focusing and spatial resolution.*** *In this context, our platform addresses the limitations of conventional THz spectroscopic techniques for micro-structured devices, which often suffer from bandwidth constraints or incompatibility with dual gating. While on-chip terahertz techniques have proven highly effective in probing the ultra-low energy electrodynamics of 2D materials [2–7], they face inherent bandwidth limitations [8], typically not exceeding 1.5 THz or 6 meV, as well as a lack of* ***independent tunability of charge density and displacement field****. Notably, much of the physics of quantum [9] and* ***vdW*** *materials [10] is expected or has been observed at energies of several to tens of meV. This energy range encompasses collective modes such as phonons [11], Mott gaps and electronic bands in Moiré systems [12], binding energies [13] as well as Higgs and Bardasis-Schrieffer modes in excitonic insulators [14, 15], and gaps and excitations of Wigner crystals [16], among others. With a bandwidth exceeding 20 meV, our technique outperforms existing methods and bridges the gap between on-chip terahertz and photoconductivity measurements [12, 17–19] while providing access to* ***robust in-situ referencing****, time and spatial resolution ($\ll$ wavelength) as well as the full material optical properties through amplitude and phase measurements of the transmitted field. In addition, our method offers a significant advantage over conventional THz scanning-probe techniques, which, despite achieving spatial resolutions of tens to hundreds of nanometers, struggle with full dual gating and require extensive calibration and a detailed understanding of the probe-sample interaction to extract reliable spectroscopic information [20]. Lastly, our approach is versatile, being adaptable to various generation techniques and cavity designs. This flexibility makes it scalable across a wide range of frequency domains, from the terahertz to the mid-infrared.*

Beyond spectroscopy, our platform enables cavity–QED studies in vdW materials with a capability unique to 2D systems: tunability. To isolate or control cavity-induced effects one may vary the cavity resonance conditions [[21]] or the material phase [[22, 23]]. Thanks to electrostatic control, 2D systems allow us to do both: first by bringing excitations on and off resonance and second by driving the material into and out of a given phase-mapping how USC

depends on and affects the underlying order. In practice, independent n and D control allow to tune the strength and nature of the excitation spectrum, enabling us to monitor cavity effects within phases and across phase transitions—an experimental lever unavailable in traditional USC platforms.

Our observation of photon-mediated bound states in 2D materials raises a broader question: can cavity-driven pairing extend to ground-state collective phenomena in condensed matter? Theory predicts that cavity coupling can reshape superconducting instabilities and critical temperatures [24–27]. Our architecture provides a natural testbed enabling unprecedented single-device sweeps across entire phase diagrams and superconducting domes to target vacuum-renormalization effects. Gate-defined superconductors in moiré graphene—including magic-angle twisted bilayer graphene [28]—and field-tuned phases in Bernal BLG [29] provide continuously tunable platforms in which to interrogate, and potentially enhance, superconducting phase transitions via tailored light–matter coupling, paving the way to engineered correlated phases under cavity control.

Looking ahead, integrating our technique with dilution refrigerators capable of reaching millikelvin base temperatures represents the next frontier. By delivering low-power, low-photon-energy THz radiation directly to the sample, our method circumvents the heating effects typically encountered with optical laser pulses in on-chip THz setups. This advance will enable broadband far-infrared spectroscopy and cavity-control of 2D materials under ultra-low-temperature conditions, thereby opening the door to investigation of delicate quantum phenomena and their collective modes— from superconducting gaps [28, 29] ***and Wigner crystal excitations [16] to fractional excitons in fractional Chern insulators [30, 31].***

Lastly, our findings raise fundamental questions about the cavity-controlled ground state. The appearance of excitonic features at finite bandgaps prompts us to ask whether the cavity-induced bound state survives once the gap fully closes. ***In this context, the highly localized, non-uniform cavity fields used here provide a powerful testbed for rigorously exploring cavity-QED phenomena and scrutinizing predictions of superradiant phase transitions [32–39]. While uniform-field no-go theorems constrain equilibrium superradiance, they do not apply when the cavity field is strongly spatially inhomogeneous([36]), as in our sub-wavelength THz meta-atom. In this regime, an equilibrium transition would be signaled by a gate-tunable softening of the lower polariton toward zero frequency together with spontaneous breaking of a discrete parity symmetry—namely, an intracavity displacement at $D = 0$. Future experiments that directly probe ground-state symmetry***

breaking—such as second harmonic generation—could reveal new ordered phases—such as cavity-driven excitonic insulators [13].

By combining strong sub-wavelength confinement with gate-tunable van der Waals heterostructures, our approach opens an exciting avenue for engineering many-body phenomena via electromagnetic vacuum fields and paves the way for deeper investigations into cavity-induced phase transitions and the fundamental nature of light–matter interactions in quantum materials.

Further points to be addressed, are reported below.

Comment 1 - The device architecture consists of a double gate structure. It is fully understandable the few layer graphite sheet acting as bottom gate (why the SiO2 layer between BG and HR Si?) but I wonder if the authors could directly bias the metallic inverse bow-tie using it as top gate instead of adding a few layer graphite top gate(?).

We address the two points separately:

1. *Role of the SiO_2 layer on high-resistivity Si.* The SiO_2 layer between the graphite back gate and and the Si substrate has no primary function in the transport and THz experiments presented in the article. However, a layer of thermal oxide (usually 285 nm) on top of a Si substrate is commonly used in the fabrication process of van der Waals heterostructures to enhance the optical contrast of thin flakes such as mono- and bilayer graphene for easier identification.

2. *Why not use the resonator plane as a top gate?* First, biasing the patterned metal that contains a bow-tie aperture would generate strong fringe fields and a highly non-uniform out-of-plane displacement field across the active region. Our resonator is intentionally *electrically insulated* from the heterostructure by an ALD AlO_x spacer so that the cavity boundary conditions are decoupled from DC gating.
   Second, biasing the resonator plane would create a charged plate capacitor with a large diameter of 4 mm. This size is determined by the clear aperture of the top immersion lens since we require the gold screen to block all incoming THz radiation. By biasing such a large resonator plane, we would thus create an electrically unstable device more susceptible to breakthroughs, short circuits and parasitic leakage paths. We added an

additional micrograph to the supplementary material to highlight the size of the gold resonator plane required to block uncoupled THz radiation.

Comment 2 - Page 6, line 17-18: "This result..." I do not see why the THz signal is insensitive to the graphite gates. Do the authors have an estimation of the charge mobility? Normally, THz resonances are very sensitive to the absorption of monolayer graphene placed in close proximity. If the mode is able to efficiently interacts with the bilayer graphene, it should interact even more (because of a larger superposition with the optical mode supported by the cavity) with the graphite top gate. Probably this has been partly ruled out by the normalization (does it take into account also the few layer graphite gates?). I do not see how efficient gating can be achieved with a "...reduced charge mobility and consequent suppression of the Drude-like response..." with these gates.

We have clarified both the referencing procedure and the role of the graphite gates and revised the manuscript text to avoid ambiguity.
All frequency-resolved THz spectra (Figs.3,4,6) are referenced to an *insulating* BLG configuration at finite D and $n = 0$, which removes baseline absorption from the optics, resonator, substrate, spacing layers *and the graphite gates*. Note that even though BLG is in an insulating phase, the gates are highly doped. Thus, since the reference trace contains the response of the same electrodes, their contribution cancels in the ratio.
However, Fig. 2B, is shown *without* division by a reference: it displays the lock-in THz field at a fixed delay (we normalize only to the trace's own minimum/maximum, and we now provide the unnormalized data in the Supplementary for completeness). Even so, the map reproduces the transport charge-neutrality line (Fig. 2A) without appreciable voltage-dependent backgrounds, showing that the THz signal tracks the BLG state while remaining insensitive to the gates prior to referencing.
Physically, few-layer graphite films that are not encapsulated exhibit low mobility; in a Drude picture this shortens the scattering time τ and spreads $\sigma_1(\omega)$ yielding a small, broad loss that is largely removed by referencing. Efficient DC gating of BLG is unaffected, as it relies on slow charge flow (compared to THz frequencies) and the large hBN gate capacitance. The use of lower-mobility or even resistive gate materials for THz-compatible devices is standard practice (e.g., ZnO gates in on-chip THz graphene experiments [6]).
As an additional control, we have fabricated a second device (D2) where we replaced both

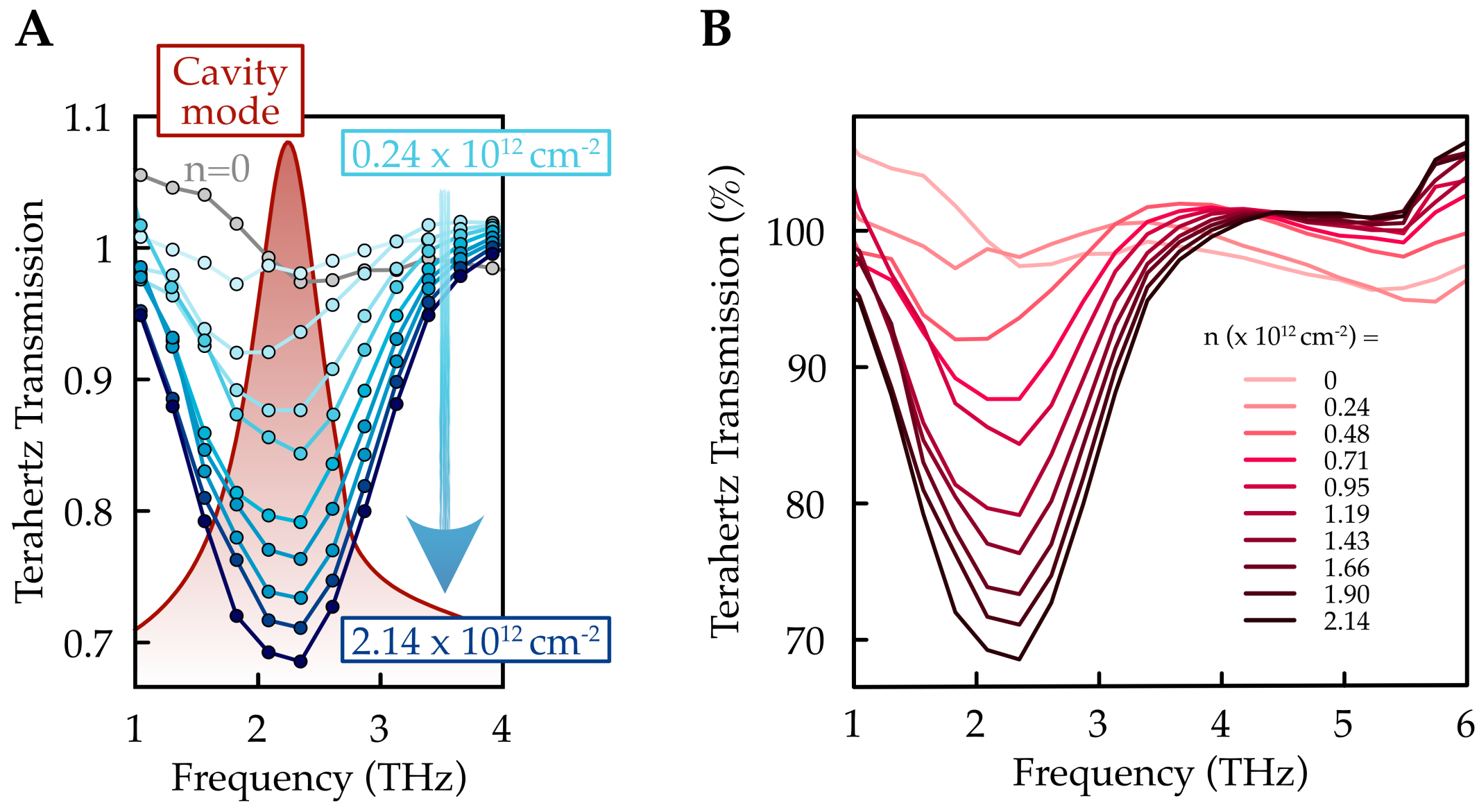

Figure R1: **A**. Doping dependence of referenced transmission. Equivalent to Figure 3B. **B** Doping dependence of referenced transmission; maximum bandwidth 1-6 THz

graphite gates with ultrathin metallic gates (4 nm Cr): the absolute transmission decreased by roughly a factor of two due to higher conductivity and larger material thickness, yet the *referenced* spectra and all trends were unchanged. This confirms that our conclusions are insensitive to the specific gate material and that gate absorption acts only as a featureless background; the corresponding data and device details are now included in the Supplementary Material.

Comment 3 - CNP: the authors identify this as the locus of n=0 (with n=carriers' concentration). Are there any residual charges? In figure 3B are reported the spectra cut lines from Figure A: what are the 0.26×10^{12} cm^{-2} and the 2.37×10^{12} cm^{-2} referring to? Is this the available range of carriers' concentration tuning?

All the doping dependent data for the full terahertz bandwidth (1 to 6 THz) and accessible doping ranges (from -0.48 to 2.12×10^{12} cm^{-2}) are already shown in Fig. 3A. Figure 3B is meant to draw attention to the main spectral feature that is the carrier-driven suppression of the cavity mode for a few representative dopings. For completeness, in the revised manuscript we add the $n = 0$ trace to Fig. 3B, and provide complete 1–6 THz line cuts in the Supplementary Material (Fig. R1 here. Note also that we have corrected for a slight calibration error in the density axis of this Figure (less than 10%; e.g., 0.26×10^{12} cm^{-2} $\rightarrow$ 0.24×10^{12} cm^{-2}).

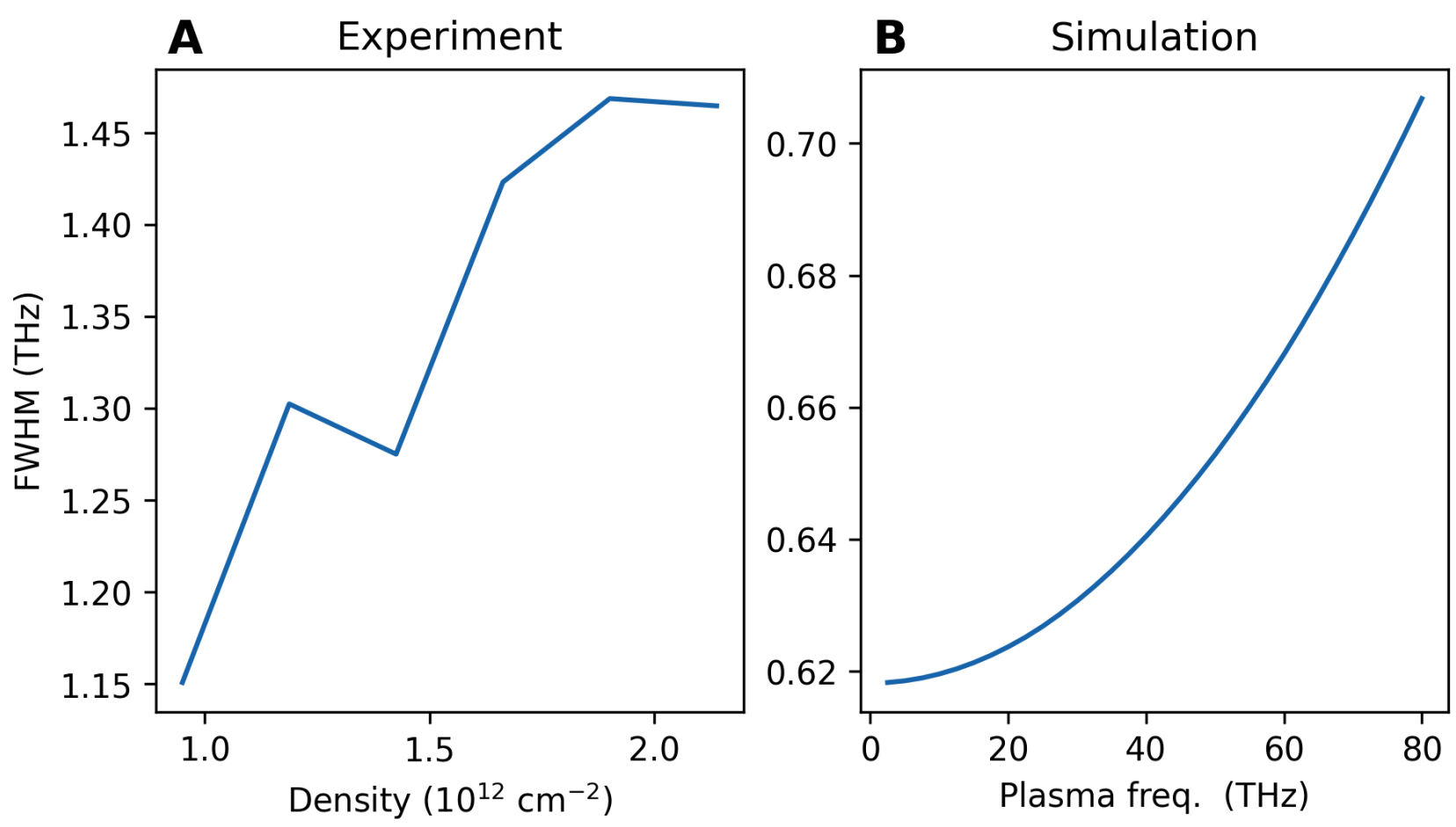

Figure R2: Linewidth as function of doping. **A** FWHM of the spectral dip in the density-dependent measurement of Fig. 3A-B. **B** FWHM of the simulated spectra in Fig. 3C as a function of the plasma frequency ω_p.

Comment 4 - Page 7, line 15 and 22. The authors refer to the carriers' "screening". Is this rather absorption of the free carriers following Drude model?

We thank the referee for the remark. We have replaced "screening" with a more precise description: carrier injection increases both absorption and, via a larger impedance mismatch, the reflectance of the BLG layer, leading to higher dissipation and reduced external field–resonator coupling, thereby lowering transmission. Prompted by this comment, we re-analyzed the line shape in Fig. 3A and confirm a systematic linewidth broadening with increasing doping (Fig. R2A), consistent with our FEM simulations (Fig. R2B).

Comment 5 - Spectroscopy: the spectral resolution is limited, I am wondering how reproducible is the approx 1% difference in Figure 2 C (inset) or Supp. Fig 2, after a full warm up of the system and realignment.

Our measurements were performed in a continuous-flow cryostat, which required multiple partial and full warm-ups and independent optical realignments. The temperature series and gate maps were taken on different days under different alignments, yet all salient features are reproducible and recur in both time and frequency domains—see Fig. R3 for a representative collection of referenced transmission spectra taken at 5 K and adapted from Figs. 4B and 6A. These measurements, taken weeks apart under different alignments and cooldowns, are extremely reproducible for each given displacement field D. The relative difference between any

two curves is $\lesssim 3\%$.

Crucially, for every sweep in D or n we acquire an *in-situ* reference at the insulating configuration ($n = 0$, finite D), and we re-acquire this reference at the start of each averaging block during long scans. Because the reference contains the day's coupling efficiency, beam pointing, and optics, taking the ratio removes alignment-dependent throughput and phase, isolating the BLG response. This interleaved referencing compensates slow drifts over hours to days and enabled week-long acquisitions (e.g., the 50 K dataset) without loss of comparability.

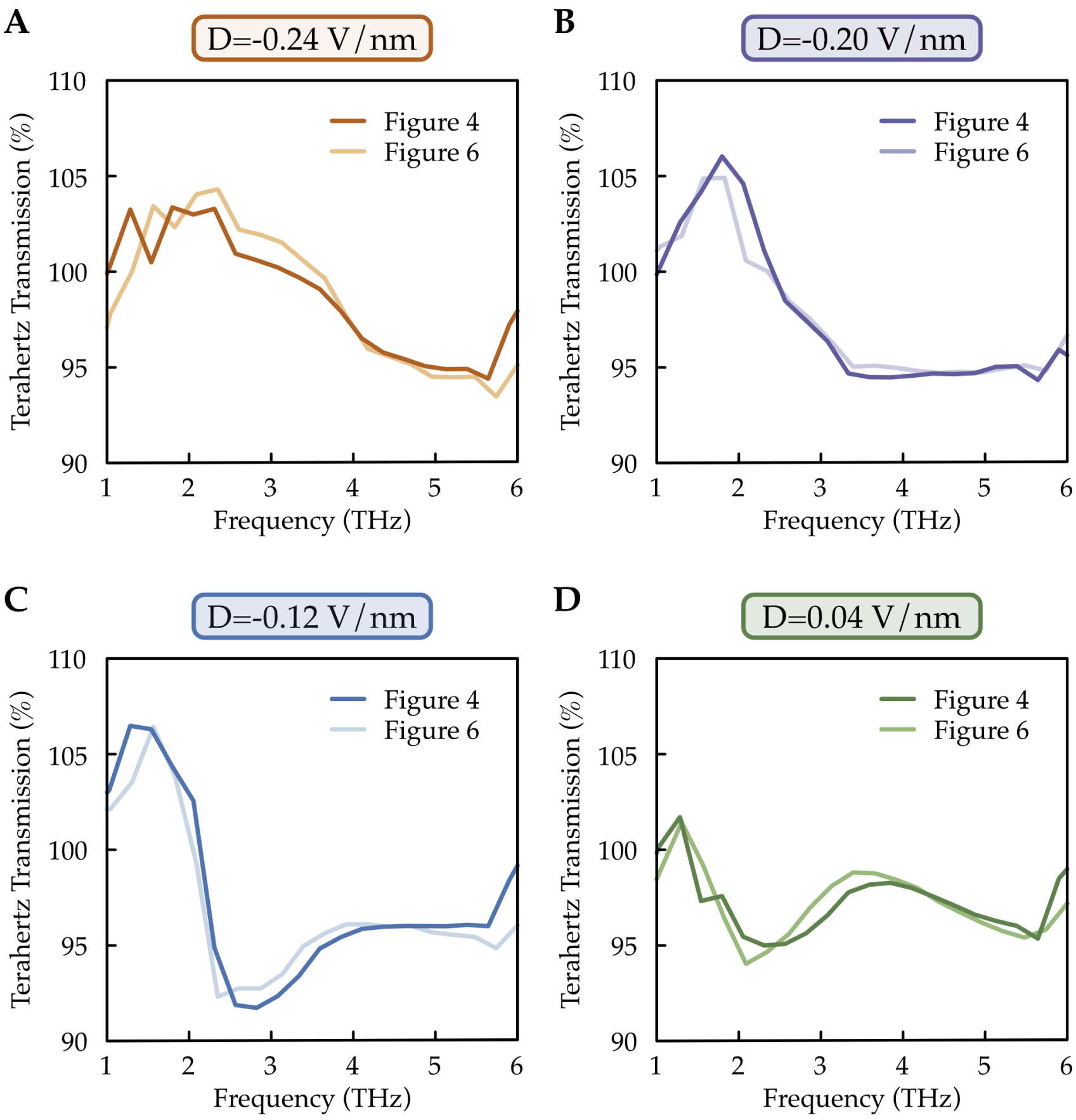

Figure R3: Referenced 5 K transmission for two separate measurements at different D and $n = 0$, adapted from Figs. 4B and 6A. **A** $D = -0.24\,\mathrm{V/nm}$. **B** $D = -0.2\,\mathrm{V/nm}$. **C** $D = -0.12\,\mathrm{V/nm}$. **D** $D = 0.04\,\mathrm{V/nm}$.

Comment 6 - Temperature range: why the authors acquired measurements only up to 50K? The splitting is still clearly visible. It would have been meaningful to observe and report the whole temperature curves, as in Y. Todorov et al "Ultrastrong Light-Matter Coupling Regime with Polariton Dots", Phys Rev. Lett 105, 196402 (2010). This might have provided further insights into the strength of the excitonic features and their coherence at higher temperatures. I feel that this set of measurements is incomplete.

We agree that a broader temperature sweep would be informative. In the revised manuscript, we extend the dataset to $T = 100\,\mathrm{K}$ and $200\,\mathrm{K}$ (Supplementary Material, Fig. R4). As the referee will appreciate, these measurements challenge the limits of what we are able to detect. As in Fig. 6, the spectral contrast decreases with increasing temperature, but the anti-crossing remains discernible at 100 K. At 200 K, noise dominates and the polariton branches are no longer resolvable within our noise floor. It is difficult to unambiguously identify the origin of the lack of polariton visibility at 200 K: Fig. 6 already shows that visibility decreases with temperature, and 200 K likely entails increased dissipation, thermally activated carriers that repopulate the bands, and consequent changes in the effective transition dipole moment. It could also reflect limitations of the measurement technique at higher temperatures. In particular, thermally activated carriers in the high-resistivity Si substrate and immersion lenses produce significant free-carrier absorption along the $\sim 6.7\,\mathrm{mm}$ Si path of the immersion assembly, which lowers throughput at elevated T. Although longer acquisitions are possible at higher T, the opposing trends—smaller BLG features and larger Si absorption—limit the attainable SNR. To mitigate drifts, we interleave *in-situ* references throughout each scan and at the start of every averaging block, enabling multi-day integrations; the 50 K, 100 K, and 200 K measurements were each acquired over several days and remain mutually consistent. We emphasize that the absence of a resolvable splitting at high T does not by itself rule out strong light–matter coupling when linewidths approach the Rabi splitting [40]. The new temperature data and acquisition details are now included in the Supplementary Material.

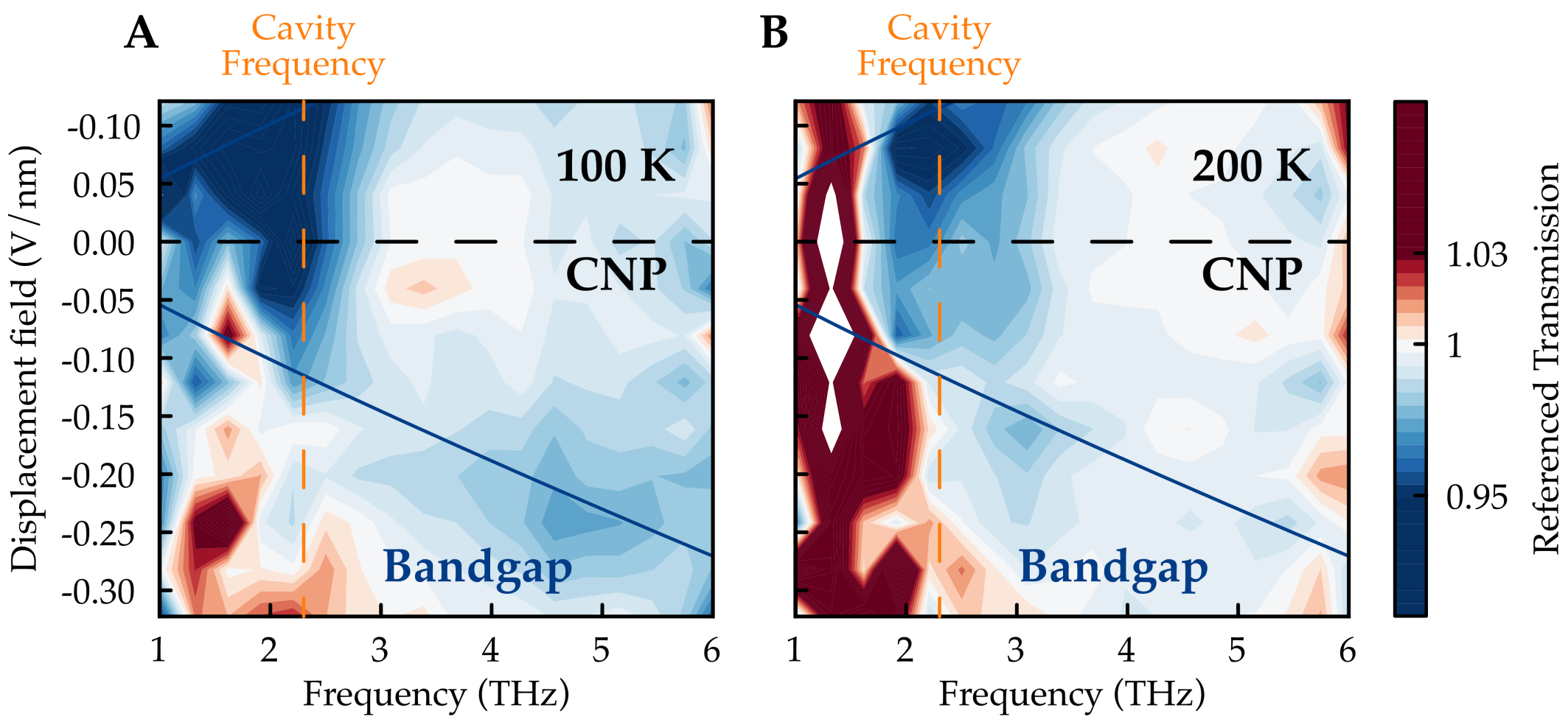

Figure R4: Displacement field-dependent THz transmission spectra at 100 K (**left**) and 200 K (**right**). The voltage parameters are identical to Fig. 6 of the main text. The spectra are referenced to the insulating state at $D = -0.38\,\mathrm{V/nm}$. The data is presented in a differently scaled color map as compared to Fig. 6 to enhance visibility of spectral features.

Referee #2 (Remarks to the Author):

Helmrich et al. performed terahertz (THz) spectroscopy on doubly gated bilayer graphene using a bowtie-shaped THz resonator with broadband transmission. Through transport measurements, they confirmed that applying a displacement field caused a gap to develop at the charge neutrality point (CNP) in the BLG. By comparing THz far-field transmission with D = 0 and finite D, they found that the relative THz transmission is modulated by the carrier density near the CNP. At the CNP, THz transmission exhibited the signature of the coupling between the cavity plasmon mode and the continuum of electron-hole transitions in BLG. They demonstrate the appearance of lower and upper polaritons as the band gap energy approaches the cavity energy by tuning the displacement field. Theoretical computations reveal that the lower polariton forms due to strong electron-hole correlations with the cavity energy, suggesting a cavity-induced bound state of electron-hole pairs.

This work clearly demonstrates strong light-matter coupling in the THz regime. Despite the fact that plasmons in BLG cannot be treated as two-level systems and the THz resonator made of gold is not necessarily a high-finesse cavity, demonstrating strong coupling between the cavity mode and BLG carriers is rather surprising.

We thank the referee for the positive assessment of our evidence for strong light–matter

coupling in a continuum system with a low-Q THz resonator.

We would also like to clarify that neither the cavity nor the observed matter response is plasmonic in nature. At THz frequencies, bulk gold ($\sim$ 200 nm in our devices) behaves as a near-perfect conductor, and our inverse bow-tie forms a sub-wavelength *slot* mode dominated by quasi-electrostatic near fields rather than optical surface plasmons; its response is accurately captured by a quasi-static description.

On the material side, the dominant absorption at $n = 0$ arises from interband transitions across the gate-tunable BLG gap. Upon doping, intraband (Drude) absorption grows and BLG plasmons may exist in principle, but a plasmonic interpretation is inconsistent with our data: the doping-dependent feature we observe is *non-dispersive* within our spectral resolution—it does not shift with carrier density—whereas 2D plasmons at fixed wave vector obey $\omega_{\text{Plasmon}} \propto \sqrt{n}$ [41] and would move markedly with n.

Using the model of Tomadin *et al.* [41], we estimate that plasmon modes commensurate with our lateral dimensions ($\geq 2\,\mu$m) [42] would remain below the cavity frequency and only barely enter our 1–6 THz window over the explored densities (see Fig. R5). These considerations show that plasmons are not the dominant absorption channel in our experiment. If present at all, any plasmonic contribution is overshadowed by the far more salient interband features that dominate the bias-dependent spectra.

We emphasize that the observed THz absorption arises from only two atomic layers: at such sub-nanometer optical thickness, even an ultrathin gold film would be largely transparent at THz frequencies. Resolving clear, gate-tunable features under these conditions underscores the unique capabilities and sensitivity of our technique.

We have added a discussion of the plasmon resonances in the resonator and BLG in the main text as well as a short discussion of the model of Tomadin et al. to the Supplementary Material.

However, due to the following technical issues, I cannot support the publication of this manuscript in Nature.

We strongly believe that the technical concerns raised by the referee can be successfully addressed (see below) and we would appreciate if the referee could reconsider their recommendation on this basis.

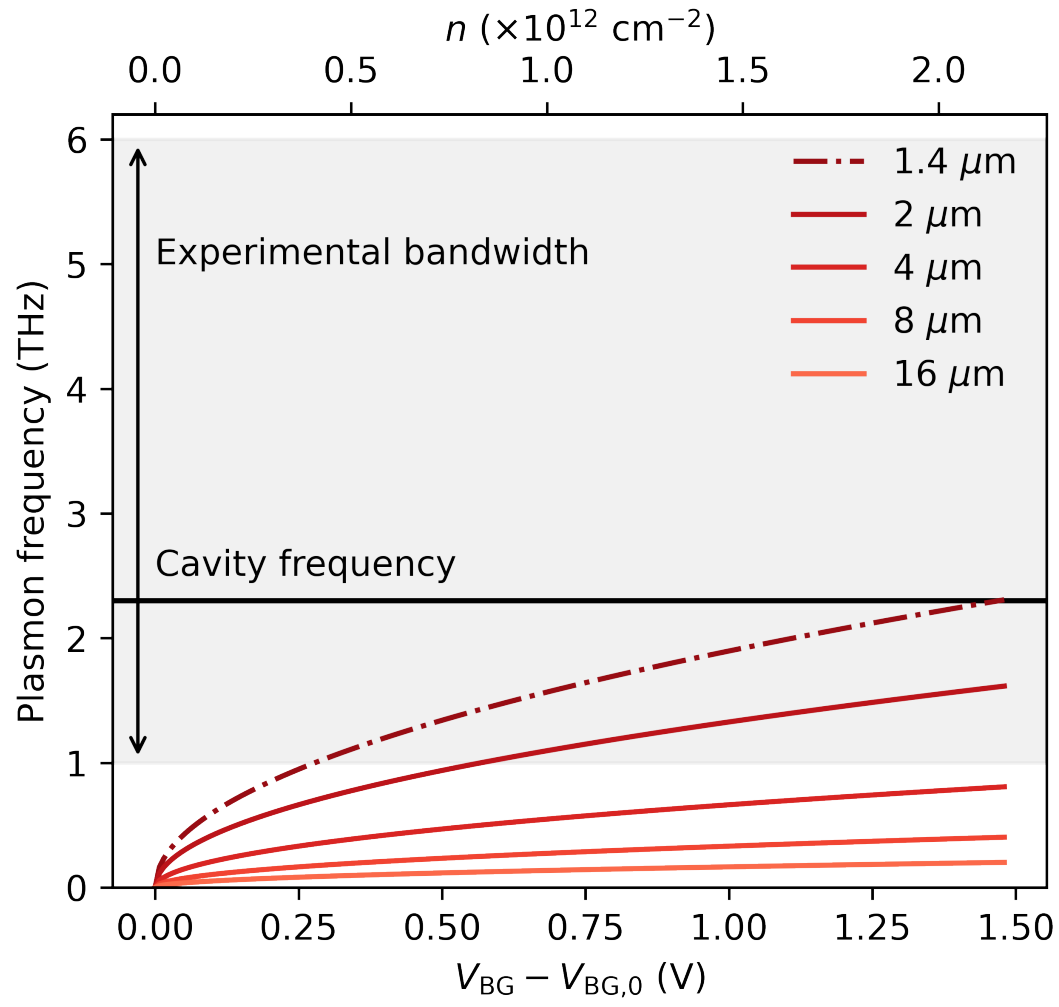

Figure R5: Observability of plasmons. Calculated gate-dependent plasmon dispersion in dual-gated BLG device following Ref. [41] (Eq. (28) of Supp. Material). The wave vector is determined by geometric length scales of the resonator. Note that the smallest geometric feature of the resonator in D1 and D2 is 2 µm. The experimental band width is limited between 1 and 6 THz and the cavity resonance is at 2.3 THz (black line). The back gate voltage axis corresponds to the voltages applied experimentally (while keeping $D = 0$) inducing the electron density n.

Comment 1 - The authors chose a delay time that corresponds to the maximum of the THz field. What justifies this choice? How does relative THz transmission (the comparison between D = 0 and D > 0) change as the delay time changes? Alternatively, how does the delay time that exhibits the maximum THz fields change with the gate voltages?

In time-domain THz spectroscopy a full spectrum at fixed (D, n, T) is obtained by recording the field $E(t)$ over all delays and Fourier transforming it; we do this wherever spectral information is required (Figs. 3, 4, and 6). This approach is extremely time-consuming and severely limits the (D, n, T) values that can be explored in a single measurement.

By contrast, the dense (D, n) map in Fig. 2B was acquired at a *fixed* delay to make the map feasible with high SNR. We deliberately chose the delay at the maximum of the transmitted field because it maximizes signal and is least sensitive to small timing drifts (near the peak, $dE/dt \approx 0$).

A fixed-delay map does not carry any frequency information and should not be over-interpreted spectroscopically: a change at one time point mixes amplitude and phase and cannot by itself distinguish absorption from phase shifts. Accordingly, Fig. 2B is used only to delineate the two gate-defined regimes—insulating versus metallic—already identified by transport, and to choose cut lines for subsequent full spectral scans.

Addressing the referee's question directly, the single–time–delay, un-referenced transmission

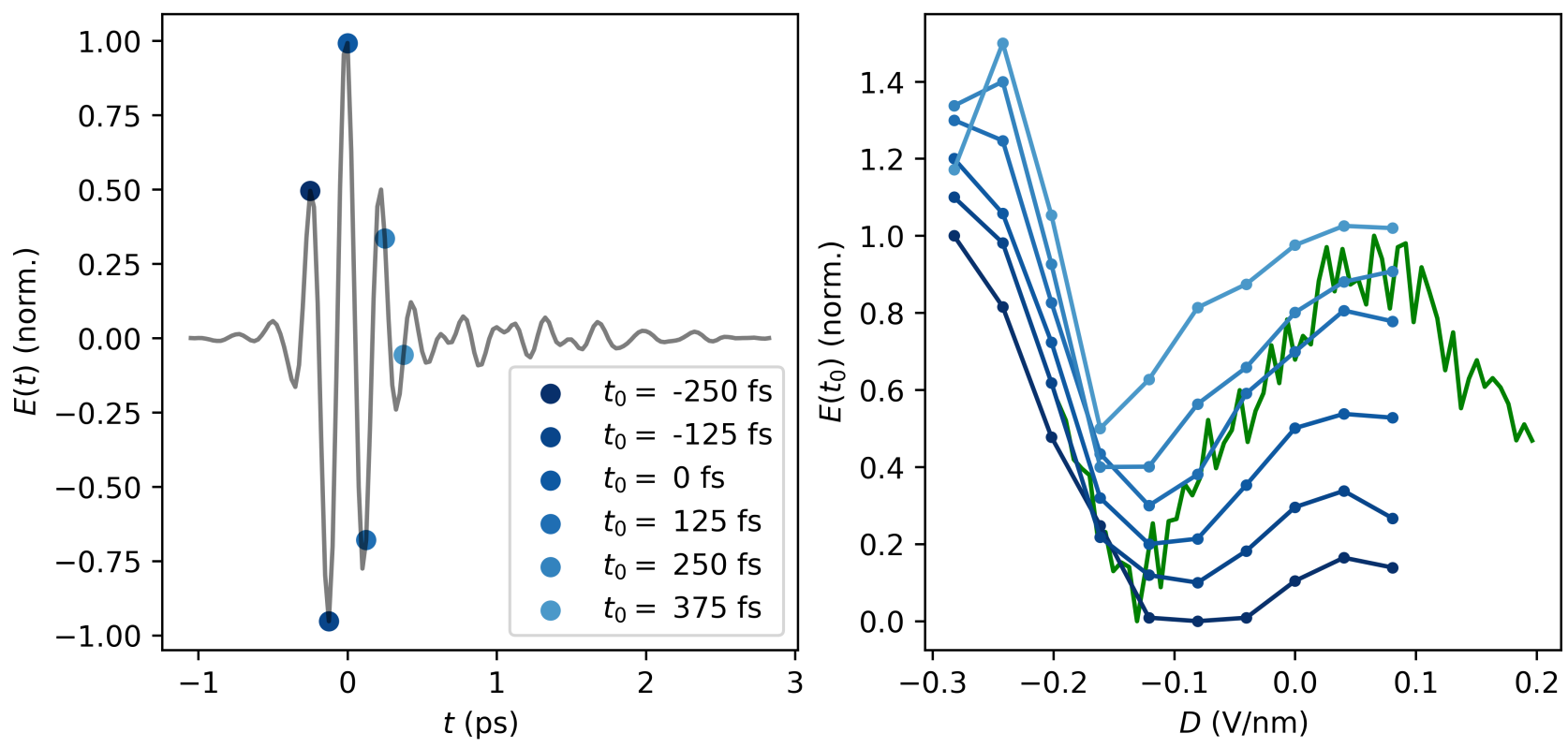

Figure R6: Displacement field-dependent THz transmission at various time delays. Left Transmitted THz pulse with various time-delays marked. **Right** Normalized transmission amplitude as function of displacement field for each time delay marked on the left panel (blue curves) along a cut at constant $n = 0$ through the map in Fig. 2B (green curve). The curves are off-set for clarity by 0.1. The data were measured at 5 K on device D1.

shows the same qualitative dependence on D when evaluated at different delays across the main pulse (see Fig. R6). While the magnitude varies slightly with delay,the position of the waveform maximum does not shift systematically with gate voltages within our temporal resolution. We now include in the Supplementary Material overlays of $E(t)$ at representative (D) and fixed-delay line scans taken at several delays (on and off the main peak). The qualitative variations are minor and the trend of Figure R6 is confirmed.

Comment 2 - In Fig. 2B, the data is shown as normalized relative THz transmission. The authors should consider presenting non-normalized and normalized signals together.

In Fig. 2B we plot the electro-optic sampling (EOS) signal at the peak of the transmitted THz waveform as a function of top and bottom gate voltages. For visualization we use a monotonic min–max rescaling:

$$T_{\mathrm{norm}} = \frac{T(V_{\mathrm{TG}}, V_{\mathrm{BG}}) - T_{\mathrm{min}}}{T_{\mathrm{max}} - T_{\mathrm{min}}},$$

where $T(V_{\mathrm{TG}}, V_{\mathrm{BG}})$ is the EOS signal and $T_{\mathrm{max}}, T_{\mathrm{min}}$ are its extrema over the displayed voltage range.

To address the referee's request, we now present the *raw* EOS map (reported in mV) *alongside* the normalized map (Figure R7); both are added to the Supplementary. The raw units arise from balanced detection: the THz field imprints a differential optical signal that is read out on a

pair of photodiodes and amplified by a transimpedance stage.

It is worth noting that while Fig. 2B is a fixed-delay EOS map with simple min–max normalization, all frequency-resolved data in Figs. 3, 4, and 6 are presented as *referenced* spectra, i.e. the complex ratio $E_{meas}(\omega)/E_{ref}(\omega)$ with the reference taken at the insulating phase ($n = 0$, finite D).

In case the referee intended to see these unreferenced signals as well, we provide such spectra in Supp. Fig. S2.

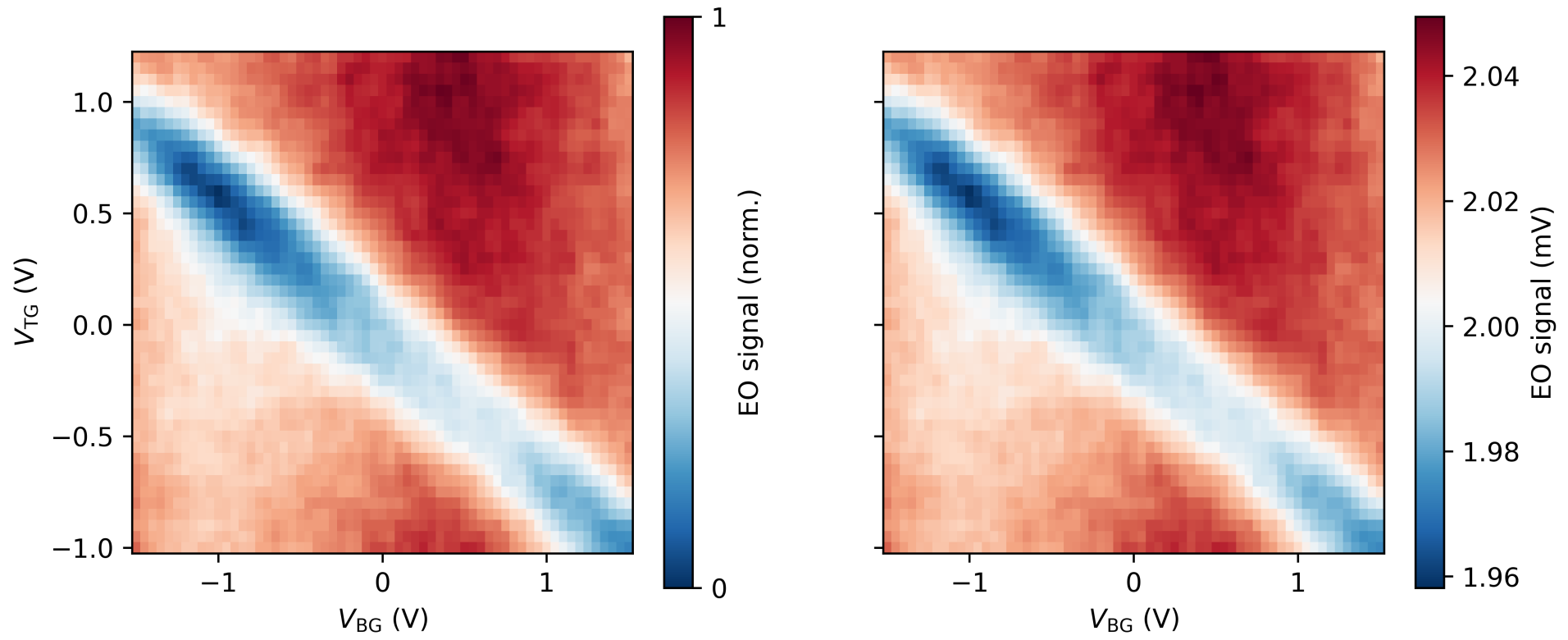

Figure R7: Normalized and un-normalized single time-domain data as presented in Fig. 2B of the main text.

Comment 3 - The experimental data in Figures 3A and 3B have sparse data points along the frequency axis. The data resolution is 0.2 THz. What is the reason for the lower frequency resolution? Could you display the data points alongside the lines connecting them in Fig. 3B?

In time-domain THz spectroscopy the spectral resolution $\Delta\nu$ is set by the available time window Δt, with $\Delta\nu \simeq 1/\Delta t$. In our setup Δt is limited to just 3.9 *ps* by (i) the first echo of the THz pulse (Fabry–Pérot reflections in the optics and substrate), which forces a truncation of $E(t)$ to avoid etalon artifacts, and (ii) the signal-to-noise ratio achievable in long scans.

Each spectrum for a fixed D or n in Figs. 3, 4, and 6 required $\sim$ 6 hours of averaging, and a full map takes nearly four days—practically capped by the cryogen hold time (100 liters of Helium used with a flow cryostat).

Increasing the resolution by a factor of two would necessitate proportionally longer time traces and averaging, which is not feasible under these constraints. The resolution of $\sim$ 0.26 THz is therefore an experimental compromise that allows to gather enough spectra for enough D and

n points.

Importantly, all spectral features observed in our experiments vary on scales well above 0.26 THz, so our conclusions are unaffected. To address the presentation: we now overlay the measured frequency samples as markers on Fig. 3B (reported here as Figure R8). We also corrected for a small calibration mistake in the density axis of Fig. 3 amounting to changes of less than 10%.

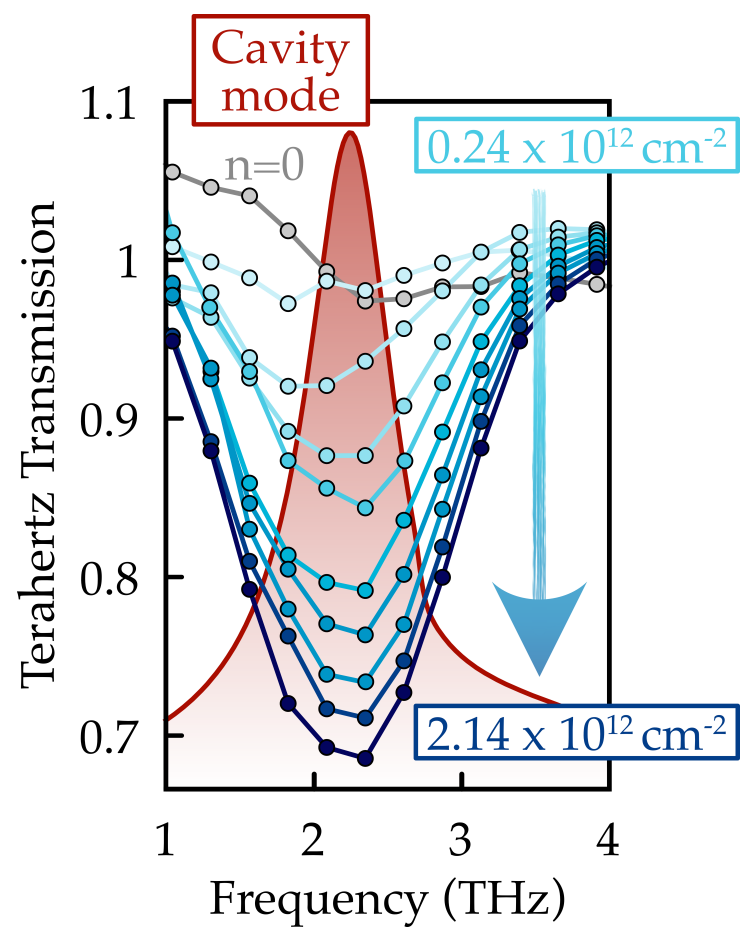

Figure R8: Image 3B of the main text.

Comment 4 - The authors drew simple curves and lines in Figures 4A and 4B, but I do not know how they created these lines from their data points. Those curves and lines are simply drawn as guides for the eyes rather than from quantitative curve fittings.

The curves drawn in Fig. 4A–B were indeed guides to the eye in the original submission; we did not intend them to be read as fits. In the revision we replace them with simulated polariton energies and provide the underlying analysis (see response to comment 5 for a detailed explanation).

Comment 5 - Nevertheless, the authors quantitatively extract the cavity resonance frequency of 2.3 THz and, more importantly, the vacuum Rabi frequency of 1 THz from these data. The authors should detail their experimental analysis and explain how they arrived at these values, providing proper error estimation. The authors state that the cavity coupling coefficient is 43%, claiming that they have at least two digits of significance. Since this is the most important quantitative claim the authors are making, they need to provide further quantitative justification.

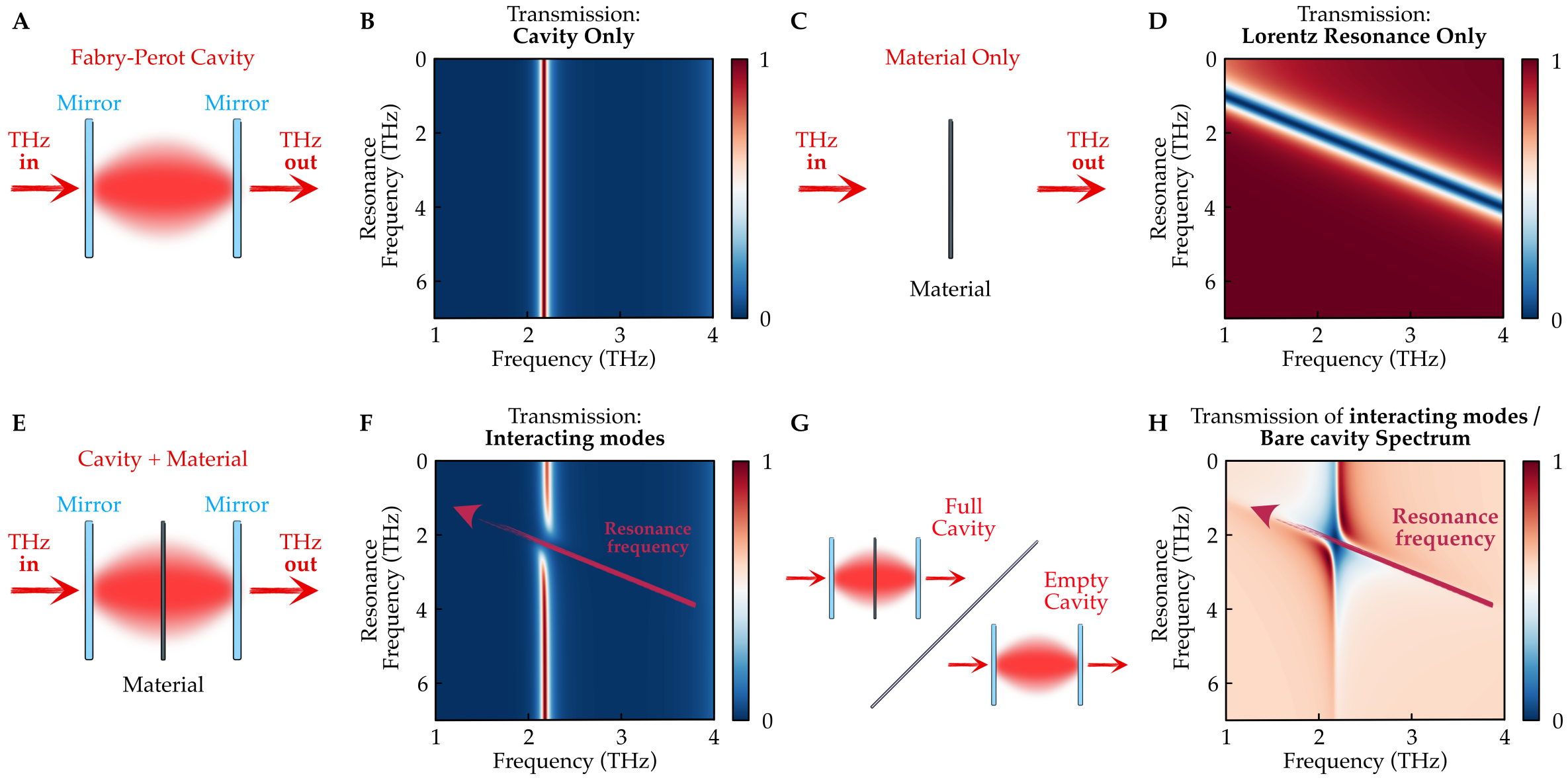

Figure R9: Strong coupling of a Lorentz oscillator to the fundamental mode of a FP cavity. **A** Schematic of a Fabry-Pérot (FP) cavity formed between two parallel metallic mirrors. The fundamental mode is sketched in red. Red arrows mark the incident and transmitted THz fields. **B** Normalized transmission spectrum of the empty FP cavity. **C** Schematic of a thin film with incident and transmitted THz fields marked by red arrows. **D** Normalized transmission spectrum of a thin film with a dielectric constant characterized by a Lorentz model as defined in Eq. (3) as the resonance frequency ω_0 is varied. **E** Schematic of the coupled system. A thin film characterized by a Lorentz resonance is placed in the center of a FP cavity and couples to its fundamental mode. The system is probed in transmission (red arrows). **F** Normalized transmission spectra of the coupled Lorentz oscillator and FP cavity system as function of resonance frequency ω_0. An anti-crossing is observed when the material and cavity frequency are resonant. **G** Schematic for in-situ referencing. The spectrum of the coupled system is divided by the spectrum of the empty cavity. **H** Transmission spectra of the coupled system referenced to the bare cavity spectrum. The spectra are computed from the data in panels B and F and are normalized for clarity. All spectra in this figure have been calculated with the transfer matrix method.

Our initial estimate of the interaction strength $((g/\omega_c = 0.43))$ was obtained at zero detuning by fitting the two observed experimental peaks with Lorentzian line shapes and taking half their separation. As the referee notices, given the rather large ~ 0.26 THz spectral resolution and noise, this procedure carries a systematic uncertainty of at least 0.13 THz in determining each peak position. For these reasons, the interaction strength is better expressed as $g/\omega_c \approx 0.43\,(\pm 0.04)$.

Furthermore, to address the referee's request for a more rigorous, reproducible estimate and to reduce sensitivity to a single noisy spectrum, we developed a second, independent analysis based on a minimal Fabry–Pérot (FP) model that reproduces the polariton dispersion. The setup includes a *thin* excitonic film $(t \ll \lambda)$ placed at the center of the cavity (at the field antinode,

Fig. R9E). The film is described by a single Lorentz pole,

$$\varepsilon(\omega) = \varepsilon_\infty + \frac{\Omega_p^2}{\omega_0^2 - \omega^2 - i\Gamma\,\omega}, \tag{1}$$

with exciton frequency ω_0, damping Γ, and oscillator strength encoded in $\Omega_p = 2\pi f_p$.
To introduce the phenomenology of the model, we first consider—*unlike the experiment*—the idealized limit of extremely *sharp* (high-Q)) cavity and matter resonances. The central frequency of the Lorentz oscillator is externally controllable, just as in the experiment. This setup captures the universal behavior of ultrastrong coupling between two modes. The *un-normalized* terahertz transmissions of the bare cavity (Figure R9A) and of the isolated material layer are (Fig. R9C) shown as a function of exciton frequency in (Fig. R9B) and (Fig. R9D), respectively. When the material is placed in the cavity ((Fig. R9E)), the two resonances hybridize and produce the avoided crossing evident in the *unnormalized* transmission plotted in Fig. R9F. To mimic the experimental procedure exactly, we then calculate the referenced spectrum by dividing the coupled-system transmission of Figure R9F by the bare-cavity transmission, thereby *highlighting only the changes induced by mode interaction*.
In the quantitative comparison with data ((Figure R10)), the cavity parameters are fixed by the bare-cavity spectrum calculated with finite-elements methods, while the Lorentz-oscillator parameters (oscillator strength and damping) are treated as fit variables; the resonance frequency is set to $\omega_0(D)$, equal to the displacement-field–controlled gap. With this procedure we faithfully reproduce the observed spectra. The experimental dataset and the corresponding simulations are shown side-by-side in Fig. R10A and Fig. R10B, respectively.
In this two-oscillator model and in the thin-film limit, the zero-detuning splitting obeys

$$\Omega_{\text{Rabi}} = 2g \;\approx\; f_p\sqrt{F}, \qquad F = \frac{t}{L_c}, \tag{2}$$

where F is the mode–volume filling fraction and L_c is the cavity length. Using the fitted parameters we obtain $g \simeq 1$ THz, i. e. $g/\omega_c \simeq 0.43$, consistent with the direct peak–splitting analysis. In this case as well, the fit under the limited spectral resolution carries an uncertainty of ≈ 0.1 THz on g.

To reflect this uncertainty and avoid over-precision, we report $g/\omega_c = 0.4$ in the main text (one significant figure), while providing $g/\omega_c = 0.43 \pm 0.04$ in the Supplementary.

An important benefit of the FP analysis is that it also provides the differential absorption

($\Delta A = A(\omega, D) - A_{empty\ cavity}(\omega)$), allowing us to quantitatively locate the polariton energies. In the revised Fig. 4B we now plot the LP/UP lines extracted from the model.

We emphasize that the precise numerical value of g is not the central message of the work; it serves to classify the regime. Both independent analyses place us robustly in ultrastrong coupling (conventionally ($g/\omega_c > 0.1$), well above threshold. The key results are the broadband THz spectroscopy of a dual-gated 2D platform and the emergence of a resonance from a continuum consistent with cavity-induced pairing; these conclusions are unchanged within reasonable variations of g (e.g., 30%–50%).

Finally, the compelling agreement of the data with the FP model underscores that a Lorentzian matter resonance is required to explain the observations, reinforcing our interpretation in terms of cavity-induced excitonic pairing.

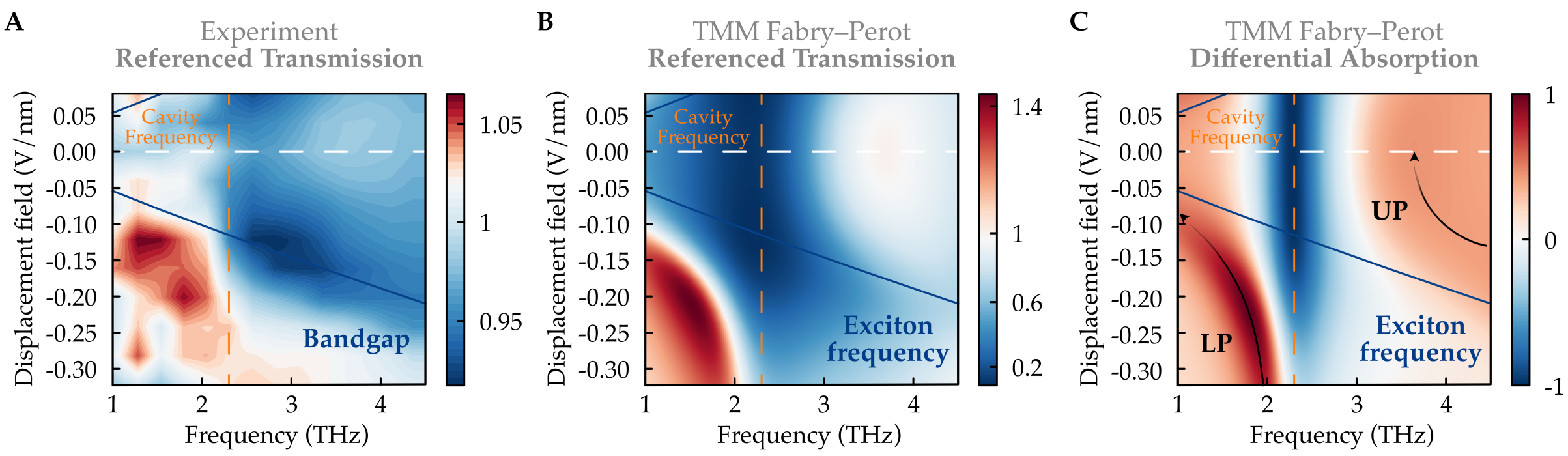

Figure R10: **A** *Experimental data*. Referenced transmission spectra for $D \neq 0$ and $n = 0$. Temperature = 5 K. **B** Simulated referenced transmission spectra using the transfer matrix method. Interaction of a cavity mode in a Fabry–Pérot setting with an exciton defined by the Lorentz model. **C** Differential absorption $\Delta A = A(\omega, D) - A_{empty\ cavity}(\omega)$. The black lines are fits to the maxima in ΔA, which identify the position of the upper (UP) and lower (LP) polaritons

Comment 6 - The theoretical model presented in Fig. 5 needs a clearer description in the text. For example, the authors discuss the Hopefield coefficient and the electron-hole correlation function without providing a proper introduction. While these quantities are discussed in the supplementary materials, the main text does not reference them or the relevant citations.

We thank the referee for pointing out that the description of the theoretical model in Fig.5 needed clearer introduction. In the revision we (i) add references in the main text to the relevant sections of the Supplementary, and (ii) insert brief, self-contained definitions of the quantities plotted in Fig. 5.

Concretely, we now state that the polariton eigenmodes are obtained by diagonalizing the

quadratic light–matter Hamiltonian in the ultrastrong-coupling regime; the resulting eigenvectors define the *Hopfield coefficients*, whose moduli squared quantify the photonic and matter fractions of each polariton. We explain that the plotted coefficient tracks the composition of the lower/upper polariton versus detuning, providing an immediate measure of hybridization. We also add a one-line introduction to the *electron–hole correlation function* as the two-body correlator that measures cavity-induced electron–hole pairing; non-zero amplitude when the continuum edge crosses the cavity signals the emergence of a bound state.

Comment 7 - The authors claim that cavity-induced electron-hole pairing is purely based on the theoretical model, but only the temperature-dependent effect shown in Fig. 6 can indirectly support this claim. While this is an exciting claim, I feel that the authors should also discuss other possible scenarios that could explain the experimental observations. Even better would be if they could present some controlled experiments, such as modifying the cavity coupling by changing the distance between the BLG and the THz cavity, or using a cavity of a different size, to demonstrate that electron-hole interactions are indeed greatly modified in the presence of strong coupling.

We address the comment in the following:

1. *Varying the BLG–cavity separation*. We agree that tuning the BLG–cavity distance would be informative. In our platform the separation is fixed by the ALD-grown AlO_x spacer ($\sim$ 140 nm) that electrically insulates the bow-tie resonator from the heterostructure. Transport experiments in quantum Hall devices with a *hovering* THz resonator ([1]) show that cavity coupling rapidly diminishes for separations of several microns—a regime for which ALD is neither intended nor practical. In our case, a meaningful change (hundreds of nanometers to microns) would require a full process re-optimization and re-design: selecting a new low-loss spacer material, calibrating its growth/deposition, and redesigning the stack and immersion optics. Moreover, thicker spacers would add THz loss (and reduce gate capacitance), further degrading the signal-to-noise ratio. While we genuinely believe that this idea is intriguing, implementing and validating such a spacer stack is therefore a separate fabrication campaign beyond the scope of the present work. We now note this as a concrete direction in the Outlook.

2. *Changing cavity dimensions.* Alternatively, one could vary the cavity geometry to tune g. However, in the inverse bow–tie the cavity frequency ω_c and the coupling g are *not* independently tunable: narrowing the tip–to–tip gap enhances field confinement (increasing g) but simultaneously shifts ω_c and, crucially, reduces external coupling to the far field, thereby lowering the signal-to-noise ratio (SNR). This trade-off is much less severe in split-ring resonators, where a capacitive gap predominantly controls the light–matter coupling while the overall size sets the resonance frequency [42]. We therefore adopted the present geometry as a compromise between achieving a large g/ω_c and maintaining measurable transmission.

   Systematic studies of the gap dependence would likely require switching to a resonator class in which coupling and frequency can be tuned more independently—e.g., complementary split–ring resonators [42]—but at the expense of substantially reduced bandwidth. A narrower spectral window will most likely fail to detect both polariton branches across the full detuning range, defeating the purpose of our broadband approach. Also in this case, implementing such a platform would entail redesign and testing beyond the scope of the present work.

3. *Alternative explanations.* We have examined other scenarios and find them inconsistent with the data. Using transfer-matrix simulations in a Fabry–Pérot geometry (see response to Comment 5), we modeled the material solely by an absorption edge without any bound state (Fig. R11D). The resulting *referenced* transmission and differential absorption are shown in Figs. R11E and R11F, respectively. While a continuum-induced reshaping of the cavity line (Fano-like interference) is visible, *no* avoided crossing and hence *no* polariton branches are observed. By contrast, a model that includes a Lorentz oscillator (Fig. R11A) reproduces the salient features in transmission (Fig. R11B), including polariton formation and the characteristic asymmetric lineshapes (Fig. R11C). We thus conclude that Fano interference of the cavity with a continuum cannot account for the robust, asymmetric two-peak structure observed as the detuning is swept; a discrete, *bound* mode is required. However, we know that our spectra when the bandgap is tuned away from the cavity resonance display only a band edge with no sharp excitonic line—consistent with deliberate Coulomb screening by the nearby gates and with estimates of weak exciton binding and large Bohr radius at the small gaps accessed here [43–46]. Since excitons are neither expected nor observed in our system, the minimal interpretation for our

observation is therefore a cavity-driven reorganization of the interband continuum into a bound state supported by our quantum model. We have revised the main text to make this logic explicit and to reference the supporting simulations in the Supplementary. Note that the simulations are shown in a narrower spectral range to focus on the relevant physics and avoid visual clutter from higher-order Fabry-Pérot modes that are not present in the bow-tie resonator. Extended spectral range simulations are available upon request.

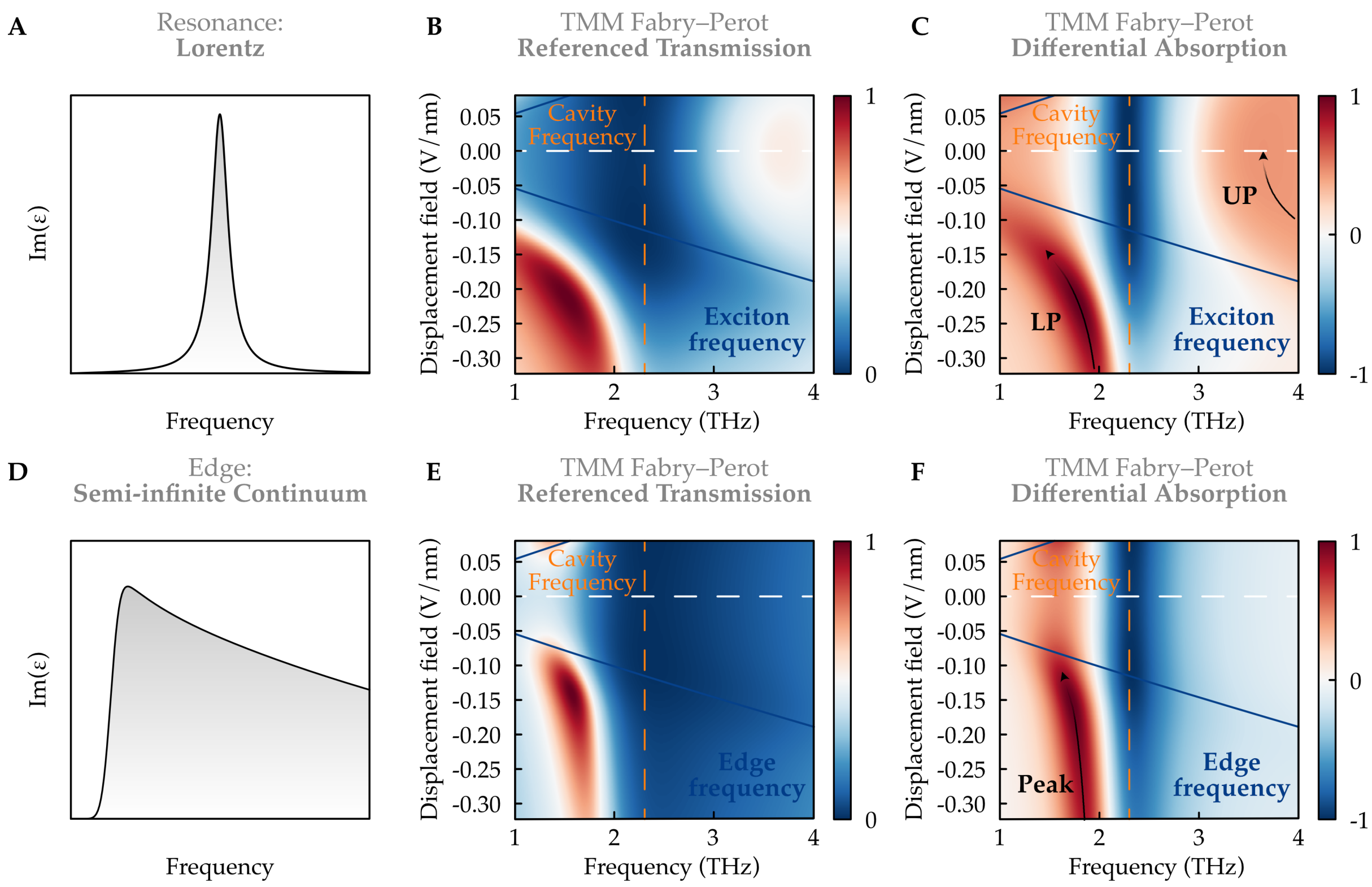

Figure R11: Electrodynamics of a Lorentzian resonance and a continuum in a FP cavity. A/D Dissipative part of the dielectric constant of a Lorentz oscillator **A** and a continuum with an edge enhancement **D**. **B/E**. Transmission spectra of a FP cavity containing a Lorentz oscillator of variable frequency (**B**) at its center or a material character. The spectra are calculated using the transfer matrix method and are normalized to the bare cavity transmission spectrum. **C/F** Differential absorption spectra computed for the same parameters as in **B/E**.

Comment 8 - It seems that the authors performed the experiment on a single device. I wonder what the effect of disorder in the sample would be. At least repeating the experiment on one more device would help to ensure that the effect is not related to point 7 above.

We appreciate the suggestion to test for device-to-device variability. We have fabricated a second device (D2) and added its data to the Supplementary, with cross-references in the main text. D2 is nominally identical to D1 in cavity geometry and BLG–cavity spacing, but employs ultrathin metallic gates (4 nm Cr) instead of few-layer graphite. As expected, the

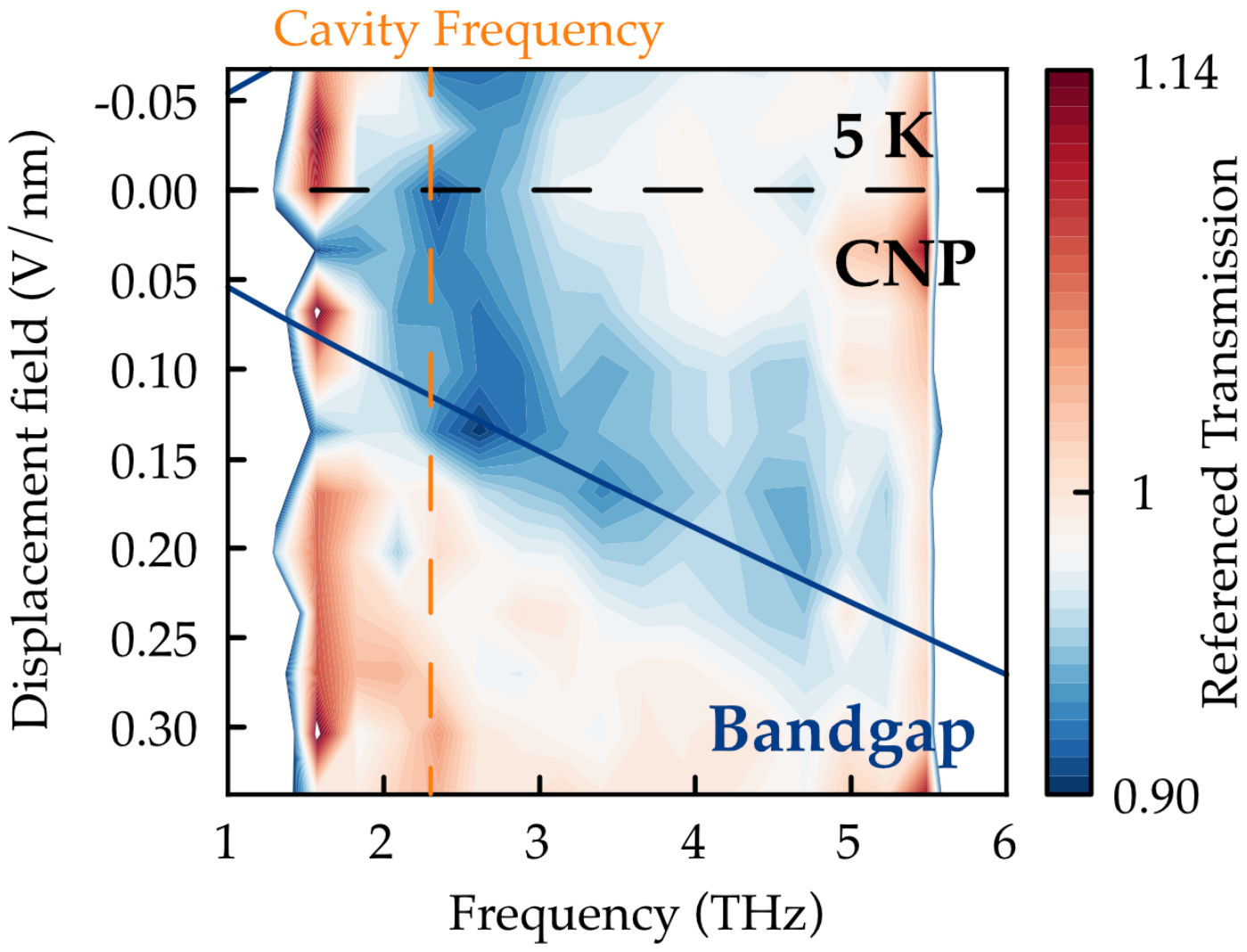

Figure R12: Displacement field-dependent THz transmission spectra for the second device (D2). The spectra are normalized to the insulating state at $D = 0.39\,\mathrm{V/nm}$ and were measured at 5 K. The horizontal black line marks the CNP and the oblique black line represent the calculated band gap within the model of Ref. [48]. The voltage parameters are similar to Fig. 6 of the main text.

rougher metal–hBN interface increases disorder in BLG [47], and free-carrier absorption in Cr reduces the absolute transmission by approximately a factor of two, severely reducing SNR and limiting the usable spectral window to 1.5–5.5 THz. Despite these disadvantages, the *referenced* spectra of D2 (Fig. R12) reproduce the key observations of D1: at $n = 0$ and finite D, we resolve the emergence of two branches and a zero-detuning gap consistent with the coupled-oscillator dispersion (main-text Fig. 4B). The extracted splitting at zero detuning agrees with D1 within our spectral resolution and fitting uncertainty. These results indicate that the effect is robust against increased disorder from metallic gates. More generally, as expected for collective light–matter hybrids, inhomogeneous broadening primarily affects linewidths rather than the Rabi gap, which we indeed find to be stable across devices. Note that in the case of D2 gate leakage currents prevent us from measuring at large negative displacement fields as we did for D1 (Fig. 4 and 6). However, the band structure of bilayer graphene is symmetric under reversal of the sign of D. So, in both cases we access the same physics. For easier comparison with data from D1, we have flipped the vertical axis in Fig.S8.

Referee #3 (Remarks to the Author):

In this work the authors claim to demonstrate the emergence of photon mediated attractive interaction in bilayer graphene integrated with a THz cavity. This is demonstrated through the emergence of a bound state like spectral feature from the lower polariton formation. The basis of this claim is the fact that they are coupling the resonance of the THz resonator with continuum of electronic states in BLG whose bandgap can be tuned via gating. The authors developed a theoretical model that accounts for the ultrastrong coupling and the emergence of the LP state where the role of photon mediated interaction is found to be the main effect. They further go on to carry out temperature dependent measurements where the estimated Rabi splitting is found to stay constant and this is once again attributed to the photon mediated interaction which should be insensitive to temperature.

Worth noting is the ability of the experimental technique to measure directly the bandgap of BLG under field tuning.

We thank the referee for highlighting the unique capability of our technique to *directly* measure terahertz excitations in 2D materials.

While the idea of photon mediated attractive interaction arising from vacuum fluctuations in a cavity to bind charged particles is very intriguing, the experimental results reported in this manuscript does not show that. Below I discuss the main concerns:

We appreciate the referee's interest in the possibility of photon-mediated attractive interactions. Below we address each concern in detail and provide additional analysis, measurements, and simulations. Taken together, the data support our interpretation of a cavity-induced bound state emerging from an interband continuum.

Before discussing each comment individually, we introduce a minimal Fabry–Pérot (FP) model that we will use throughout the response to guide our arguments. The setup includes a *thin* excitonic film $(t \ll \lambda)$ placed at the center of the cavity (at the field antinode, Fig. R13E). The

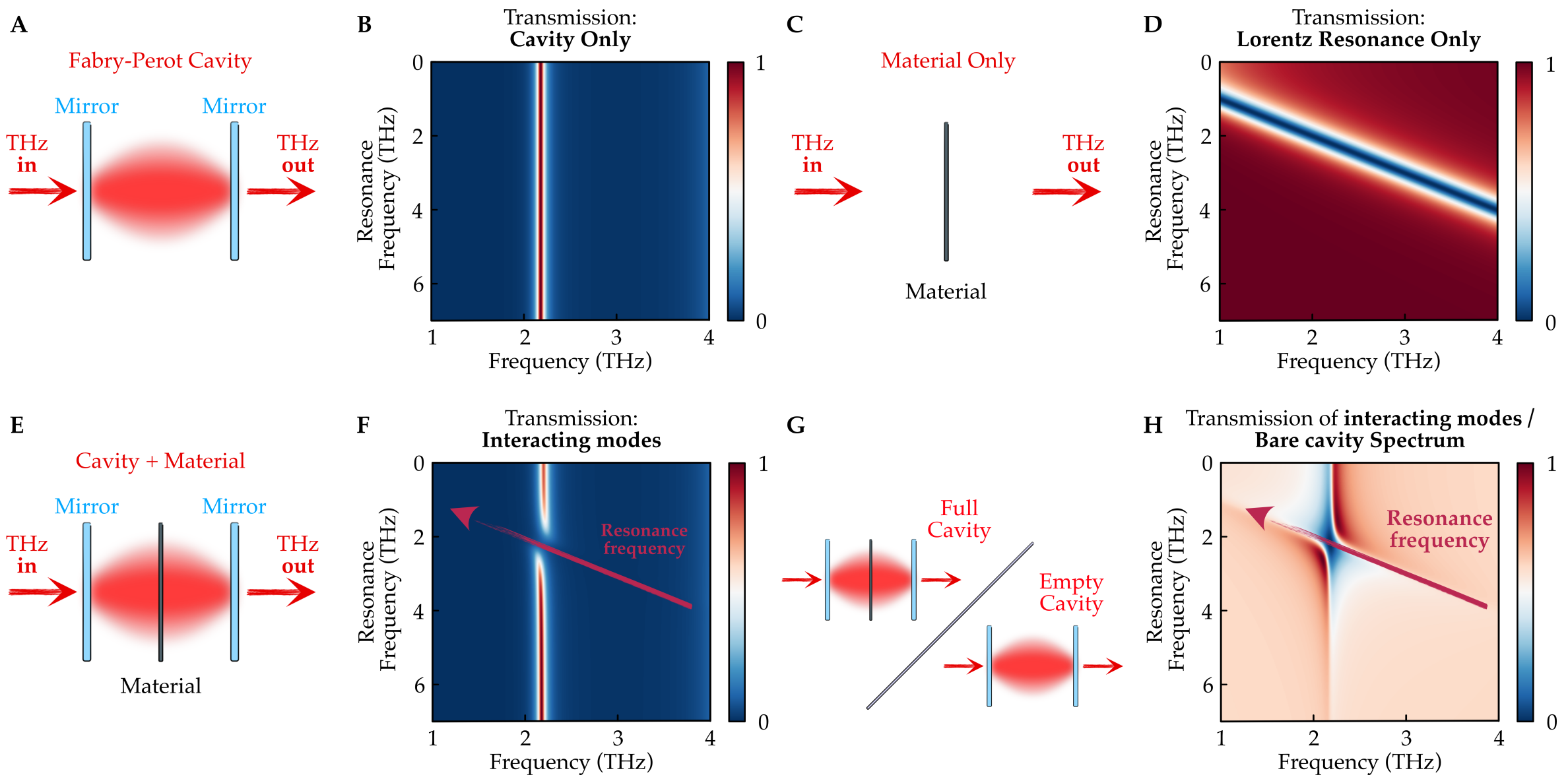

Figure R13: Strong coupling of a Lorentz oscillator to the fundamental mode of a FP cavity. A Schematic of a Fabry-Pérot (FP) cavity formed between two parallel metallic mirrors. The fundamental mode is sketched in red. Red arrows mark the incident and transmitted THz fields. **B** Normalized transmission spectrum of the empty FP cavity when the frequency ω_0 of the Lorentz oscillator is tuned. **C** Schematic of a thin film with incident and transmitted THz fields marked by red arrows. **D** Normalized transmission spectrum of a thin film with a dielectric constant characterized by a Lorentz model as defined in Eq. (3) as the resonance frequency ω_0 is varied. **E** Schematic of the coupled system. A thin film characterized by a Lorentz resonance is placed in the center of a FP cavity and couples to its fundamental mode. The system is probed in transmission (red arrows). **F** Normalized transmission spectra of the coupled Lorentz oscillator and FP cavity system as function of resonance frequency ω_0. An anti-crossing is observed when the material and cavity frequency are resonant. **G** Schematic for in-situ referencing. The spectrum of the coupled system is divided by the spectrum of the empty cavity. **H** Transmission spectra of the coupled system referenced to the bare cavity spectrum. The spectra are computed from the data in panels B and F and are normalized for clarity. All spectra in this figure have been calculated with the transfer matrix method.

film is described by a single Lorentz pole,

$$\varepsilon(\omega) = \varepsilon_\infty + \frac{\Omega_p^2}{\omega_0^2 - \omega^2 - i\Gamma\,\omega}, \tag{3}$$

with exciton frequency ω_0, damping Γ, and oscillator strength encoded in $\Omega_p = 2\pi f_p$.

To introduce the phenomenology of the model, we first consider—*unlike the experiment*—the idealized limit of extremely *sharp* (high-Q)) cavity and matter resonances. The central frequency of the Lorentz oscillator is externally controllable, just as in the experiment. This setup captures the universal behavior of ultrastrong coupling between two modes. The *un-normalized* terahertz transmissions of the bare cavity (Figure R13A) and of the isolated material layer are (Fig. R13C) shown as a function of exciton frequency in (Fig. R13B) and (Fig. R13D), respectively. When the material is placed in the cavity ((Fig. R13E)), the two resonances hybridize and produce the avoided crossing evident in the *unnormalized* transmission plotted in Fig. R13F. To mimic

the experimental procedure exactly, we then calculate the referenced spectrum by dividing the coupled-system transmission of Figure R13F by the bare-cavity transmission, thereby *highlighting only the changes induced by mode interaction*.

With the working model in hand, we can now explore how a strongly coupled light–matter system evolves as the coupling strength is increased, and how this would appear in a *normalized* transmission measurement analogous to ours (division by the bare-cavity spectrum). In the model, the coupling is tuned by increasing the Lorentz oscillator's plasma frequency (oscillator strength). As the coupling grows (Fig. R14A–H), the Rabi splitting increases, the lower polariton (LP) gains contrast and becomes more pronounced, while the upper polariton (UP) weakens —an asymmetric intensity evolution that mirrors the experiment.

In the quantitative comparison with data ((Figure R15)), the cavity parameters are fixed by

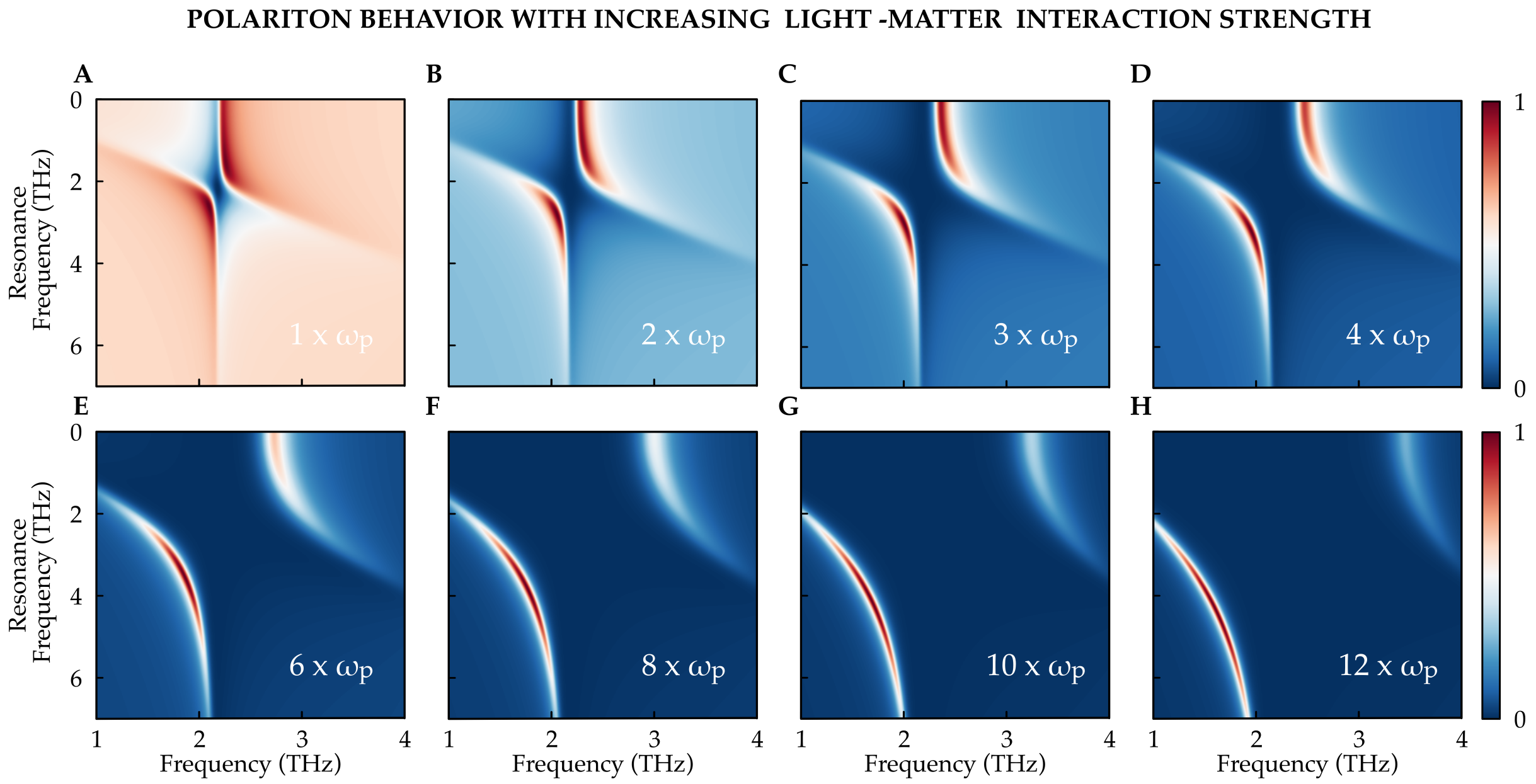

Figure R14: Weak to ultrastrong coupling of a Lorentz oscillator in a FP cavity. A-H Referenced transmission spectra of a Lorentz oscillator with tunable frequency ω_0 placed at the center of a FP cavity at various plasma frequencies ω_p (marked in each panel). The spectra are computed using the transfer-matrix method and normalized to the bare cavity transmission without oscillator present. The color scale is normalized for each panel separately.

the bare-cavity spectrum calculated with finite-elements methods, while the Lorentz-oscillator parameters (oscillator strength and damping) are treated as fit variables; the resonance frequency is set to $\omega_0(D)$, equal to the displacement-field–controlled gap. With this procedure we faithfully reproduce the observed spectra. The experimental dataset and the corresponding simulations are shown side-by-side in Fig. R15A and Fig. R15B, respectively.

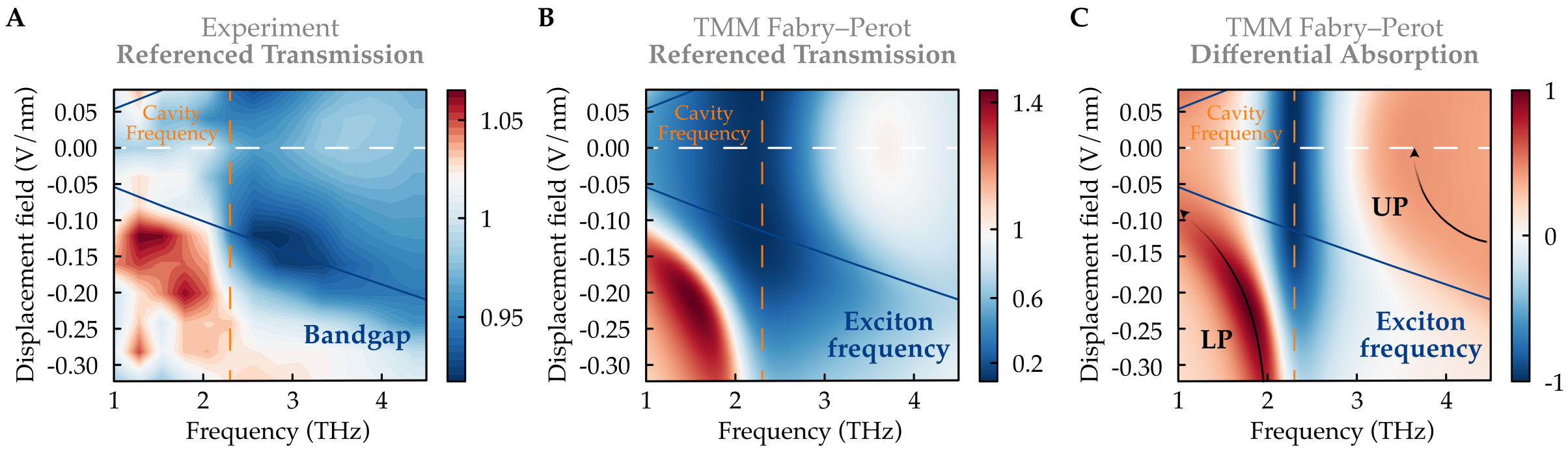

Figure R15: **A** *Experimental data*. Referenced transmission spectra for $D \neq 0$ and $n = 0$. Temperature = 5 K. **B** *Simulated referenced transmission spectra* using the transfer matrix method. Interaction of a cavity mode in a Fabry–Pérot setting with an exciton defined by the Lorentz model. **C** Simulated differential absorption $\Delta A = A(\omega, D) - A_{empty\ cavity}(\omega)$. The black lines are fits to the maxima in ΔA, which identify the position of the upper (UP) and lower (LP) polaritons.

Comment 1 - Firstly, the present experiments uses a THz probe to observe the polariton states. This is unlike the ones reported in Refs 23-25 from the Faist group where there is no external electromagnetic radiation being input. The experiments reported here are similar to standard white light reflectivity or transmission experiments that have been reported since the first work of Weisbuch (1992). So what is new here? I am not questioning if polariton states are the new eigen states in the presence of cavity. But the results shown here does not in any way demonstrate the role of vacuum fluctuations.

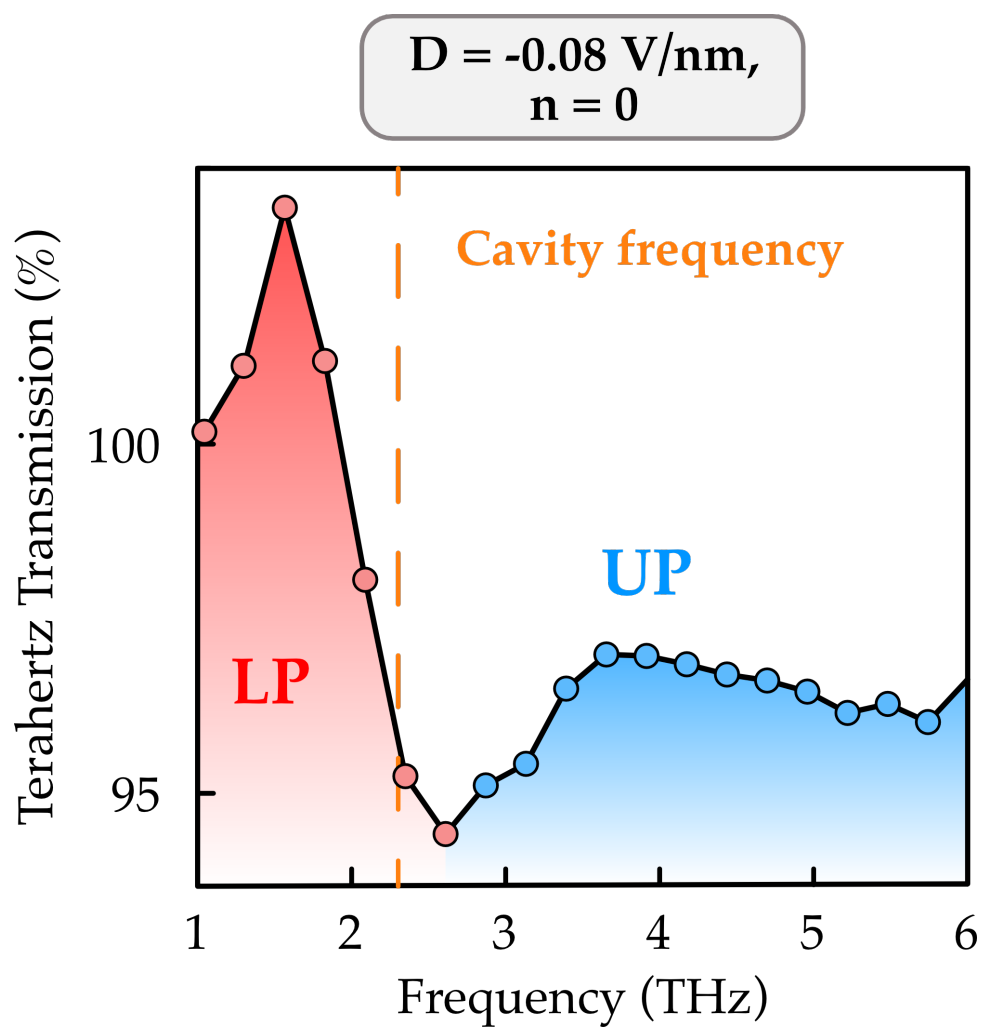

Figure R16: Referenced transmission spectrum for when the bandgap approaces the cavity frequency ($\omega_g \approx \omega_c$). The two observed peaks are associated with the lower (LP) and upper (UP) polaritons.

We thank the referee for raising this important point regarding the role of the THz probe.

The novelty of our work lies not in the measurement principle—which does indeed employ a weak probe to interrogate the system and constitutes the THz analogue of white-light reflectivity/transmission used at optical wavelengths—but in the underlying physics we observe and model.

What fundamentally distinguishes our observation from conventional quantum-well or 2D-semiconductor systems (as in Weisbuch *et al.* [49]) is that the excitonic resonance that participates in strong coupling is *absent* when the system is tuned off resonance (See Fig. R19). The observed lack of excitons in BLG is consistent with weak Coulomb binding at terahertz frequencies, large Bohr radius and screening by nearby gates [43–46]. Yet, on resonance, we observe a robust anti-crossing (Fig. R16). This raises a fundamental question: *How does one obtain a polariton splitting when no discrete resonance exists to begin with?*

As detailed in our response to Comment 2, coupling to a continuum alone cannot yield a polariton splitting, and as shown in our response to Comment 3, the observed splitting is a genuine light-matter anti-crossing, not an artifact. The quantum model of Fig.5 addresses this puzzle directly. The electron-hole correlation function in Fig.5E reveals that photon exchange binds electrons and holes, *inducing* a discrete bound state out of an interband continuum. This represents a qualitatively different mechanism: rather than cavity fields hybridizing with pre-existing excitons, they *create* the bound state itself through photon-mediated attractive interactions.

Related works in which cavity coupling primarily modifies transport properties [22, 23, 50] focus on ground-state behavior; by contrast, we show that cavity fields can *reshape the excitation spectrum* by creating a cavity-induced resonance.

Regarding the role of vacuum fluctuations versus probe photons: the referee correctly notes that linear-response THz spectroscopy cannot unambiguously distinguish between purely quantum vacuum fluctuations and probe-mediated photon exchange. In conventional systems, polariton formation can be explained semiclassically. To address this concern and avoid ambiguity, we have revised our wording throughout the manuscript, replacing ”vacuum fluctuations” with ”cavity fields” or ”photon-mediated interactions” where appropriate. Nevertheless, we would like to point out that the failure of continuum-only classical models to reproduce our data (Comment 2, Fig. R17, Suppl. Info. 7), together with the success of our quantum model, supports the need for a quantum-mechanical description of the interaction and resonance formation.

The core observation remains: a discrete bound state that does not exist in the bare material

is induced by coupling to the cavity through photon-mediated electron-hole attraction. This mechanism fundamentally differs from the hybridization of pre-existing resonances and extends polariton physics into a regime where the cavity actively reshapes the excitation spectrum.

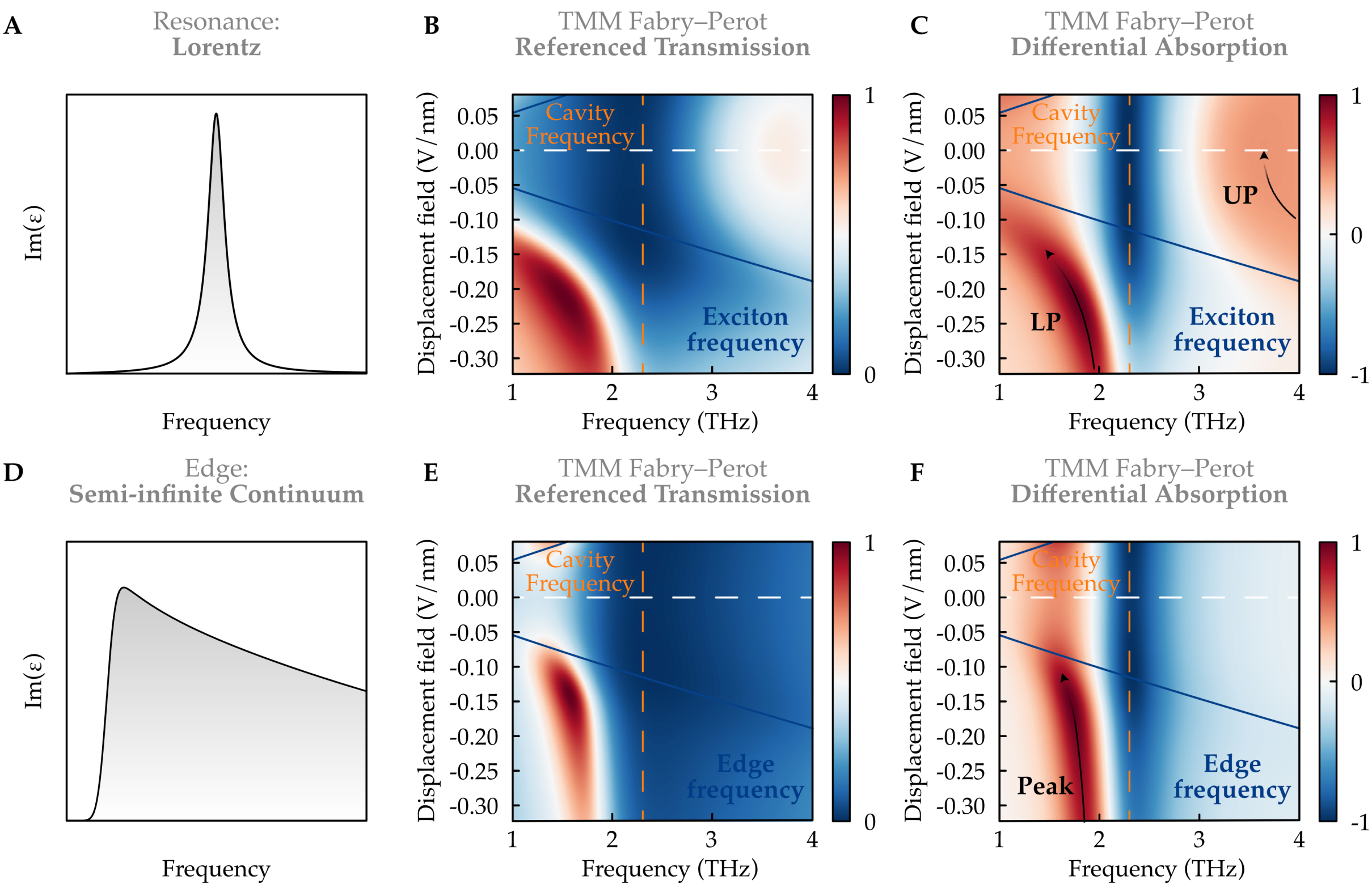

Figure R17: Electrodynamics of a Lorentzian resonance and a continuum in a FP cavity. A/D Dissipative part of the dielectric constant of a Lorentz oscillator **A** and a continuum with an edge enhancement **D**. **B/E**. Transmission spectra of a FP cavity containing a Lorentz oscillator of variable frequency at its center (**B**) or a material characterized by an absorption edge. The spectra are calculated using the transfer matrix method and are normalized to the bare cavity transmission spectrum. **C/F** Differential absorption spectra computed for the same parameters as in **B/E**.

Comment 2 - Emergence of polariton states when coupling of a continuum of electronic states to a cavity photon is used as the evidence for the formation of a bound state. Isn't Fano resonances that have been studied extensively an example of a narrow spectral feature arising from the coupling between a discrete state with a continuum? Why is the feature observed not attributed to the usual Fano resonance?

We thank the referee for raising the connection to Fano interference [51], which lets us clarify both the similarity and the crucial differences. In the conventional Fano resonance, a discrete level (e.g., a cavity mode) couples *weakly* to a broad continuum, yielding an asymmetric lineshape via interference [52, 53]. This produces a single, dispersively shifted and broadened

resonance—often accompanied by a characteristic zero—but *not* a true normal-mode splitting. By contrast, our data exhibit two *dispersive* branches whose separation is minimal at zero detuning and that track an avoided crossing as the BLG gap is tuned. This behavior requires two hybrid poles (LP/UP) in the response rather than a single Fano-distorted resonance.

We make this distinction quantitative. Transfer-matrix calculations in a Fabry–Pérot stack that include only an interband absorption edge—*even with a generous enhancement at the edge, similar to a van Hove singularity*—distort the cavity line in a Fano-like fashion but never produce an avoided crossing (Fig. R17D–F). In contrast, a minimal model that places a thin Lorentz oscillator at the field antinode reproduces the observed LP/UP dispersion and asymmetric profiles (Fig. R17A–C), matching the experimental referenced spectra (Fig.R15A) across detuning. The quantum mechanical framework in Fig.5 of the main text further shows that strong coupling to a *semi-infinite* continuum (bounded from below by the gap) can reorganize spectral weight and generate a bound state, while a second branch remains above—precisely the phenomenology we observe. Because the continuum has a lower bound, sufficiently strong coupling can expel a hybrid mode below the edge, converting it into a bound state. Related analyses in the intersubband context of qunatum wells reach the same conclusion in the strong-coupling limit [54].

In short, while Fano physics can account for asymmetric reshaping of a single line, it does not explain a robust, detuning-dependent anti-crossing. Our spectra therefore require, and our modeling corroborates, the presence of a discrete bound mode—absent off resonance—that is *created* by the cavity field out of the interband continuum. We have added a concise discussion of this point in the main text and direct the reader to the Supplementary for side-by-side "continuum-only" versus "Lorentz" simulations. Note that the simulations are shown in a narrower spectral range to focus on the relevant physics and avoid visual clutter from higher-order Fabry-Pérot modes that are not present in the bow-tie resonator. Extended spectral range simulations are available upon request.

Comment 3 - A bigger concern in this context is the following: In Fig. 3, the authors investigate the effect of cavity frequency on terahertz (THz) transmission at finite charge density ($n \neq 0$) and zero displacement field ($D = 0$). In both experiments (Figs. 3A, B) and simulations (Fig. 3C), the cavity manifests as a dip in transmission, consistent with screening of the cavity mode by injected carriers, as the authors correctly noted. In contrast, a bandgap in BLG at finite

D and n=0 appears as a shoulder of a broad peak in THz transmission (Fig. 4A). Notably, the cavity dip is narrower than the absorption peak, which is expected since the latter arises from continuum transitions, not a resonance like excitonic transition. When the gap energy and cavity frequency coincide, it is not surprising that a narrow dip superimposed on a broad peak yields a two-peak structure. However, the authors interpret this as the emergence of upper and lower polaritons, which is questionable. It is unclear why dressing the cavity dip with a continuum absorption would convert it into a peak. Even in the context of exciton-polaritons especially in plasmonic systems, similar two-peak structures—arising from the superposition of a narrow spectral feature (here from cavity) and broad absorption—have led to overinterpretation. The authors should clarify why this is not the case.

We believe this concern stems from conflating two distinct measurement configurations. *Figure 3* probes the *doped, ungapped* case ($D = 0$, $n \neq 0$), where intraband (Drude) carriers are injected into the material. Injecting carriers increases both absorption and reflection, thus reducing transmission at the cavity mode. Consequently, the ratio between the doped transmission spectrum and the reference spectrum taken in the insulating phase ($T_{D=0,n\neq0}/T_{D\ll0,n=0}$, Fig. 3A,B) shows a *dip* around ω_c whose depth grows with n. By construction, when the system is undoped ($n = 0$) this Drude–induced dip cannot develop.

By contrast, *Figure 4* probes the *gapped, charge–neutral* regime ($n = 0$, $|D| > 0$): there are no free carriers to produce the Drude-induced dip at the cavity mode. Even thermal activation is negligible ($k_B T \approx 0.43$ meV $\ll \hbar\omega_c \approx 9.5$ meV). In this regime the interband continuum is bounded below by the gap edge $E_g(D)$; as $E_g/\hbar$ is tuned through ω_c, the spectra show two dispersive branches with a minimum splitting at zero detuning—an avoided crossing. The dip mechanism of Fig. 3 (present only for $n \neq 0$ at $D = 0$) is therefore irrelevant for Fig. 4 ($n = 0$), and the referee's explanation cannot account for two branches that move with detuning.

To further substantiate that charge injection and "cavity dips" do not underlie the phenomenology of Fig. 4, we repeated the measurement at a fixed D both *without* and *with* injected carriers. The value of D was chosen so that LP and UP are simultaneously visible in the neutral case. As shown in Fig. R18, introducing even a small concentration of carriers suppresses the polaritonic features, confirming that the two effects are unrelated: the two-branch structure at $n = 0$ arises from hybridization across the gap, whereas the $D = 0$, $n \neq 0$ "dip" is due to Drude screening.

Regarding the wording "shoulder of a broad peak" and "absorption peak" for Fig. 4:

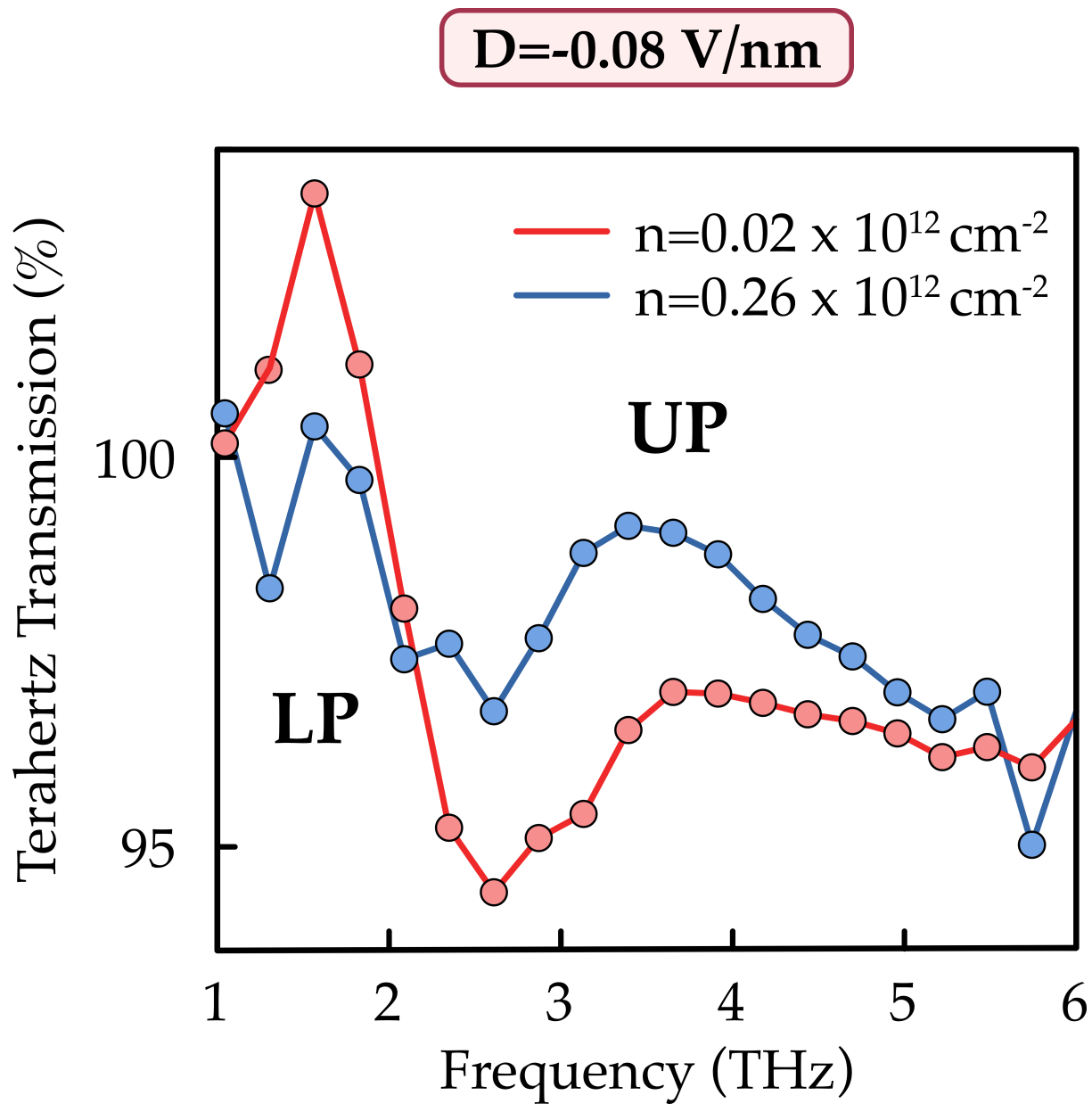

Figure R18: Influence of doping on spectrum at finite displacement field Experimental transmission spectra obtained with n-type doping at finite displacement field $D = -0.08\,\mathrm{V/nm}$. The doping is non-zero for the blue curve and vanishing for the red curve. The spectra are referenced to the insulating state at $D = -0.36\,\mathrm{V/nm}$ and measured at 5 K on D1.

our measurement is in *transmission*. Larger absorption corresponds to *lower* transmission (a depression), not a peak. To make this explicit, we include a schematic (Fig. R19A) showing how an absorption edge produces a downturn in transmission. In Fig. R19B we plot the first four traces at $n = 0$ and $D \ll 0$ (the gap approaching ω_c but before a clear anticrossing; the -0.20 V/nm trace already shows the nascent LP). In this regime the interband edge is clearly identifiable (arrows) and manifests as a *transmission depression* at higher frequencies, as expected.

The apparent "broad peak" appears only in the lightest trace ($D = -0.32$ V/nm) and only near the low-frequency boundary of our acquisition, where the noise floor is higher. We acknowledge this feature and cannot, at present, assign a definitive physical origin to the low-frequency depression.

Two clarifications: First, the reference spectrum was taken in a strongly insulating phase ($D = -0.36$ V/nm in this panel) and is therefore expected to be featureless; nonetheless, small residual artifacts can persist. For example, we occasionally observe a slight overshoot above 100% even when the gap is fully open (Fig. R19B), indicating that the sample can be marginally less absorbing than the reference just below the edge. Second, this vertical offset does not affect identification of the interband edge or its detuning-dependent evolution. Overall, the trend across all curves prior to resonance crossing is consistent with a *moving absorption edge*, not a broad peak, and the same edge is clearly visible in the temperature series of Fig. 6, providing an

independent cross-check.

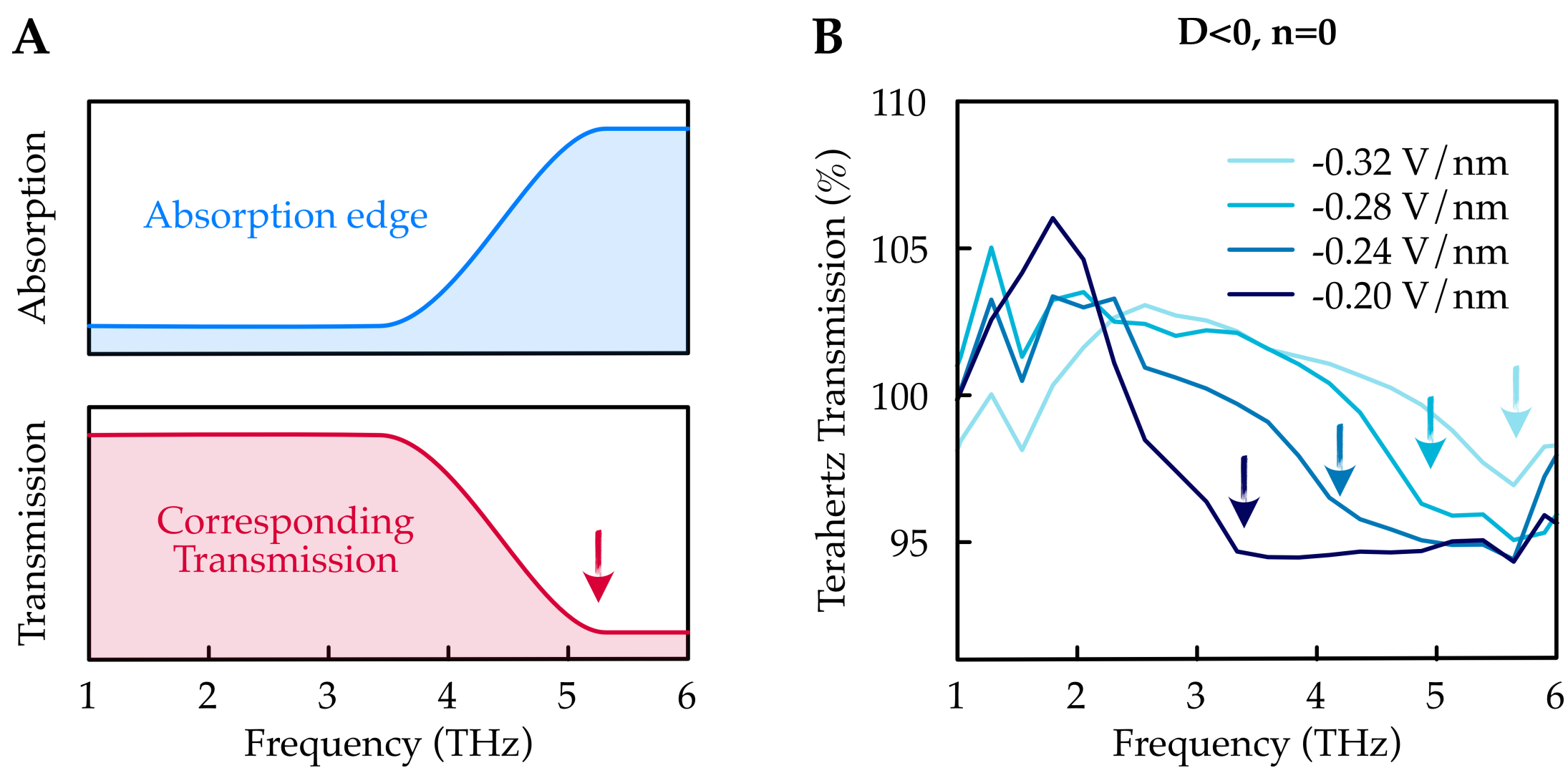

Figure R19: Band edge absorptionA Schematic of THz absorption by a band edge (top) and the corresponding transmission spectrum (bottom) with an arrow marking the location of the band edge. **B** Experimental transmission spectra at various displacement fields $|D| > 0$ and $n = 0$ referenced to the insulating state $D = -0.36\,\mathrm{V/nm}$. The arrows mark the onset of absorption, similarly to panel A. Data obtained from device D1 at 5 K. The line cuts are extracted from Fig. 4A/B of the main text.

Lastly, in response to wording like "especially in plasmonic systems," we clarify that neither the cavity nor the observed matter response is plasmonic in nature. At THz frequencies, bulk gold ($\sim$200 nm in our devices) behaves as a near-perfect conductor, and our inverse bow-tie forms a subwavelength *slot* mode dominated by quasi-electrostatic near fields rather than optical surface plasmons; its response is accurately captured by a quasi-static description. On the material side, the dominant absorption at $n = 0$ arises from interband transitions across the gate-tunable BLG gap. Upon doping, intraband (Drude) absorption grows, and BLG plasmons may exist in principle. However, a plasmonic interpretation is inconsistent with our data: the doping-dependent feature we observe (Figure 3) is *non-dispersive*—it does not shift with carrier density—whereas 2D plasmons at fixed wavevector obey $\omega_{\mathrm{pl}} \propto \sqrt{n}$ and would move markedly with n [41]. In addition, using the model of Tomadin *et al.* [41], we estimate that plasmon modes commensurate with our lateral dimensions ($> 2\ \mu$m) would remain below the cavity frequency and only barely enter our 1–6 THz window over the explored densities (Fig. R20).

Comment 4 - Isn't the carrier induced mode screening just the fact that with increasing doping the transmission decreases because of loss. It is surprising that the authors do not see a change in linewidth.

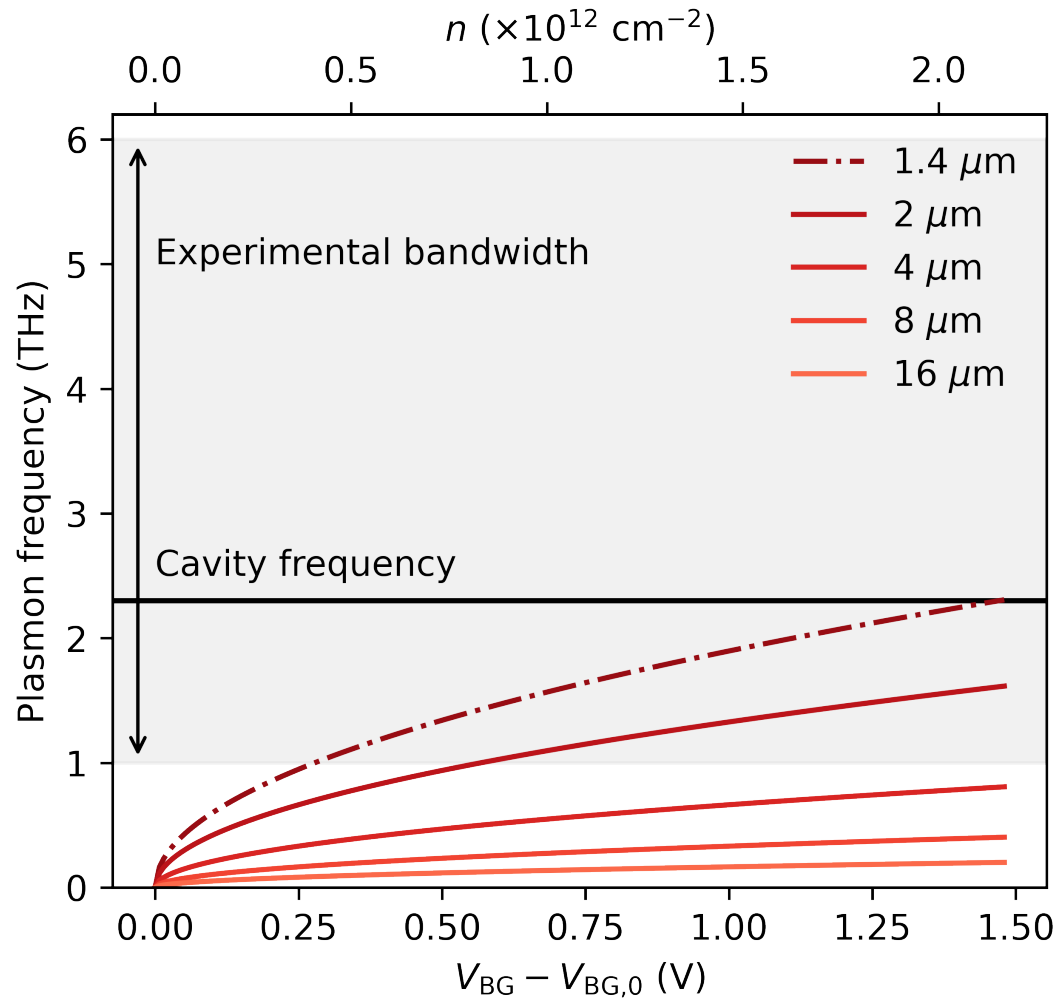

Figure R20: Observability of plasmons. Calculated gate-dependent plasmon dispersion in dual-gated BLG device following Eq. (28). The wave vector is determined by geometric length scales of the resonator. Note that the smallest geometric feature of the resonator in D1 and D2 is 2 µm. The experimental band width is limited between 1 and 6 THz and the cavity resonance is at 2.3 THz (black line). The back gate voltage axis corresponds to the voltages applied experimentally (while keeping $D = 0$) inducing the electron density n.

We thank the referee for the remark. We have replaced "screening" with a more precise description: carrier injection increases both absorption and, via a larger impedance mismatch, the reflectance of the BLG layer, leading to higher dissipation and reduced external field–resonator coupling, thereby lowering transmission. Prompted by this comment, we re-analyzed the line shape in Fig. 3A and confirm a systematic linewidth broadening with increasing doping (Fig. R21A), consistent with our FEM simulations (Fig. R21B).

Comment 5 - The interpretation of two peaks as resulting from light-matter coupling is further complicated by the observation of similar features at D = 0, where BLG should exhibit no bandgap at all. One can understand this as the overlap of a cavity-induced dip with a weak absorption peak at D = 0. Even without a gap, the density of states is insufficient to screen THz transmission, producing a transmission peak. Meanwhile, the cavity resonance can still induce a sharp dip, even at low carrier density. Could the authors clarify why polariton formation should occur in the absence of a bandgap?

We reiterate that the spectra in Fig. 4 are taken at $n = 0$ and the "dip" mechanism operative at $D = 0$, $n \neq 0$ of Fig. 3 is not relevant in this case. In light of our clarification in Response to Comment 3 and the new measurements for finite D and n (Fig.R18), we believe that this alternative interpretation can be set aside.

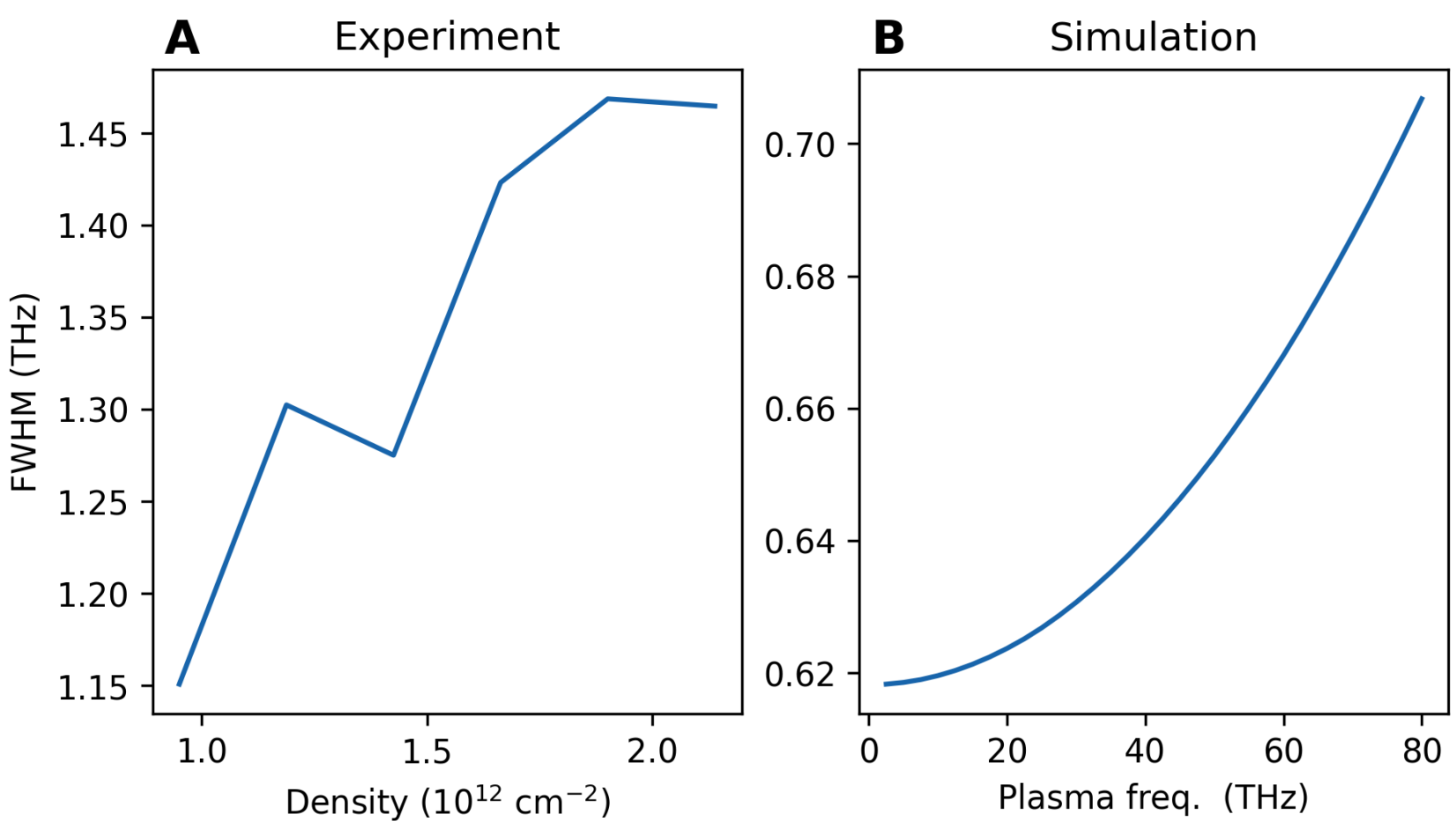

Figure R21: Linewidth as function of doping. **A** FWHM of the spectral dip in the density-dependent measurement of Fig. 3A-B. **B** FWHM of the simulated spectra in Fig. 3C as a function of the plasma frequency ω_p.

Returning to Fig. 4 and its $n = 0$ nature, the remaining point is why a pronounced upper polariton (UP) is still visible at $D = 0$. This behavior is ubiquitous in ultrastrongly coupled light–matter systems and is captured by our Fabry–Pérot simulations (Figs. R13, R14, and R15): as the matter resonance softens toward zero frequency, the UP persists and ceases to disperse, while the lower polariton (LP) loses spectral weight and becomes unresolvable (see Fig. R14). The situation is entirely analogous to Landau polaritons (e.g., [55–57]). In these systems the cyclotron (Landau-level) transition frequency $\omega_c(B) = eB/m^*$ is tuned by the magnetic field and tends to zero as $B \to 0$. When $\omega_c(B)$ is swept across the cavity resonance, the LP progressively loses spectral weight and becomes unresolvable, whereas the UP survives.
In both our simulations and in Landau-polariton experiments, even when the underlying matter excitation tends to zero energy, the UP remains.

The explanation for this behavior in our case (and in related systems) is shown in Fig. 5F of the main text: as $E_g \to 0$, the photonic Hopfield weight of the *surviving* branch tends to unity, i.e., the UP becomes a cavity-like resonance *renormalized* by the interband continuum. At the same time, the electron–hole correlation function associated with the bound state vanishes as the gap closes (Fig. 5F). Thus, observing the UP at $D = 0$ does *not* imply that a bound-state LP survives in the gapless limit; rather, it is the expected cavity-like branch in the ultrastrong-coupling continuation as $E_g \to 0$.

Finally, we draw the referee's attention to the broader outlook outlined at the end of the manuscript: several theoretical works predict that if cavity fields can *create* bound states—as in our case—there may exist regimes where such correlations persist in, or strongly influence, the

ground state, opening a route toward superradiant-like physics in equilibrium settings. While highly speculative and beyond the scope of the present work, our platform provides a concrete path to test these ideas.

Comment 6 - With increasing temperature, thermally activated carriers can screen THz transmission, thus reducing the broad absorption feature. This can explain the temperature dependence observed. However, the authors instead invoke increased non-radiative decay at elevated temperatures. Since the transitions involve a continuum with broad momentum spread and no sharp resonance, it is unclear why non-radiative decay should play a major role. Even if a reorganization into exciton-like states occurs, the resulting transitions would remain broad, since electron-hole pairs for all momenta across broad energy range are involved and bound by cavity photon-mediated interaction (unless this is a resonant effect).

We first clarify the normalization we employ and explain why this is relevant to addressing the referee concerns. Thermally activated carriers *do* significantly reduce the absolute THz transmission—most prominently in the high-resistivity Si substrate and immersion lenses, which introduce free-carrier absorption along the ~ 6.7 mm Si path at elevated T. To remove such temperature-dependent (but D- and n-independent) baselines in the optics, substrate, cavity, or even BLG, *all* spectra in Fig. 6 are referenced to an *in-situ* trace taken at the *same* temperature. Hence, temperature-induced changes that are unrelated to the D-driven light–matter hybridization divide out; the residual trends in Fig. 6 reflect the temperature dependence of the *hybrid* modes themselves.

On the role of dissipation: increasing temperature is well known to broaden linewidths, whether one considers a sharp resonance or an absorption edge. Although the underlying interband manifold is broad, the far-field response samples $q \approx 0$ transitions; the hybrid mode's width therefore *does* inherit the temperature-dependent matter decoherence. This is exactly the situation where the *splitting* (set by g) can remain essentially T-insensitive, while the two branches become unresolvable once the combined linewidth approaches $2g$ [40].

A further, complementary effect is the temperature dependence of the *oscillator strength* and thus of the effective coupling g. Thermal population modifies the band occupations that enter the interband dipole matrix element and its phase space, reducing the edge contrast; this weakens the visibility of the polaritons in transmission. Temperature-driven changes in

population and oscillator strength are a known lever in THz strong/ultrastrong coupling (see, e.g., the temperature series in ref. [58].

Comment 7 - Experimental data in Fig. 4a and 4b are not convincing - especially the claim about upper polariton. In fact 4a with its asymmetric lineshape looks very much like the formation of a Fano resonance.

We would appreciate clarification on what, specifically, the referee finds unconvincing in Fig. 4A–B, and which peak is considered "Fano-like," given that the data show *two* dispersive peaks. In the meantime, we draw attention to the normalized transmission line cuts in Figs. 6E and 6F (reproduced below as Fig. R22A,B), chosen to highlight detunings $D = -0.012$ V/nm and $D = 0.0$ V/nm where the LP and UP peaks are clearly resolved and visible at different temperatures. These line cuts substantiate that we are observing two clear, dispersive peaks which are not compatible with a single Fano-distorted cavity line. We refer the referee to the response to comment 2 for a more detailed discussion.

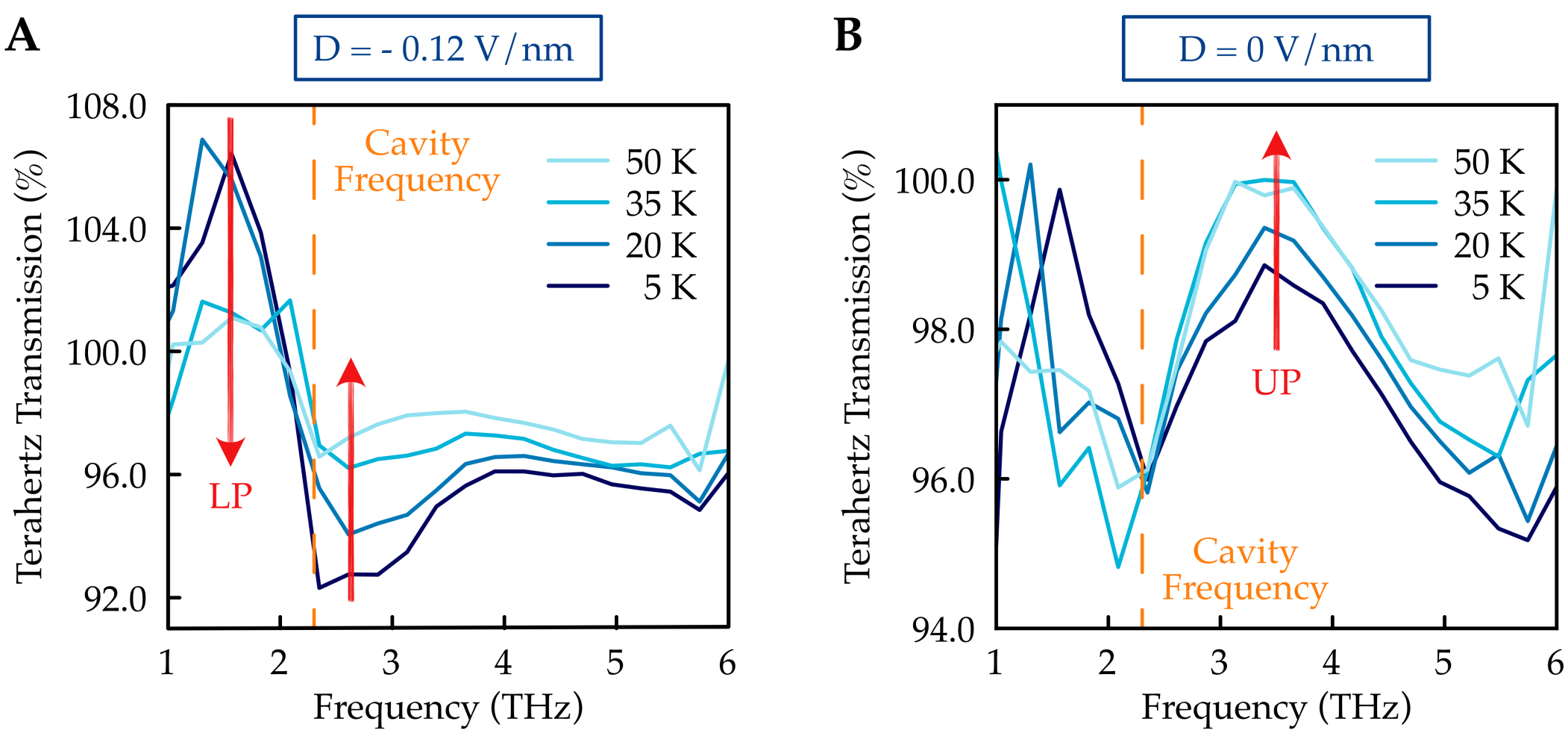

Figure R22: Referenced transmission spectra at two selected displacement fields $D = -0.12$ V/nm (**A**) and $D = -0$ V/nm (**B**) at various temperatures (device D1). Reproduction of Fig. 6E,F of the main text.

Comment 8 - Authors claim that the cavity-driven pairing "reproduces the main experimental spectral features (Fig 4C)". However, notable discrepancy is observed between Fig. 4C and 5B. In Fig. 4C the frequency for UP decreases with decreasing bandgap. In contrast, frequency for UP increases with decreasing bandgap in Fig. 5B. Could the authors comment on this inconsistency?

We would like to clarify that Figs. 4C and 5B are generated from the *same* quantum model using identical parameters and display the *same* underlying quantity: the spectral function. In Fig. 4C we show the spectral function of the coupled system *divided by* the bare-cavity spectral function, following the experimental normalization for direct comparison; Fig. 5B shows the *unreferenced* spectral function. Because the referenced map is a *ratio* of two frequency-dependent functions with a broad cavity maximum near ω_c, the locations of local maxima in the ratio need not coincide with the true polariton poles and can appear to bend with the *opposite* trend. Intuitively, dividing by a non-uniform cavity response enhances features spectrally closer to the cavity peak and suppresses those farther away. This weighting is the origin of the visual discrepancy noted for the UP; when plotted without normalization—or when the polariton poles are overlaid—the two panels are fully consistent.

Comment 9 - The direct measurement of the bandgap of BLG under field tuning is a nice result. Overall, I also found the experimental technique to be very nice. However, the present work is not about these two aspects.

We thank the referee for their favorable assessment of our novel experimental technique and its application to the field-tunable band gap of BLG. We would like to emphasize that this experimental capability—particularly in combination with full, independent dual-gate tuning and broadband THz access exceeding 20 meV—is unprecedented and unmatched by existing techniques. While we agree that the primary focus of our work is the demonstration of cavity-induced bound states and photon-mediated pairing, the spectroscopic platform itself represents a significant methodological advance.

Some minor comments:

Comment 10 - Will be good to see the spectrum of the bare cavity. Since the cavity-induced dip in Fig. 3 is more prominent at higher n, could the authors perform experiments similar to Fig. 4 but at finite n.

The referee raises two distinct points, which we address separately:

1. *Bare-cavity spectrum.* In principle, one could measure the bare-cavity transmission by

fabricating a chip with only the resonator and dividing its spectrum by that of a bare Si wafer mounted between the same immersion lenses. In practice, this *ex-situ* procedure is dominated by alignment-to-alignment (chip and cryostat tilt, focus, beam centering) and chip-to-chip variability, so differences between "cavity-only" and "device" stacks are conflated with mounting and fabrication changes rather than intrinsic properties. We have verified that this approach is unreliable for our setup; we therefore refer the referee to the simulated spectrum in Fig. 3B. Finite-element simulations are a standard workhorse in THz cavity QED and consistently provide accurate predictions; for example, for our device the COMSOL-designed frequency matched the experimental resonance within our resolution.

We emphasize that the key strength of our experimental approach is that a *reference* can be taken *in situ* under identical alignment by opening the gap (large negative D, $n = 0$), thereby factoring out source and optics.

2. *Fig. 4-type scans at finite n.* As noted in our Response to Comment 3, we performed measurements in which we first set a finite D (where both LP and UP are visible) and then introduced carriers. We have performed the experiment with n-type doping as in Fig. 3. Even a modest density $\left(n \sim 2 \times 10^{11}\,\mathrm{cm}^{-2}\right)$ dramatically suppresses the polaritonic contrast (Fig. R23). Consequently, repeating Fig. 4 at finite n does not yield additional insight into the hybrid dispersion; rather, it shows the expected loss-driven suppression of strong-coupling features. For transparency, we include the finite-n line cuts at fixed D in the Supplementary and reference them in the main text.

Comment 11 - Could the authors include the line cut at n = 0 in Fig. 3B? Additionally, could they extend the x-axis to 6 THz, at least in the Supplementary Information. I wonder whether the data show two peaks near the cavity dip, as it seems so by from the map in Fig. 3A around $n = 0.25$?

We have added the $n = 0$ line cut to Fig. 3B (main-text window 1–4 THz) and we provide the corresponding 1–6 THz line cut in the Supplementary (Fig. R24A,B below). Indeed, two spectral features are visible in the data at $n = 0$, these are the two polariton branches. These features are strongly damped as soon as carriers are introduced to the system $n = 0.24 \times 10^{12}\,\mathrm{cm}^{-2}$, similar to what happens at finite D as shown in Fig. R23 and as discussed in the response to

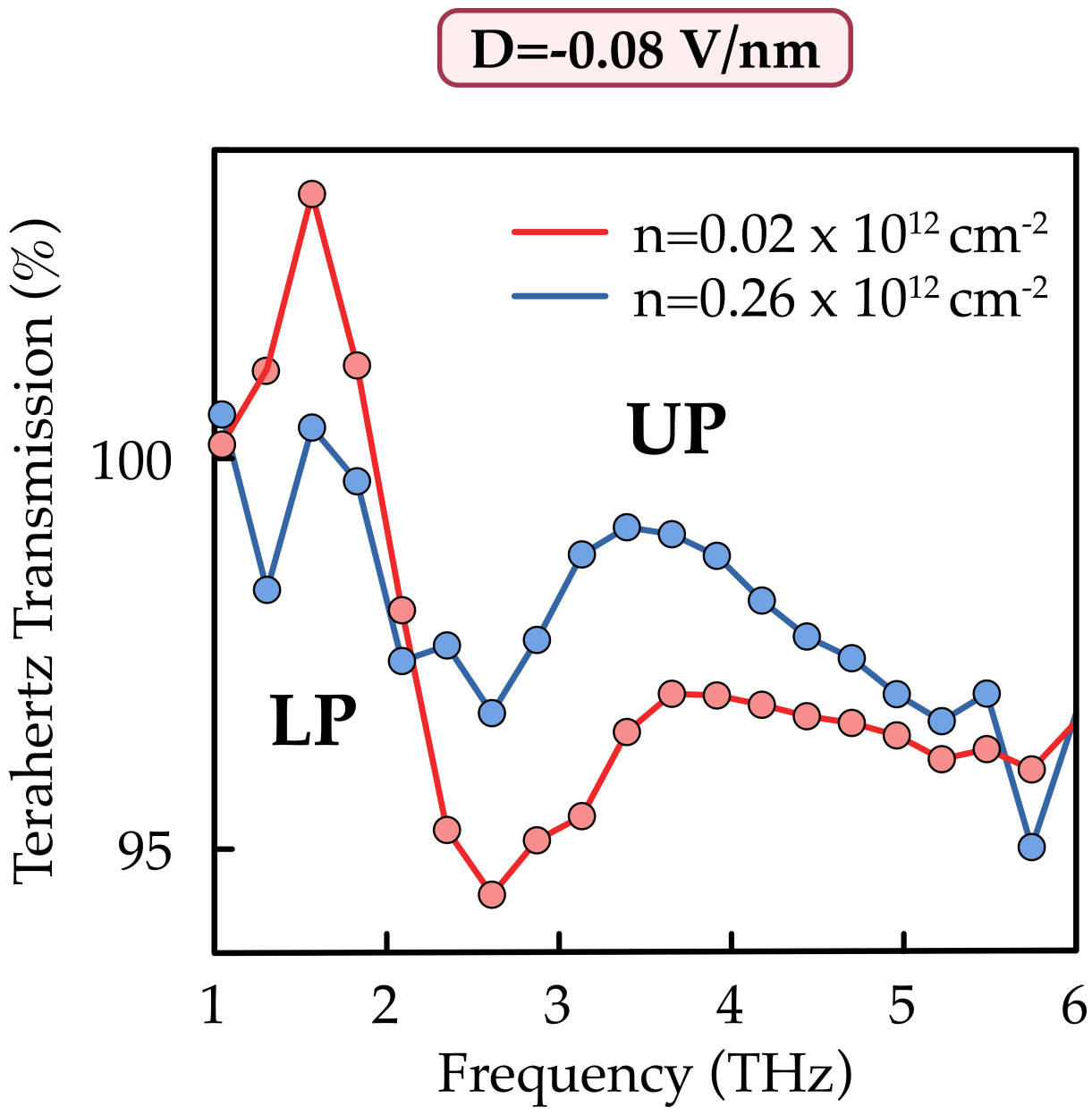

Figure R23: Influence of doping on spectrum at finite displacement field. Experimental transmission spectra obtained with n-type doping at $D = -0.08\,\mathrm{V/nm}$. The doping is non-zero for the blue curve and vanishes for the red curves. The spectra are referenced to the insulating state at $D = -0.36\,\mathrm{V/nm}$ and measured at 5 K on D1.

the previous comment. We would like to note that we have corrected for a slight calibration error in the density axis of this Figure (less than 10%; e.g., $0.26 \times 10^{12}\,\mathrm{cm}^{-2} \rightarrow 0.24 \times 10^{12}\,\mathrm{cm}^{-2}$).

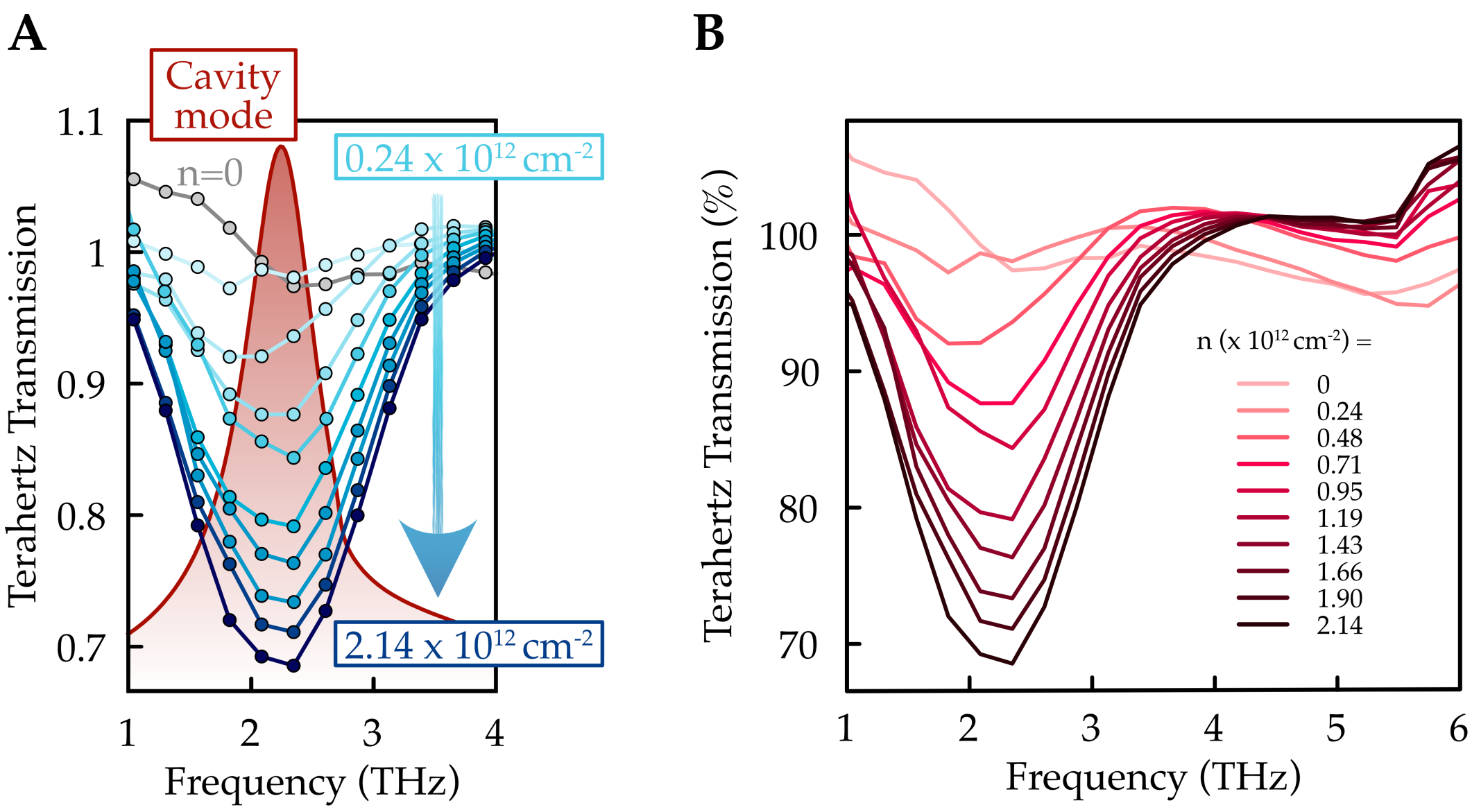

Figure R24: Doping-dependent transmission spectra. **A** Referenced transmission spectra measured at $D = 0$ for increasing electron density (blue) overlaid with the simulated cavity transmission spectrum (red). This panel is a reproduction of Fig. 3B of the main text. **B** Same experimental data as in panel **A** but displayed on an extended spectral axis from 1 to 6 THz. The spectra are referenced to the insulating state at $D = -0.38\,\mathrm{V/nm}$ and were measured at 5 K on device D1.

In summary, while the idea of the paper is intriguing, the experimental results fail to support

the claims and hence I do not recommend publication of this work in Nature. As a new THz spectroscopy technique, it will fit well in a discipline specific journal.

Referee #4 (Remarks to the Author):

I co-reviewed this manuscript with one of the reviewers who provided the listed reports.

We thank the referee for their contribution to the peer review process of our manuscript.

References

[1] J. Enkner, L. Graziotto, D. Boriçi, F. Appugliese, Ch. Reichl, G. Scalari, N. Regnault, W. Wegscheider, C. Ciuti, and J. Faist. Tunable vacuum-field control of fractional and integer quantum Hall phases. *Nature*, 641(8064):884–889, 2025.

[2] P. Gallagher, C.-S. Yang, T. Lyu, F. Tian, R. Kou, H. Zhang, K. Watanabe, T. Taniguchi, and F. Wang. Quantum-critical conductivity of the Dirac fluid in graphene. *Science*, 364(6436):158–162, 2019.

[3] J. W. McIver, B. Schulte, F.-U. Stein, T. Matsuyama, G. Jotzu, G. Meier, and A. Cavalleri. Light-induced anomalous hall effect in graphene. *Nature Physics*, 16(1):38–41, 2020.

[4] A. M. Potts, A. K. Nayak, M. Nagel, K. Kaj, B. Stamenic, D. D. John, R. D. Averitt, and A. F. Young. On-Chip Time-Domain Terahertz Spectroscopy of Superconducting Films below the Diffraction Limit. *Nano Letters*, 23(9):3835–3841, 2023.

[5] W. Zhao, S. Wang, S. Chen, Z. Zhang, K. Watanabe, T. Taniguchi, A. Zettl, and F. Wang. Observation of hydrodynamic plasmons and energy waves in graphene. *Nature*, 614(7949):688–693, 2023.

[6] K. Yoshioka, G. Bernard, T. Wakamura, M. Hashisaka, K. Sasaki, S. Sasaki, K. Watanabe, T. Taniguchi, and N. Kumada. On-chip transfer of ultrashort graphene plasmon wave packets using terahertz electronics. *Nature Electronics*, 7(7):537–544, Jul 2024.

[7] G. Kipp, H. M. Bretscher, B. Schulte, D. Herrmann, K. Kusyak, M. W. Day, S. Kesavan, T. Matsuyama, X. Li, S. M. Langner, J. Hagelstein, F. Sturm, A. M. Potts, Ch. J. Eckhardt, Y. Huang, K. Watanabe, T. Taniguchi, A. Rubio, D. M. Kennes, M. A. Sentef, E. Baudin, G. Meier, M. H. Michael, and J. W. McIver. Cavity electrodynamics of van der Waals heterostructures. arxiv:2403.19745, 2024.

[8] D.R. Grischkowsky. Optoelectronic characterization of transmission lines and wave-guides by terahertz time-domain spectroscopy. *IEEE J. Sel. Top. Quantum Electron*, 6(6):1122–1135, 2000.

[9] D. N. Basov, R. D. Averitt, D. van der Marel, M. Dressel, and K. Haule. Electrodynamics of correlated electron materials. *Rev. Mod. Phys.*, 83:471–541, 2011.

[10] K. S. Novoselov, A. Mishchenko, A. Carvalho, and A. H. Castro Neto. 2D materials and van der Waals heterostructures. *Science*, 353(6298):aac9439, 2016.

[11] S. Haastrup, M. Strange, M. Pandey, Th. Deilmann, P. S. Schmidt, N. F. Hinsche, M. N. Gjerding, D. Torelli, P. M. Larsen, A. C. Riis-Jensen, J. Gath, K. W. Jacobsen, J. J. Mortensen, Th. Olsen, and K. S. Thygesen. The Computational 2D Materials Database: high-throughput modeling and discovery of atomically thin crystals. *2D Mater.*, 5(4):042002, 2018.

[12] J. Yang, G. Chen, T. Han, Q. Zhang, Y.-H. Zhang, L. Jiang, B. Lyu, H. Li, K. Watanabe, T. Taniguchi, Z. Shi, T. Senthil, Y. Zhang, F. Wang, and L. Ju. Spectroscopy signatures of electron correlations in a trilayer graphene/hBN moiré superlattice. *Science*, 375(6586):1295–1299, 2022.

[13] L. Ma, P. X. Nguyen, Z. Wang, Y. Zeng, K. Watanabe, T. Taniguchi, A. H. MacDonald, K. F. Mak, and J. Shan. Strongly correlated excitonic insulator in atomic double layers. *Nature*, 598(7882):585–589, 2021.

[14] Z. Sun and A. J. Millis. Bardasis-Schrieffer polaritons in excitonic insulators. *Phys. Rev. B*, 102:041110, 2020.

[15] F. Xue, F. Wu, and A. H. MacDonald. Higgs-like modes in two-dimensional spatially indirect exciton condensates. *Phys. Rev. B*, 102:075136, 2020.

[16] S. Brem and E. Malic. Terahertz Fingerprint of Monolayer Wigner Crystals. *Nano Letters*, 22(3):1311–1315, 2022.

[17] L. Ju, L. Wang, T. Cao, T. Taniguchi, K. Watanabe, S. G. Louie, F. Rana, J. Park, J. Hone, F. Wang, and P. L. McEuen. Tunable excitons in bilayer graphene. *Science*, 358(6365):907–910, 2017.

[18] D. A. Bandurin, E. Mönch, K. Kapralov, I. Y. Phinney, K. Lindner, S. Liu, J. H. Edgar, I. A. Dmitriev, P. Jarillo-Herrero, D. Svintsov, and S. D. Ganichev. Cyclotron resonance overtones and near-field magnetoabsorption via terahertz Bernstein modes in graphene. *Nat. Phys.*, 18(4):462–467, 2022.

[19] Q. Ma, R. Krishna Kumar, S.-Y. Xu, F. H. L. Koppens, and J. C. W. Song. Photocurrent as a multiphysics diagnostic of quantum materials. *Nat. Rev. Phys.*, 5(3):170–184, 2023.

[20] X. Guo, K. Bertling, B. C. Donose, M. Brünig, A. Cernescu, A. A. Govyadinov, and A. D. Rakić. Terahertz nanoscopy: Advances, challenges, and the road ahead. *Appl. Phys. Rev.*, 11(2):021306, 2024.

[21] G. Jarc, S. Y. Mathengattil, A. Montanaro, F. Giusti, E. M. Rigoni, R. Sergo, F. Fassioli, S. Winnerl, S. Dal Zilio, D. Mihailovic, P. Prelovšek, M. Eckstein, and D. Fausti. Cavity-mediated thermal control of metal-to-insulator transition in $1T$-TaS_2. *Nature*, 622(7983):487–492, 2023.

[22] J. Enkner, L. Graziotto, D. Boriçi, F. Appugliese, Ch. Reichl, G. Scalari, N. Regnault, W. Wegscheider, C. Ciuti, and J. Faist. Enhanced fractional quantum Hall gaps in a two-dimensional electron gas coupled to a hovering split-ring resonator. arxiv:2405.18362, 2024.

[23] F. Appugliese, J. Enkner, G. L. Paravicini-Bagliani, M. Beck, Ch., W. Wegscheider, G. Scalari, C. Ciuti, and J. Faist. Breakdown of topological protection by cavity vacuum fields in the integer quantum Hall effect. *Science*, 375(6584):1030–1034, 2022.

[24] I. Lu, D. Shin, M. K. Svendsen, H. Hübener, U. De Giovannini, S. Latini, M. Ruggenthaler, and A. Rubio. Cavity-enhanced superconductivity in MgB_2 from first-principles quantum electrodynamics (QEDFT). *Proc. Natl. Acad. Sci. U.S.A.*, 121(50):e2415061121, 2024.

[25] Jonathan B. Curtis, Zachary M. Raines, Andrew A. Allocca, Mohammad Hafezi, and Victor M. Galitski. Cavity quantum eliashberg enhancement of superconductivity. 122(16):167002. Publisher: American Physical Society.

[26] Frank Schlawin, Andrea Cavalleri, and Dieter Jaksch. Cavity-mediated electron-photon superconductivity. 122(13):133602. Publisher: American Physical Society.

[27] Valerii K. Kozin, Even Thingstad, Daniel Loss, and Jelena Klinovaja. Cavity-enhanced superconductivity via band engineering. 111(3):035410. Publisher: American Physical Society.

[28] Y. Cao, V. Fatemi, S. Fang, K. Watanabe, T. Taniguchi, E. Kaxiras, and P. Jarillo-Herrero. Unconventional superconductivity in magic-angle graphene superlattices. *Nature*, 556(7699):43–50, 2018.

[29] H. Zhou, L. Holleis, Y. Saito, L. Cohen, W. Huynh, C. L. Patterson, F. Yang, T. Taniguchi, K. Watanabe, and A. F. Young. Isospin magnetism and spin-polarized superconductivity in Bernal bilayer graphene. *Science*, 375(6582):774–778, 2022.

[30] Jiaqi Cai, Eric Anderson, Chong Wang, Xiaowei Zhang, Xiaoyu Liu, William Holtzmann, Yinong Zhang, Fengren Fan, Takashi Taniguchi, Kenji Watanabe, Ying Ran, Ting Cao, Liang Fu, Di Xiao, Wang Yao, and Xiaodong Xu. Signatures of fractional quantum anomalous Hall states in twisted $MoTe_2$. *Nature*, 622(7981):63–68, 2023.

[31] N. Paul, A. Abouelkomsan, A. Reddy, and L. Fu. Shining light on collective modes in moiré fractional Chern insulators. 2025.

[32] X. Li, M. Bamba, N. Yuan, Q. Zhang, Y. Zhao, M. Xiang, K. Xu, Z. Jin, W. Ren, G. Ma, S. Cao, D. Turchinovich, and J. Kono. Observation of Dicke cooperativity in magnetic interactions. *Science*, 361(6404):794–797, 2018.

[33] D. Kim, S. Dasgupta, X-Ma, J.-M. Park, H.-T. Wei, X. Li, L. Luo, J. Doumani, W. Yang, D. Cheng, R. H. J. Kim, H. O. Everitt, S. Kimura, H. Nojiri, J. Wang, S. Cao, M. Bamba, K. R. A. Hazzard, and J. Kono. Observation of the magnonic Dicke superradiant phase transition. *Science Advances*, 11(14):eadt1691, 2025.

[34] G. Mazza and A. Georges. Superradiant quantum materials. *Phys. Rev. Lett.*, 122:017401, 2019.

[35] G. M. Andolina, F. M. D. Pellegrino, V. Giovannetti, A. H. MacDonald, and M. Polini. Cavity quantum electrodynamics of strongly correlated electron systems: A no-go theorem for photon condensation. *Phys. Rev. B*, 100:121109, 2019.

[36] G. M. Andolina, F. M. D. Pellegrino, V. Giovannetti, A. H. MacDonald, and M. Polini. Theory of photon condensation in a spatially varying electromagnetic field. *Phys. Rev. B*, 102:125137, 2020.

[37] Y. Ashida, A. İmamoğlu, J. Faist, D. Jaksch, A. Cavalleri, and E. Demler. Quantum Electrodynamic Control of Matter: Cavity-Enhanced Ferroelectric Phase Transition. *Phys. Rev. X*, 10:041027, 2020.

[38] J. B. Curtis, M. H. Michael, and E. Demler. Local fluctuations in cavity control of ferroelectricity. *Phys. Rev. Res.*, 5:043118, 2023.

[39] T. Liu and Q. J. Wang. Magnetopolariton in bilayer graphene: A tunable ultrastrong light-matter coupling. *Phys. Rev. B*, 89(12):125306, 2014.

[40] S. De Liberato. Virtual photons in the ground state of a dissipative system. *Nat. Comm.*, 8(1):1465, 2017.

[41] A. Tomadin, M. Carrega, and M. Polini. Microscopic theory of plasmon-enabled resonant terahertz detection in bilayer graphene. *Phys. Rev. B*, 103:085426, 2021.

[42] S. Rajabali, E. Cortese, M. Beck, S. De Liberato, J. Faist, and G. Scalari. Polaritonic nonlocality in light–matter interaction. *Nature Photonics*, 15(9):690–695, 2021.

[43] P. Li and I. Appelbaum. Excitons without effective mass: Biased bilayer graphene. *Phys. Rev. B*, 99:035429, 2019.

[44] J. C. G. Henriques, Itai Epstein, and N. M. R. Peres. Absorption and optical selection rules of tunable excitons in biased bilayer graphene. *Phys. Rev. B*, 105:045411, 2022.

[45] M. O. Sauer and Th. G. Pedersen. Exciton absorption, band structure, and optical emission in biased bilayer graphene. *Phys. Rev. B*, 105:115416, 2022.

[46] M. F. C. Martins Quintela, N. M. R. Peres, and T. Garm Pedersen. Tunable nonlinear excitonic optical response in biased bilayer graphene. *Phys. Rev. B*, 110:085433, 2024.

[47] E. Icking, L. Banszerus, F. Wörtche, F. Volmer, Ph. Schmidt, C. Steiner, S. Engels, J. Hesselmann, M Goldsche, K. Watanabe, T. Taniguchi, Ch. Volk, B. Beschoten, and Ch. Stampfer. Transport Spectroscopy of Ultraclean Tunable Band Gaps in Bilayer Graphene. *Adv. Electron. Mater.*, 8(11):2200510, 2022.

[48] E. McCann and M. Koshino. The electronic properties of bilayer graphene. *Reports on Progress in Physics*, 76(5):056503, 2013.

[49] C. Weisbuch, M. Nishioka, A. Ishikawa, and Y. Arakawa. Observation of the coupled exciton-photon mode splitting in a semiconductor quantum microcavity. *Phys. Rev. Lett.*, 69(23):3314–3317, December 1992.

[50] L. Graziotto, J. Enkner, S. Chattopadhyay, J. B. Curtis, E. Koskas, Ch. Reichl, W. Wegscheider, G. Scalari, E. Demler, and J. Faist. Cavity QED Control of Quantum Hall Stripes. arxiv:2502.15490, 2025.

[51] U. Fano. Effects of Configuration Interaction on Intensities and Phase Shifts. *Phys. Rev.*, 124:1866–1878, 1961.

[52] M. Kroner, A. O. Govorov, S. Remi, B. Biedermann, S. Seidl, A. Badolato, P. M. Petroff, W. Zhang, R. Barbour, B. D. Gerardot, R. J. Warburton, and K. Karrai. The nonlinear Fano effect. *Nature*, 451(7176):311–314, Jan 2008.

[53] M. F. Limonov, M. V. Rybin, A. N. Poddubny, and Y. S. Kivshar. Fano resonances in photonics. *Nature Photonics*, 11(9):543–554, 2017.

[54] E. Cortese, I. Carusotto, R. Colombelli, and S. De Liberato. Strong coupling of ionizing transitions. *Optica*, 6(3):354–361, 2019.

[55] G. Scalari, C. Maissen, D. Turčinková, D. Hagenmüller, S. De Liberato, C. Ciuti, C. Reichl, D. Schuh, W. Wegscheider, M. Beck, and J. Faist. Ultrastrong Coupling of the Cyclotron Transition of a 2D Electron Gas to a THz Metamaterial. *Science*, 335(6074):1323–1326, 2012.

[56] Curdin Maissen, Giacomo Scalari, Federico Valmorra, Mattias Beck, Jérôme Faist, Sara Cibella, Roberto Leoni, Christian Reichl, Christophe Charpentier, and Werner Wegscheider. Ultrastrong coupling in the near field of complementary split-ring resonators. *Phys. Rev. B*, 90:205309, 2014.

[57] X. Li, M. Bamba, Q. Zhang, S. Fallahi, G. C. Gardner, W. Gao, M. Lou, K. Yoshioka, M. J. Manfra, and J. Kono. Vacuum Bloch–Siegert shift in Landau polaritons with ultra-high cooperativity. *Nat. Photonics*, 12(6):324–329, 2018.

[58] Y. Todorov, A. M. Andrews, R. Colombelli, S. De Liberato, C. Ciuti, P. Klang, G. Strasser, and C. Sirtori. Ultrastrong Light-Matter Coupling Regime with Polariton Dots. *Phys. Rev. Lett.*, 105:196402, Nov 2010.

Referee response:

Cavity-Driven Attractive Interactions in Quantum Materials

F. Helmrich[1], H. S. Adlong[1,2], M. Kroner[1], I. Khanonkin[1], G. Scalari[1], J. Faist[1], A. İmamoğlu[1], and T. F. Nova[1]

[1]Institute for Quantum Electronics, ETH Zürich, Auguste-Piccard-Hof 1, 8093 Zürich, Switzerland

[2]Institute of Theoretical Physics, ETH Zürich, Wolfgang-Pauli-Strasse 27, 8093 Zürich, Switzerland

Legend:

(i) Maroon: Reviewer comments,

(ii) *Italic: manuscript extracts*,

(iii) Black: Answers to comments.

Response

Referee 1 (Remarks to the Author):

In this revised version the authors answered all the queries raised in my previous review. Specifically:

-C) Data and methodologies. Furhter Temperature measurements have been performed (more T points below and above 100 K might have helped, but it is a minor issue).The effect of gates on the measurements (mostly clarifications, though I wonder the 4 nm Cr uniformity and effectiveness of the second sample) have been exhaustively addressed.

We thank the referee for recognizing the additional experimental effort in the revised submission. We agree that a denser temperature series could in principle be informative. However, the signal-to-noise ratio deteriorates rapidly at elevated temperatures, and each high-temperature measurement already requires multi-day acquisition times, in practice approaching about a week per dataset. For this reason, we limited the extended temperature series to two additional points, 100 K and 200 K, which we believe are sufficient to establish the trend within

the experimental limits of the technique.

Concerning device D2, the continuity of the 4 nm Cr gate film was verified by scanning electron microscopy (SEM). We added the following sentence to the SI 1: *The continuity of the 4 nm Cr film used for the gates in device D2 was confirmed by scanning electron microscopy (SEM).*

D) Statistics.The reproducibility of the measurements from day to day (e.g. Fig R3) and on a different sample support the validity of their approach.

-B) and H) Originiality and context. The authors also modified the manuscript to better promote their approach and findings in a wider range of future applications and theoretical scenarios.

Overall, it is a nice physics and an interesting experimental configuration, appealing to a broad audience and I am therefore happy to support publication.

We thank the referee for their positive assessment of our work.

Referee 1 (Comments on the response to Referee 3)

I read again the authors' response, the manuscript and the very detailed, extensive SI.

The third reviewer seems more critical on the data interpretation and has doubts about the effective realization of an ultrastrong coupling regime with the current experimental conditions.

The key point is the interpretation of the dispersive features as arising from a Fano-like coupling to continuum. Whilst indeed Fano shapes might yield dispersive features, the explanation provided by the authors seem definitively more convincing. The dispersive features, which is clearly observed and include as well an upper polariton, cannot be described by simple Fano resonance, but it requires a discrete state.

We thank the referee for recognizing that the dispersive features in our data, including and especially the upper polariton, are clearly resolved experimentally.

At the same time, we realized that some readers may find high-dynamic-range colormaps (Fig. 3D or Fig.5) or even staggered line plots (Fig. 3A) difficult to interpret. In the colormap representation, the much stronger LP and polaritonic gap dominate the dynamic range, which can make the UP appear visually suppressed when a single common color scale is used. To better highlight the UP, in Fig. F1 we replot the data from Fig. 5A of the main text over the

restricted frequency range 2.3–5.4 THz, spanning from the cavity frequency to near the upper limit of our spectral window. This removes the LP contribution and excludes the noisier high-frequency tail, making the evolution of the UP more clearly visible.

In the new presentation, for each displacement field D we rescale the vertical axis (min-to-max within the displayed frequency window) to highlight the UP peak and its dispersive behavior. This representation avoids the potentially misleading impression that can arise in colormaps with a large dynamic range, where the UP is present but visually compressed relative to the LP.

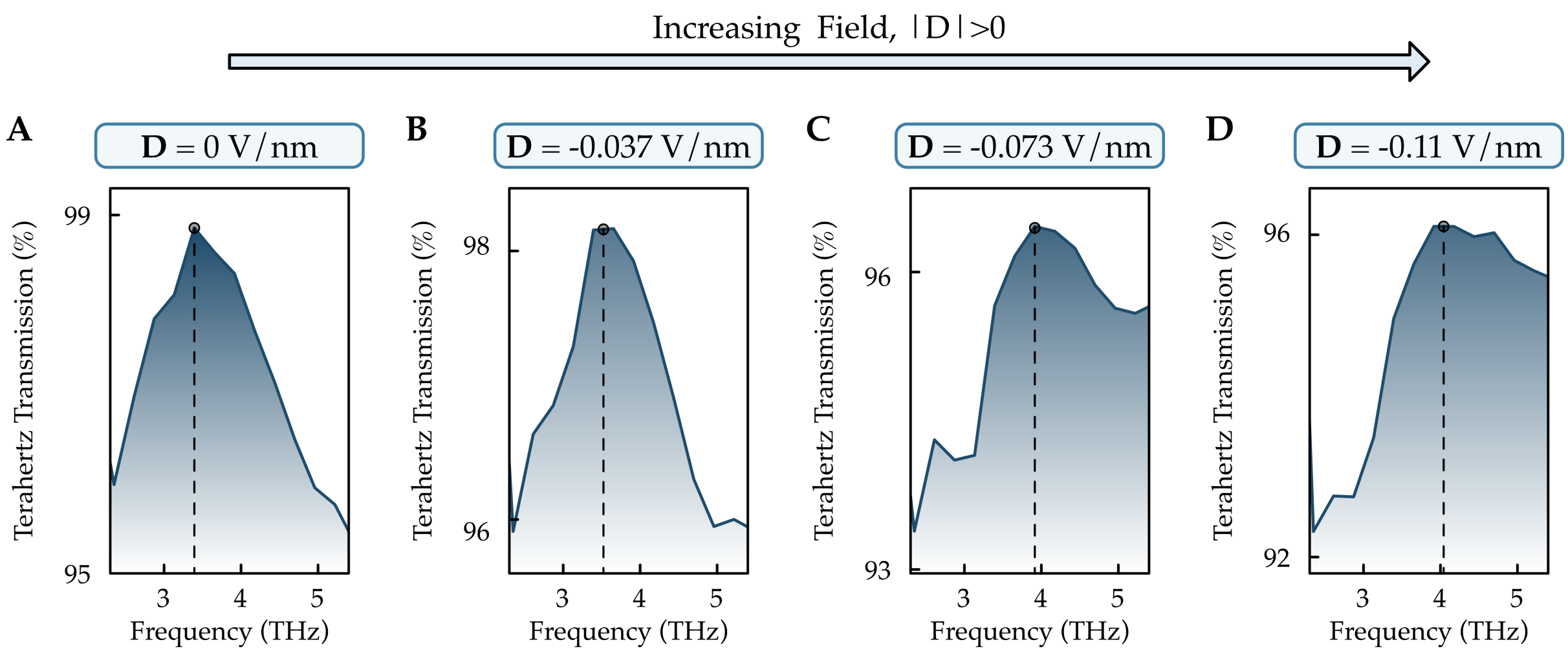

Figure F1: Upper polariton peak as a function of displacement field. The data is adapted from Fig. 5A of the main text. The frequency window is restricted to 2.3–5.4 THz (i.e., from just above the cavity frequency up to near the upper end of our spectral window), thereby removing the LP contribution and excluding the noisy high-frequency tails. The vertical axis is rescaled *independently for each D* (min-to-max within the displayed frequency window) to highlight the UP peak. The broken line highlights the peak position. **A** $D = 0\,\mathrm{V/nm}$. **B** $D = -0.037\,\mathrm{V/nm}$. **C** $D = -0.073\,\mathrm{V/nm}$. **D** $D = -0.11\,\mathrm{V/nm}$.

The quantum modelling (already provided in the first version) and the arguments reported by the authors in the SI and in the response, are also providing a theoretical frame to back up the measurements (Figure 5 and Section 8 SI), and a surely oversimplified, yet convincing explanation (Section 7 SI Figure R13) capable to reproduce the phenomenon.

In details, the more critical comments:

Comment 1: novelty has been explicitly addressed (photon induced emerging of an excitonic resonance); “vacuum fluctuations” ambiguities sorted. The physical mechanism behind these measurements has been discussed extensively with a formal quantum model (not really ”a minimal toy model”) and a semiclassical model.

Comment 2 and 7: Fano resonance. On top of a qualitative discussion the authors attempted

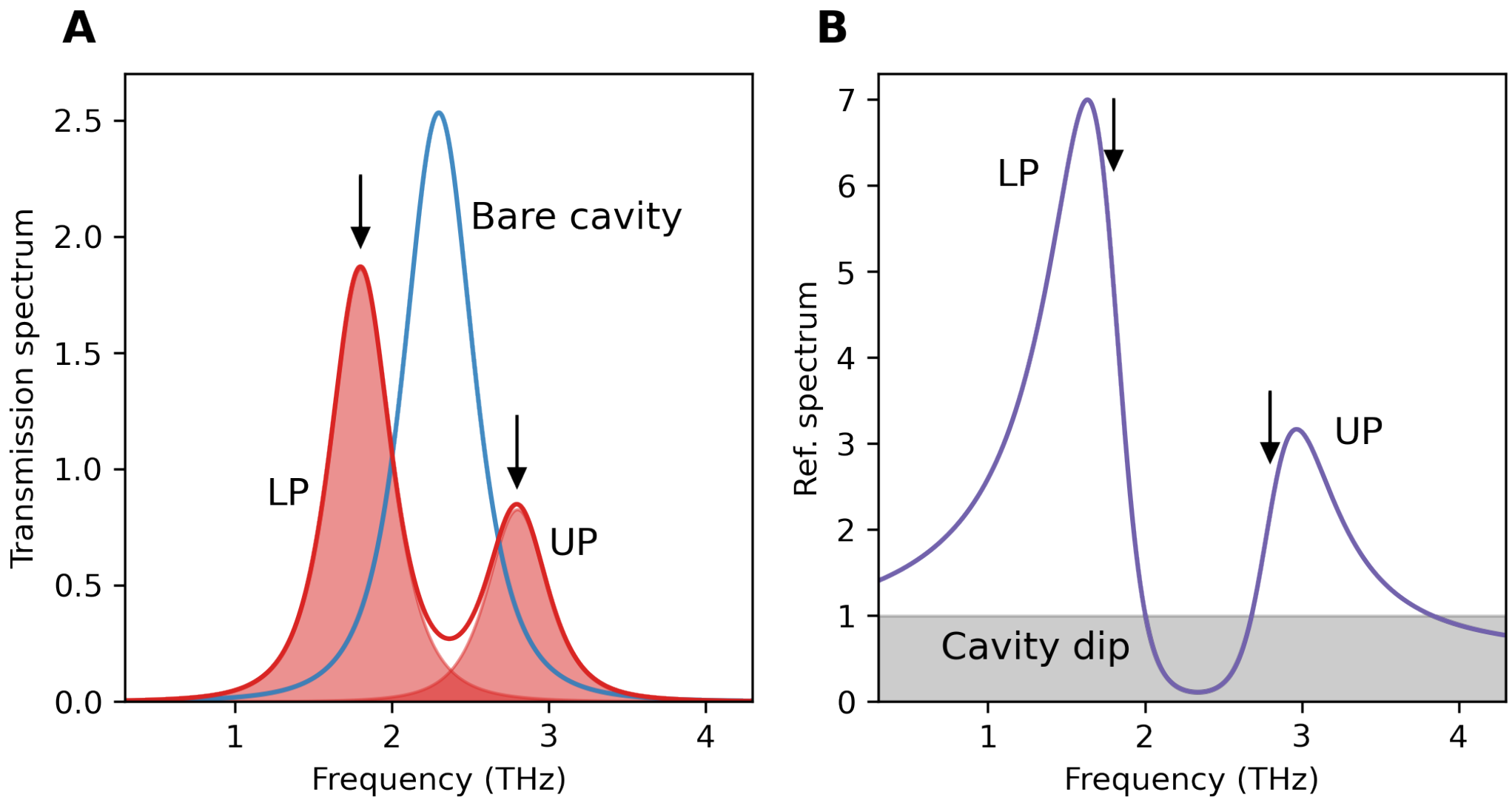

Figure F2: Schematic of the spectral referencing scheme. **A** Lorentzian transmission spectra for a strongly coupled system consisting of LP and UP (red line) as well as for the bare cavity (blue line). The red-shaded areas designate the individual transmission spectra of LP and UP. The arrows mark the LP and UP line positions (i.e. the peak positions). **B** Referenced transmission spectrum obtained by dividing the polariton spectrum (red line in panel A) by the bare cavity spectrum (blue line in panel A). The arrows mark the line positions extracted from panel A. The gray shading highlights transmission below unity, i.e. locations where the bare transmission is larger than the one of the polaritonic system.

also a quantitative modelling of the Fano system (Figure R17). The Fano dispersive feature seems far less convincing in describing what I can see as a clear anti-crossing (e.g Fig 4) which naturally requests the presence of a discrete state rather than a continuum.

Comment 3: Dips vs peaks in Figure 3 and 4. I believe that this is rather a misreading, or a lack of clarity from the authors, on the figures' normalization. The comments about dips, or waveforms' shoulders are in my opinion minor issues, that have been clarified in the revised version.

To address this point we have expanded the explanation of the normalization procedure in the SI. In Fig. F2, we now present an idealized schematic of the referencing scheme, showing explicitly that dividing a polaritonic doublet by the bare-cavity response naturally produces the phenomenology observed in the experiment. The polaritons are physically present as peaks in the raw transmission spectrum [1]; the apparent "dip" between them arises only after normalization and is therefore a direct mathematical consequence of referencing to the uncoupled cavity resonance.

Comment 5: polariton formation mechanism. This was addressed in Fig 5, and section 7 in the SI. The persistence of the UP for $D \to 0$ is also explained.

Comment 6: Temperature trend. Rather than thermally activated carriers' screening (as suggested by the reviewer) which is mainly taken out in the normalization performed at each T, the flattening of the polariton splitting seems more consistent with the (commonly found in literature) T blurring of the spectral linewidths, which ultimately affects the visibility. The explanation on the non-radiative pattern decay -proposed by the authors in the main text- is a viable explanation on the asymmetry between the UP an LP trend. I would have expected a more symmetric behavior according to the reviewer's suggested mechanism.

In general: I have been working (and published) on Fano resonances as well as on the strong coupling regime. I am far more inclined to attribute these findings to the latter case. The measurements on the second sample, with lower S/N, do not shed a new light on the findings, yet are not contradicting the results presented in the manuscript, mainly based on the first sample. I find the data on sample 2 instead corroborating the same narrative and mostly stressing the reproducibility of these complex measurements.

We greatly appreciate the extra time and attention the referee devoted to re-examining the manuscript, our response, and the Supplementary Information, and in particular, their thorough discussion of the concerns raised by Referee 3. We thank the referee for their strong support.

Referee 2 (Remarks to the Author):

The authors have successfully addressed the concerns I raised in my previous submission in a thorough and technically convincing manner. The additional data from the second device, the supplementary analyses and simulations, and the clarifications have significantly strengthened the manuscript. I therefore recommend accepting this manuscript following the minor revisions aimed at improving clarity and presentation outlined below.

1. Although the authors have softened the language surrounding 'vacuum fluctuations' and emphasized 'photon-mediated interactions', it would be helpful to explicitly reiterate in the main text conclusion (not only in the supplementary information) that the interpretation relies on linear-response spectroscopy and a quantum model rather than a direct probe of ground-state pairing.

We agree with the referee that this is an important point to make explicit. We did not intend to state or imply that our measurements provide a direct probe of ground-state pairing. To avoid any possible ambiguity, we have revised the following sentence in the Discussion:

Our observation of photon-mediated bound states in 2D materials raises a broader question: can cavity-driven pairing extend to ground-state collective phenomena in condensed matter?

to

Although our observation of photon-mediated bound states is based on linear-response THz spectroscopy and therefore cannot be taken as direct evidence of ground-state pairing, it raises a broader question: can cavity-driven pairing extend to ground-state collective phenomena?

2. A short intuitive sentence contrasting continuum reshaping with conventional exciton–polaritons could also help non-experts to appreciate why this system is unusual.

We thank the referee for this helpful suggestion. We agree that this contrast can help non-specialist readers appreciate why the present system is unusual. In fact, the revised manuscript already contained a discussion of this distinction in the paragraph beginning "We note that the phenomenology observed here...", where we contrasted conventional ultrastrong coupling to a single discrete resonance with the cavity-induced reshaping of a continuum of interband transitions in our system. Following the referee's suggestion for clarity, and in line with the editor's request to shorten the text, we have streamlined this passage to make the contrast more direct and intuitive. The revised text now reads:

We note that in conventional USC, the observed behavior arises from coupling a cavity mode to a pre-existing discrete *matter resonance, such as an exciton in interband systems [2, 3]. In stark contrast, our system couples the cavity field to a* continuum *of interband electron-hole transitions [4], for which individual oscillator strengths are weak and linewidths are broad, such that well-defined polaritonic modes are not generally expected. The very emergence of polaritons in our experiment therefore suggests a fundamentally different mechanism: as predicted in ref. [5], the cavity field can act as an* effective pairing interaction *that binds electron–hole pairs across a broad momentum range. This photon-mediated*

interaction reorganizes the continuum into a discrete state: the LP (black line in Fig 3D) emerges as a bound-like state "pulled out" of the continuum (the white-hatched area below the gap and cavity lines in Fig. 3D), while in the UP the cavity mode hybridizes with higher-energy transitions (black line in Fig. 3D). Such a continuum-to-discrete reorganization departs significantly from standard USC physics and illustrates the profound ability of cavity electromagnetic fields to mediate attractive interactions in 2D materials.

3. The explanation for the persistence of the upper polariton as the gap closes is now correct and well-motivated from a physical point of view (e.g. the Hopfield weight becoming photonic and the analogy to Landau polaritons). To help non-specialist readers avoid misinterpretation, the authors may wish to consider stating something along the following lines: 'The persistence of the UP at D = 0 does not imply survival of the bound state but rather reflects the cavity-like branch in the ultrastrong-coupling continuation.'

We thank the referee. We agree that this clarification helps prevent over-interpretation, and we now state explicitly in the main text that the persistence of the UP as the gap closes does not imply survival of the bound state, but reflects the increasingly cavity-like branch in the ultrastrong-coupling continuation.

Finally, we consider the UP, whose matter component is predominantly formed by high-energy transitions involving large-momentum states (Fig. 4D). As the bandgap is reduced below the cavity frequency, these continuum states become progressively dressed by cavity photons, as illustrated in Fig. 4F. ***We emphasize that the persistence of the UP at*** $D = 0$***, in both experiment and theory, does not imply that the bound state survives in this limit. Rather, it is the expected continuation of the cavity-like upper branch, entirely analogous to Landau polariton systems, where the UP also remains visible as*** $B \to 0$ ***[6]***

Referee 3 (Remarks to the Author):

The authors have now revised the manuscript to address the concerns raised by the reviewers. However, the revised version still fails to convince the reader about the validity of the experimental results. Below I address the main concerns:

1. To start off Device 2 data in figure R12 makes the case even weaker! I do not understand what the authors mean when they say: ” Despite these disadvantages, the referenced spectra of D2 (Fig. R12) reproduce the key observations of D1: at n = 0 and finite D, we resolve the emergence of two branches and a zero-detuning gap consistent with the coupled-oscillator dispersion”. It is unclear what is observed here that is similar to that of Device !. I am including the two figures below. How does one extract the Rabi splitting from the data of Device 2?

We agree with the referee that the data quality of device D2 is markedly worse than that of device D1, due to the lower transmission and higher disorder, as discussed in the SI and in our previous response. Nevertheless, we remain convinced that the key observations are similar to those of device D1: at $n = 0$ and finite D we still observe the opening of a polaritonic gap (Fig. F3A and B), which becomes more pronounced as the displacement field is reduced (blue region in the colormap). Importantly, this feature is distinct from the bandgap edge observed at large displacement fields: it appears as a dip between two shoulders and is deepest where the bandgap line and the cavity line cross, consistent with an anticrossing scenario. In addition, despite the reduced contrast, we still resolve a feature consistent with an upper polariton (Fig. F3A and B), which is the central observation of our work and is not naturally captured by a conventional Fano picture. The reduced bandwidth and increased noise make the determination of the lower polariton peak position more challenging in device D2; however, they do not affect the central qualitative observation of an opening polaritonic gap. Finally, we clarify that *we did not attempt, nor did we claim*, to extract a Rabi splitting from device D2. While the data from D2 confirm the trend and general behavior of the interaction, we make no quantitative claims.

2. In linecut shown in R16 seems to be taken from the color map of R15. However it is unclear where the upper polariton is in the color map at -0.08 V/nm.

The linecut shown in Fig. R16 is *not* extracted from Fig. R15; it is extracted from Fig. R18, which corresponds to a separate (standalone) measurement included to illustrate that charge injection suppresses the polaritonic features.

More importantly, we understand the concern that the referee has raised repeatedly regarding the visibility of the upper polariton (UP) in the color maps. We realized that some readers

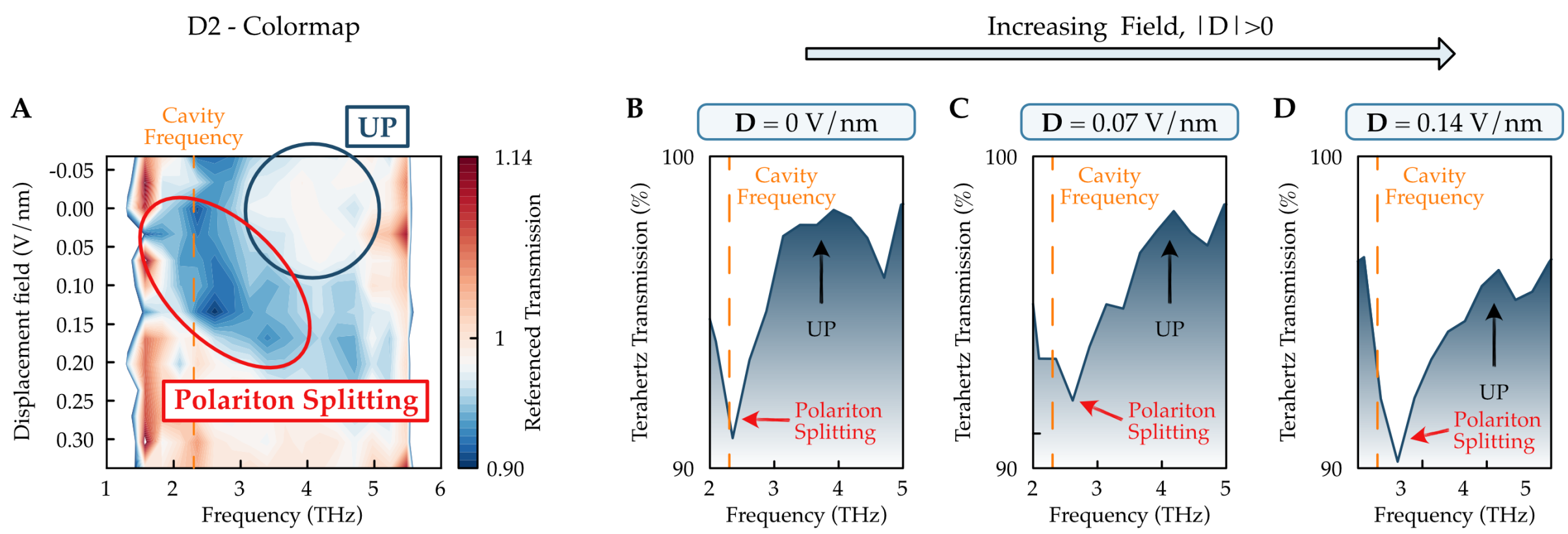

Figure F3: Device 2 Polariton Features A Colormap. **B** Line cuts for three representative displacement fields.

may find high-dynamic-range colormaps (Fig. 3D or Fig.5) or even staggered line plots (Fig. 3A) difficult to interpret. In the colormap representation, the much stronger LP and polaritonic gap dominate the dynamic range, which can make the UP appear visually suppressed when a single common color scale is used. To better highlight the UP, in Fig. F4 we replot the data from Fig. 5A of the main text over the restricted frequency range 2.3–5.4 THz, spanning from the cavity frequency to near the upper limit of our spectral window. This removes the LP contribution and excludes the noisier high-frequency tail, making the evolution of the UP more clearly visible.

For each displacement field D, we rescale the vertical axis (min-to-max within the displayed frequency window) to highlight the UP peak and its dispersive behavior. This presentation avoids the potentially misleading impression that can arise from a colormap with a large dynamic range, where the UP is present but visually compressed relative to the LP.

3. The authors continue to claim that they observe a dispersive upper polariton branch. However, the experimental data does not show that! As we show below how one can easily get the two features: We have illustrated in Fig. A how a transmission dip arising from the cavity mode (as seen in the n=0, D=0 case), combined with a broad peak in transmission (as seen in the n=0, D=-0.32V/nm case), can trivially produce two peaks.

We fundamentally disagree with the premise of the referee's analysis in Comment 3. To challenge our observation of polariton splitting, the referee has constructed a synthetic spectrum (Referee Fig. A) by mathematically summing two distinct referenced spectra taken at different displacement fields—specifically, the transmission at $D = 0$ (semimetallic state) and

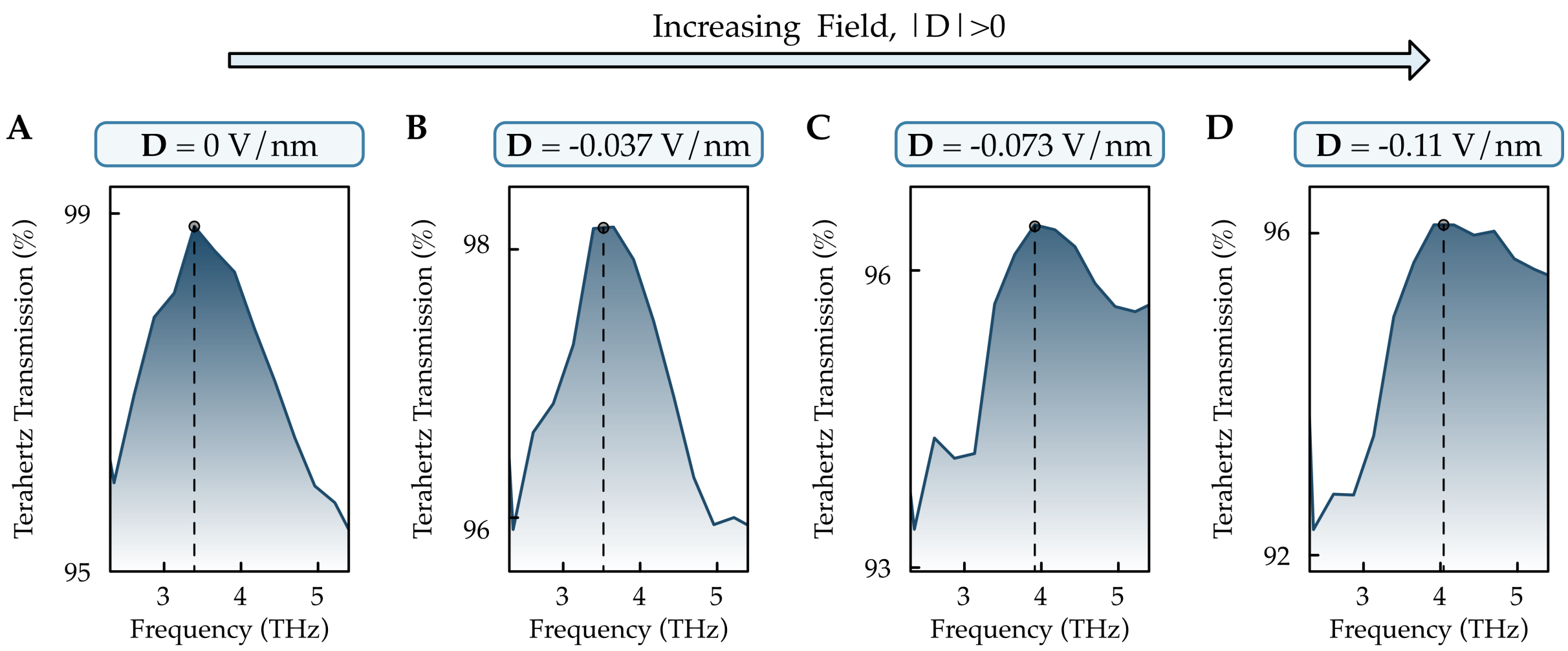

Figure F4: Upper polariton peak as a function of displacement field. The data is adapted from Fig. 5A of the main text. The frequency window is restricted to 2.3–5.4 THz (i.e., from just above the cavity frequency up to near the upper end of our spectral window), thereby removing the LP contribution and excluding the noisy high-frequency tails. The vertical axis is rescaled *independently for each D* (min-to-max within the displayed frequency window) to highlight the UP peak. The broken line highlights the peak position.**A** $D = 0\,\text{V/nm}$. **B** $D = -0.037\,\text{V/nm}$. **C** $D = -0.073\,\text{V/nm}$. **D** $D = -0.11\,\text{V/nm}$.

the transmission at $D = -0.32\,\text{V/nm}$ (gapped state)—and implicitly treating this arithmetic sum as if it could represent our physical measurements.

We find this procedure scientifically unjustifiable. It is neither part of our analysis nor physically meaningful for our experiment. The resulting ”two-peak” (even ”three-peak”?) feature is a mathematical artifact that has no bearing on the physics of our device. We reject this ”addition model” for four specific reasons:

1. **Clear evidence of a dispersing UP.** As already shown in our response to Comment 2 and in Fig. F4, the data display a clear and unambiguous UP peak that changes in position (disperses) as a function of displacement field. We therefore believe that the referee’s concern arises from the visual presentation of the colormap, whose large dynamic range can obscure the prominence of the UP in the raw spectra.

2. **Summing referenced transmission spectra is mathematically and physically invalid.** All data presented in our manuscript represent *referenced transmission spectra*. For each (D, n, T), we record the time-domain THz waveform, Fourier transform it, and divide the resulting spectrum by an in-situ reference taken in the highly insulating configuration ($n = 0$, large $|D|$) at the same temperature. We never add spectra taken at different D (or n). The only averaging we perform is over repeated acquisitions at the *same* gate setting to improve signal-to-noise.

Mathematically, if $\widetilde{T}(D,\omega)$ denotes the referenced transmission,

$$\widetilde{T}(D,\omega) = \frac{T(D,\omega)}{T_{\mathrm{ref}}(\omega)} \tag{1}$$

then the referee's operation corresponds to:

$$\widetilde{T}(D_1,\omega) + \widetilde{T}(D_2,\omega) = \frac{T(D_1,\omega) + T(D_2,\omega)}{T_{\mathrm{ref}}(\omega)}. \tag{2}$$

Summing two such ratios—obtained from entirely different physical states of the material—yields a quantity with no physical meaning. Most importantly, this addition has no electrodynamic interpretation for a single device at an intermediate D. The optical response of a system at an intermediate displacement field D is determined by the specific dielectric function $\epsilon(D)$ of the material at that exact field. It cannot be approximated by the arithmetic sum of the transmission of the system at $D = 0$ and the transmission at D_{max}, as done by the referee.

The referee's operation effectively assumes the system behaves as an incoherent superposition of two independent materials (one metallic, one gapped) measured simultaneously. This corresponds, at best, to an incoherent mixture of transmitted power from two distinct macroscopic regions held simultaneously at D_1 and D_2. That scenario does not apply here: our device is tuned by a single pair of gate voltages, resulting in a single, uniform displacement field across the active region. There is therefore no physical basis for representing an intermediate-D spectrum as the sum of spectra from two extreme D states.

3. **The experimental evidence contradicts a superposition model.** If the observed two-peak structure were simply the trivial sum of a static "cavity dip" and a static "broad peak," as the referee suggests, these features would remain fixed background artifacts. Instead, our data demonstrate highly tunable, resonant behavior. If this were a simple superposition, why would the response depend so strongly on gate tuning? For example, the "cavity dip" should then be present with the same position and comparable intensity in all spectra for all displacement-field values. This behaviour is entirely absent in our data. Why would the peaks instead disperse, shift in frequency, and change their relative intensities precisely when the bandgap is tuned through the cavity frequency?

   In our data (Fig. 4B), the splitting is fundamentally resonant. We do not observe two

peaks at large detuning; rather, we see a single transmission edge that splits only at the anti-crossing condition. The intensity and spectral position of the polariton peaks depend strictly on the detuning, which is wholly incompatible with a static "addition" artifact.

4. **The "broad peak" relies on an isolated boundary effect, not a persistent physical feature.** Finally, we must reiterate that the referee has hand-picked the only trace among those shown in our manuscript that appears to display a "broad peak". As we explicitly demonstrated in our previous response (Fig. F5), the traces adjacent to $D = -0.32$ V/nm clearly exhibit a transmission *edge* corresponding to the tunable bandgap of bilayer graphene, not a broad peak.

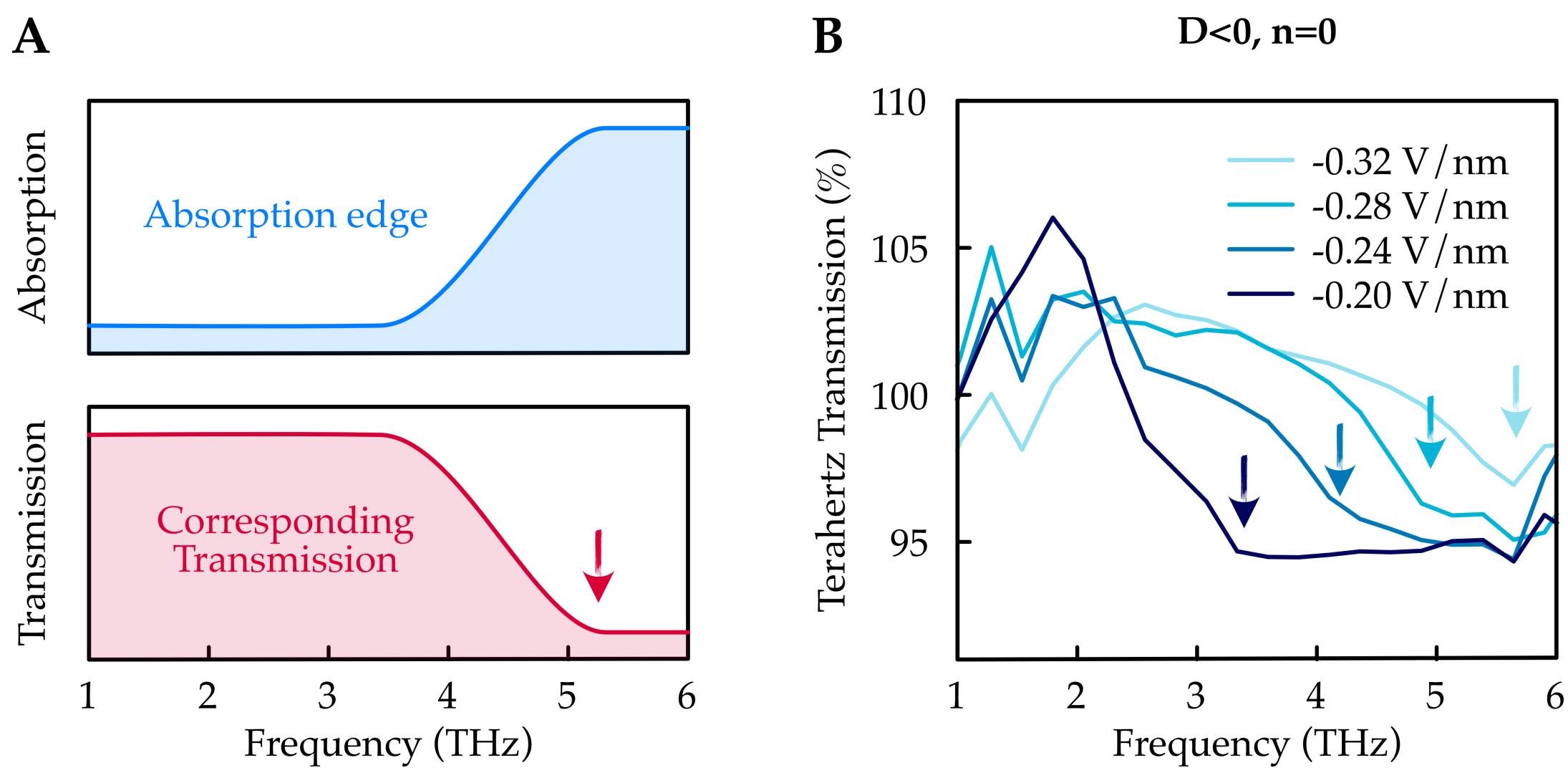

Figure F5: Band edge absorption A Schematic of THz absorption by a band edge (top) and the corresponding transmission spectrum (bottom) with an arrow marking the location of the band edge. **B** Experimental transmission spectra at various displacement fields $|D| > 0$ and $n = 0$ referenced to the insulating state $D = -0.36$ V/nm. The arrows mark the onset of absorption, similarly to panel A. Data obtained from device D1 at 5 K. The line cuts are extracted from Fig. 3A/B of the main text.

This bandgap edge can be mistaken for a "broad peak" only in the highest-field trace selected by the referee ($D = -0.32$ V/nm), and only because of a signal decrease at the low-frequency edge of our acquisition window, where the noise floor is substantially higher. While we cannot assign a definitive physical origin to this low-frequency depression, we emphasize that it is unique to this trace. Building a synthetic spectrum around this isolated, boundary-affected feature, while disregarding the behavior of the surrounding traces, is therefore not a reliable basis for interpreting the data.

In their response, the authors argue that the regime in Fig. 4 is distinct from that in Fig. 3 and that there is no expected transmission dip in the Fig. 4 case (charge-neutral), since it occurs

at n=0 and there is no charge injection. However, even in Fig. 3 for the case of n=0, D=0 (where BLG should be a semimetal), the data clearly show a decrease in transmission coincident with the cavity resonance.

As is clear from Fig.3A,D (reproduced in Fig. F6), even at $n = 0$ and $D = 0$ we still observe a feature consistent with the upper polariton and a remaining polaritonic gap. The decrease in transmission at the cavity frequency noted by the referee is indeed present, and it is due to the hybridization between the cavity mode and the electronic continuum response at charge neutrality; this dip-like lineshape is further emphasized by our referencing/normalization procedure, which enhances relative changes around the cavity resonance. We have already discussed in our previous response how and why we still observe an upper polariton and a related gap even for $n = 0$ and $D = 0$: this is a ubiquitous behavior of ultrastrongly coupled systems, including, for example, Landau polaritons [1, 6].

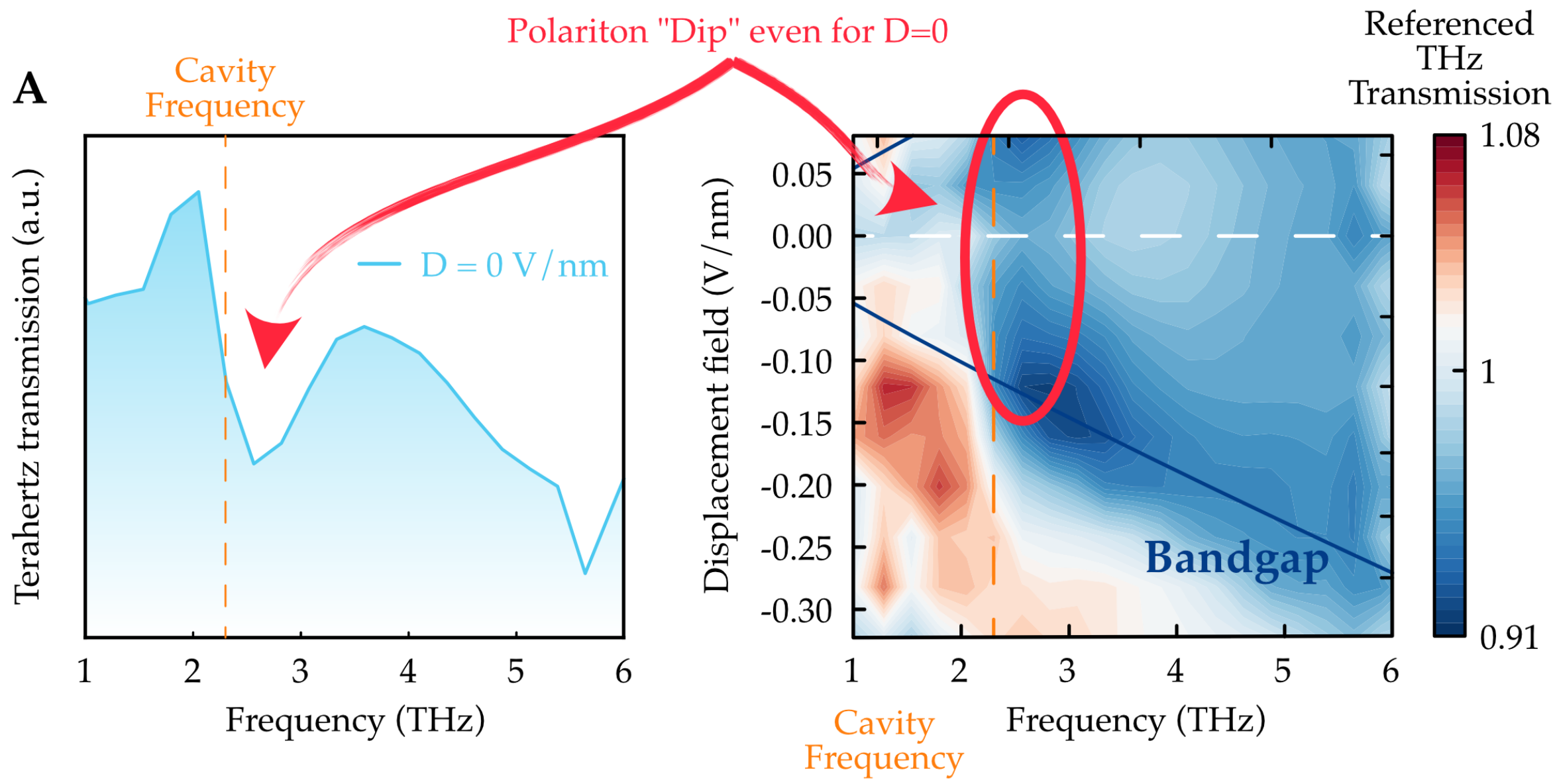

Figure F6: Polariton dip at $D = 0$ adapted from Figure 4 of main text A Line cut. **B** Colormap.

We also stress that the $n = 0$, $D = 0$ data shown in Figs. 2 and 3 correspond nominally to the same measurement point, so the fact that both display a similar cavity-coincident dip is desirable and expected. Of course, the difference between the two data sets arises when moving away from $n = 0/D = 0$: in Fig.2 we vary n at fixed $D = 0$ (carrier injection), whereas in Fig.3 we vary D while keeping $n = 0$ (no intentional carrier injection). We therefore stand by our statement that these two regimes should be considered separately. Finally, we believe we had

already demonstrated this aspect most directly by the additional standalone dataset, showing that the polaritonic features are strongly suppressed once charges are injected into the gapped system, i.e. when both n and D are driven away from zero. We repeat this result here (Fig. F7) because it constitutes the strongest experimental evidence against the referee's interpretation and should resolve any reasonable remaining doubt.

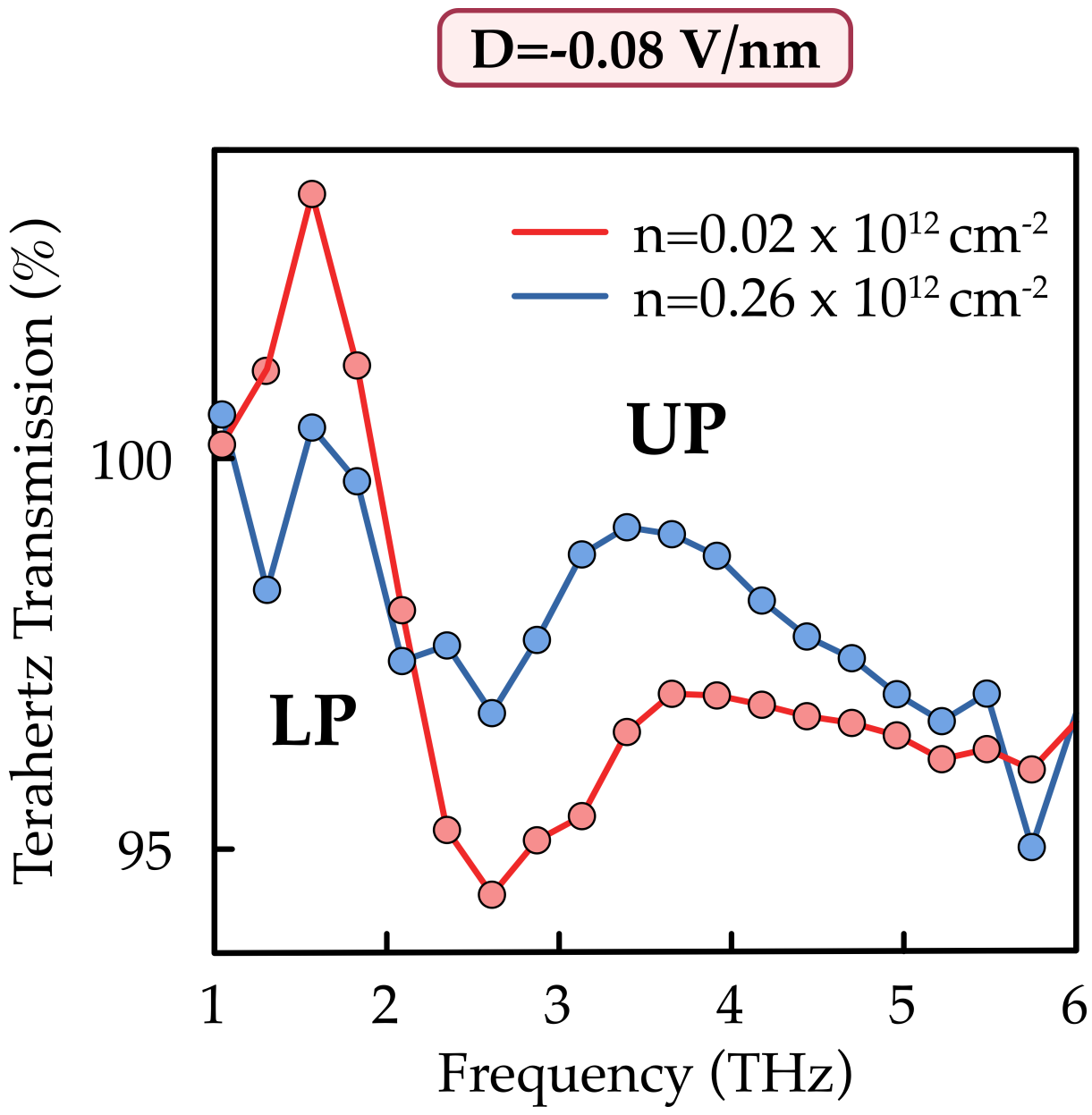

Figure F7: Influence of doping on spectrum at finite displacement field Experimental transmission spectra obtained with n-type doping at finite displacement field $D = -0.08\,\mathrm{V/nm}$. The doping is non-zero for the blue curve and vanishing for the red curve. The spectra are referenced to the insulating state at $D = -0.36\,\mathrm{V/nm}$ and measured at 5 K on D1.

Secondly, if one accepts that the cavity mode is not visible in the gapped case, it is then unclear how two peaks emerge. Even if it comes from the redistributed oscillator strength of the matter mode, the matter mode manifests only as a shoulder (rather than a peak). If the bare cavity mode is not optically visible, it is not evident why the resulting hybrid modes should appear as two peaks rather than two dips, or a dip plus a distortion of the broad background. This point remains insufficiently addressed.

We believe the referee's confusion stems from an intuition based on standard cavities or metallic patch arrays, which typically manifest as dips in transmission. Our device, however, employs a complementary metasurface—specifically, an inverse bow-tie aperture in a metallic screen.

As is well established in terahertz polaritonics [1], the resonant mode of such a complemen-

tary structure acts as a bandpass filter, manifesting as a pronounced *peak* in transmission, not a dip. When this cavity mode strongly couples to a matter excitation, the original single transmission peak splits into two distinct transmission peaks corresponding to the lower and upper polaritons (the hybrid modes, Fig.F8A). Because we are probing the system in transmission, we observe these polaritons through their photonic fraction, meaning they naturally appear as peaks.

The appearance of ”two peaks with a dip in-between” in our data is the direct, mathematically expected result of our *in-situ* referencing procedure. To isolate the effect of the light-matter interaction, we divide the transmission spectrum of the coupled system by the reference spectrum (the highly insulating state, which effectively represents the bare cavity).

Consider the arithmetic of dividing a split-polariton spectrum by a bare-cavity spectrum:

- At the bare cavity frequency (ω_c), the coupled system has a minimum between the two polariton branches (low transmission), while the reference spectrum has its maximum (the bare cavity peak). The resulting ratio is therefore less than 1, creating a prominent relative dip.
- At the polariton frequencies (ω_{LP} and ω_{UP}), the coupled system exhibits transmission maxima, while the reference spectrum has lower transmission at these detuned frequencies. The resulting ratio is therefore greater than 1, yielding two distinct peaks.

In Fig. F8, we present an idealized schematic of this normalization procedure. This demonstrates that dividing a polaritonic doublet by a bare-cavity singlet inherently produces the exact phenomenology we observe. The polaritons are indeed physically present as peaks in the raw transmission [1]; the ”dip” between them is simply the mathematical consequence of dividing by the uncoupled cavity resonance. This fully accounts for the observed lineshapes without requiring any artificial distortion or summation of backgrounds.

More significantly, the experimental signatures do not demonstrate anticrossing. The peak positions in Fig.3B do not consistently follow the black guide line for the purported upper polariton. In fact, a plot of the extracted/quantified positions of two peaks for different D from the experimental data is also missing.

We remind the referee that the black lines in Fig.3B are *not* guides to the eye. As detailed

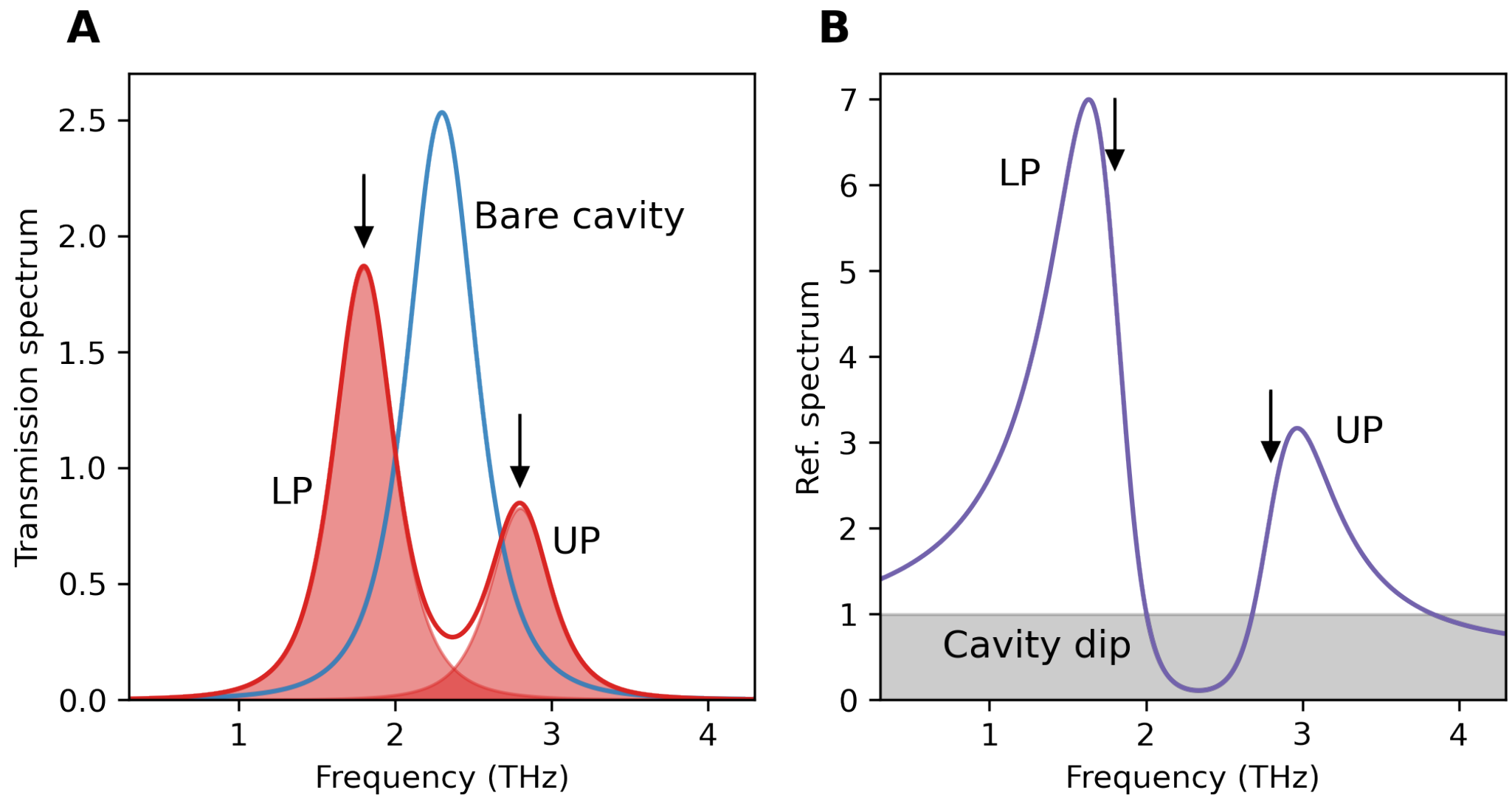

Figure F8: Schematic of the spectral referencing scheme. **A** Lorentzian transmission spectra for a strongly coupled system consisting of LP and UP (red line) as well as for the bare cavity (blue line). The red-shaded areas designate the individual transmission spectra of LP and UP. The arrows mark the LP and UP line positions (i.e. the peak positions). **B** Referenced transmission spectrum obtained by dividing the polariton spectrum (red line in panel A) by the bare cavity spectrum (blue line in panel A). The arrows mark the line positions extracted from panel A. The gray shading highlights transmission below unity, i.e. locations where the bare transmission is larger than the one of the polaritonic system.

extensively in our previous response (see Response to Comment 5 of the previous round), they are obtained from a quantitative comparison of the data with a transfer-matrix model.

To demonstrate directly that this modeling captures the experimental data, we provide a replotted version of the data from Fig.3 (see Fig. F9). To overcome the high dynamic range that visually suppresses the weaker UP branch in a global colormap, we have isolated the LP and UP regions and applied independent color scales to each.

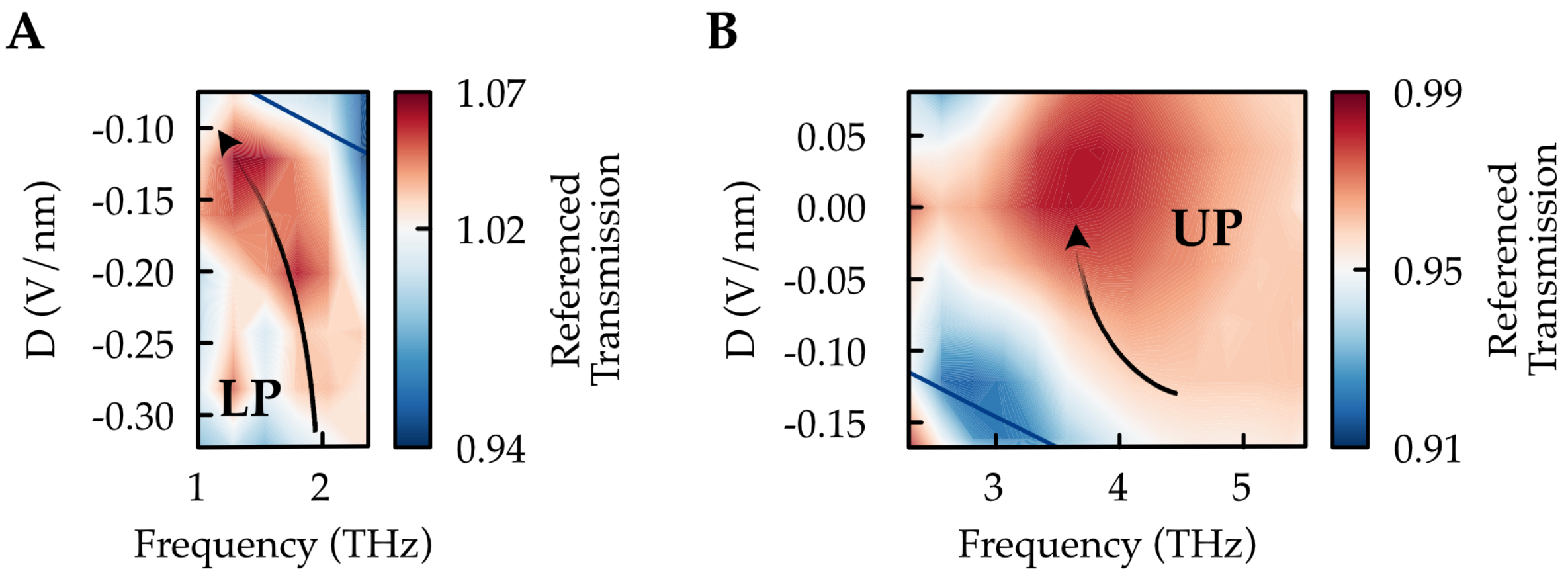

Figure F9: Polariton dispersion for LP and UP, adapted from Figure 4 of main text **A** Lower Polariton dispersion. **B** Upper Polariton dispersion.

Overlaid on this plot are the LP and UP lines obtained from the transfer-matrix modelling. When viewed with this optimized dynamic range, it is clear that the experimental peaks consistently track the modeled UP and LP lines. We believe that the agreement is remarkably close and provides clear evidence for the anticrossing behavior characteristic of ultrastrong coupling.

We would also like to once more point out that the data in Fig.3 is referenced—that is, divided by the strongly peaked bare-cavity spectrum. Mathematically, dividing a double-peaked spectrum (the coupled polaritons) by a strongly peaked function (the bare cavity resonance) inherently shifts the apparent local maxima of the resulting ratio away from the true physical resonance poles (see the arrows in Fig. F8). Consequently, the apparent peaks visible in the normalized 2D map of Fig.3B *cannot and should not* perfectly overlap with the actual energy levels of the lower and upper polaritons.

For this reason, simply extracting and plotting the local maxima from the referenced experimental data, as the referee suggests, would be misleading. The appropriate way to infer the true polariton positions is through comparison with the transfer-matrix model, which captures the full electrodynamics of the coupled system and accounts for the normalization procedure simultaneously.

4. A related concern in Comment 6 also remains unresolved. I argued that the observed temperature dependence could arise from thermally activated carriers screening the THz response. The authors state that referencing each trace in Fig. 6 to an in situ measurement taken at constant D and the same temperature removes temperature-induced effects. This argument is incorrect: the density of thermally activated carriers depends not only on temperature but also on the bandgap, which is being tuned by the displacement field. Referencing to a fixed-D trace therefore cannot eliminate gap-dependent thermal activation effects. The authors' response does not address this physical point.

We agree completely with the referee's physical premise: the density of thermally activated carriers in bilayer graphene depends on both temperature and the displacement-field-tuned bandgap. Consequently, referencing to a fixed-D trace does not—and physically cannot—remove thermal-activation effects that vary with the gap itself. What the normalization does change, however, is the relevant quantity entering the referenced spectrum. Because each trace

is divided by an in-situ spectrum taken at the same temperature but deep in the insulating regime, the measurement is not sensitive to the absolute density of thermally activated carriers, but to the *change* in their THz response relative to that large-gap reference. This effect should be less pronounced at lower temperatures.

However, there appears to be a fundamental misunderstanding regarding what we claimed our normalization procedure achieves. We *never* claimed that referencing removes the intrinsic, temperature-dependent response of bilayer graphene.

As stated in our previous response, the *in-situ* referencing is strictly necessary to remove *extrinsic*, macroscopic temperature-dependent backgrounds. Specifically, at elevated temperatures, the 6.7 mm-thick silicon solid immersion lenses and the silicon substrate generate a massive amount of thermally activated carriers. This creates a severe free-carrier absorption background that dramatically lowers the absolute terahertz throughput of the entire experimental setup. Because this extrinsic silicon absorption is independent of D and n, referencing to an insulating BLG state at the same temperature perfectly divides out the setup's transmission losses, leaving only the intrinsic response of the BLG device.

Regarding the intrinsic response of the BLG itself, we fully agree with the referee that thermally activated carriers play a major role, and we explicitly state this in the manuscript.

As the temperature increases, the spectral contrast decreases: the LP and the spectral dip become progressively less pronounced (Fig. 5E), whereas the UP becomes relatively more prominent (Fig. 5F). This trend is consistent with enhanced dissipation and thermally activated carriers across the gap. These effects preferentially wash out the lower-energy features associated with states near the band edge, while the higher-energy UP remains comparatively less affected (Fig. 4D).

In our previous response (Response to Comment 6), we also specifically wrote:

Thermal population modifies the band occupations that enter the interband dipole matrix element and its phase space, reducing the edge contrast; this weakens the visibility of the polaritons in transmission.

The referee argues that thermally activated carriers screen the THz response, which diminishes the absorption features. This is entirely consistent with our explanation and the referee's

physical point does not contradict our findings; rather, it is the exact mechanism we already invoke to explain the temperature-dependent fading of the ultrastrong coupling features.

5. Minor comment: It will be helpful if the authors stuck to using frequency in X axis (Fig. 4b versus 5b).

We appreciate the referee's suggestion but prefer to retain the current choice. In Fig. 4 of the main text, several different theoretical quantities are plotted as a function of the same tuning parameter, namely the bandgap. We therefore keep the x-axis consistent across all panels to facilitate direct comparison within the figure.

We also intentionally express this tuning parameter in meV rather than converting it to THz. This avoids possible confusion with the frequency axis used for the spectroscopy, which is plotted in THz to allow direct comparison with the experimental data. In other words, keeping the bandgap in meV helps distinguish the control parameter from the spectroscopic frequency axis.

In summary, the theoretical idea is interesting, however, the experimental results fail to support the claims even in the revised version. It appears that there is a simpler explanation for the observations reported. The second device data makes it even weaker! And hence I do not recommend publication of this work in Nature.

References

[1] C. Maissen, G. Scalari, F. Valmorra, M. Beck, J. Faist, S. Cibella, R. Leoni, Ch. Reichl, Ch. Charpentier, and W. Wegscheider. Ultrastrong coupling in the near field of complementary split-ring resonators. *Phys. Rev. B*, 90:205309, 2014.

[2] S. De Liberato. Perspectives for gapped bilayer graphene polaritonics. *Phys. Rev. B*, 92:125433, 2015.

[3] V. G. M. Duarte, P. Ninhos, C. Tserkezis, N. Asger Mortensen, N. M. R. Peres, and A. J. Chaves. Tunable exciton polaritons in biased bilayer graphene. *Phys. Rev. B*, 111:075411, 2025.

[4] N. S. Averkiev and M. M. Glazov. Light-matter interaction in doped microcavities. *Phys. Rev. B*, 76(4):045320, 2007.

[5] J. Khurgin. Excitonic radius in the cavity polariton in the regime of very strong coupling. *Solid State Communications*, 117:307–310, 2001.

[6] G. Scalari, C. Maissen, D. Turčinková, D. Hagenmüller, S. De Liberato, C. Ciuti, C. Reichl, D. Schuh, W. Wegscheider, M. Beck, and J. Faist. Ultrastrong Coupling of the Cyclotron Transition of a 2D Electron Gas to a THz Metamaterial. *Science*, 335(6074):1323–1326, 2012.
